# Robust integration of single-cell datasets with imbalanced modality composition

**DOI:** 10.1038/s41467-026-72933-4

**Published:** 2026-05-14

**Authors:** Qiongyu Sheng, Yang Zhou, Fengping Zhu, Li Xu, Shuilin Jin

**Affiliations:** 1 School of Mathematics, Harbin Institute of Technology, Harbin, China; 2 Department of Neurosurgery, Huashan Hospital, Shanghai Medical College, Fudan University, Shanghai, China; 3 Department of Neurosurgery, The Fourth Division Hospital of Xinjiang Production and Construction Corps, Yining, China; 4 College of Computer Science and Technology, Harbin Engineering University, Harbin, China

**Keywords:** Data integration, Computational models, Statistical methods, Data mining

## Abstract

Single-cell multimodal datasets often exhibit heterogeneous and incomplete modality coverage, posing a challenge for data integration known as mosaic integration. Here, we present Palette, a flexible and interpretable computational framework for mosaic integration of single-cell multimodal data. Palette employs a variant of principal component analysis to disentangle technical noise from biological variation, and leverages the topological structure of the data to accommodate imbalanced modality composition. In systematic benchmarks, Palette consistently outperforms state-of-the-art mosaic integration algorithms, while robustly mixing datasets with various modality compositions. Applied to complex scenarios such as cross-condition and cross-species analyses, Palette preserves meaningful biological signals, enabling the identification of condition-specific cell states and rare subpopulations. We further demonstrate that Palette extends beyond single-cell mosaic integration to accommodate other challenging scenarios. Together, these results position Palette as a robust and versatile framework for harmonizing complex multimodal datasets and facilitating their joint analysis across diverse biological contexts.

## Abstract (web summary)

Single-cell multimodal data integration presents a substantial challenge due to the heterogeneous and incomplete modality compositions. Here, the authors introduce Palette, a flexible and interpretable data integration method, enabling the efficient alignment of complex multimodal datasets.

## Introduction

With advances in single-cell sequencing technologies, it is now possible to profile individual cells across various omics layers, including the transcriptome^1^, epigenome^2,3^, and proteomics^4^. More recently, a suite of multimodal single-cell assays has emerged^5–11^, enabling the simultaneous measurement of distinct modalities within the same cell. Moreover, parallel advances in spatial profiling have extended these capabilities to intact tissue sections, achieving near single-cell resolution of molecular features in situ^12–17^. Together, these developments result in increasingly complex and heterogeneous datasets that span diverse experimental batches, modalities, and spatial contexts. Integrating such multimodal datasets is essential for illuminating the regulatory interplay among different molecular layers. This task, referred to as mosaic integration, aims to harmonize independently generated datasets, each characterized by a set of modalities, into a coherent joint representation^18^. However, it remains challenging due to both cross-modality discrepancies and intra-modality batch effects^19^.

Several computational approaches have recently been proposed for mosaic integration. Among them, variational autoencoder (VAE)-based models, such as totalVI^20^, MultiVI^21^, and Cobolt^22^ have demonstrated success in fusing bimodal measurements. However, their architectures are tailored to specific modality pairs, most notably single-cell RNA sequencing (scRNA-seq) and single-cell assay for transposase-accessible chromatin using sequencing (scATAC-seq) data, constraining both flexibility and scalability^18^. More recent models, including scVAEIT^23^, Multigrate^24^, and MIDAS^25^, extend VAE frameworks to handle multiple modalities. However, these approaches typically require extensive hyperparameter tuning, and their nonlinear latent embeddings can obscure biological interpretability. In contrast, linear integration techniques, such as scMoMaT^26^ and StabMap^27^, offer, in principle, seamless scalability to an arbitrary number of modalities. In practice, however, their performance depends critically on the shared features and the selection of the reference dataset. Moreover, most existing tools overlook heterogeneity in modality composition across datasets, which potentially erodes integration capability.

Here, we present Palette, a flexible computational framework for mosaic integration that operates without relying on explicit biological assumptions. Palette projects cells into a modality-agnostic latent space that preserves biological variation while eliminating technical noise, and supports both supervised and unsupervised integration. To address imbalanced modality composition across batches, Palette constructs a mosaic bipartite graph (MBG) that captures the dataset’s topological structure and propagates information from observed to missing modalities, enabling robust integration across heterogeneous batches. The resulting latent embedding is directly usable for downstream joint analysis, including clustering, cell type annotation, and visualization. Through systematic benchmarking on bimodal and trimodal datasets, we show that Palette consistently outperforms state-of-the-art mosaic integration methods and effectively addresses modality composition heterogeneity across batches. Beyond benchmark tasks, Palette demonstrates strong performance in more challenging settings. In cross-condition and cross-species mosaic integration, it accurately aligns cells while preserving meaningful biological signals. In diagonal integration tasks, Palette effectively leverages linked features across modalities to enable reliable cross-modality knowledge transfer even when there are very few linked features.

## Results

### Overview of Palette

**Fig. 1:**
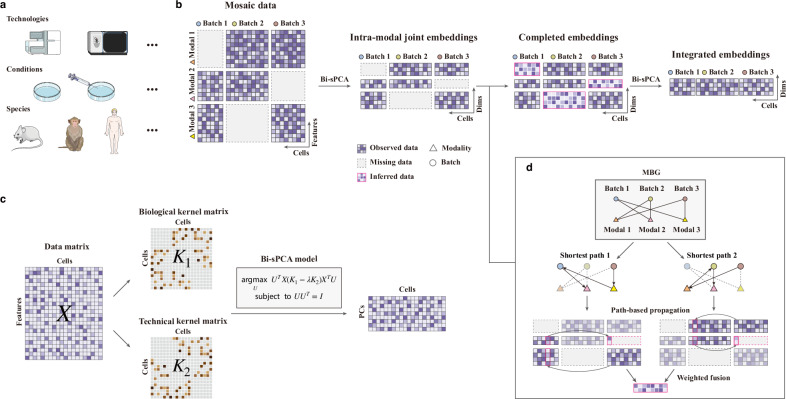
Overview of the Palette mosaic integration framework. **a** Palette supports mosaic data input for different technologies, biological conditions, and species. Two sequencing equipment illustrations were adapted from NIAID NIH BioArt (https://bioart.niaid.nih.gov/). The remaining illustrations were adapted from Servier Medical Art (https://smart.servier.com/), licensed under CC BY 4.0 (https://creativecommons.org/licenses/by/4.0/). Direct links: https://bioart.niaid.nih.gov/bioart/386, https://bioart.niaid.nih.gov/bioart/639, https://smart.servier.com/smart_image/petri-dish-empty/, https://smart.servier.com/smart_image/syringe/, https://smart.servier.com/smart_image/mouse-3/, https://smart.servier.com/smart_image/rhesus-macaque/, and https://smart.servier.com/smart_image/smart-naked-man/. **b** Schematic of the Palette mosaic integration workflow. **c** Diagram of the Bi-sPCA algorithm used to construct a latent space by disentangling biological variation from technical noise. **d** Illustration of the inference of low-dimensional representations for unobserved modalities using the MBG.

Palette is designed to integrate mosaic data, which contains multimodal batches from different sequencing technologies, conditions, or species (Fig. 1a), embedding all cells into a common modality-agnostic low-dimensional space (Fig. 1b and Methods). The framework begins with independently integrating each data modality across batches using bi-supervised principal component analysis (Bi-sPCA), a new dimensionality reduction model aiming to disentangle biological signals from technical effects. Bi-sPCA operates on kernel representations of both biological and technical variation, learning a projection that maximizes biological variation in the embedding while minimizing unwanted technical effects (Fig. 1c and Methods). To avoid the influence of imbalanced modality composition, Palette then infers the missing modality data within batches. Specifically, Palette constructs an MBG to capture the structural topology of data. The MBG is an undirected, unweighted, and connected bipartite graph where nodes represent batches and modalities, and edges indicate the presence of a given modality in a batch. This graph sequentially connects the partially overlapping modalities across batches. For batches that lack measurements in specific modalities, Palette identifies shortest paths in the MBG that connect the batch to the missing modality (Fig. 1d). These paths define biologically informed propagation routes across the latent spaces of the shared modalities, enabling inference of low-dimensional representations for unobserved modalities by transferring information through matched neighbors across batches. Palette employs multiple paths to infer the missing data for each cell in a weighted approach. Finally, both real and inferred representations of all batches are jointly embedded into a unified low-dimensional space using Bi-sPCA again.

In addition to unsupervised integration, Palette supports a supervised mode where cell type labels are incorporated to guide subspace learning and improve alignment of biologically meaningful structures across batches and modalities. To facilitate knowledge transfer from integrated reference data to newly acquired datasets, we further developed a reference-based integration module for palette (Supplementary Fig. 1 and Methods). Given an integrated reference and an unintegrated query, Palette leverages shared modalities as bridges to align them into a common latent space, thereby enabling accurate knowledge transfer and consistent downstream analysis across datasets.

### Palette enables accurate mosaic integration and reference-based integration

We first assessed the integration performance of Palette, compared with five state-of-the-art mosaic integration methods, including both statistical (StabMap and scMoMaT) and deep learning-based approaches (scVAEIT, Multigrate, and MIDAS). We curated five multimodal single-cell datasets spanning both trimodal and bimodal modalities^9,28,29^, constructing six mosaic integration tasks, four trimodal and two bimodal (Supplementary Figs. 2–4 and Methods). Specifically, for trimodal integration, we designed a full-modality single-hop and a multi-hop scenario using the TEA-seq technology data^9^, corresponding to TEA scenarios 1 and 2, respectively. To evaluate integration across different sequencing technologies, we also created two scenarios of single-hop and multi-hop, referred to as bone marrow mononuclear cell (BMMC) scenarios 1 and 2, respectively, by combining two different technologies of the human BMMCs dataset. Additionally, except for BMMC scenario 2, the other three trimodal integration scenarios are designed to handle extensive modality missingness. For bimodal integration, we generated these two scenarios using the retina^28^ and Ab-seq^29^ datasets, respectively. To mitigate sampling bias, each scenario was repeated across five random subsamples, resulting in a total of 30 sub-experiments (Supplementary Data 2 and “Methods”). Following Luecken et al.^30^, we applied eight metrics to assess biological variation conservation and batch effect removal, which were aggregated into a weighted overall integration score (Methods). Moreover, to evaluate the performance in coordinating the heterogeneous modality compositions across batches, we introduced modality composition labels and calculated the modality mixing score (Methods).

**Fig. 2:**
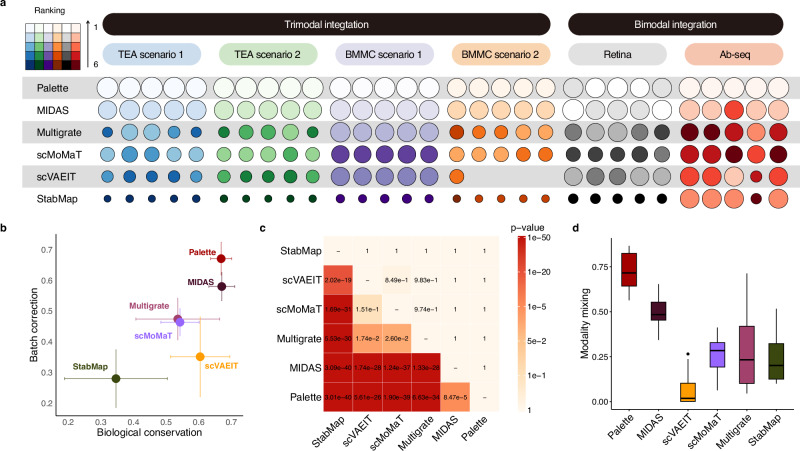
Systematic benchmarking of Palette against state-of-the-art integration methods. **a** Integration ranking across methods on each sub-experiment. The radius of each circle indicates the overall integration scores, ranging from 0 to 1. **b** Scatter plot of mean biological conservation scores against mean batch correction scores for different integration methods. Error bars represent the standard error across the tasks evaluated for each method. **c** Heatmap of one-sided paired Wilcoxon signed-rank test *p*-values. **d** Box plot of overall modality mixing scores across methods. The central lines mark the median values, the boxes show the quartiles, and the whiskers show the rest of the distribution. In our benchmarking, scVAEIT failed to complete four sub-experiments in the BMMC scenario 2 due to training stagnation. For this method, means, medians, standard errors, and *p*-values were calculated based on the remaining 26 completed sub-experiments. Source data are provided as a Source data file.

Across the 30 sub-experiments, Palette consistently demonstrated superior integration performance, achieving the highest overall integration score in 28 cases and ranking second in the remaining two (Fig. 2a and Supplementary Figs. 2–10). Palette showed strong performance across both biological conservation and batch correction, ranking first in batch correction and on par with MIDAS in biological conservation (Fig. 2b, Supplementary Figs. 2b, e, 3b, e, and 4b, e). A one-sided paired Wilcoxon signed-rank test across all integration metrics further confirmed Palette’s overall advantage (Fig. 2c). On the other hand, Palette achieved the highest modality mixing scores across all benchmark tasks (Fig. 2d, Supplementary Figs. 2c, f, 3c, f, and 4c, f), indicating its robustness in harmonizing with diverse modality structures. We then evaluated the integration scenario of imbalanced cell type compositions by randomly removing one cell type per batch across all sub-experiments (see Supplementary Data 3 for detailed cell type information). Under this setting, Palette maintained high performance, indicating its remarkable robustness (Supplementary Figs. 11 and 12). Moreover, we evaluated supervised mosaic integration by incorporating cell type labels as prior information. The supervised mode of Palette consistently outperformed the unsupervised mode across multiple metrics (Supplementary Figs. 13 and 14), underscoring the benefits of leveraging manual annotations, an aspect often overlooked by existing methods. In addition, recent work shows that supervised integration approaches often exhibit sensitivity to variation in the quality or completeness of cell type labels^31^. We therefore systematically examined how annotation perturbations influence supervised integration performance. Quantitative results indicate Palette exhibits stable performance across a range of realistic annotation conditions, including incomplete or noisy labels and different annotation strategies, while maintaining a consistent advantage over unsupervised integration (Supplementary Figs. 15, 16, and Supplementary Note 1).

We next tested Palette’s reference-based integration capabilities. We employed six batches of the human BMMC data, containing both three CITE-seq and three 10x Multiome batches, as the reference. Both unsupervised and supervised integration of Palette were applied, constructing two comprehensive references (Supplementary Fig. 17 and Methods). We curated six additional batches (three CITE-seq and three 10x Multiome batches) from the human BMMC data to generate ten query datasets, containing three unimodal, four diagonal-modality, and three paired multimodal configurations (Supplementary Data 4 and Methods). Palette successfully integrated the reference and query in tasks, effectively mixed the reference and query datasets, and produced clear, separated cell types (Supplementary Fig. 18). We further quantitatively evaluated reference-based integration performance using label transfer accuracy and reference-query mixing (Methods). In addition to the default algorithm, fastMNN^32^, in our reference-based integration framework, we also incorporated two other widely used integration methods, Seurat v3^33^ and Harmony^34^, into the Palette framework to enable a more comprehensive evaluation of reference-based integration performance. We compared the performance of Palette with MIDAS and Multigrate. Quantitative results demonstrate that Palette consistently outperformed MIDAS and Multigrate in label transfer accuracy (Supplementary Fig. 19a), particularly excelling in scenarios involving novel modality compositions in the query data, an area where MIDAS and Multigrate showed performance degradation. In contrast, Palette demonstrated stable and high performance across different modality compositions. Quantitative analysis of reference-query mixing further confirmed Palette’s superiority (Supplementary Fig. 19b). Moreover, a comprehensive assessment using multiple metrics confirmed that Palette’s reference-based integration performance closely approaches that of de novo integration, reinforcing its utility for scalable, modular, and extensible downstream analysis (Supplementary Fig. 20).

### Palette enables the conservation of subtle biological effects

**Fig. 3:**
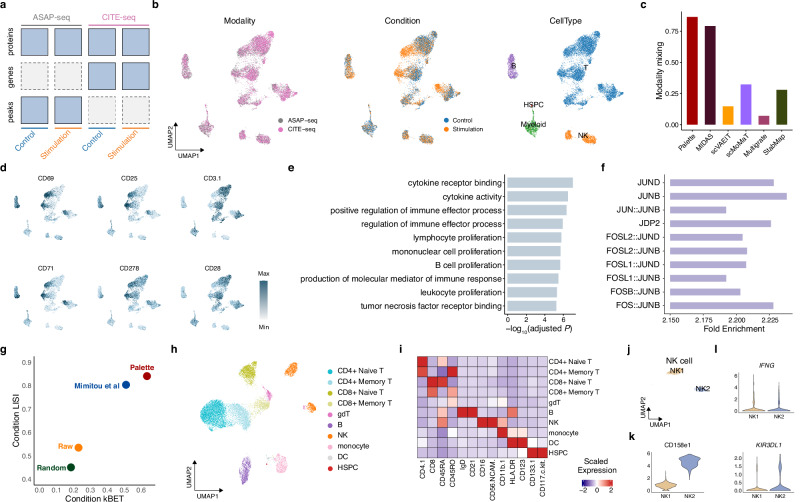
Results on the cross-condition human PBMC dataset. **a** Schematic of the modality composition and biological conditions of each batch in the cross-condition human PBMC dataset. **b** UMAP visualizations of the integrated cell embeddings, colored by modality composition (left), condition (middle), and cell type labels (right), respectively. **c** Comparison of modality mixing score for different integration methods. **d** UMAP visualization of the expression patterns of condition-related differentially expressed proteins. **e** GO enrichment terms for genes upregulated in the stimulation-enriched cluster. The length of the bar represents the -log_10_(adjusted *P* value) from one-tailed Fisher’s test. *P* values were corrected for multiple-hypothesis testing using the Benjamini–Hochberg method. **f** Motifs overrepresented in peaks are upregulated in the stimulation-enriched cluster. **g** Comparison of cross-condition T cell mixing in different data. **h** UMAP visualization of the integrated cell embeddings after removing Palette-identified T cell-specific DEFs, colored by fine-grained cell type labels. **i** The expression patterns of cell type markers on the protein layer. **j** UMAP visualization of NK cell subclusters. **k** Violin plots of CD158e1 expression across subclusters on the protein layer. **l** Violin plots of *IFNG* (top) and *KIR3DL1* (bottom) expression across subclusters on the transcriptome layer. Source data are provided as a Source data file.

Integrating mosaic datasets with batches from different biological conditions is a challenging scenario, as the inherent variation of mosaic integration is confounded with the biological variation introduced by conditions. To test the capability of Palette in such integration, we employed a well-controlled human peripheral blood mononuclear cells (PBMCs) dataset from Mimitou et al.^10^ (Methods), comprising two CITE-seq and two ASAP-seq batches, with each technology containing one control and one T cell stimulated condition (Fig. 3a). Among all tested methods, Palette, MIDAS, and scMoMaT yielded notably better modality mixing in the uniform manifold approximation and projection (UMAP) embeddings, while the remaining methods struggled with batch alignment (Fig. 3b and Supplementary Fig. 21). Quantitatively, Palette achieved the highest modality mixing scores (Fig. 3c).

Palette-integrated result revealed a subtle but consistent stimulation-associated aggregation within T cells (Fig. 3b), suggestive of underlying condition-specific biological variation. To determine whether this pattern reflects true biological effects, we performed unsupervised clustering followed by differential expression (DE) analysis on T cells across all three modalities (Supplementary Fig. 22a and Methods). This analysis identified 84 DE proteins, 1189 DE genes, and 9097 DE peaks, showing concordance with the original study (Fisher’s exact test^35^, *p* < 2e-17; Supplementary Fig. 22b and Methods). Notably, both Palette and Mimitou et al. consistently identified key molecular signatures at the proteomics level, including the upregulation of canonical T cell activation markers (CD69, CD25, CD71, and CD278 proteins) in stimulation-enriched T cells and elevated expression of CD3 and CD28 proteins in control-enriched cells^36–39^ (Fig. 3d). At the transcriptome level, genes such as *CXCR4*, *TFRC*, and *IL2RA* were consistently detected by both analyses (Supplementary Fig. 22c). Gene Ontology (GO) enrichment analysis of genes upregulated in the stimulation-enriched cluster revealed overrepresentation of pathways associated with cytokine signaling, immune effector regulation, and cell proliferation, consistent with previous studies^40,41^ (Fig. 3e). Motif enrichment analysis of differentially accessible chromatin regions identified significant enrichment for binding motifs of the AP-1 and FOS transcription factor families, along with their dimeric complexes (Fig. 3f), corroborating previous reports^42,43^. Moreover, the motif-level DE patterns strongly agreed with those from the original study (Supplementary Fig. 22d).

To test whether the observed condition-specific T cell clustering was driven by these T-cell-specific differentially expressed features (DEFs), we performed a feature ablation study. Specifically, we independently removed an equal number of T-cell-specific DEFs identified by Palette and by Mimitou et al. from each modality. As a control, we also randomly removed the same number of features from each modality. We then integrated these three modified datasets using Palette and evaluated condition mixing of T cells using both k-nearest-neighbor batch effect test (kBET)^44^ and local inverse Simpson’s Index (LISI)^34^ (Supplementary Fig. 22e–g). Removal of T cell-specific DEFs, particularly those identified by Palette, led to an increase in mixing between stimulated and control T cells (Fig. 3g), indicating that these features encode condition-associated T cell state variation. These results indicate that Palette not only supports effective modality alignment but also preserves fine-grained, condition-specific biological variation.

We further investigated whether Palette integration could disentangle condition-associated and condition-shared biological signals. After Palette-identified T cell-specific DEFs removal, we expect the primary effect of feature removal to be confined to T cell stimulation-derived state variation, while major lineage structure among immune populations should remain stable. For this purpose, we annotated T cells into five finer subtypes, with other immune populations largely preserved (Fig. 3h, i, and Supplementary Fig. 23a–c), resulting in a total of ten immune cell subtypes and high correspondence with the original annotations at the level of major immune lineages (Supplementary Fig. 23d, e). Notably, the two natural killer (NK) cell subclusters (NK1 and NK2) remained clearly separable after removing T cell-specific DEFs (Fig. 3j), indicating that lineage-intrinsic heterogeneity unrelated to the stimulation condition was retained. DE analysis revealed that CD158e1 protein (*KIR3DL1* gene), a known inhibitory receptor of NK cytotoxicity^45^, was upregulated in NK2 (Fig. 3k, l), whereas NK1 cells exhibited higher expression of *IFNG*, a key cytokine regulating NK activation function^46^ (Fig. 3l). Moreover, NK1 cells were enriched in immune response and chemokine signaling pathways, whereas NK2 cells showed signatures associated with immune regulation and metabolic processes (Supplementary Fig. 23f). Together, these results demonstrate that Palette effectively integrates heterogeneous multimodal data while preserving biologically meaningful variations, both in terms of global condition-driven responses and fine-scale cell state heterogeneity.

### Palette facilitates cross-species data analysis

**Fig. 4:**
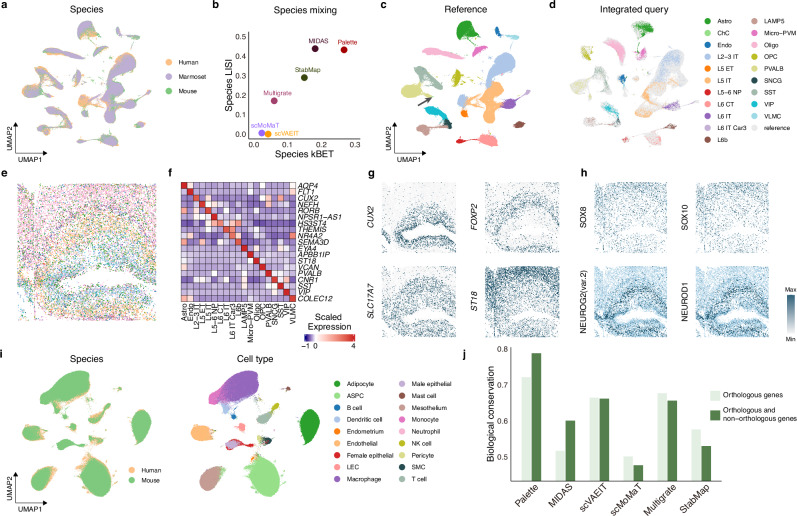
Cross-species integration results. **a** UMAP visualization of integrated cross-species MOp cell embeddings, colored by species. **b** Comparison of species mixing scores across integration methods. **c** UMAP visualization of integrated MOp embeddings, colored by cell type, with the ChCs indicated. **d** UMAP visualization of reference-based integration results, with cell type labels transferred from the reference to the query. **e** Spatial distribution of predicted cell type labels in the query data. **f** Heatmap showing expression patterns of marker genes for the predicted labels. **g** Spatial expression patterns of four marker genes. **h** Spatial activity patterns of four inferred transcription factor motifs. **i** UMAP visualizations of integrated cross-species WAT cell embeddings using both orthologous and non-orthologous genes, colored by species (left) and cell type (right). **j** Bar plot comparing biological conservation scores across integration methods using different gene sets. For each method, the left bar indicates performance using only orthologous genes, while the right bar represents performance using both orthologous and non-orthologous genes. Source data are provided as a Source data file.

We further assessed Palette’s ability to perform cross-species mosaic integration. We assembled a primary motor cortex (MOp) dataset comprising eight batches from human, marmoset, and mouse, spanning both transcriptome and chromatin accessibility modalities^47,48^. The dataset exhibits a pronounced imbalance in cell numbers across modality compositions (Supplementary Data 1), which further complicates coherent cross-modality and cross-species alignment. For the scRNA-seq data, we retained orthologous genes shared across the three species as common features. In contrast, the scATAC-seq data lacked directly matched features across species (Supplementary Fig. 24a), presenting an integration task of greater biological and technical complexity. Integration results revealed that MIDAS, scVAEIT, scMoMaT, and StabMap failed to achieve effective alignment across species and batches (Supplementary Fig. 24b and Methods). In contrast, Palette and Multigrate successfully integrated the data, producing well-mixed embeddings across both species and batches (Fig. 4a and Supplementary Fig. 24b). Quantitative evaluations using kBET and LISI scores for species mixing, batch correction, and modality alignment further demonstrated that Palette consistently outperformed competing methods across the evaluated metrics (Fig. 4b and Supplementary Fig. 24c), underscoring its robustness in addressing cross-species variation, batch effects, and modality heterogeneity. Based on the Palette-integrated embeddings, we identified 20 major cell types (Fig. 4c), with canonical marker genes from each species showing consistent expression patterns within their corresponding cell types (Supplementary Fig. 25). Notably, we detected a rare neuronal subtype, chandelier cells (ChCs), exclusively in the human dataset, with 142 ChCs among 257,943 total cells (~0.055%) (Fig. 4c and Supplementary Fig. 25a, d), highlighting Palette’s sensitivity in identifying rare cell populations.

To evaluate Palette’s reference-based integration capability, we used a slide-tags spatial transcriptomics dataset from the human cerebral cortex^14^ as the query and the integrated cross-species MOp data as the reference. Palette successfully aligned reference and query data (Fig. 4d), and accurately transferred labels from the reference to the query, showing high concordance with the coarse-grained annotations in the original dataset (Supplementary Fig. 26a–d). Moreover, spatial distributions of the transferred labels recapitulated known cortical architecture, confirming the biological validity of the transferred identities (Fig. 4e). Marker genes for each cell type were specifically enriched in the expected populations and spatial domains (Fig. 4f, g), and motif activity inference further revealed cell type-specific regulatory programs (Supplementary Fig. 26e and Methods), supporting the effectiveness of Palette’s reference-based strategy. For example, SOX family motifs were predominantly enriched in oligodendrocytes (Oligo) and endotheliocytes (Endo) (Fig. 4h), while L5 IT and L2-3 IT cells showed strong enrichment of NEUROG2 and NEUROD1 motifs, consistent with previous findings^49–51^.

Most existing cross-species single-modality integration methods typically rely solely on orthologous genes and treat the task as a conventional batch correction problem. However, this strategy overlooks species-specific biological signals encoded in non-orthologous genes. To evaluate the potential added value of incorporating non-orthologous genes, we applied Palette to a cross-species single-cell transcriptome atlas of white adipose tissue (WAT) from human and mouse^52^, comprising 36 batches and 363,870 cells. High-quality cell type annotations provided by the original study enabled supervised integration using Palette. We first performed integration using only orthologous genes and benchmarked Palette against five state-of-the-art mosaic integration methods (Methods), as well as six widely adopted single-modality algorithms spanning both supervised (scANVI^53^, scPoli^54^, and our previously proposed SIGNAL^55^) and unsupervised settings (fastMNN, Seurat v3, and Harmony). We found that supervised methods consistently outperformed unsupervised ones, where Palette successfully integrated the data, ranking first in both integration and species mixing scores (Supplementary Fig. 27 and Methods), underscoring its strong capacity to align cross-species data.

We then extended our analysis by treating orthologous genes as shared modalities and non-orthologous genes as species-specific modalities, constructing a mosaic integration task. Palette-integrated embeddings showed well-mixed batches and separated cell types, achieving the highest overall integration scores and modality mixing scores (Fig. 4i and Supplementary Fig. 28a–d). Incorporating non-orthologous genes improved the performance of Palette, MIDAS, and Multigrate (Supplementary Fig. 28e). However, only Palette and MIDAS demonstrated enhanced biological conservation score under this setting (Fig. 4j). In contrast, the remaining mosaic integration methods exhibited decreased biological conservation performance under this scenario. Palette consistently outperformed all other methods across both integration and biological conservation metrics. Consistent with the supervised setting, Palette also benefited from incorporating non-orthologous genes in unsupervised integration, resulting in improved integration and biological conservation scores (Supplementary Fig. 28f). These results highlight Palette’s unique ability to leverage the information of non-orthologous genes to capture additional biological signals and improve cross-species integration, offering a powerful solution for cross-species integration beyond conventional approaches.

### Palette enables mosaic integration of low-resolution spatial multimodal data

We then evaluated the performance of Palette on the unsupervised mosaic integration scenario for the low-resolution spatial multimodal dataset. We collected data from three sections of human tonsil tissue profiled using the 10x Genomics Visium platform, which enables simultaneous measurement of RNA and surface protein abundance by the sequencing of antibody-derived tags (ADTs)^56^. Due to the platform’s inherent spatial resolution limitations, each spot captures mixed signals from multiple cells. We simulated a realistic mosaic integration scenario by excluding protein and RNA data in the second and third sections, respectively (Supplementary Fig. 29a). The expert manual histological annotations based on histological images were used for quantitative benchmarking (Supplementary Fig. 29b). Among the Palette and the other five competing methods, MIDAS, scVAEIT, and scMoMaT failed to align modalities across tissue sections effectively, resulting in modality-segregated or batch-driven clusters (Supplementary Fig. 29c and Methods). In contrast, Palette, Multigrate, and StabMap produced well-integrated embeddings across sections and modalities (Supplementary Fig. 29c). Notably, Palette consistently outperformed all competitors in biological conservation, batch correction, and modality mixing metrics (Supplementary Fig. 29d, e), demonstrating superior integration performance.

We further identified 11 distinct clusters based on the Palette-integrated data (Supplementary Fig. 29f). Clusters 1 and 7 were marked by high expression of B cell markers, including *CD19*, *MS4A1*, and *IGHD* in the RNA modality^57,58^, as well as PAX5^59^ and CXCR5^60^ in the protein modality, suggesting B cell–rich follicular tissue identity (Supplementary Fig. 29g). Notably, cluster 7 also exhibited elevated expression of *CR2*, *BCL2A1*, and *LMO2* at the RNA level^61–63^, along with PCNA and PDCD1 at the protein level^64,65^, indicating a germinal center (GC) tissue. Clusters 2, 5, and 11 were characterized by high expression of T cell markers, including *CD3E* in both modalities, *CD4* in RNA, and CD8A in protein, suggesting T cell-enriched lymphoid follicular regions. Clusters 4 and 9 exhibited high expression of fibroblast and myofibroblast markers, including *TXNDC5*^66^ in RNA and VIM^67^ and ACTA2^68^ in protein, suggestive of connective tissue identity. In contrast, clusters 3, 6, 8, and 10 exhibited high expression of epithelial markers, such as *KRT5*^69^ in both modalities and *CD9* in RNA, indicating the presence of epithelial tissue.

The spatial distribution patterns of these inferred clusters were highly consistent with the original manual annotations, accurately recapitulating the anatomical architecture of the tonsil tissue (Supplementary Fig. 29h). These results collectively underscore the effectiveness, flexibility, and scalability of Palette, highlighting its applicability to low-resolution spatial datasets with incomplete modality coverage, a common characteristic of most real-world spatial omics experiments.

### Palette enables diagonal integration based on pseudo-modality matrix

Palette employs a connected MBG for mosaic integration. However, in the absence of locally shared modalities, the task is essentially diagonal integration^19^, which disrupts MBG connectivity. Consequently, Palette is unable to perform integration under such conditions. Some algorithms address this by constructing pseudo-modality matrices to introduce linked features^70^ across modalities. For example, deriving a gene activity matrix from scATAC-seq^71^ to integrate with scRNA-seq data. We implemented this strategy by adding pseudo-modality nodes to the MBG, restoring cross-modality links (Supplementary Fig. 30 and Methods).

**Fig. 5:**
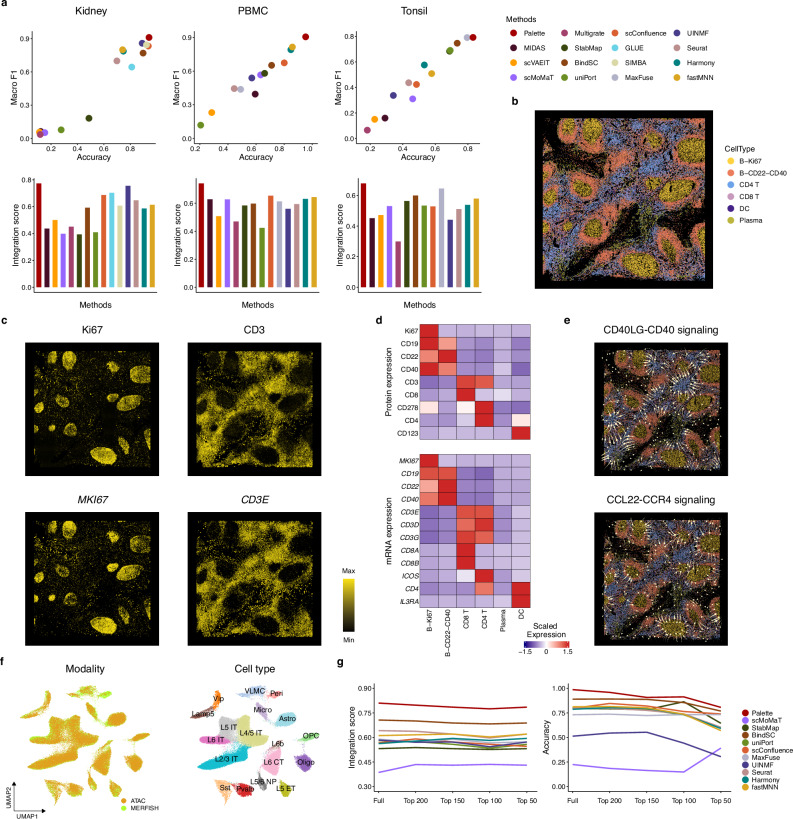
Diagonal integration results. **a** Comparison of label transfer metrics (top row) and overall integration scores (bottom row) across three tasks for different integration methods. **b** Overview of the CODEX human tonsil dataset. **c** Spatial expression patterns of representative markers: protein (top row; Ki-67) and inferred mRNA expression (bottom row; *MKI67*) for a proliferation marker (left); protein (top row; CD3) and inferred mRNA expression (bottom row; *CD3E*) for a T cell marker (right). **d** Expression patterns of selected cell type markers across the protein and inferred mRNA layers. **e** Signaling directionality of CD40LG–CD40 (top row) and CCL22-CCR4 (bottom row) interactions. **f** UMAP visualizations of integrated cell embeddings in the mouse MOp dataset, colored by modality (left) and re-annotated cell type (right). **g** Line plots comparing overall integration scores (left) and label transfer accuracy (right) for different integration methods under reduced feature overlap. Source data are provided as a Source data file.

We evaluated this strategy across three diagonal integration tasks, each involving a pair of unshared modalities from six datasets: (1) scRNA-seq and scATAC-seq data from human kidney^72^, (2) scRNA-seq data^73^ and cytometry by time of flight (CyTOF) proteomics data (30 markers)^74^ from human PBMC, and (3) scRNA-seq data^75^ and co-detection by indexing (CODEX) spatial proteomics data (46 markers)^15^ from human tonsil. We benchmarked Palette against mosaic integration methods as well as six diagonal integration algorithms: (1) the general-purpose methods BindSC^76^, uniPort^77^, and scConfluence^78^; (2) GLUE^79^ and SIMBA^80^, which are designed for strongly linked modalities, such as scRNA-seq and scATAC-seq; (3) and MaxFuse^70^, which targets weakly linked modalities, such as scRNA-seq and proteomic data. We also included three batch integration methods (fastMNN, Seurat v3, and Harmony) and UINMF^81^, which requires globally shared features. Integration performance was assessed by cross-modality label transfer accuracy and overall integration performance. Across all three tasks, Palette consistently outperformed other methods, achieving the highest performance (Fig. 5a and Supplementary Figs. 31–33).

Focusing on the human tonsil integration task, we further assessed whether knowledge transfer based on Palette’s integration results accurately characterizes the immune microenvironment. Annotated CODEX data revealed spatially organized immune architectures of human tonsils, including GCs enriched for B cells with elevated expression of Ki67 (B-Ki67) and surrounding marginal zones containing B cells co-expressing CD22 and CD40 (B-CD22–CD40). T cells were localized primarily to the B-cell follicle border, where they interacted with B cells (Fig. 5b). This interaction plays a central role in the formation and maintenance of GCs^82^. We then inferred transcriptome expression for CODEX data and compiled markers for the major cell types in human tonsils based on previous studies^15,75,83–87^. Key markers exhibited modality-consistent spatial enrichment (Fig. 5c, d, and Supplementary Fig. 34). Moreover, for markers absent from the CODEX antibody panel displayed transcriptome expression patterns matching expected cell type distributions (Supplementary Fig. 35a). DE analysis on both the original and transferred cell type labels for both scRNA-seq data and the CODEX inferred transcriptomics data, followed by a four-way Fisher’s exact test^88^, revealed highly significant DE gene overlaps for all six major cell types (false discovery rate (FDR) < 1e-300 for all comparisons; Supplementary Fig. 35b, c, and Methods), confirming accurate cross-modality alignment and knowledge transfer. We then leveraged the inferred transcriptomics data and spatial coordinates from CODEX data to perform spatial cell-cell communication analysis using COMMOT^89^ and ligand-receptor interactions from the CellPhoneDB database^90^. Notably, we identified CD40LG–CD40 interactions between CD4 T cells and B-CD22–CD40 cells (Fig. 5e and Supplementary Fig. 35d), a critical signaling axis essential for GC formation, B cell activation, and affinity maturation within the tonsil^91^. We also detected CCL22-CCR4 signaling between B cells and T cells, a pathway implicated in T cell recruitment and immune regulation within the GC microenvironment^92^. These findings further highlight Palette’s ability to achieve accurate cross-modality integration.

Finally, we collected scATAC-seq data^93^ and spatially resolved multiplexed error-robust fluorescence in situ hybridization (MERFISH) data (254 genes)^94^ from mouse MOp. Using the gene activity matrix from scATAC-seq and the corresponding expression profiles from MERFISH, we constructed pseudo-modality data comprising 253 linked features shared across both modalities. Palette successfully aligned the modalities, but slight discrepancies exist in the original cell type labels. To standardize labels, we computed silhouette widths (SWs) for MERFISH-profiled cells in the Palette-integrated embedding, treated cells with SW > 0 as high-confidence anchors, and assigned labels to the remaining cells using k-nearest neighbors (kNN) in the integrated embedding (Fig. 5f and Supplementary Fig. 36c). For comparison, we applied the same label assignment process to the other 13 algorithms. Across all methods, Palette-based reannotation achieved the highest adjusted Rand index (ARI) and average silhouette width (ASW) in the low-dimensional space of each modality, surpassing those of the original labels (Supplementary Fig. 36a, b). Hierarchical clustering of reassigned cell type similarities revealed consistent cross-modality concordance (Supplementary Fig. 36d). Moreover, marker expressions were specifically expressed in the corresponding cell types^93,95^ (Supplementary Fig. 36e–g). These results indicate that the cell type reannotated based on Palette integration is reliable. We next benchmarked Palette against the eleven integration algorithms on the mouse MOp dataset using Palette-reannotated labels, with Palette achieving top performance across all metrics (Supplementary Fig. 37). To assess the robustness of Palette under conditions with limited linked features, we ranked the 253 linked features according to their importance for differentiating cell types (Methods). We then generated pseudo-modality data using the top 200, 150, 100, and 50 most informative genes, and applied 11 integration methods that rely on cross-modality feature linkage. Notably, Palette maintained superior performance across all feature sets, demonstrating robustness to the reduction in linked features (Fig. 5g and Supplementary Fig. 38).

## Discussion

In this study, we presented Palette, a flexible and interpretable framework for the mosaic integration of single-cell multimodal data. Palette supports a wide range of integration scenarios and was systematically evaluated across diverse datasets, demonstrating its robustness, accuracy, and scalability.

Mosaic integration poses unique challenges due to both batch effects and heterogeneity in modality composition. Existing methods often fail to address the latter sufficiently, as they generally do not distinguish between the two factors mentioned above. However, batch effects and modality composition heterogeneity are fundamentally different, and conflating them risks suboptimal correction. Palette explicitly models the two factors through dedicated, targeted components. To assess the effectiveness of this design, we introduced modality composition labels and quantitatively evaluated integration performance. The quantitative results confirmed that Palette effectively mitigated modality composition heterogeneity and consistently outperformed state-of-the-art methods.

Benefiting from its modular design, Palette extends beyond mosaic integration to support horizontal, rectangular, and diagonal integration tasks. We first benchmarked Palette against state-of-the-art unsupervised and supervised horizontal integration methods across diverse scRNA-seq datasets^30,96^ (Supplementary Fig. 39 and Supplementary Note 2). As part of this evaluation, the semi-supervised algorithm ssSTACAS and its unsupervised counterpart STACAS^97^ were also included. We further demonstrated Palette’s applicability to horizontal integration in other modalities by visualizing scATAC-seq^30^ and ADT^98^ datasets using UMAP embeddings (Supplementary Fig. 40). Palette’s rectangular integration capability was similarly illustrated on paired single-nucleus RNA-seq (snRNA-seq) and single-nucleus ATAC-seq (snATAC-seq) datasets from the human heart^99^. Finally, we applied Palette to diagonal integration tasks, where it achieved accurate cross-modality alignment across multiple datasets.

For scenarios involving integrated reference and unintegrated query, Palette exploits shared modalities as bridges to perform reference-based integration. This approach prevents modality composition imbalance between the reference and query datasets, ensuring alignment in a comparable feature space. Through the shared bridge modality, Palette maps the query data into the space of the globally integrated reference, allowing it to inherit the biological structure captured across batches and modalities. In addition, the strategy progressively incorporates query datasets into the reference, effectively constructing an expanded embedding. A key advantage of this approach is that, once aligned to the reference, it enables newly aligned datasets to serve as bridges for integrating additional queries. This eliminates the need for repeated de novo mosaic integration, thus offering a scalable solution for large and continuously expanding data collections.

The growing availability of curated cell type annotations in single-cell multimodal datasets has driven recent horizontal integration methods to leverage supervised strategies for improved integration accuracy. However, supervised integration remains largely unexplored in the mosaic integration scenario. To our knowledge, Palette is the first method to explicitly support this. We demonstrated that incorporating such prior knowledge yields more accurate integration than unsupervised approaches. Nevertheless, global cell type annotations may not always be available. One potential solution is to apply automated cell annotation tools to infer missing labels. Moreover, supervised and reference-based strategies in Palette can be combined: a labeled dataset can serve as the reference, to which unlabeled datasets are subsequently aligned via reference-based integration.

## Methods

### Palette

#### Overview

Palette takes as input appropriately preprocessed mosaic datasets with diverse modality compositions across batches, accompanied by batch annotations and, optionally, cell type annotations for each cell. The mosaic integration workflow includes three key steps: (1) intra-modal joint dimensionality reduction, (2) MBG-guided inferring of missing modality matrices, and (3) cross-batch alignment. Moreover, Palette enables reference-based integration, allowing newly acquired mosaic data to align with an already integrated multimodal reference. As output, Palette learns a shared latent representation across batches and modalities, rather than a globally corrected expression matrix in the original feature space. Below, we first introduce the core component of the framework, called bi-supervised principal component analysis (Bi-sPCA), and then detail each step of Palette.

#### Bi-sPCA

Bi-sPCA is an extension of supervised principal component analysis (sPCA) model^100^, a PCA technique grounded in the Hilbert–Schmidt independence criterion (HSIC)^101^. As a supervised dimensionality reduction method, sPCA is particularly suited for classification and regression tasks. We briefly review sPCA here to facilitate the subsequent description of Bi-sPCA.

Given a data matrix $X\in {{\mathbb{R}}}^{d\times n}$ with $n$ samples and $d$ features, and a kernel $K\in {{\mathbb{R}}}^{n\times n}$ derived from the response variable, sPCA aims to identified a subspace in which the projected data is maximally dependent on the response. The objective function of sPCA is formulated as

1 $$\begin{array}{c}{{{\rm{argmax}}}}_{U}{\mbox{HSIC}}\left({\left({U}^{T}X\right)}^{T}{U}^{T}X,K\right)\\={{{\rm{argmax}}}}_{U}\frac{1}{{\left(n-1\right)}^{2}}{\mbox{tr}}\left({X}^{T}U{U}^{T}{XHKH}\right)\\={{{\rm{argmax}}}}_{U}{\mbox{tr}}({U}^{T}{XHKH}{X}^{T}U),\\ {\mbox{subject to}}U{U}^{T}={I}_{l},\end{array}$$

where $U\in {{\mathbb{R}}}^{d\times l}$ is the projected matrix containing the top $l$ eigenvectors of the matrix ${XHKH}{X}^{T}$, $H={I}_{n}-\frac{1}{n}e{e}^{T}$ is the centering matrix, $e\in {{\mathbb{R}}}^{n}$ is a column vector of ones, and ${I}_{n}$ and ${I}_{l}$ are $n$- and $j$-dimensional identity matrices, respectively. In practice, the kernel $K$ is typically computed based on the similarity of response variables.

The sPCA model captures the projection directions of greatest relevance to a target variable. We now consider the unwanted variable that is independent of the variable of interest, proposing the Bi-sPCA model. Bi-sPCA aims to find a linear transformation that maximizes the dependence on a target variable while minimizing the dependence on an unwanted variable. Let ${K}_{1}\in {{\mathbb{R}}}^{n\times n}$ and ${K}_{2}\in {{\mathbb{R}}}^{n\times n}$ denote the kernel matrices encoding similarities in the response variable of interest and the unwanted variable, respectively. The Bi-sPCA objective is formulated as:

2 $$\begin{array}{c}{{{\rm{argmax}}}}_{U}\left({\mbox{HSIC}}\left({U}^{T}X\right){U}^{T}X,{K}_{1}\right)-\lambda \left(\left({\mbox{HSIC}}\left({U}^{T}X\right){U}^{T}X,{K}_{2}\right)\right)\\={{{\rm{argmax}}}}_{U}\left({\mbox{tr}}\left({U}^{T}{XH}{K}_{1}H{X}^{T}U\right)-\lambda {\mbox{tr}}\left({U}^{T}{XH}{K}_{2}H{X}^{T}U\right)\right),\\ {\mbox{subject to}}{U}^{T}U={I}_{l},\end{array}$$

where $\lambda \ge 0$ is a trade-off parameter that balances the relative importance of these two variables. When $\lambda=0$, the model is sPCA. Defining $\widetilde{X}={XH}$ as the row-centered version of $X$, the objective can be further simplified as:

3 $$\begin{array}{c}{{{{\rm{argmax}}}}}_{U}{{\mbox{tr}}}\left({U}^{T}\widetilde{X}\left({K}_{1}-\lambda {K}_{2}\right){\widetilde{X}}^{T}U\right),{{\mbox{subject to}}}\,{U}^{T}U={I}_{l}\end{array}.$$

The solution to Bi-sPCA can be obtained by computing the top $l$ eigenvectors associated with the largest eigenvalues of $\widetilde{X}\left({K}_{1}-\lambda {K}_{2}\right){\widetilde{X}}^{T}$.

#### Intra-modal joint dimensionality reduction

Palette first performs dimensionality reduction independently for each modality on the modality-specific common feature space. When non-globally shared features correspond to distinct biological measurements, Palette allows them to be modeled separately as additional modalities, rather than being forced into the intersected feature space. If a modality is present in only a single batch, standard techniques, such as singular value decomposition (SVD), PCA, or latent semantic indexing (LSI), are applied directly. For modalities that appear in multiple batches, Palette employs Bi-sPCA to project the data into a low-dimensional space, eliminating batch effects while preserving biological variation. The construction of kernel matrices within Bi-sPCA differs depending on the availability of cell type annotations, supporting both supervised and unsupervised modes.

*Case1. Construction of kernel matrices in the supervised mode.* In the supervised setting, cell type annotations are available. First, Palette selects a subset of representative cells from each batch. Specifically, SWs are computed for all cells based on their cell type assignments. Within each cell type, cells are ranked by their SW values, and the top 25% (by default) are selected. By sampling representative cells, Palette is more robust to the potentially mislabeled cell annotation and is more computationally effective than using all cells in subsequent calculations. Second, to define the kernel of biological variation, Palette constructs a cell type co-occurrence matrix ${G}^{m}\in {{\mathbb{R}}}^{{r}_{m}\times {r}_{m}}$ for all representative cells in modality $m\in M$, where $M$ is the set of modalities, and ${r}_{m}$ denotes the total number of representative cells. The elements of ${G}^{m}$ are defined as follows:

4 $$\begin{array}{c}{G}^{m}(i,j)=\left\{\begin{array}{c}\frac{1}{{r}_{m,{b}_{c}}\bullet {r}_{m,{b}_{c}^{{\prime} }}\bullet {Q}_{c}},i\in {S}_{{b}_{c}},j\in {S}_{{b}_{c}^{{\prime} }},\\ \frac{2}{{r}_{m,{b}_{c}}^{2}},i,j\in {F}_{b},\\ 0,{{\mbox{otherwise}}},\end{array}\right.\end{array}$$

where ${r}_{m,{b}_{c}}$ and ${r}_{m,{b}_{c}^{{\prime} }}$ denote the number of representative cells of cell type $c$ in batches $b$ and ${b}^{{\prime} }$, respectively, ${Q}_{c}$ is the number of batches in which cell type $c$ appears, ${S}_{{b}_{c}}$ and ${S}_{{b}_{c}^{{\prime} }}$ denote the set of cells with cell type identity $c$ from batch $b$ and ${b}^{{\prime} }$, respectively, and ${F}_{b}$ denotes the set of cells of cell type $c$ that are exclusive to batch $b$. We define the biological effect kernel ${K}_{{{\mbox{bio}}}}^{m}\in {{\mathbb{R}}}^{{r}_{m}\times {r}_{m}}$ for modality $m$ as the normalized version of ${G}^{m}$ such that the sum of all its entries equals 1:

5 $${K}_{{{\mbox{bio}}}}^{m}=\frac{{G}^{m}}{{\sum }_{i,j}{G}^{m}(i,j)}$$

In our previous study, we found that batch effects varied across cell types^102^. Thus, we finally define a batch effect kernel matrix by connecting cells of the same cell type within the same batch. We construct a matrix ${B}^{m}\in {{\mathbb{R}}}^{{r}_{m}\times {r}_{m}}$, where the elements of ${B}^{m}$ are defined as

6 $${B}^{m}(i,j)=\left\{\begin{array}{c}\frac{1}{{r}_{m,{b}_{c}}^{2}},i,j\in {S}_{{b}_{c}},\\ 0,{{\mbox{otherwise.}}}\end{array}\right.$$

Similar to ${K}_{{{\mbox{bio}}}}^{m}$, we define the batch effect kernel ${K}_{{{\mbox{batch}}}}^{m}\in {{\mathbb{R}}}^{{r}_{m}\times {r}_{m}}$ as

7 $$\begin{array}{c}{K}_{{{\mbox{batch}}}}^{m}=\frac{{B}^{m}}{{\sum }_{i,j}{B}^{m}(i,j)}\end{array}$$

*Case 2. Kernel matrix construction in unsupervised mode.* In the unsupervised setting, where cell type annotations are unavailable, Palette operates as follows. First, within each batch, Palette assigns cluster labels to cells using the Louvain algorithm^103^. Representative cells for each cluster are then selected following the procedure outlined in case 1. We also tested different clustering algorithms and showed that the integration results were robust under this variation (Supplementary Fig. 41). Second, Palette identifies similar clusters across batches for modality $m$. The similarity between cluster ${c}_{i}^{{b}_{1}}$ in batch ${b}_{1}$ and cluster ${c}_{j}^{{b}_{2}}$ in batch ${b}_{2}$ is defined as the median cosine similarity between cells from ${c}_{i}^{{b}_{1}}$ and cells from ${c}_{j}^{{b}_{2}}$ within the low-dimensional space derived via SVD. For cluster ${c}_{i}^{{b}_{1}}$, a similarity value with cluster ${c}_{j}^{{b}_{2}}$ is retained only if it exceeds a threshold $T=\,\max \{{T}_{1},{T}_{2}\}$, where ${T}_{1}$ is a global modality-specific threshold (${T}_{1}=0.6$ for RNA, ATAC, and ADT, and 0.5 for the pseudo-modality matrix by default) and ${T}_{2}=\,\cos ({\theta }_{c}+{\theta }_{m})$ defines an angular similarity constraint. ${\theta }_{c}$ represents the median angle of the cell expression vectors within ${c}_{i}^{{b}_{1}}$. ${\theta }_{m}$ is a modality-specific angular offset (${\theta }_{m}=\frac{{{{\rm{\pi }}}}}{12}$ for RNA and ADT, $\frac{{{{\rm{\pi }}}}}{9}$ for ATAC, and $\frac{{{{\rm{\pi }}}}}{6}$ for pseudo-modality matrix by default). After filtering, each cluster ${c}_{i}^{{b}_{1}}$ retains the matching cluster with the highest retained similarity (if any) from each other batch for kernel construction. Third, Palette constructs a cell state co-occurrence matrix ${G}^{m}$ based on the identified similar clusters. Different from Eq. 4, to avoid incorrect connections between two different cell states, for each cell in cluster ${c}_{i}^{{b}_{1}}$, we randomly set the entries of ${k}_{1}$ cells (${k}_{1}=1$ by default) in its similar cluster ${c}_{j}^{{b}_{2}}$ to 1, and set all other entries to 0. Next, we aggregate all cluster pairs for each batch pair, yielding a batch-pair matrix. This matrix is then normalized by its total connection weight to produce a submatrix of ${G}^{m}$. The full graph ${G}^{m}$ combines all normalized batch-pair matrices and is symmetrized: $({G}^{m}+{({G}^{m})}^{T})/2$. If there are batch-specific clusters, ${G}^{m}$ contains no information about them. Thus, we further define:

8 $${G}^{m}={G}^{m}+{R}^{m},$$

where ${R}^{m}\in {{\mathbb{R}}}^{{r}_{m}\times {r}_{m}}$ is a diagonal matrix with diagonal entries given by

9 $${R}^{m}(i,\,i)=\left\{\begin{array}{cc}v,& i\in F,\\ 0,& \,{{{\rm{otherwise}}}},\end{array} \right.$$

where $v$ denotes the median of the sums of all nonzero rows in ${G}^{m}$, and $F$ denotes the set of cells belonging to clusters without a shared counterpart. Fourth, Palette constructs the biological and batch effect kernel matrices. The biological kernel is constructed as Eq. 5. For the batch effect kernel, we connect each cell only to itself. Moreover, to ensure compatibility in scale between the biological effect kernel and the batch effect component, we define the batch effect kernel as a diagonal matrix, where each diagonal entry corresponds to the row sum of the respective row in ${K}_{{{{\rm{bio}}}}}^{m}$:

10 $${K}_{{{{\rm{batch}}}}}^{m}(i,\,i)={\sum }_{j}{K}_{{{{\rm{bio}}}}}^{m}(i,j).$$

*Joint dimensionality reduction via Bi-sPCA.* For centered and scaled data matrix ${X}^{m}\in {{\mathbb{R}}}^{{p}_{m}\times {n}_{m}}$ of modality $m$, where ${n}_{m}$ denotes the number of cells and ${p}_{m}$ is the number of features, let ${A}^{m}\in {{\mathbb{R}}}^{{p}_{m}\times {r}_{m}}$ be the submatrix of representative cells extracted from ${X}^{m}$. Palette applies the Bi-sPCA model based on ${A}^{m}$:

11 $$\begin{array}{c}{{{{\rm{argmax}}}}}_{{U}^{m}}{{{\rm{tr}}}}({({U}^{m})}^{T}{A}^{m}({K}_{{{{\rm{bio}}}}}^{m}-{\lambda }^{m}{K}_{{{{\rm{batch}}}}}^{m}){({A}^{m})}^{T}{U}^{m}),\\ {{{\rm{subject}}}}\,{{{\rm{to}}}}{({U}^{m})}^{T}{U}^{m}={I}_{{d}^{m}},\end{array}$$

where ${U}^{m}\in {{\mathbb{R}}}^{{p}_{m}\times {d}_{m}}$ contains the top ${d}_{m}$ eigenvectors (${d}^{m}=40$ for RNA, and ${d}^{m}=30$ for ATAC and ADT by default), and ${\lambda }^{m}$ denotes the modality-specific parameter (${\lambda }^{m}=0.8$ for RNA and ADT, ${\lambda }^{m}=0.5$ for ATAC, and ${\lambda }^{m}=0.9$ for pseudo-modality matrix by default). Then, all cells from modality $m$ are projected by: ${Z}^{m}={({U}^{m})}^{T}{X}^{m}$. To alleviate the computational burden of applying Bi-sPCA to datasets with extremely high feature dimensionality, such as scATAC-seq data, Palette incorporates two pre-dimensionality reduction strategies. The first is random projection (RP), a computationally efficient technique that maps the original data matrix to a lower-dimensional subspace using a Gaussian random matrix ${R}^{m}\in {{\mathbb{R}}}^{d\times {p}_{m}}$, where ${p}_{m}$ is the number of features, and $d$ denotes the projection dimension. Based on the Johnson–Lindenstrauss lemma, we set $d$ to $\frac{4\,\log {p}_{m}}{\frac{{\varepsilon }^{2}}{2}-\frac{{\varepsilon }^{3}}{3}}$, where $\varepsilon$ is the distortion tolerance (set to 0.1 by default), and $.$ denotes the ceiling function. The second strategy is LSI, a standard technique widely adopted for scATAC-seq dimensionality reduction. Importantly, Palette achieved consistent integration performance across both strategies (Supplementary Fig. 42), highlighting its robustness to the choice of pre-reduction method.

#### MBG

To represent the modality composition across batches, we construct a MBG, an undirected, unweighted bipartite graph where nodes correspond to either batches or modalities, and an edge connects a batch to a modality if the modality is present in that batch. To ensure the proper operation of the Palette algorithm, the MBG must be connected, meaning that there exists at least one path between every batch and every modality in the dataset. This ensures that information can propagate between all observed and unobserved batch–modality combinations.

#### MBG-guided inferring of missing modality matrices

To infer the low-dimensional representation of a missing modality $m$ of a batch $b$, Palette leverages the MBG to guide information propagation through the following steps:

*Candidate paths enumeration.* We first enumerate all shortest paths in the MBG from batch $b$ to modality $m$. Each path defines a valid sequence of alternating batch and modality nodes, through which shared modality information can be transferred. Formally, a shortest path can be written as: ${p}^{(b\to m)}=(b,{m}_{1},{b}_{1},{m}_{2},\ldots,{b}_{k},m)$, where each pair $({b}_{i},{m}_{l})$ and $({m}_{l},{b}_{i{\prime} })$ corresponds to valid batch–modality or modality–batch edge in the MBG. We define any batch node in ${p}^{(b\to m)}$, excluding the starting batch $b$, as an intermediate batch node. If a path contains exactly one intermediate batch node, it is designated as a single-hop path; otherwise, it is a multi-hop path. A mosaic integration task is classified as a multi-hop task if any multi-hop paths are identified; otherwise, it is considered a single-hop task.

Modality sparsity may potentially influence the effectiveness of path propagation, raising the possibility that a short path relying on sparse modalities could underperform compared with a longer path using denser modalities. To examine this potential trade-off, we evaluated how modality sparsity and path length may interact during propagation and how these factors relate to integration quality (Supplementary Fig. 43 and Supplementary Note 3). The results support the design of the Palette path selection strategy.

*Path selection via nearest neighbor distances.* For each shortest path and each cell $c$ in batch $b$, we identify cross-batch nearest neighbors at each intermediate step for the preparation of path-based propagation. For the first intermediate batch ${b}_{1}$, we compute pairwise distances between cell $c$ in $b$ and cells in ${b}_{1}$, based on the shared modality ${m}_{1}$. The top $q$ ($q=2$ by default) nearest neighbors and their distances are retained. This process is repeated independently for all shortest paths, yielding a pool of nearest-neighbor candidates. Among all candidates, we select the top $q$ paths ${P}_{c}^{(b\to m)}=\{{p}_{ci}^{(b\to m)}|i=1,2,\ldots,q\}$ corresponding to the nearest neighbors to cell $c$, with associated distances ${D}_{c}=\{{d}_{ci}|i=1,2,\ldots,q\}$. Here, ${P}_{c}^{(b\to m)}$ denotes the selected set of propagation paths for cell $c$, and ${D}_{c}$ contains the respective initial distances for these paths. These paths are used to propagate information and infer modality $m$.

*Path-based propagation.* For each selected path, information is propagated iteratively across batches via shared modalities. Starting from the nearest neighbor cell in the first intermediate batch, we identify its nearest neighbor in the next batch based on the next shared modality. This step-by-step process continues until a cell with an observed representation in the target modality $m$ is reached.

*Weighted fusion of representations.* Each selected propagation path yields a valid low-dimensional representation in modality $m$. These $q$ candidate embeddings are then aggregated using a soft weighting scheme based on their initial distances:

12 $${\hat{u}}_{c}^{m}=	 {\sum }_{k=1}^{n}{s}_{ck}\cdot {u}_{{c}_{k}}^{m},\\ {s}_{ck}=	 \frac{{e}^{-{d}_{ck}}}{{\sum }_{j=1}^{n}{e}^{-{d}_{cj}}},$$

where ${\hat{u}}_{c}^{m}$ denotes the inferred low-dimensional representation of cell $c$ in modality $m$, and ${u}_{{c}_{k}}^{m}$ is the observed low-dimensional representation of the final cell reached via the $k$-th propagation path.

We denote the inferred low-dimensional representations of modality $m$ for batch $b$ as ${\hat{U}}^{(m,b)}\in {{\mathbb{R}}}^{{d}_{m}\times {n}_{b}}$. The procedure described above can also be adapted to infer the expression profiles of missing modalities, with a slight modification to Eq. 12, leading to:

13 $${\hat{x}}_{c}^{m}={\sum }_{k=1}^{n}{s}_{ck}\cdot {x}_{{c}_{k}}^{m},$$

where ${\hat{x}}_{c}^{m}\in {{\mathbb{R}}}^{{p}_{m}}$ denotes the inferred expression vector of cell $c$ in modality $m$ with dimensionality ${p}_{m}$, and ${x}_{{c}_{k}}^{m}\in {{\mathbb{R}}}^{{p}_{m}}$ denotes the observed expression vector of the final cell reached via the $k$-th propagation path. To assess Palette’s ability to infer missing modalities, we evaluated its performance on the five benchmark tasks that are available for this analysis (Supplementary Figs. 44, 45, and Supplementary Note 4). The results show that Palette reliably infers missing modalities across these settings.

#### Cross-batch alignment

After inferring the missing modality representations for each batch, Palette integrates the multimodal data by concatenating the low-dimensional embeddings across all batches and modalities. Let ${U}^{m}\in {{\mathbb{R}}}^{{d}_{m}\times n}$ denote the column-stacked low-dimensional representation for modality $m$, which has already been scaled and centered, where $n$ is the total number of cells across all batches. To balance modality-specific variation in the integrated space, we normalize each ${U}^{m}$ by its feature dimensionality: ${\bar{U}}^{m}=\frac{{U}^{m}}{{d}_{m}}$. We then concatenate the normalized low-dimensional representation across all modalities and denote the resulting matrix as $\bar{U}\in {{\mathbb{R}}}^{d\times n}$, where $d$ is the sum of dimensions of the low-dimensional representations across all modalities.

To mitigate potential bias introduced by the modality inference, we apply Bi-sPCA to obtain the integrated embedding. We first construct a global shared nearest neighbor (SNN) graph $G$ based on $\bar{U}$. Edges connecting cells within the same batch are removed. If cell type labels are available for the dataset, we further remove edges that connect cells with different cell types. For each remaining cell and each other batch, we retain the top ${k}_{2}$ (${k}_{2}=5$ by default) edges with the highest SNN weights. The graph is then symmetrized: $G=(G+{(G)}^{T})/2$.

For the biological effect kernel, if every cell in $G$ has at least one edge, we directly define the kernel as ${K}_{{{{\rm{bio}}}}}=G$. Otherwise, we define ${K}_{{{{\rm{bio}}}}}=G+R$, where $R\in {{\mathbb{R}}}^{n\times n}$ is a diagonal matrix with diagonal entry

14 $$R(i,i)=\left\{\begin{array}{c}w,i\in I(G),\,\\ 0,{{{\rm{otherwise}}}},\end{array} \right.$$

where $w$ denotes the median of the row sums of all non-isolated nodes in $G$, and $I(G)$ denotes the set of isolated nodes in $G$. Similar to the definition of the batch effect kernel, we define the bias kernel ${K}_{{{{\rm{bias}}}}}\in {{\mathbb{R}}}^{n\times n}$ as a diagonal matrix whose entries are the row sums of ${K}_{{{{\rm{bio}}}}}$:

15 $${K}_{{{{\rm{bias}}}}}(i,i)={\sum }_{j}{K}_{{{{\rm{bio}}}}}(i,j).$$

The projection vectors are then computed using the Bi-sPCA model:

16 $$\begin{array}{c}\begin{array}{c}{{{{\rm{argmax}}}}}_{V}{{{\rm{tr}}}}({V}^{T}\bar{U}({K}_{{{{\rm{bio}}}}}-\lambda {K}_{{{{\rm{bias}}}}}){\bar{U}}^{T}V),\end{array}\\ {{{\rm{subject}}}}\,{{{\rm{to}}}}{V}^{T}V={I}_{o},\end{array}$$

where $V\in {{\mathbb{R}}}^{d\times o}$ contains the top $o$ eigenvectors ($o=20$ by default), and $\lambda$ is the global parameter ($\lambda=0.5$ by default). Finally, the integrated embedding is calculated using $Z={V}^{T}\bar{U}$.

#### Reference-based integration

Given the reference dataset $X$, with corresponding integrated embedding $Z$, and the unintegrated query dataset $Y$, we assume that each batch in the query dataset shares at least one modality with the reference dataset. Let ${M}_{X}$ and ${M}_{Y}$ denote the sets of modalities in the reference and query datasets, respectively, and let ${M}_{{Y}_{s}}={M}_{Y}\cap {M}_{X}$ be their shared subset. The corresponding submatrix of the query data is denoted ${Y}_{s}$, containing only these modalities in ${M}_{{Y}_{s}}$. For each query batch, we identify reference batches whose modality composition either exactly matches or is a strict subset of that in the query batch. Each shared modality is then independently harmonized using established integration tools, such as fastMNN, Seurat v3, or Harmony. In this study, we adopt fastMNN as the default method. For each query batch $q$, we denote by ${\hat{Y}}_{s}^{(q)}\in {{\mathbb{R}}}^{{d}_{q}\times {n}_{q}}$ the row-stacked matrix obtained by concatenating the harmonized low-dimensional representations of the selected modalities in batch $q$. Similarly, ${\hat{X}}_{q}^{(r)}\in {{\mathbb{R}}}^{{d}_{q}\times {n}_{r}}$ denotes the corresponding matrix of the matched reference batches, where ${d}_{q}$ denotes the dimensionality of the harmonized matrices for the selected modalities in batch $q$, and ${n}_{q}$, ${n}_{r}$ denote the number of cells in the query batch $q$ and the reference set $r$, respectively. To infer a query embedding aligned with the integrated reference space, let ${Z}^{(r)}$ be the integrated embedding of the reference set $r$. We construct two SNN graphs ${G}_{q}^{{{{\rm{ref}}}}}\in {{\mathbb{R}}}^{{n}_{r}\times {n}_{r}}$ and ${G}_{q}^{{{{\rm{query}}}}}\in {{\mathbb{R}}}^{{n}_{r}\times {n}_{r}}$ based on ${Z}^{(r)}$ and ${\hat{X}}_{q}^{(r)}$, respectively. Symmetric normalization is then applied to both graphs using their corresponding degree matrices. The resulting normalized graphs are used as the reference kernel matrix ${K}_{q}^{{{{\rm{ref}}}}}$ and the query kernel matrix ${K}_{q}^{{{{\rm{query}}}}}$, respectively. We subsequently apply the Bi-sPCA model to learn a transformation matrix ${V}^{(q)}$ that captures the components in ${\hat{X}}_{q}^{(r)}$ aligned with the structural variation in the reference embedding. Formally, we solve:

17 $$\begin{array}{c}{{{{\rm{argmax}}}}}_{{V}^{({{{\rm{q}}}})}}{{{\rm{tr}}}}({({V}^{(q)})}^{T}{\hat{X}}_{q}^{(r)}({K}_{q}^{{{{\rm{ref}}}}}-{\lambda }_{q}{K}_{q}^{{{{\rm{query}}}}}){V}^{(q)}{({\hat{X}}_{q}^{(r)})}^{T}),\\ {{{\rm{subject}}}}\,{{{\rm{to}}}}{({V}^{(q)})}^{T}{V}^{(q)}={I}_{{r}_{q}}.\end{array}$$

Then, we project both ${\hat{Y}}_{s}^{(q)}$ and ${\hat{X}}_{q}^{(r)}$ using ${V}^{(q)}$. To infer the query embedding, we fit the linear model: ${({V}^{(q)}{\hat{Y}}_{s}^{(q)})}^{T}={D}^{({{{\rm{q}}}})}{({V}^{(q)}{\hat{X}}_{q}^{(r)})}^{T}+E$, where ${D}^{({{{\rm{q}}}})}\in {{\mathbb{R}}}^{{n}_{q}\times {n}_{r}}$ is the coefficient matrix, and $E\in {{\mathbb{R}}}^{{n}_{q}\times {d}_{q}}$ is the residual error matrix, with elements assumed to be i.i.d. following a normal distribution. The solution is given by ${D}^{(q)}={({V}^{(q)}{\hat{Y}}_{s}^{(q)})}^{T}{({({V}^{(q)}{\hat{X}}_{q}^{(r)})}^{T})}^{{{\dagger}} }$, where ${{\dagger}}$ denotes the pseudoinverse. The inferred embedding of the query batch $q$, denoted by ${\hat{Z}}^{q}\in {{\mathbb{R}}}^{o\times {n}_{q}}$, is given by ${\hat{Z}}^{(q)}={Z}^{(r)}{({D}^{(q)})}^{T}$. Finally, the inferred query embedding is aligned with the global reference embedding $Z$. The resulting harmonized embeddings for the reference and query, denoted by ${Z}^{{{{\rm{ref}}}}}$ and ${Z}^{{{{\rm{query}}}}}$, respectively, serve as unified low-dimensional representations for downstream analyses, including visualization, clustering, and knowledge transfer.

#### Label transfer

We perform label transfer using a kNN approach in Palette-integrated data. Given a labeled dataset $X$ and an unlabeled dataset $Y$, we assign labels to cells in $Y$ based on their nearest neighbors in $X$. Specifically, for each cell in $Y$, we identify its $k$ ($k=5$ by default) nearest neighbors in the low-dimensional embedding learned by Palette and determine its label via majority voting based on the labels of these neighbors.

#### Missing modality inference for query data

For query cells lacking measurements in modality $Y$, we infer their missing profiles by identifying their top $k$ nearest neighbors ($k=5$ by default) in the reference dataset, where modality $Y$ is observed. We then apply a soft weighting scheme described in Eq. 12. The final inferred modality vector for each query cell is computed as a weighted average of the modality $Y$ profiles of its reference neighbors.

#### Parameters

*Bi-sPCA parameter in Palette.* The Bi-sPCA model includes a single regularization parameter, $$\lambda$$. Intuitively, the choice of this parameter is influenced by the sizes of the two kernel matrices. In the Palette algorithm, we account for the scale differences between the two kernels by normalizing them before inputting them into the Bi-sPCA model. However, an improper choice of $$\lambda$$ may affect the stability of the model’s solution. Since Bi-sPCA is solved by computing the eigenvectors corresponding to the top $$d$$ largest eigenvalues in magnitude, the sign of the eigenvalues must also be considered. As $$\lambda$$ increases, the eigenvalues of the matrix $$X({K}_{1}-\lambda {K}_{2}){X}^{T}$$ decrease overall. If $$\lambda$$ is too large, the absolute values of the top $$d$$ largest eigenvalues become very small, making it difficult for the algorithm to converge and resulting in an insufficient number of eigenvectors. To ensure numerical stability, Palette restricts $$\lambda$$ to the range $$[0,1)$$. When $$\lambda=1$$, the matrix $$X({K}_{1}-\lambda {K}_{2}){X}^{T}$$ becomes semi-negative definite, which, as mentioned earlier, prevents the algorithm from guaranteeing a sufficient number of eigenvectors.

*Parameters in Palette.* We use the dataset of the third sub-experiment under the TEA scenario 1, as well as the human BMMC CITE-seq and 10x Multiome dataset, to evaluate the parameter sensitivity of Palette. The investigated tuning parameters include (i) the Bi-sPCA regularization parameter $\lambda$ used for different modalities and integration stages, (ii) the clustering resolution for kernel matrix construction in the unsupervised mode, (iii) the dimensionality settings for intra-modal joint reduction and final embedding, and (iv) the number of propagation paths. The results revealed that Palette is robust to these tuning parameters (Supplementary Figs. 46 and 47).

### Datasets

#### Mosaic integration benchmark datasets

We benchmarked mosaic integration using five multimodal single-cell datasets^9,28,29^: the human PBMC TEA-seq dataset, the human BMMC CITE-seq dataset, the human BMMC 10x Multiome dataset, the human BMMC Ab-seq dataset, and the human retina 10x Multiome dataset. Cell type annotations provided in the original studies were used as ground truth. Except for the human retina 10x Multiome dataset, whose cell type annotations were assigned based on transcriptomic profiles and further validated using the chromatin accessibility modality, all other datasets were annotated using multimodal consensus-based cell type labels. We defined six mosaic integration scenarios by systematically simulating missing modalities through random removal or splitting of batches. To mitigate sampling bias, each scenario was repeated five times with independent random seeds, resulting in 30 sub-experiments (summarized in Supplementary Data 2). Details of each scenario are provided below.

TEA scenarios 1 and 2 were based on the human PBMC TEA-seq dataset, which comprises four batches profiling RNA, ATAC, and ADT simultaneously. For both scenarios, we selected 3994 highly variable genes (HVGs) for the transcriptome, retained 126,817 peaks for chromatin accessibility, and used all 46 surface proteins for the ADT modality. In TEA scenario 1, batches were randomly permuted. The top-ranked batch retained all three modalities, while the second, third, and fourth retained only ATAC, RNA, and ADT, respectively. In TEA scenario 2, the shuffled batches retained RNA and ADT (first), RNA and ATAC (second), ADT only (third), and ATAC only (fourth), respectively.

BMMC scenarios 1 and 2 were constructed using human BMMC CITE-seq and 10x Multiome datasets from the NeurIPS 2021 single-cell multimodal data integration benchmark. The CITE-seq dataset contains three batches (cite-s1d1, cite-s1d2, cite-s1d3; renamed CITE 1, CITE 2, and CITE 3), each jointly profiling RNA and ADT. The 10x Multiome dataset also contained three batches (multiome-s1d1, multiome-s1d2, multiome-s1d3; renamed Multiome 1, Multiome 2, and Multiome 3), each measuring paired RNA and ATAC. For both scenarios, we selected 3547 HVGs for the scRNA-seq data, retained 113,709 peaks for chromatin accessibility, and used all 134 surface proteins for the ADT modality. In the BMMC scenario 1, one of the six batches was randomly omitted. In the BMMC scenario 2, two batches from each technology were selected, and modality pairing was masked by splitting each batch into two subsets, retaining only one modality per subset.

The retina scenario was constructed using the human retina 10x Multiome dataset, comprising eight batches with paired scRNA-seq and scATAC-seq data. We selected 3000 HVGs for the scRNA-seq data, and retained 481,868 peaks for chromatin accessibility. After random permutation, the first three batches retained RNA only, the next three ATAC only, and the final two both modalities.

The Ab-seq scenario was constructed using the human BMMC Ab-seq dataset, comprising six batches from three young and three aged donors, each profiling paired RNA and ADT modalities. We retained all 461 genes for the scRNA-seq data and all 97 surface proteins for the ADT data. In each sub-experiment, two batches were randomly selected from each donor group: one with the RNA modality removed and the other with the ADT modality removed.

#### Human BMMC reference-based integration datasets

Both reference and query datasets were derived from the NeurIPS 2021 single-cell multimodal data benchmark. The global reference was constructed from six batches of human BMMC datasets corresponding to BMMC scenarios 1 and 2. The query set comprised six batches: three CITE-seq (cite-s2d1, cite-s2d4, and cite-s2d5) and three 10x Multiome (multiome-s2d1, multiome-s2d4, and multiome-s2d5) datasets. For feature selection, we adopted the RNA, ATAC, and ADT feature sets previously defined for the corresponding human BMMC CITE-seq and 10x Multiome datasets in our benchmark. To mimic diverse integration challenges, we systematically generated query variants by removing specific modalities, yielding three unimodal, four diagonal-modality, and three paired-multimodal datasets. Detailed dataset configurations are provided in Supplementary Data 4.

#### Cross-condition human PBMC mosaic dataset

We collected four batches of human PBMC multimodal data, including two batches of CITE-seq data and two batches of ASAP-seq data^10^. The two technologies measured paired RNA and ADT data and paired ATAC and ADT data, respectively. In the data sequenced by the two technologies, one batch of data was stimulated with anti-CD3/CD28 and IL-2 for 6 h, called the “stim” group, and the other batch was cultured in the absence of stimulation, called the “control” group. We first performed data quality control and cell type annotation based on the Seurat R package (v4.4.0). In brief, cells were filtered by the criteria nCount_adt > 400 & nCount_adt < 15000 & nFeature_rna > 500 & nFeature_rna < 6000 & nCount_rna > 600 & nCount_rna < 30000 & percent.mt < 10 for CITE-seq data, and nCount_adt > 400 & nCount_adt < 15000 & nCount_atac > 500 & nCount_atac < 3e4 & nucleosome_signal < 2 & TSS.enrichment > 2 for ASAP-seq data. Then, the FindMultiModalNeighbors function was employed on the PCA spaces of RNA and ADT (top 50 components), and the LSI space of ATAC (2–50 components). Next, the function FindClusters was run (with parameters algorithm = 3, resolution = 0.5), followed by manual annotation. A total of five populations were identified (“T”, “NK”, “Myeloid”, “HSPC”, and “dirt”), and “dirt” was removed from subsequent analyses. For feature selection, we selected 2936 HVGs for the scRNA-seq data, retained 99,942 peaks for the scATAC-seq data, and used all 227 surface proteins for the ADT data.

#### Cross-species multimodal MOp dataset

We compiled a cross-species MOp dataset consisting of 257,943 single cells of human, mouse, and marmoset from two studies^47,48^, and the 10x Genomics website. For both human and mouse, the dataset included three batches: one scRNA-seq batch, one scATAC-seq batch, and one multimodal batch with paired scRNA-seq and scATAC-seq data. For the marmoset, two scRNA-seq batches were available. To enable cross-species integration, orthologous genes were identified based on gene annotations and sequence orthology, and 4659 HVGs shared across all three species were selected. For the chromatin accessibility modality, peak features were unified within each species before integration.

#### Human cortex Slide-tags dataset

Transcriptome expression and spatial coordinate data for the human cortex Slide-tags dataset were obtained from Russell et al.^14^. We retained all 17,441 nuclei provided in the original study without additional quality control. To perform reference-based integration, we used the human transcriptomics data from the cross-species MOp dataset as the reference and the Slide-tags dataset as the query, using 3000 HVGs as shared features.

#### Cross-species WAT scRNA-seq dataset

This dataset included 36 batches in total (22 batches from human and 14 batches from mouse), comprising 363,870 cells (66,149 human cells and 297,721 mouse cells), obtained from Emont et al.^52^. We selected 4394 HVGs from the set of orthologous genes shared between human and mouse. In addition, we selected 987 and 633 non-orthologous genes specific to human and mouse, respectively, to be used as species-specific features in subsequent integration.

#### Human tonsil 10x Visium dataset

This dataset comprised three spatial sections from human tonsil tissue, each jointly profiled for scRNA-seq and ADT data using the 10x Visium platform, obtained from Yan et al.^56^. Spots without original annotations were excluded, resulting in a total of 13,305 retained spots. For downstream integration analyses, we selected 3000 HVGs from the scRNA-seq data and all 31 measured proteins from the ADT data.

#### Diagonal integration benchmark datasets

We collected four sets of data for diagonal integration benchmarking experiments, including human kidney data (unpaired scRNA-seq and scATAC-seq data), human PBMC data (unpaired scRNA-seq and CyTOF data), and human tonsil data (unpaired scRNA-seq and CODEX data).

The human kidney data were retrieved from Muto et al.^72^. We used the data expression matrix provided by the original study and the gene activity matrix of scATAC-seq data for integration, treating the original labels as the ground truth. For downstream integration analyses, we selected 2000 HVGs for the scRNA-seq data and retained 96,676 peaks for the scATAC-seq data.

The human PBMC scRNA-seq data from Hao et al.^73^ and the CyTOF data from Hartmann et al.^74^. For scRNA-seq data, we subset one batch (“Batch2”) for subsequent experiments. We selected thirteen cell types at level 2 (“B intermediate”, “B memory”, “B naive”, “CD14 mono”, “CD4 naive”, “CD4 TCM”, “CD4 TEM”, “CD8 TEM”, “ASDC”, “cDC1”, “cDC2”, “pDC”, and “NK”) and reannotated them into six coarse-grained cell types (“B”, “CD14 mono”, “CD4 T”, “CD8 T”, “DC”, and “NK”). For the CyTOF dataset, 50,000 cells were randomly downsampled and clustered using the Seurat R package (v4.4.0). Specifically, clustering was performed with the FindClusters function on the top 15 principal components, using a resolution of 1. A total of seven populations were identified (“B”, “CD14 mono”, “CD4 T”, “CD8 T”, “DC”, “NK”, and “toss”), and “toss” was removed from subsequent analyses. Finally, we selected 2000 HVGs for the scRNA-seq data and retained all 30 CyTOF marker proteins as input features for downstream integration.

The human tonsil scRNA-seq data from King et al.^75^ and the CODEX data from Kennedy-Darling et al.^15^. For two datasets, we directly utilized the processed data as well as the cell type annotations provided by Chen et al.^70^. We selected 2000 HVGs for the scRNA-seq data and retained all 30 CODEX marker proteins as input features for downstream integration.

#### Mouse MOp dataset

We collected scATAC-seq data and MERFISH data from two recent studies^93,94^ in the mouse MOp. For scATAC-seq data, cells were filtered by the criteria nFeature_ATAC > 1000 & nFeature_ATAC < 10000 & nCount_ATAC > 1000 & nCount_ATAC < 15000. And cells annotated as “L2/3 IT”, “Astro”, “L6 CT”, “L6 IT”, “Peri”, “L5 IT”, “Pvalb”, “L5/6 NP”, “micro”, “L4 IT”, “Oligo”, “L5 ET”, “Sst”, “Lamp5”, “OPC”, “VLMC”, “vip”, and “L6b” were retained for subsequent analyses. For MERFISH data, we subset three samples (“mouse1_sample1”, “mouse1_sample2”, and “mouse1_sample3”) from the entire data matrix. Cells annotated as “L2/3 IT”, “astro”, “L6 CT”, “L6 IT”, “peri”, “L5 IT”, “Pvalb”, “L5/6 NP”, “micro”, “L4/5 IT“, “oligo”, “L5 ET”, “Sst”, “Lamp5”, “OPC”, “VLMC”, “vip”, and “L6b” were retained for subsequent analyses. For downstream integration analyses, we retained all 254 genes for MERFISH and 309,344 peaks for chromatin accessibility modality.

For analyses with reduced linked features, we ranked the importance of each feature of classification contribution for MERFISH data. We follow the steps of Chen et al.^70^ to carry out the process. Briefly, we first use all cross-modality corresponding features to train a random forest model (using the function randomForest from the R package randomForest (v4.7.1.2), with default parameters) to predict cell type labels (adjusted by Palette). Then, the function varImp from the R package caret (v6.0.94) with default parameters was employed to calculate the importance score for each feature. Finally, we rank the features based on their importance scores and select the top 200, top 150, top 100, and top 50 most important features, respectively.

#### Horizontal and rectangular integration datasets

We compiled three datasets for single-cell scRNA-seq data integration benchmarking from two studies. The human MTG scRNA-seq dataset from Suresh et al.^96^ comprises five batches and 137,303 cells across 24 cell types. We selected 4,000 HVGs as features and retained all cells without additional quality control. The human immune and pancreas datasets from Luecken et al.^30^, comprise ten batches with 33,506 cells across 14 cell types and nine batches with 16,382 across 16 cell types, respectively. For both datasets, we selected 2000 HVGs as features and retained all cells without additional quality control. The number of HVGs was determined according to dataset complexity, defined by the number of cell types. In addition, we compiled scATAC-seq and ADT datasets to demonstrate Palette’s effectiveness in horizontal integration for modalities beyond transcriptomic data. The scATAC-seq dataset from Luecken et al.^30^, consisting of three batches and 84,813 cells. All 96,924 peaks were used as features, and no additional cell filtering was applied. The ADT dataset from human PBMC was generated using scCUT&Tag-pro, a technology that jointly profiles histone modifications and surface proteins^98^. This dataset included eight batches and 53,542 cells. We used all 173 measured protein markers as features, without further quality control at the cell level.

For rectangular integration analysis, we utilized paired snRNA-seq and snATAC-seq data from the human heart collected by Kanemaru et al.^99^, consisting of ten batches and 106,573 nuclei. We selected the top 3000 HVGs for the snRNA-seq data. For chromatin accessibility, peak calling was performed using fragment data following the authors’ original pipeline (https://github.com/Teichlab/HCA_Heart_ver2/tree/main/ATAC), and peaks located on chromosomes 1–22 were retained for downstream analysis.

### Analysis details

#### Data pre-processing

In the mosaic integration task in this manuscript, we mainly used three modalities: scRNA-seq, scATAC-seq, and ADT. For the scRNA-seq data, we performed standard log-normalization using the NormalizeData function from the Seurat R package. HVGs were identified independently for each batch using FindVariableFeatures. Unless otherwise noted, we used the union of HVGs across batches for downstream analyses (except for the human retina dataset, in which we used Seurat’s SelectIntegrationFeatures function to rank HVGs across batches and selected the top 3000 genes for integration). For scATAC-seq data, we first remove the peaks on the sex chromosomes to ensure that the data is an appropriate input for any algorithm. It is worth mentioning that the Palette algorithm does not inherently require the removal of the peaks on the sex chromosomes. Then, we used the reduce function of the Signac R package (v1.9.0) to merge all retained peaks across batches to obtain a new set of peaks and recalculated the peak by cell count matrix using the new peaks. We recalculated the peak sets in human PBMC TEA-seq scATAC-seq, human retina scATAC-seq, and cross-species MOp scATAC-seq data. For the other scATAC-seq data, we used the original peak sets provided by the authors. Next, we calculate the inverse document frequency (IDF) for each peak in batches. IDF for peak $i$ in batch $j$ was defined as ${{{{\rm{IDF}}}}}_{ij}={N}_{j}/{n}_{ij}$, where ${N}_{j}$ was the total number of cells in batch $j$ and ${n}_{ij}$ was the sum of counts for peak $i$ across cells in batch $j$. We multiply the count value of each peak by the corresponding IDF and employ the normalize data function of the Seurat R package. For ADT data, we kept all features as input and performed centered log ratio transformation using the Seurat R package.

In the diagonal integration task in this manuscript, we used data from five modalities, including scRNA-seq, scATAC-seq, CyTOF, MERFISH, and CODEX. For scRNA-seq data, we followed the same preprocessing steps as the mosaic integration task, first normalizing and then selecting HVGs. For scATAC-seq data, we kept all peaks as input and normalized them using RunTFIDF in the Signac R package. For CyTOF data, we kept all features and performed centered log ratio transformation using the Seurat R package. For MERFISH data, we kept all features and performed standard normalization procedures using the NormalizeData function from the Seurat R package. For CODEX data, we directly used the normalized data provided by Chen et al.^70^.

For pseudo-modality matrix construction, we first identify a set of features from each of the two modality datasets. These two sets of features are connected one-to-one, such as proteins and their coding genes, and gene activity in ATAC data and corresponding genes. Next, we performed quantile normalization on the sub-matrices from both modalities. To improve signal-to-noise ratio, we performed kNN smoothing on the sub-matrices of human PBMC scRNA-seq, human tonsil RNA, and mouse MOp scATAC-seq data.

#### Benchmark data integration methods

We benchmarked Palette integration against fourteen data integration methods. scVAEIT^23^, Multigrate^24^, scMoMaT^26^, scANVI^53^, scPoli^54^, uniPort^77^ scConfluence^78^, GLUE^79^, SIMBA^80^, and MaxFuse^70^ were executed using the Python packages “scVAEIT” (v1.0.2), “multigrate” (v0.0.2), “scmomat” (v0.2.2), “scvi” (v1.0.4), “scarches” (v0.5.9), “uniport” (v1.3), “scconfluence” (v0.1.1), “scglue” (v0.4.0), “simba” (v1.2), and “maxfuse” (v0.0.2), respectively. MIDAS^25^ was executed using the Python script provided at https://github.com/labomics/midas/tree/reproducibility/. StabMap^27^, UINMF^81^, SIGNAL^55^, BindSC^76^, Seurat v3^33^, Harmony^34^, and fastMNN^32^ were executed using the R packages “StabMap” (v0.1.8), “rliger” (v2.0.1), “SIGNAL” (v1.0.0), “bindSC” (v1.0.0), “Seurat” (v4.4.0), “Harmony” (v1.2.0), and “batchelor” (v1.14.1), respectively. All methods were benchmarked using 20-dimensional integrated embeddings. For Seurat, canonical correlation analysis (CCA) was used as the default integration mode; reciprocal PCA (RPCA) was used when CCA failed due to dataset size constraints. Except where noted, all methods were run with their default integration parameters. To ensure consistency, we aimed to provide all methods with identical input features. However, due to memory constraints, additional feature selection was required in specific scenarios. For scATAC-seq data, we selected the top 50,000 highly variable peaks for Multigrate in BMMC scenario 2 and the Retina scenario, scMoMaT in the Retina scenario, and scVAEIT in TEA scenarios 1 and 2, as well as the Retina scenario. For StabMap in the Retina scenario, peaks were selected according to its official tutorial, yielding approximately 31,000 peaks. Similarly, for UINMF on the human kidney and mouse MOp datasets, we selected the top 2000 peaks as per its recommended preprocessing workflow. Moreover, we evaluated Palette under a limited feature size setting, in which the number of input features was restricted to the minimum used within each evaluation scenario (Supplementary Figs. 48 and 49). Additionally, we determined the reference dataset for StabMap based on the following principles: in mosaic integration, the dataset with the most comprehensive modality was chosen as the reference, while in diagonal integration, the dataset with the largest number of cells was selected.

#### Cross-condition human PBMC mosaic integration analysis

We used the integration results of the Palette method for clustering. In T cells, the clusters were divided into a ‘control’ enriched group and a “stimulation” enriched group. Next, we used the script provided by Mimitou et al.^10^ (https://github.com/caleblareau/asap_reproducibility) to perform differential expression analysis on ATAC, RNA, and ADT, respectively. Then, for each modality, the Palette differential expression features (DEFs) were identified using the following criteria: FDR < 0.01 and the absolute value of log fold change > 0.5 for ATAC; FDR < 0.01 and the absolute value of log fold change > 0.4 for RNA; *p* < 0.01 and the absolute value of log fold change > 0.5 for ADT. The DEFs from Mimitou et al. were identified using the criteria of the original paper. The significance of DEFs overlap was determined by Fisher’s exact test^35^ using the fisher.test function in the stats (v4.2.3) R package. GO enrichment analysis was conducted using the clusterProfiler R package (v4.6.2) based on the top 75 DE genes, with a threshold of *p* < 0.05.

#### Cross-species multimodal MOp integration

We integrated cross-species MOp multimodal data using Palette and five competing methods under a unified preprocessing workflow. We did not modify the model architecture of any method. For methods that cannot jointly ingest multiple epigenomic modalities (MIDAS and scVAEIT), we provided an epigenomic representation in their closest supported configuration by using a gene activity matrix (generated using the GeneActivity function in the Signac R package (v1.9.0) with default parameters) as a proxy. In contrast, Palette, scMoMaT, Multigrate, and StabMap can accommodate arbitrary modality combinations and were provided the modality matrices in their native supported formats. No architecture modifications or method-specific hyperparameter tuning were introduced.

#### Cross-species WAT scRNA-seq integration

For tasks relying solely on orthologous genes, all methods received identical input matrices. For tasks incorporating non-orthologous genes, input configurations were adapted based on method capabilities. MIDAS, scVAEIT, and StabMap natively support features not shared across all batches. For these methods, non-orthologous genes were supplied as non-globally shared features within the scRNA-seq modality. Palette, scMoMaT, and Multigrate typically expect globally shared feature sets within a modality. To incorporate non-orthologous genes without altering the source code, we supplied these genes as a separate, additional input modality. This strategy allows the utilization of species-specific information while adhering to the algorithmic constraints of the methods.

#### Low-resolution spatial multimodal integration

We treated low-resolution spatial mosaic datasets mathematically identically to single-cell mosaic data, with the primary distinction being resolution (spot vs. cell). No model architectures were modified. All methods received identically preprocessed data and identical feature sets. They were executed following the standard procedures described in their original publications, utilizing their native supported data formats.

#### Human tonsil datasets analysis

In addition to the metric-based benchmarking, we performed differential expression analysis on scRNA-seq data using both original and transferred cell type labels, using the FindAllMarkers function in the Seurat R package. Similarly, we inferred transcriptome expression on CODEX data, performing differential expression analysis using both original and transferred cell type labels, respectively. For the above four cases, the differential expression genes were identified using the unified criteria: *p* < 0.05 and the absolute value of log fold change > 0.25. The significance of marker overlap was determined by a four-way Fisher’s exact test^88^ using the supertest function in the SuperExactTest (v1.1.0) R package. Spatial cell-cell communication was performed based on the spatial location information of CODEX data and the inferred transcriptome expression using the COMMOT (v0.0.3) Python package and the ligand-receptor information from CellPhoneDB (v4.0) database^90^.

#### Runtime and memory scalability analysis

We evaluated the scalability of Palette by increasing the number of cells under different modality compositions while using default parameter settings. Two bimodal scenarios were created from human BMMC CITE-seq (RNA and protein) and 10x Multiome (RNA and ATAC) datasets, and a trimodal scenario (RNA, protein, and ATAC) was generated by combining the two technologies. The transcriptome, proteome, and chromatin-accessibility modalities were represented by 3000 highly variable genes, 134 surface proteins, and 113,709 peaks, respectively. Datasets of different sizes were constructed by subsampling or oversampling cells (15 k–500 k for bimodal, 10 k–500 k for trimodal). In all scenarios, each batch contained 5000 cells, so the batch number scaled with the dataset size. In the trimodal task, the CITE-seq and 10x Multiome datasets were constrained to have equal sample sizes in each test. In bimodal tasks, 20% of batches were assigned to the full multimodal subset and 40% to each unimodal subset, except in the 15 k cell dataset, where only three batches were available and thus evenly divided. Runtime and peak memory usage were recorded using the peakRAM (v1.0.2) R package. All experiments were conducted on a Linux workstation equipped with an AMD EPYC 7513 CPU (1.50 GHz) and 256 GB of RAM. The results of this analysis are available in Supplementary Fig. 50.

### Evaluation metrics

#### Biological conservation metrics

The biological conservation metrics are composed of normalized mutual information (NMI), adjusted Rand index (ARI), cell type average silhouette width (cASW), and cell type local inverse Simpson’s Index (cLISI).

*NMI*. NMI measures the similarity between cell type labels and clustering labels, which was defined as follows:

18 $${{{\rm{NMI}}}}=\frac{2\times I(Y;C)}{H(Y)+H(C)},$$

where $Y$ and $C$ are true cell type labels and clustering labels respectively, $H(.)$ represents entropy, and $I(Y;C)$ represents mutual information between $Y$ and $C$. NMI has a range of 0 to 1, and higher values indicate better match.

*ARI*. ARI measures the overlap between cell type labels and clustering labels similarly, which is the corrected-for-chance version of the Rand index (RI). ARI takes into account both agreement and disagreement between two types of labels. ARI has a range of 0–1, and higher values indicate a better match.

cASW. The average silhouette width (ASW) is the average value of SW across all cells, which typically ranges from −1 to 1. It measures how similar the cell is to its own label compared to other labels. The cASW is modified on ASW, which is defined as follows:

19 $${{{\rm{cASW}}}}=\frac{1}{2\times |C|}{\sum }_{i\in C}(s(i)+1),$$

where $s(i)$ is the SW for cell $i$, $C$ represents the set of all cells, and $|C|$ represents the number of cells in set $C$. The cASW has a range of 0–1, and higher values indicate a better match.

*cLISI*. The cLISI measures how well cell separation occurs across cell types based on the local inverse Simpson’s Index (LISI) score. Original LISI ranges from 1 to $N$, where $N$ is the number of unique labels. The cLISI scales the value to $[0,\,1]$, and higher values indicate better separation.

*Biological conservation score*. The biological conservation score is defined as the mean value of NMI, ARI, cASW, and cLISI following the recent study^30^. We formulize this metric as follows:

20 $${S}_{{{{\rm{bio}}}}}=({{{\rm{NMI}}}}+{{{\rm{ARI}}}}+{{{\rm{cASW}}}}+{{{\rm{cLISI}}}})/4,$$

where ${S}_{{{{\rm{bio}}}}}$ indicates the biological conservation score.

#### Batch correction metrics

The batch correction metrics are composed of batch average silhouette width (bASW), integration local inverse Simpson’s index (iLISI), k-nearest-neighbor batch effect test (kBET), and Graph connectivity.

*bASW.* The bASW is similar to the cASW but measures how well cell mixing occurs across batches, which is defined as follows:

21 $${{{\rm{bASW}}}}=\frac{1}{|M|}{\sum }_{j\in M}{\sum }_{i\in {C}_{j}}\frac{1-|s(i)|}{|{C}_{j}|},$$

where $|s(i)|$ is the absolute SW for cell $i$, ${C}_{j}$ represents the set of cells in batch $j$, $|{C}_{j}|$ represents the total number of cells in batch $j$, and $|M|$ represents the number of unique batch labels. The bASW has a range of 0–1, and higher values indicate better mixing.

*iLISI*. The iLISI is similar to the cLISI but evaluates batch mixing in local neighborhoods. The iLISI scales the value to $[0,\,1]$, and higher values indicate better mixing.

*KBET*. The kBET measures the batch mixing performance on the integrated kNN graph. It uses Pearson’s ${\chi }^{2}$ test to compare the similarity between local and global batch label distribution through multiple random samplings. The result of kBET is 1 minus the average test rejection rate, ranging from 0 to 1, and higher values indicate better mixing.

*Graph connectivity*. Graph connectivity (GC) measures the connectivity of cells from the same cell type in the global kNN graph. For a cell type $c$, we defined the corresponding subgraph as ${G}_{c}({N}_{c};{E}_{c})$. Then we identified the size of the largest connected component of the subgraph, denoted as $|{{\mathrm{LCC}}}({G}_{c})|$. The cell type-specific GC score is calculated by $|{{\mathrm{LCC}}}({G}_{c})|/|{N}_{c}$|, where $|{N}_{c}|$ represents the number of cells in cell type $c$. The total GC score is defined as follows:

22 $${{\mathrm{GC}}}=\frac{1}{|C|}{\sum }_{c\in C}|{{\mathrm{LCC}}}({G}_{c})|/|{N}_{c}|,$$

where $|C|$ denotes the number of unique cell types.

*Batch correction score*. The batch correction score is defined as the mean value of bASW, iLISI, kBET, and GC score following the recent study^30^. We formulize this metric as follows:

23 $${S}_{{{{\rm{batch}}}}}=({{{\rm{bASW}}}}+{{{\rm{iLISI}}}}+{{{\rm{kBET}}}}+{{{\rm{GC}}}})/4,$$

where ${S}_{{{{\rm{batch}}}}}$ indicates the batch correction score.

#### Overall integration score

We followed Luecken et al.^30^ to compute an overall integration score, which is defined as follows:

24 $${S}_{{{{\rm{overall}}}}}=0.6\times {S}_{{{{\rm{bio}}}}}+0.4\times {S}_{{{{\rm{batch}}}}},$$

where ${S}_{{{{\rm{overall}}}}}$ denotes the overall integration score.

#### Modality mixing metrics

To quantify the mixing of cells originating from different modality compositions, we employed three metrics: the cell-type-stratified Seurat alignment score (CSAS), modality kBET, and CiLISI^31^. These metrics characterize the extent of modality mixing in mosaic integration settings but are not intended to serve as standalone measures of overall integration quality. It should therefore be evaluated together with additional biological metrics. In this study, integration quality was assessed by combining the modality mixing metric with the biological conservation and batch correction metrics.

*CSAS*. The Seurat alignment score (SAS), originally introduced by Butler et al.^104^, measures how well datasets are aligned in a shared embedding space. To account for heterogeneity across cell types, we computed SAS separately within each cell type and reported the median value as CSAS. For a given cell type $c$, datasets representing different modality compositions were first downsampled to match the smallest group. SAS was then computed on the kNN graph as

25 $${{{\mathrm{SAS}}}}_{c}=1-\frac{{\bar{x}}_{c}-\frac{{K}_{c}}{{N}_{c}}}{{K}_{c}-\frac{{K}_{c}}{{N}_{c}}},$$

where ${N}_{c}$ is the number of modality compositions present in cell type $c$, and ${\bar{x}}_{c}$ is the average number of neighbors sharing the same modality composition. Following Butler et al.^104^, ${K}_{c}$ was set to 1% of the downsampled cell count. As with SAS, CSAS ranges from 0 to 1, with higher values indicating stronger modality mixing.

*Modalty kBET*. To evaluate mixing using local neighborhood homogeneity, we adapted kBET by treating modality composition labels as the grouping variable. kBET was computed within each cell type, and the median rejection rate across cell types was used as the modality kBET score.

*CiLISI*. CiLISI, originally proposed by Andreatta et al.^31^, evaluates batch mixing by computing iLISI within individual cell types. To account for variability in cell type abundance and the differing modality compositions, we implemented a modified version. Within each cell type, datasets were downsampled to the smallest modality group, and iLISI was calculated on the downsampled cells. The resulting values were normalized to the range 0–1, and the median across all cell types was reported as CiLISI.

*Modality mixing score*. The modality mixing score is defined as the mean value of CSAS, modality kBET, and CiLISI. We formulize this metric as follows:

26 $${S}_{{{{\rm{mod}}}}}=({{{\rm{CSAS}}}}+{{{{\rm{kBET}}}}}_{{{{\rm{mod}}}}}+{{{\rm{CiLISI}}}})/3,$$

where ${S}_{{{{\rm{mod}}}}}$ indicates the modality mixing score, and ${{{{\rm{kBET}}}}}_{{{{\rm{mod}}}}}$ denotes modality kBET, respectively. We further tested the behavior of these three metrics against four additional metrics using a suite of simulated scenarios designed to probe their sensitivity and robustness (Supplementary Fig. 51 and Supplementary Note 5).

#### Reference query and cross-species mixing metrics

To assess mixing across different sources, we used the same three metrics as for modality mixing, replacing modality labels with dataset or species labels as appropriate. The overall score was defined as the mean of these three metrics.

#### Label prediction metrics

For the reference-based integration and diagonal integration task, label transfer performance was evaluated using accuracy and macro F1.

*Accuracy*. The accuracy metric was used to measure the label transfer performance at the dataset level. It is defined as the proportion of correctly predicted labels in the total number of cells in the dataset. The accuracy has a range of 0–1, and higher values indicate better label transfer performance.

*Macro F1*. The macro F1 score measures label transfer performance at the cell type level. It is defined as the mean of the label transfer F1 scores of each cell type in the dataset. The macro F1 score ranges from 0 to 1, and higher values indicate better label transfer performance.

### Reporting summary

Further information on research design is available in the Nature Portfolio Reporting Summary linked to this article.

## Supplementary information

Supplementary Information

Peer Review File

Description of Additional Supplementary Files

Supplementary Data 1

Supplementary Data 2

Supplementary Data 3

Supplementary Data 4

Reporting Summary

## Source data

Source Data

## Data Availability

All datasets used in this manuscript are publicly available. The human PBMC TEA-seq dataset^9^ is available in the Gene Expression Omnibus (GEO) database under accession code GSE158013. The human BMMC CITE-seq and 10x Multiome datasets we used to construct benchmark data, reference, and query are available in the GEO database under accession code GSE194122. The human retina 10x Multiome dataset^28^ is available in the GEO database under accession code GSE196235. The human BMMC Ab-seq dataset^29^ is available at https://figshare.com/projects/Single-cell_proteogenomic_reference_maps_of_the_human_hematopoietic_system/94469. The HCA BMMC scRNA-seq reference dataset is available at https://explore.data.humancellatlas.org/projects/cc95ff89-2e68-4a08-a234-480eca21ce79. The cross-condition human PBMC dataset^10^ is available in the GEO database under accession code GSE156473. Five batches of the cross-species MOp dataset (Human_10xV3, Human_SNARE, Mouse_10xV3, Marmoset_10xV3, and Marmoset_SNARE)^47^ are available at https://assets.nemoarchive.org/dat-ek5dbmu. Two batches of the cross-species MOp dataset (Human_10xMultiome_ATAC and Mouse_10xMultiome)^48^ are available in the GEO database under accession code GSE229169. One batch of the cross-species MOp dataset (Mouse_10xATAC) is available at https://www.10xgenomics.com/cn/datasets/fresh-cortex-from-adult-mouse-brain-p-50−1-standard-1-1-0. The human cortex Slide-tags dataset^14^ is available at https://singlecell.broadinstitute.org/single_cell/study/SCP2169/slide-tags-snrna-seq-on-human-tonsil. The cross-species WAT scRNA-seq dataset^52^ can be accessed via the CELLxGENE portal at https://cellxgene.cziscience.com/collections/fe0e718d-2ee9-42cc-894b-0b490f437dfd. The human tonsil 10x Visium dataset^56^ is available at 10.5281/zenodo.12654113. The human kidney scRNA-seq and scATAC-seq datasets^72^ can be accessed at https://datasets.cellxgene.cziscience.com/0da9127b-6faf-458c-a124-d04315f4db6e.h5ad and https://datasets.cellxgene.cziscience.com/2d8349bd-3045-4b9a-bbad-301ea3190fef.h5ad, respectively. The human PBMC scRNA-seq dataset^73^ is available in the GEO database under accession code GSE164378. The human PBMC CyTOF dataset^74^ is available at http://flowrepository.org/id/FR-FCM-Z249. The human tonsil scRNA-seq dataset^75^ is available in the GEO database under accession code GSE165860. The human tonsil CODEX dataset^15^ is available at https://onlinelibrary.wiley.com/doi/10.1002/eji.202048891. The mouse MOp MERFISH dataset^94^ is available at https://doi.brainimagelibrary.org/doi/10.35077/g.21. The mouse MOp scATAC-seq dataset^93^ is available at https://catlas.org/mousebrain/#!/. The human MTG dataset^96^ can be accessed via the Allen Brain Map portal at https://portal.brain-map.org/atlases-and-data/rnaseq. The human immune scRNA-seq, the human pancreas scRNA-seq and the mouse brain scATAC-seq dataset^30^ can be accessed at 10.6084/m9.figshare.12420968. The human PBMC ADT dataset^98^ is available at 10.5281/zenodo.5504061. The human heart 10x Multiome dataset^99^ is available at https://www.heartcellatlas.org. More details of the datasets are provided in Supplementary Data 1. Processed datasets used for the analyses have been deposited in Zenodo at 10.5281/zenodo.18045027. Source data are provided with this paper.

## Code availability

The code used to develop the model in this study is publicly available and has been deposited in GitHub at https://github.com/qiongyusheng/Palette, under the AGPL-3.0 license. The specific version of the code associated with this publication is archived in Zenodo and is accessible via 10.5281/zenodo.18773001^105^. The source code used to reproduce the results in this manuscript is available at GitHub: https://github.com/qiongyusheng/Palette_reproducibility and at Zenodo: 10.5281/zenodo.18743288^106^.
