# Supplementary material for: Robust integration of single-cell datasets with imbalanced modality composition
Source: Nat Commun. 2026 May 14;17:6423. doi: 10.1038/s41467-026-72933-4 (PMC13376370; doi:10.1038/s41467-026-72933-4)
Supplement: Supplementary file 1 — Supplementary Information [file 41467_2026_72933_MOESM1_ESM.pdf]

Supplementary Information

Robust integration of single-cell datasets with imbalanced modality composition

Qiongyu Sheng[1], Yang Zhou[1*], Fengping Zhu[2,3], Li Xu[4*], and Shuilin Jin[1*]

[1] School of Mathematics, Harbin Institute of Technology, Harbin, China

[2] Department of Neurosurgery, Huashan Hospital, Shanghai Medical College, Fudan University, Shanghai, China

[3] Department of Neurosurgery, The Fourth Division Hospital of Xinjiang Production and Construction Corps, Yining, China

[4] College of Computer Science and Technology, Harbin Engineering University, Harbin, China

* Correspondence: Yang Zhou (yangz@hit.edu.cn); Li Xu (xuli@hrbeu.edu.cn); Shuilin Jin (jinsl@hit.edu.cn)

Contents

Supplementary Notes ... 3

Supplementary Note 1. ... 3

Supplementary Note 2. ... 4

Supplementary Note 3. ... 4

Supplementary Note 4. ... 5

Supplementary Note 5. ... 5

Supplementary Methods ... 7

Supplementary Figures ... 13

Supplementary References ... 74

Supplementary Notes

Supplementary Note 1. Evaluation of Palette supervised integration performance under variable cell type annotation conditions

To assess how the quality and completeness of cell type labels influence Palette's supervised integration, we simulated label sets of varying reliability by randomly shuffling or masking 5%, 10%, and 20% of the labels. Palette was then re-run in supervised mode using these perturbed labels, and the resulting integrations were quantitatively evaluated (Supplementary Fig. 15). In nearly all cases, supervised integration with perturbed labels continued to outperform the unsupervised alternative, indicating that supervised integration remains effective even when labels are partially disrupted. Moreover, across all integration tasks, most evaluation metrics showed limited sensitivity to increasing noise or missingness, suggesting that Palette is stable with respect to label quality and completeness.

Considering annotation strategies used for multimodal datasets may also influence integration performance, we next evaluated the effect of annotation modality on integration performance. The human BMMC datasets generated by CITE-seq and 10x Multiome, whose original labels were derived from multimodal consensus, were re-annotated using transcriptome-based unimodal information. To avoid introducing human-driven biases, we employed two fully data-driven Seurat-based procedures: label transfer from the HCA BMMC scRNA-seq reference[1] and batch integration followed by k-means clustering to generate cluster labels. Using these two unimodal annotation schemes, we re-ran Palette supervised integration for the BMMC scenario 1 task (Supplementary Fig. 16a and b). Compared with multimodal consensus labels, unimodal annotations led to moderately lower scores for batch correction, biological conservation, and overall integration, whereas modality mixing performance was comparable or slightly improved (Supplementary Fig. 16c). Nevertheless, supervised integration under unimodal annotations consistently outperformed the unsupervised setting.

Collectively, these analyses indicate that Palette's supervised integration exhibits stable behavior across a range of realistic annotation perturbations, including incomplete labels, noisy labels, and differences in annotation strategy.

Supplementary Note 2. Evaluation of Palette horizontal integration on scRNA-seq data

To evaluate Palette's horizontal integration performance, we compared it with representative state-of-the-art single-cell integration algorithms. These included unsupervised methods (fastMNN[2], Seurat v3[3], Harmony[4], and STACAS[5]), supervised methods (scANVI[6], scPoli[7], and SIGNAL[8]), and the semi-supervised method ssSTACAS[9]. Since scRNA-seq data remains the most widely used modality for horizontal integration, we benchmarked all algorithms, and with Palette in both supervised and unsupervised modes, across three real scRNA-seq datasets (MTG, immune, and pancreas)[10,11].

Overall integration performance showed that scPoli, Palette supervised mode, and STACAS were the top-performing methods across the three tasks (Supplementary Fig. 39a). Among the unsupervised approaches, Palette unsupervised mode ranked second. We also observed that both modes of Palette achieved high biological conservation scores (Supplementary Fig. 39b). Following Andreatta et al.[9], we further examined CiLISI and cell type ASW, two metrics considered more suitable for evaluating batch correction and biological conservation. Using these metrics, Palette still maintained high biological conservation (cell type ASW) scores across datasets (Supplementary Fig. 39c).

We next assessed the sensitivity of supervised and semi-supervised methods to the quality and completeness of cell type labels. To this end, we perturbed labels by randomly shuffling or masking 5%, 10%, and 20% of the cell type annotations, and re-ran the supervised and semi-supervised algorithms using these modified labels. ssSTACAS showed the highest robustness in both perturbation scenarios, maintaining stable cell type ASW scores under increasing label noise and missing (Supplementary Fig. 39d). Palette preserved stable performance under increasing levels of label missing, with slightly higher sensitivity to label shuffling compared to ssSTACAS.

Supplementary Note 3. Path selection evaluation under varying modality sparsity

Here, we used the human PBMC TEA-seq dataset to generate three distinct gene sets and two distinct peak sets through different feature selection strategies (Supplementary Methods), thereby constructing a six-modality mosaic integration scenario (Supplementary Fig. 43a). We first quantified the sparsity of each modality. Then, for each missing modality, three propagation paths were evaluated: (1) Palette's default path selection mode, i.e., the shortest path, which may traverse either sparse or dense modalities; (2)

relatively shorter paths that traverse a larger number of sparser modalities; and (3) relatively longer paths that traverse a larger number of denser modalities.

Quantitative evaluations of the integrated embeddings under these three paths showed that Palette's default path selection achieved the best performance across all metrics (Supplementary Fig. 43b). The shorter path traversing sparser modalities ranked second, performing comparably to the longer path traversing denser modalities in batch correction metrics and outperforming it in all remaining metrics. These results indicate that choosing the shorter available path is generally more effective than selecting longer paths, even when the shorter path traverses sparser modalities than those included in the longer path.

Supplementary Note 4. Benchmarking modality inference performance

We evaluated missing-modality inference using five benchmark scenarios. BMMC Scenario 1 was excluded because the missing modality lacks real measurements and therefore cannot be quantitatively assessed. For each remaining scenario, one sub-experiment was selected for inference (Supplementary Methods). Palette was compared with three methods capable of performing missing-modality inference: MIDAS, Multigrate, and scVAEIT.

Inference performance was assessed in a modality-specific manner within each task using data structure and clustering-based metrics following a recent benchmark study[12] (Supplementary Methods). Across all scenarios and nearly all metrics, Palette either achieved the highest performance scores or exhibited performance comparable to the top-performing methods. An exception was observed for the standardized mean squared error (sMSE) metric on scATAC-seq data, where Palette did not rank among the top-performing methods (Supplementary Figs. 44 and 45). Overall, these results indicate that Palette enables accurate and reliable missing modality inference across diverse multimodal settings.

Supplementary Note 5. Evaluation of modality mixing metrics

To evaluate the behavior of modality mixing metrics, we assessed seven metrics using a suite of simulated scenarios. These metrics include three measures (details of metric definitions are provided in the Methods), CSAS, modality kBET, and CiLISI, which are computed separately within each cell type, as well as four additional metrics computed at the dataset level, including the original kBET, SAS, iLISI, and LISI F1.

We first simulated datasets with varying degrees of modality mixing across modality compositions (Supplementary Fig. 51a and Supplementary Methods). As the mixing level increased, all metrics except the original kBET exhibited a consistent upward trend (Supplementary Fig. 51b). The reduced sensitivity of the original kBET in this setting can be attributed to the presence of modality-specific cell types, which violate its assumption of shared label distributions across datasets. Under perfect mixing, only modality kBET, CSAS, and CiLISI reached values close to 1, whereas the remaining metrics remained comparatively low.

We next examined the effect of imbalanced data sizes across modality compositions by progressively removing cells from Modality 1 under the Well Mixed setting (Supplementary Methods). In this scenario, iLISI and LISI F1 decreased as the degree of imbalance increased, while all other metrics remained largely stable (Supplementary Fig. 51c). This behavior indicates that most metrics are robust to unequal data sizes across modality compositions, whereas iLISI and LISI F1 are more sensitive to data size imbalance.

Finally, we evaluated scenarios with mismatched cell type compositions across modality compositions. We distinguished between balanced settings, in which all modality compositions share identical cell type compositions, and imbalanced settings, in which specific cell types are present in only a subset of modality compositions (Supplementary Fig. 51d and Supplementary Methods). In the imbalanced setting, SAS and iLISI achieved high scores (Supplementary Fig. 51e). In contrast, under balanced settings, the original kBET, SAS, and iLISI produced inflated values, reflecting their insensitivity to cell type mismatches.

Taken together, these results show that the metrics quantifying within cell types, modality kBET, CSAS, and CiLISI, are robust to imbalanced cell type distributions and unequal data sizes across modality compositions, while remaining sensitive to true modality mixing. We therefore selected these three metrics as the primary measures of modality mixing throughout this study.

Supplementary Methods

Comparison between Bi-sPCA and existing dimensionality reduction methods. Bi-sPCA is designed to address dimensionality reduction problems in which learned representations are expected to be associated with a variable of interest while being as independent as possible of an unwanted source of variation. The multi-view learning methods, such as canonical correlation analysis (CCA) and its variants, aim to identify projections that maximize correlation between two data views, typically treating the views symmetrically. In contrast, Bi-sPCA operates on a single data matrix and uses kernelized supervision to regulate dependence on multiple external variables. Its objective is not to align two views, but to learn the low-dimensional representation with respect to predefined factors.

Bi-sPCA is also distinct from multi-view or joint PCA approaches, which seek to learn a shared low-dimensional subspace across multiple views by minimizing reconstruction error or enforcing low-rank structure. Such methods primarily focus on capturing shared variance across views and do not explicitly model competing sources of dependence. By contrast, Bi-sPCA is formulated to disentangle variation associated with different external variables, which act on the same data matrix, making it particularly suitable for integration scenarios where supervision and confounder control are both required.

Comparison between the MBG-guided propagation strategy and StabMap's strategy. The MBG-guided propagation strategy in Palette and the approach used in StabMap both exploit predefined mosaic data structures to guide integration, but they differ fundamentally in graph construction and algorithmic role.

Specifically, StabMap relies on a Mosaic Data Topology (MDT), a weighted graph whose nodes correspond to batches and whose edge weights are determined by the number of shared features between batches. StabMap integration is reference-based: one or multiple reference batches are specified, and all other batches are mapped to the reference embedding along paths determined by MDT edge weights, resulting in a fixed, batch-level mapping strategy. In contrast, Palette constructs an unweighted Mosaic Bipartite Graph (MBG) whose nodes represent both batches and modalities, with edges encoding only the presence of a modality in a batch. The MBG does not impose a reference batch or a predefined mapping order. Instead, it defines admissible paths for information transfer and is used to infer low-dimensional representations of missing modalities. Propagation in Palette is path-conditioned and cell-specific, with optimal paths selected dynamically based on nearest-neighbor distances and multiple candidate paths fused through distance-based weighting.

Thus, although both MDT and MBG encode mosaic data structure, MDT in StabMap guides reference-based batch mapping through weighted batch–batch relationships, whereas MBG in Palette serves as a structural prior for adaptive, path-conditioned inference of missing modalities. The MDT- and MBG-based strategies address distinct integration objectives and are not interchangeable.

An explanation of parameter selection in Palette. Palette integration involves several parameters that are specified at different stages of the workflow, including the quantile threshold for selecting representative cells, the thresholds T_1 and T_2 for identifying similar clusters across batches, and the Bi-sPCA regularization parameter λ used at different integration stages. Here, we explain the rationale behind the default settings of these parameters and provide practical guidance for selecting appropriate values when applying Palette to new data modalities.

Quantile threshold for representative cell selection. The quantile threshold determines the proportion of representative cells selected within each cell type or cluster, based on silhouette width (SW) rankings as described earlier. Higher quantile thresholds correspond to selecting fewer cells. This design serves two purposes: it reduces computational complexity and improves robustness to noisy or potentially mislabeled cells. For modalities where measurements are sparse and cell–cell separability is weak, using a higher quantile threshold (i.e., retaining fewer, more representative cells) can reduce the influence of ambiguous observations on downstream similarity estimation. For large-scale datasets, higher thresholds can also reduce runtime.

Global modality-specific thresholds T_1 and T_2. The thresholds T_1 and T_2 are jointly used to filter similar clusters across batches in the unsupervised setting. Specifically, T_1 acts as a global, modality-specific lower bound on cluster similarity, whereas T_2 is the cosine value of an adaptive angular constraint that accounts for within-cluster variability through the combination of the median cluster angle θ_c and a modality-specific offset θ_m. A cluster-pair similarity is retained only if it exceeds $T = \max\{T_1, T_2\}$. These two thresholds play complementary roles: T_1 controls a global similarity baseline across batches, while T_2 adjusts the similarity requirement according to the intrinsic structure of each cluster. In practice, when within-dataset separability and cross-batch consistency are strong, users may consider slightly more stringent settings (higher T_1, smaller θ_m); conversely, when signals are weak or sparse, more permissive settings can help avoid discarding potentially related clusters.

Bi-sPCA regularization parameter λ. The Bi-sPCA framework includes a regularization parameter that controls the balance between suppressing unwanted technical variation and preserving biologically meaningful signals. In Palette, λ is used in two distinct contexts.

During the joint dimensionality reduction stage, λ^m is specified in a modality-specific manner to account for differences in dimensionality and sparsity across modalities. Modalities with relatively dense feature-by-cell matrices and strong cell–cell separability, such as scRNA-seq and ADT, often tolerate moderately larger λ^m, whereas sparse modalities, such as scATAC-seq, may require more conservative settings to reduce the risk of over-correction. Pseudo-modality matrices may also require slightly stronger regularization in some settings, and we therefore recommend starting from a conservative baseline and refining if needed.

In the subsequent cross-batch alignment stage, a single global λ parameter is used to regulate the suppression of batch-associated variation across modalities. While this parameter serves a different role from λ^m, its selection follows a similar principle: stronger regularization is appropriate when technical heterogeneity across batches is pronounced, while monitoring potential loss of biological structure.

Practical guidance for parameter selection on new modalities. While Palette provides robust default parameters for established modalities (ADT, scRNA-seq, and scATAC-seq), which were used for all benchmarks in this study, users may encounter novel or domain-specific modalities. When applying Palette to a new modality, we recommend a principled "warm-start then refine" strategy. If the new modality shares similar characteristics, including signal-to-noise ratio, dimensionality, and sparsity patterns, with an established modality, its default parameters should serve as the initial baseline. Otherwise, we suggest the following systematic procedure.

Quantile threshold for representative cell selection. In most cases, the default quantile threshold for representative cell selection is sufficient. For extremely large datasets or modalities with very sparse measurements and weak separability, this threshold can be moderately increased to stabilize representative cell selection.

Global modality-specific thresholds T_1 *and* T_2. If no established modality defaults are applicable, we recommend using the pseudo-modality defaults (described in the Methods). This configuration provides a permissive filtering regime intended to reduce the risk of over-filtering potentially informative cross-batch similarity signals for integration. Note that these parameters are effectively bypassed in supervised integration modes.

Bi-sPCA regularization parameter λ. If cell-type labels are available, tuning should be guided by the interplay between batch correction and biological conservation metrics (both described in the Methods). An observation of high batch correction scores coupled with low biological conservation may suggest over-correction, necessitating a decrease in λ^m. Conversely, persistent batch separation despite preserved biological structure suggests under-correction, warranting an increase in λ^m. In the absence of labels, we recommend using batch-wise clustering consistency, such as mean ASW for each batch on the integrated embedding, as a proxy for biological conservation.

Finally, for the global cross-batch alignment parameter λ, while the default value is designed to be conservative and robust, it can be further refined following the same dual-criterion principle as λ^m, balancing alignment strength against biological conservation. When labels are available, users can optimize λ by monitoring the modality mixing score (as defined in the Results) instead of the batch mixing score. Otherwise, the default setting generally provides a robust and conservative choice.

Transcriptomics data horizontal integration methods. We benchmarked Palette with both unsupervised and supervised integration mode against eight data integration methods. scANVI[6] and scPoli[7] were executed using the Python packages 'scvi' (v1.0.4) and 'scarches' (v0.5.9), respectively. Seurat v3[3], Harmony[4], fastMNN[2], and SIGNAL[8] were executed using the R packages 'Seurat' (v4.4.0), 'harmony' (v1.2.0), 'batchelor' (v1.14.1), and 'SIGNAL' (v1.0.0), respectively. Both ssSTACAS[9] and STACAS[5] were run using the 'STACAS' (v2.3.0) R package. All methods were benchmarked using 20-dimensional integrated embeddings. Except where noted, all methods were run with their default integration parameters.

Path selection analysis. We simulated modality-specific expression matrices with different sparsity levels by selecting distinct feature sets from the scRNA-seq and scATAC-seq modalities of the human PBMC TEA-seq dataset. For scRNA-seq, feature selection was performed using the FindVariableFeatures function in the Seurat R package, with the selection.method parameter set to "mean.var.plot," "dispersion," and "vst," respectively, yielding three feature sets containing 1,676, 4,725, and 3,994 genes. For scATAC-seq, we used two feature selection strategies. First, we applied FindVariableFeatures in Seurat with selection.method = "vst" and retained peaks ranked between 50,001 and 100,000. Second, we used the FindTopFeatures function in the Signac R package (default parameters) and similarly retained peaks ranked between 50,001 and 100,000. These feature sets were used to construct modality matrices exhibiting varying degrees of sparsity.

Modality inference analysis. We conducted missing-modality inference experiments using five of the six benchmark tasks that support this evaluation. For each task, one sub-experiment was randomly selected for inference (TEA Scenario 1: 2; TEA Scenario 2: 3; BMMC Scenario 2: 1; Retina: 3; Ab-seq: 4). Palette was compared with three methods designed for missing-modality inference: MIDAS, scVAEIT, and Multigrate. To ensure consistency across inferred outputs, we used the top 50,000 highly variable scATAC-seq peaks as input for all tasks except Ab-seq. For a given modality within a task, inference was performed across multiple batches. Performance was first evaluated separately for each batch using the corresponding metrics, and batch-level values were then averaged to obtain a single modality-level performance score used for comparison across methods and tasks.

Data simulation. We generated simulated datasets with different degrees of modality mixing across modality compositions using the splatter R package (v1.22.1). Specifically, three baseline scenarios were generated, referred to as Separated, Weakly Mixed, and Well Mixed, by jointly setting the parameters batch.facScale and batch.facLoc to 0.1, 0.03, and 0, respectively. Each simulated dataset consisted of three modality compositions (denoted as Modality 1, Modality 2, and Modality 3) and a total of 6,000 cells. Two cell types, termed Group 1 and Group 2, were present in equal proportions. To construct datasets used for evaluating modality mixing under the presence of modality-specific cell types, we applied an additional filtering step to all three baseline scenarios. Specifically, Group 1 cells were removed from Modality 3, and Group 2 cells were removed from Modality 2. The resulting datasets, which exhibit imbalanced cell type distributions across modality compositions, were used for assessing metric sensitivity to varying degrees of modality mixing. To simulate an imbalance in cell numbers across modality compositions, we focused on the filtered Well Mixed dataset described above. From this dataset, we randomly removed 20%, 50%, and 80% of cells from Modality 1, thereby generating three levels of increasing cell number imbalance across modality compositions. These datasets were used for evaluating metric robustness to unequal data sizes. To simulate incorrect cell type alignment across modality compositions, we considered two settings based on the Well Mixed scenario. In the balanced setting, cell type labels in Modality 1 of the original Well Mixed dataset were swapped, such that cells originally labeled as Group 1 were relabeled as Group 2, and vice versa. In this case, all modality compositions retained identical cell type compositions despite the label permutation. In the imbalanced setting, the same label swapping was applied to Modality 1 of the filtered Well Mixed dataset, in which Group 1 had been removed from Modality 3 and Group 2 from

Modality 2. This procedure resulted in mismatched cell type compositions across modality compositions and was used to evaluate metric behavior under cell type misalignment.

Modality inference metrics. We quantitatively evaluated modality inference performance using the evaluation metrics described by Liu et al.[12], which cover both data structure metrics and clustering-based evaluation metrics. The data structure metrics include sMSE relative to the ground-truth data, preservation of feature correlation structure (pFCS), and preservation of differential expression statistics (pDES).

sMSE. We computed the standardized mean squared error between the inferred and ground-truth matrices, with lower values indicating closer agreement.

pFCS. For the inferred and ground-truth data, we independently selected highly variable features (100 for scRNA-seq and ADT, and 1,000 for scATAC-seq). Using each dataset's own feature set, we computed cell–cell correlation matrices and quantified preservation as the mean Pearson correlation between the two matrices.

pDES. For each cell type, DE analysis was performed separately on the inferred and ground-truth data using limma (v3.54.2) to obtain DE feature sets. Cell–cell correlation matrices were then computed from each dataset using its corresponding DE features, and preservation was measured as the mean Pearson correlation between the two matrices.

For clustering-based evaluation, we used ARI, ASW, and NMI, as introduced in Methods section, to quantify the agreement between the Louvain-derived cluster labels and the ground-truth labels.

Supplementary Figures

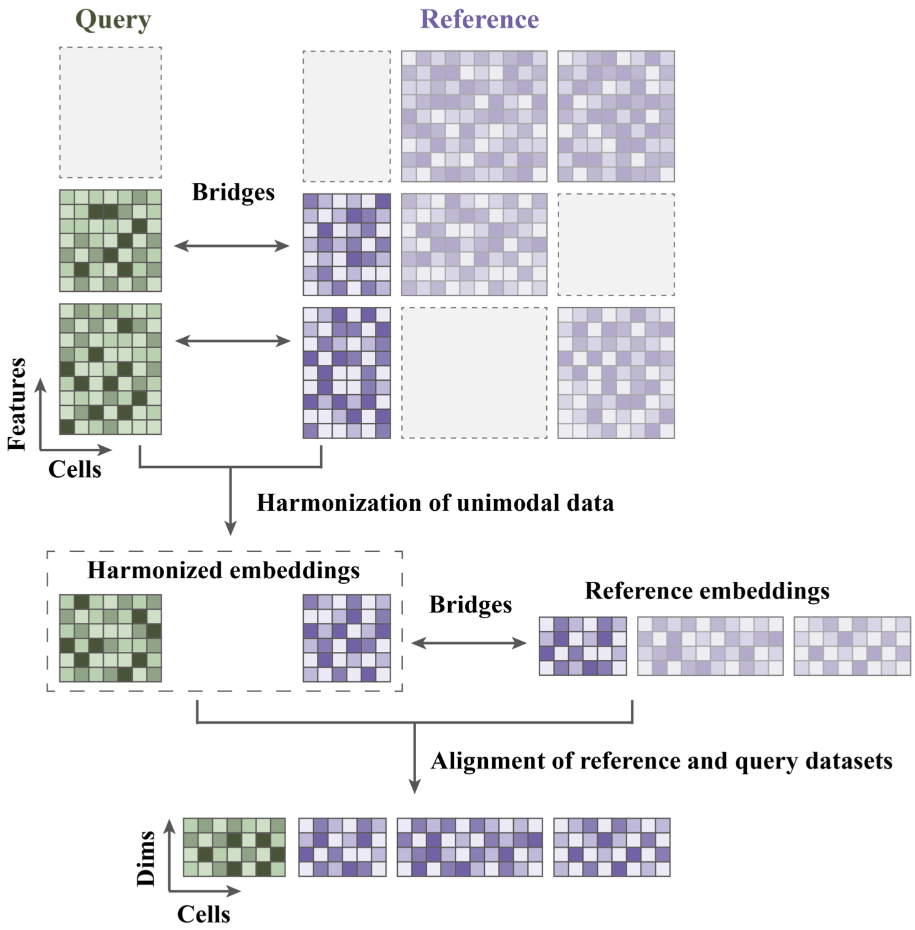

Supplementary Figure 1. Schematic illustration of Palette reference-based integration framework.

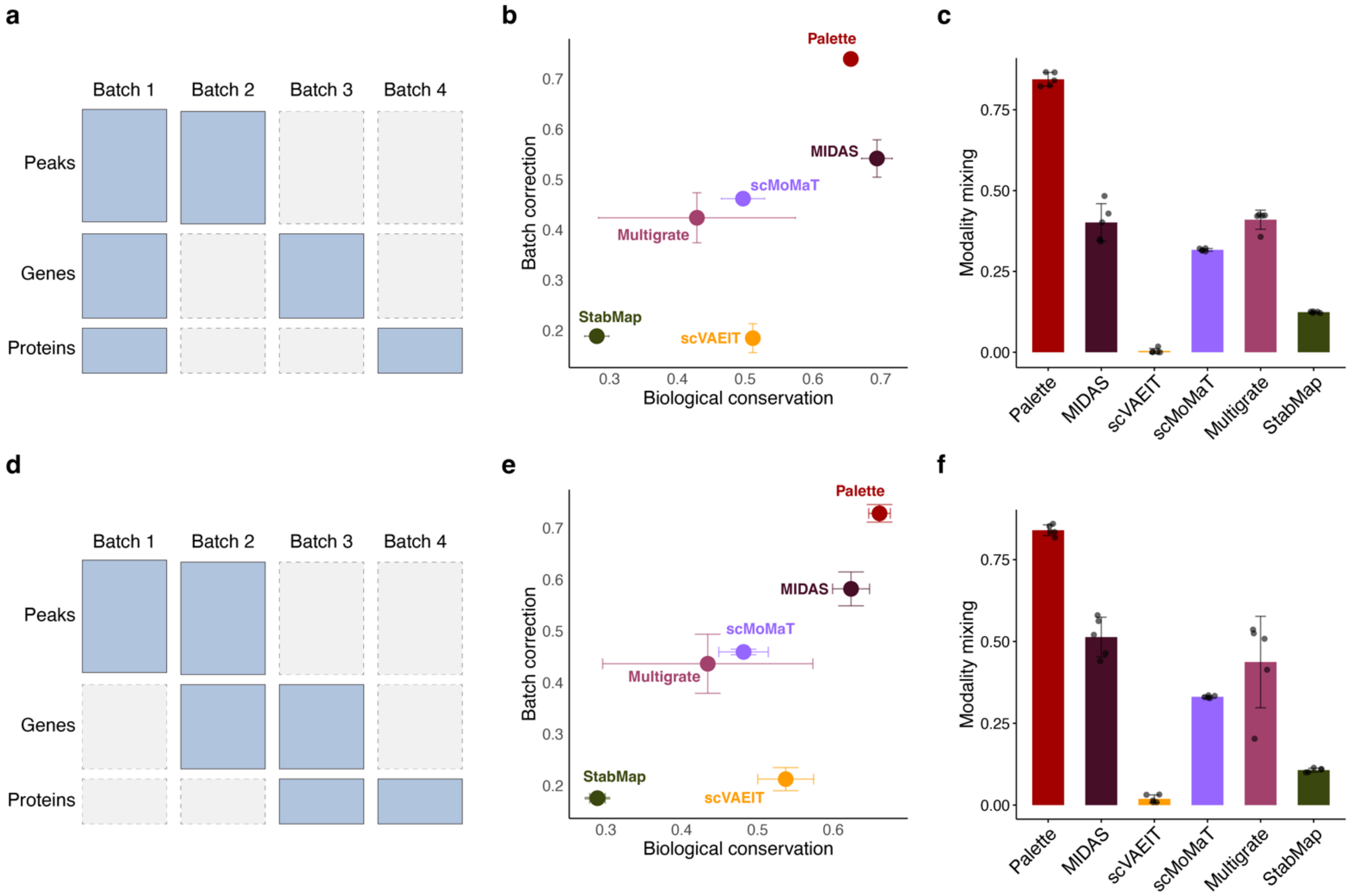

Supplementary Figure 2. Benchmarking of Palette and other integration methods on the TEA scenarios 1 and 2. The top row (**a**–**c**) summarizes results for TEA scenario 1: (**a**) modality composition of each batch; (**b**) comparison of mean biological conservation versus mean batch correction across methods; and (**c**) mean modality mixing scores. The bottom row (**d**–**f**) presents the corresponding results for TEA scenario 2. Error bars represent the standard error across evaluated tasks, from $n = 5$ randomly generated sub-experiments.

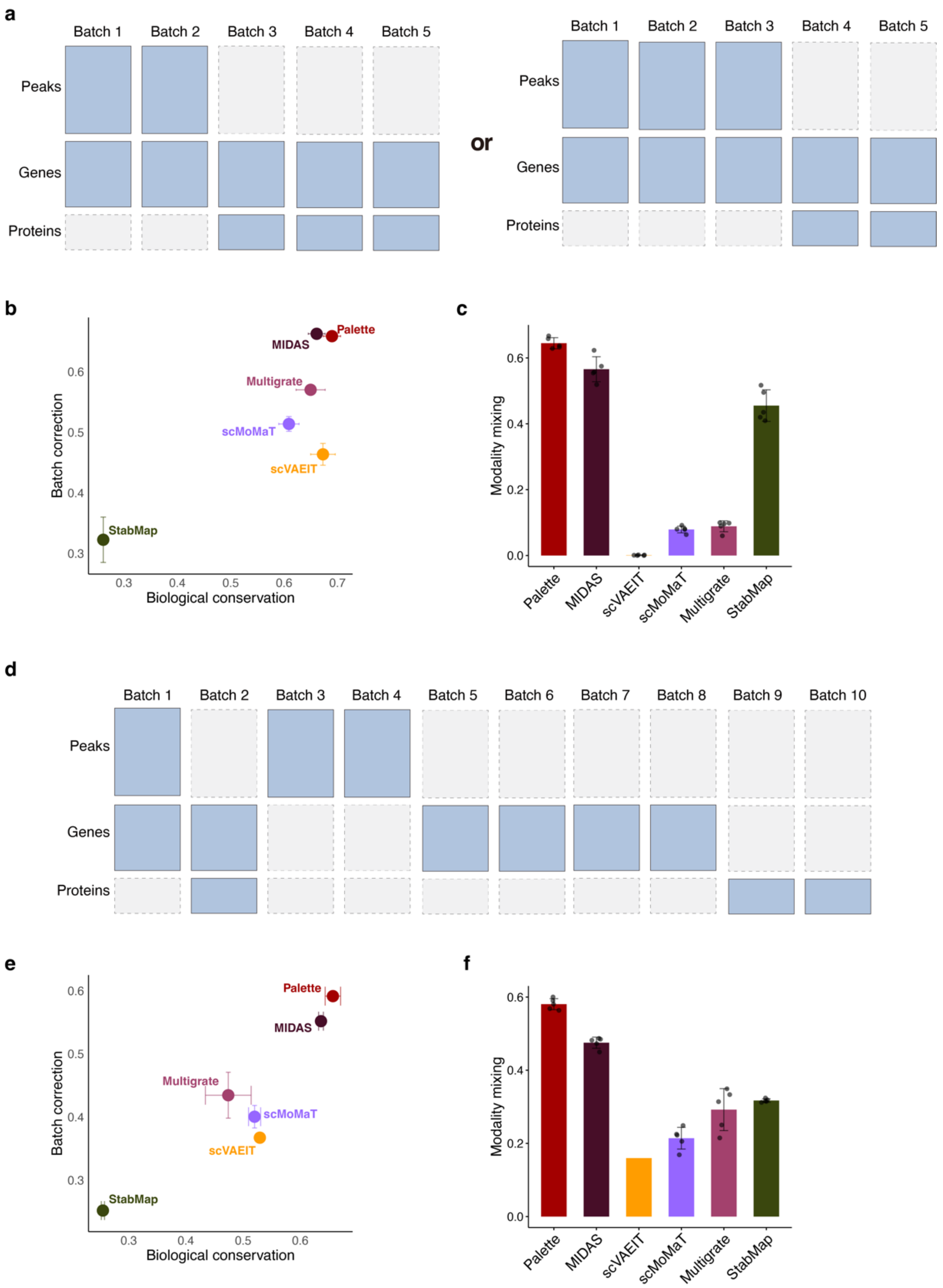

Supplementary Figure 3. Benchmarking of Palette and other integration methods on the BMMC scenarios 1 and 2. The top two rows (**a**–**c**) summarize results for BMMC scenario 1: (**a**) modality composition of each batch; (**b**) mean biological conservation versus batch correction across methods; and (**c**) mean modality mixing scores. The bottom two rows (**d**–**f**) present the corresponding results for BMMC scenario 2. Error bars represent the standard error across evaluated tasks, from $n = 5$ randomly generated

sub-experiments. For BMMC scenario 2, scVAEIT completed only one sub-experiment, so no error bars are shown for this method.

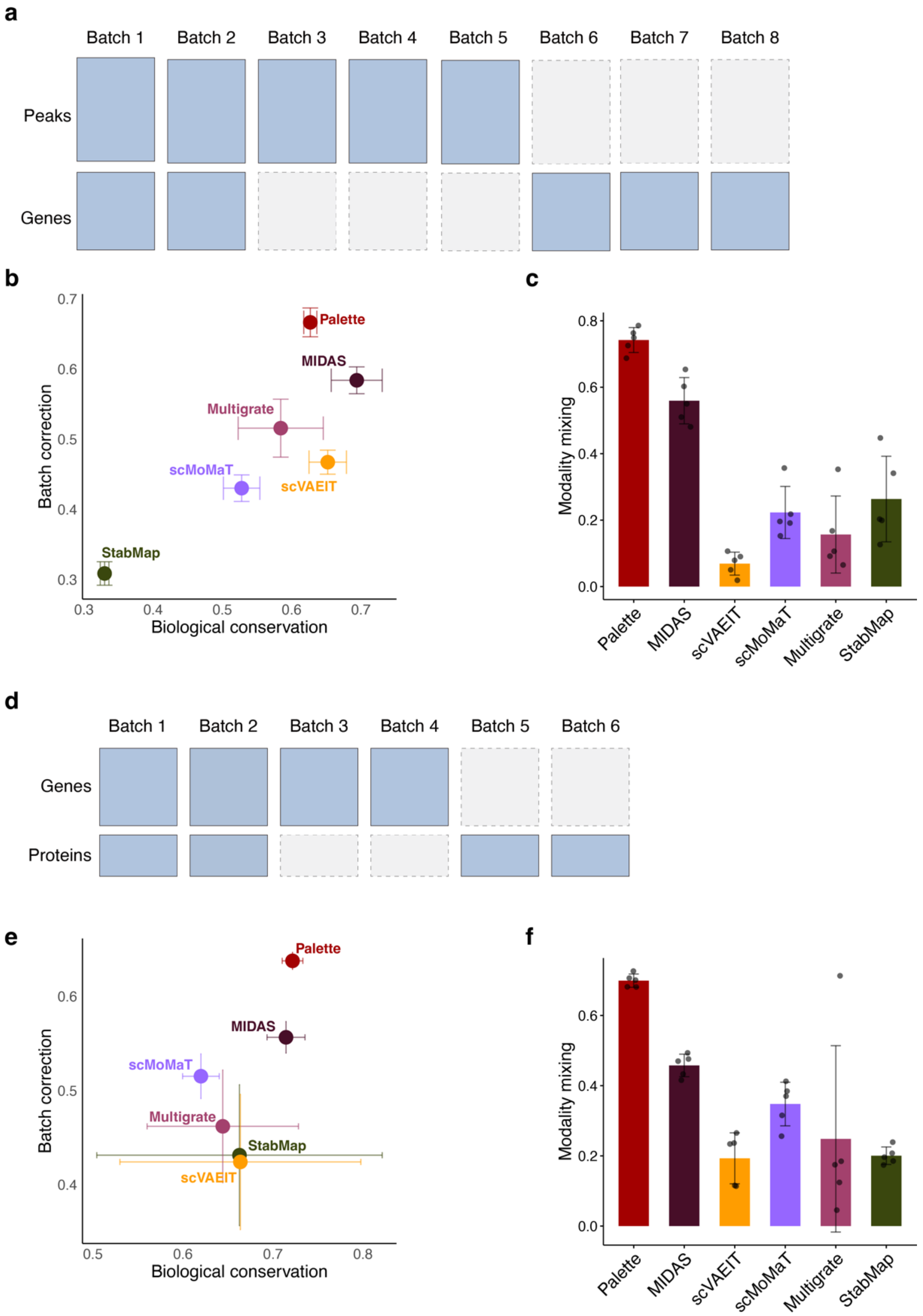

Supplementary Figure 4. Benchmarking of Palette and other integration methods on the Retina and Ab-seq datasets. The top two rows (**a–c**) summarize results for the Retina dataset: (**a**) modality

composition of each batch; (**b**) comparison of mean biological conservation versus mean batch correction across methods; and (**c**) mean modality mixing scores. The bottom two rows (**d–f**) present the corresponding results for the Ab-seq dataset. Error bars indicate the standard error across evaluated tasks, from n = 5 randomly generated sub-experiments.

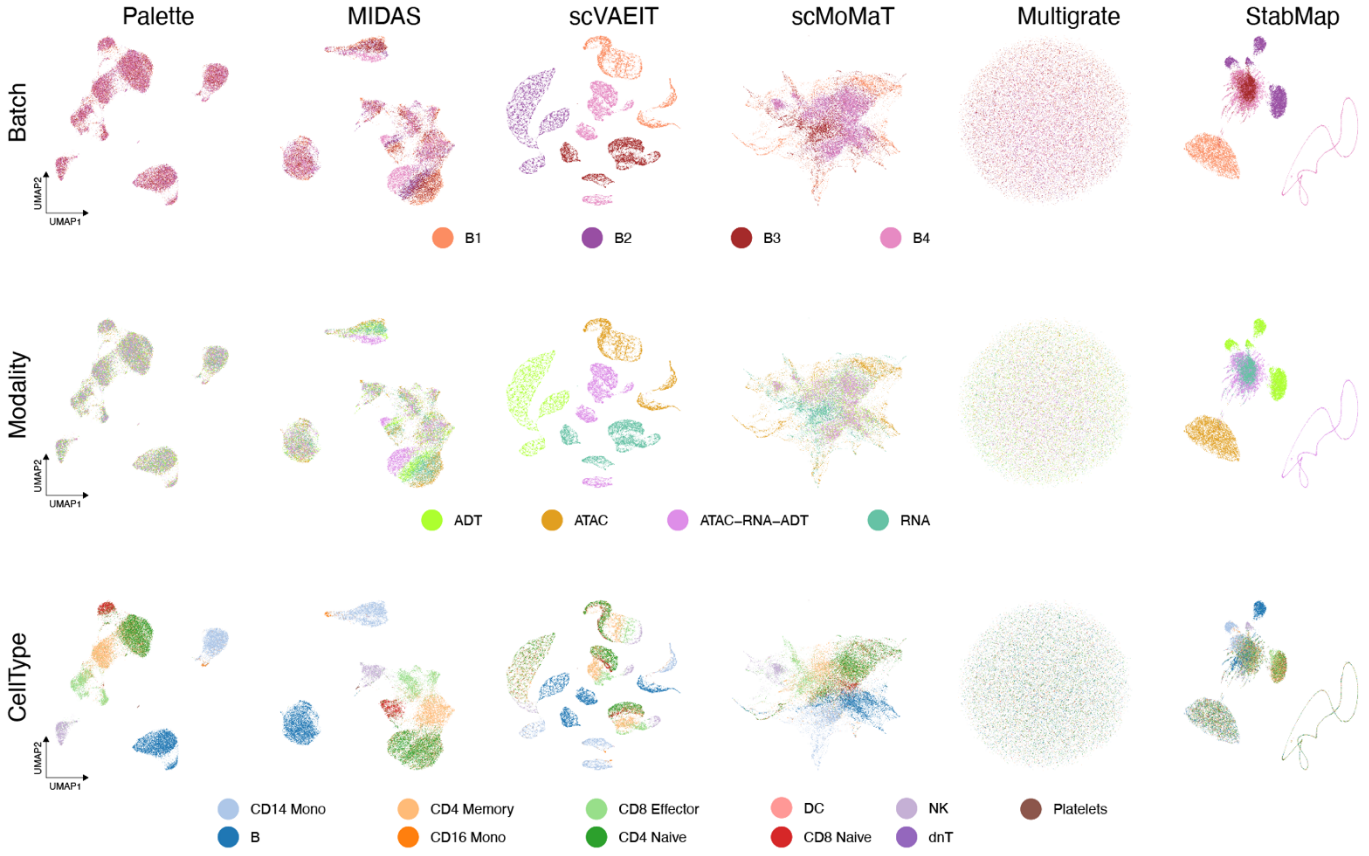

Supplementary Figure 5. UMAP visualizations of integrated cell embeddings generated by Palette and five other integration methods for the first dataset from the TEA scenario 1. Cells are colored by batch (top row), modality composition (middle row), and cell type (bottom row).

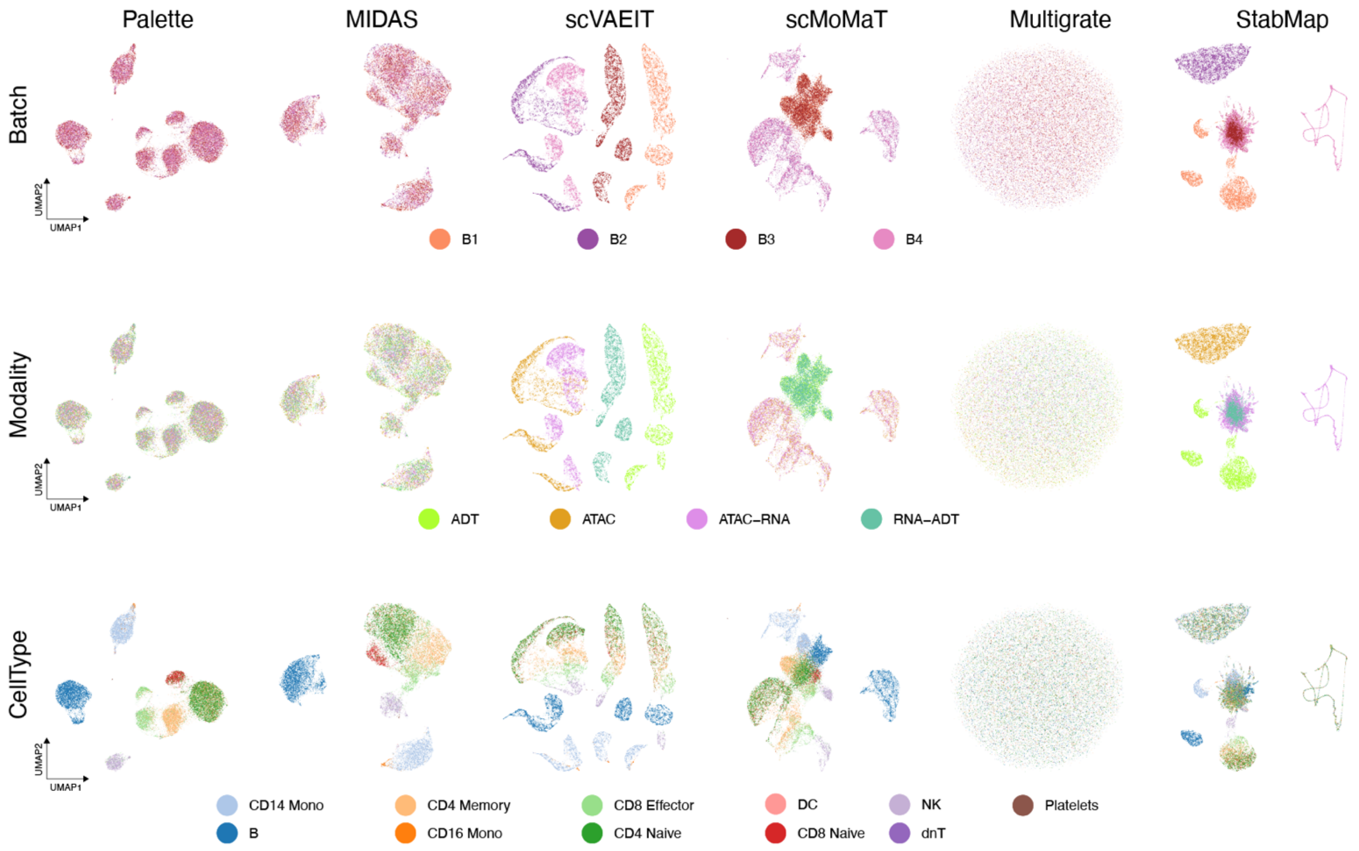

Supplementary Figure 6. UMAP visualizations of integrated cell embeddings generated by Palette and five other integration methods for the first dataset from the TEA scenario 2. Cells are colored by batch (top row), modality composition (middle row), and cell type (bottom row).

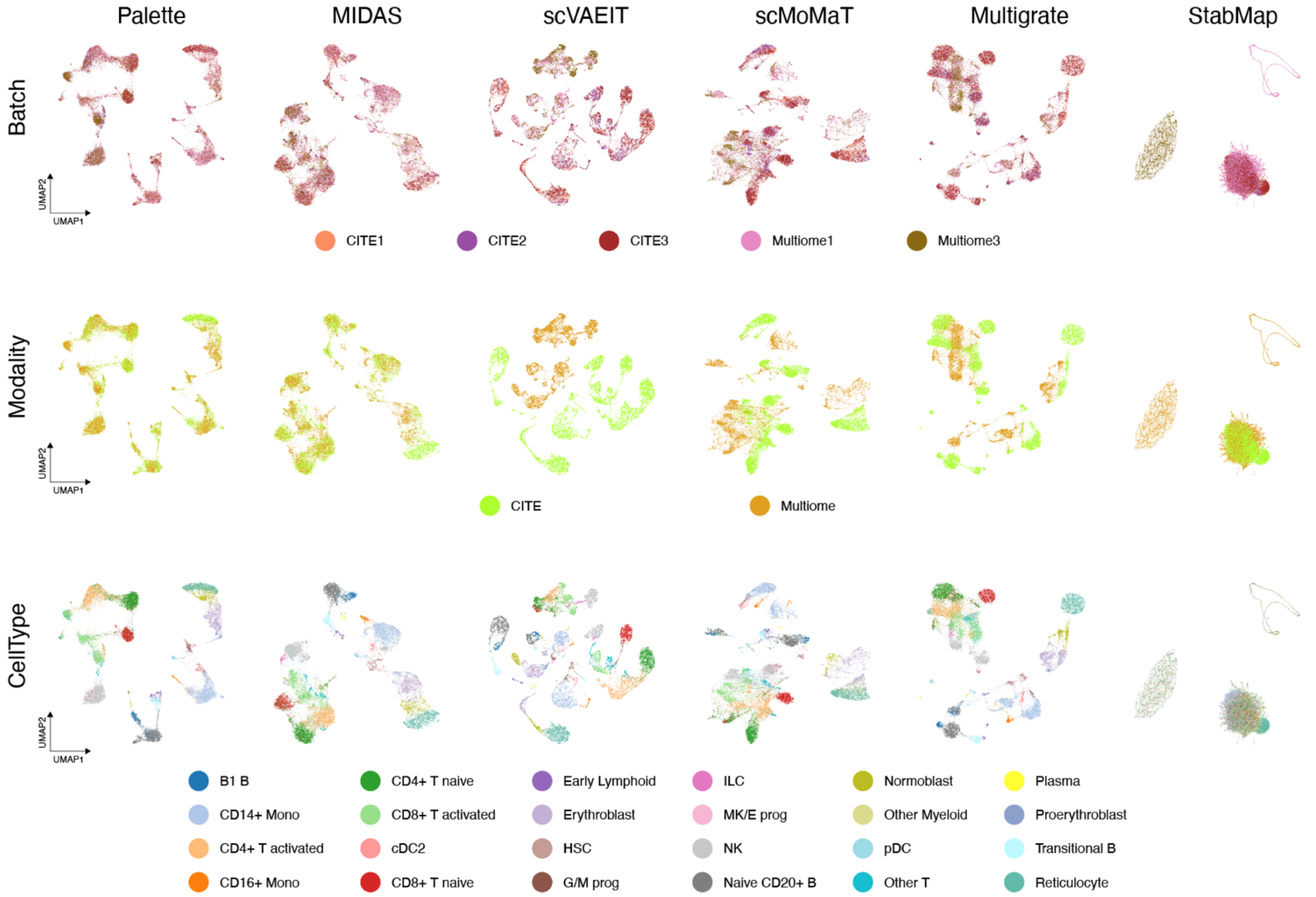

Supplementary Figure 7. UMAP visualizations of integrated cell embeddings generated by Palette and five other integration methods for the first dataset from the BMMC scenario 1. Cells are colored by batch (top row), modality composition (middle row), and cell type (bottom row).

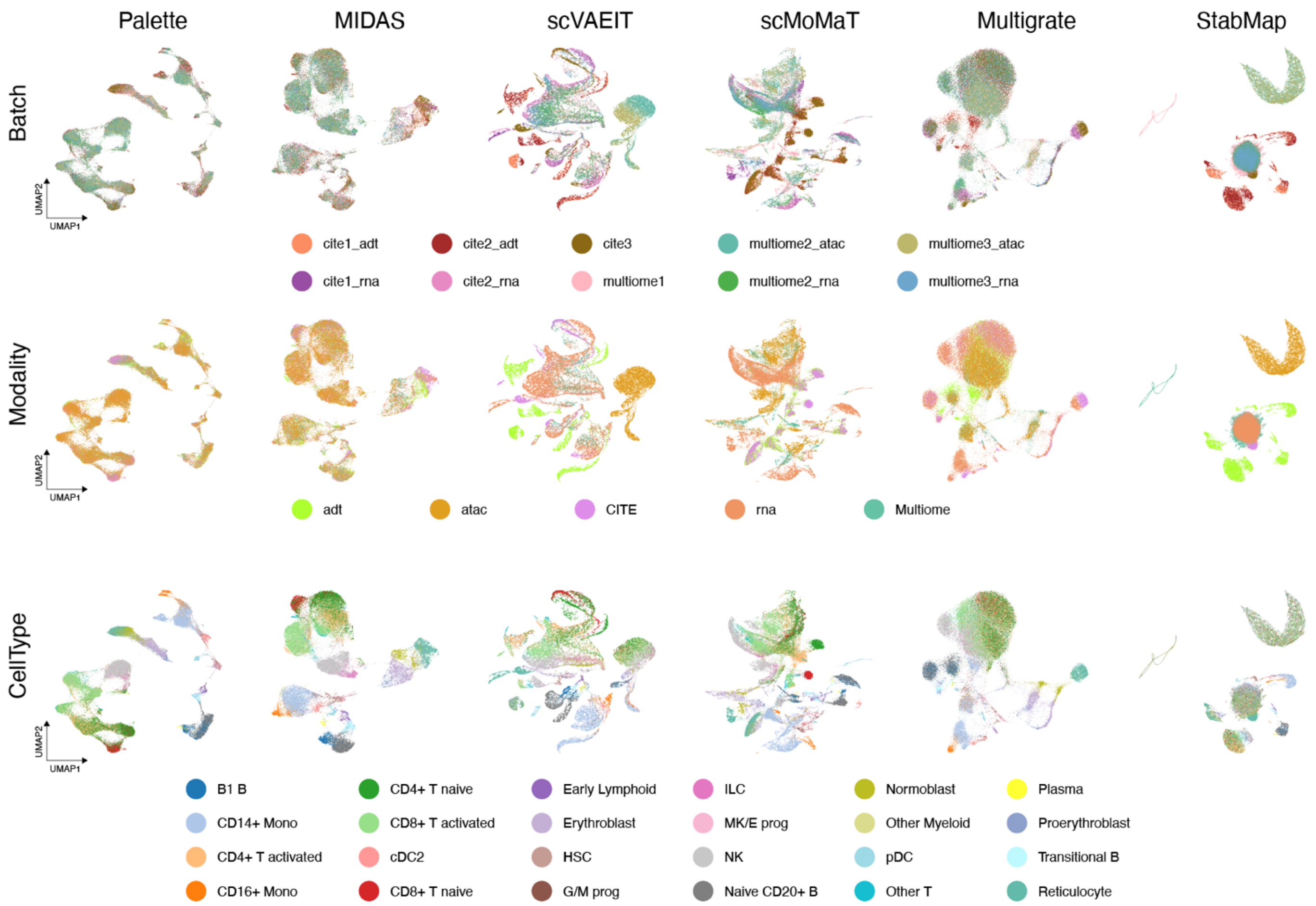

Supplementary Figure 8. UMAP visualizations of integrated cell embeddings generated by Palette and five other integration methods for the first dataset from the BMMC scenario 2. Cells are colored by batch (top row), modality composition (middle row), and cell type (bottom row).

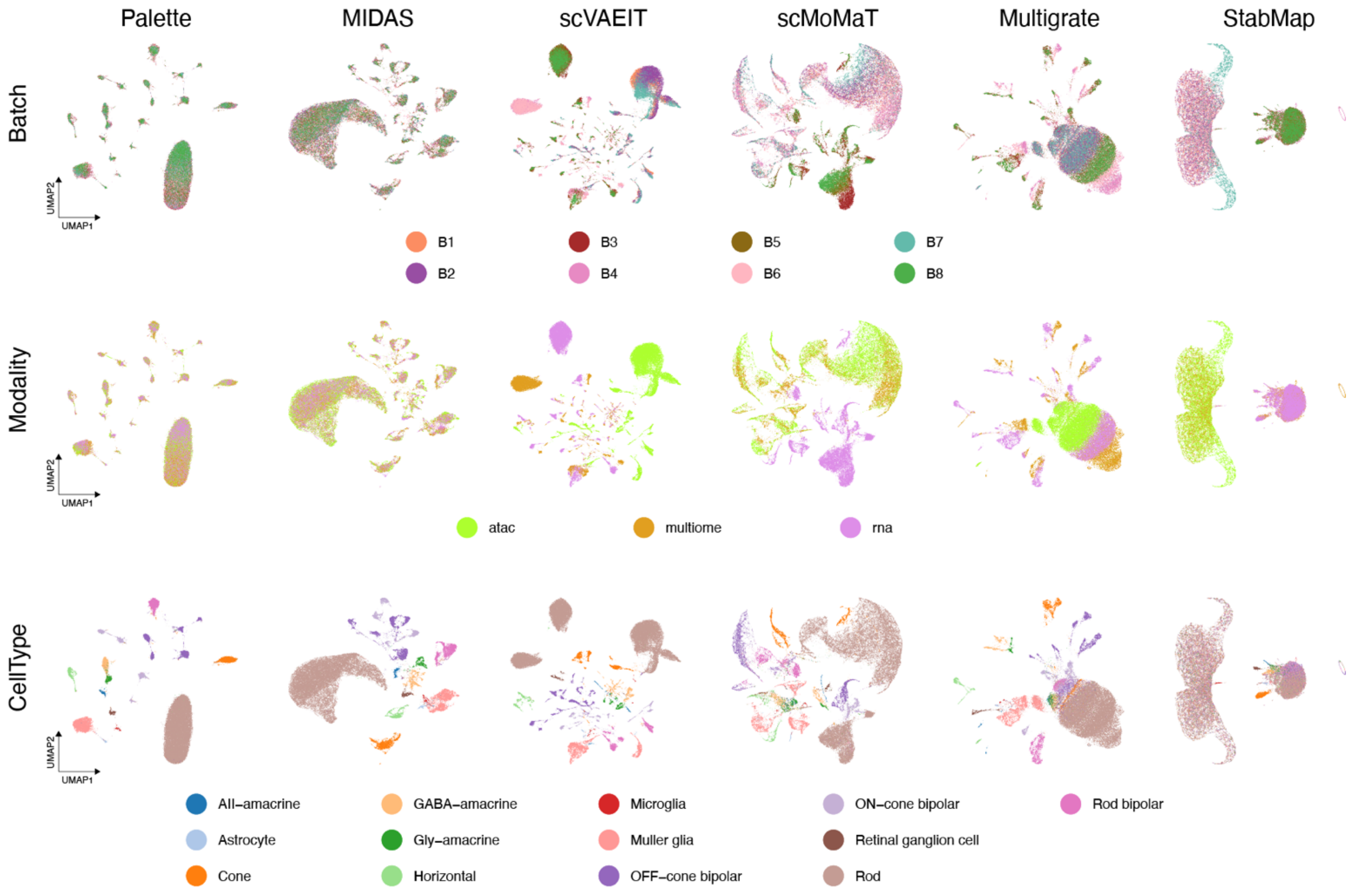

Supplementary Figure 9. UMAP visualizations of integrated cell embeddings generated by Palette and five other integration methods for the first dataset from the retina scenario. Cells are colored by batch (top row), modality composition (middle row), and cell type (bottom row).

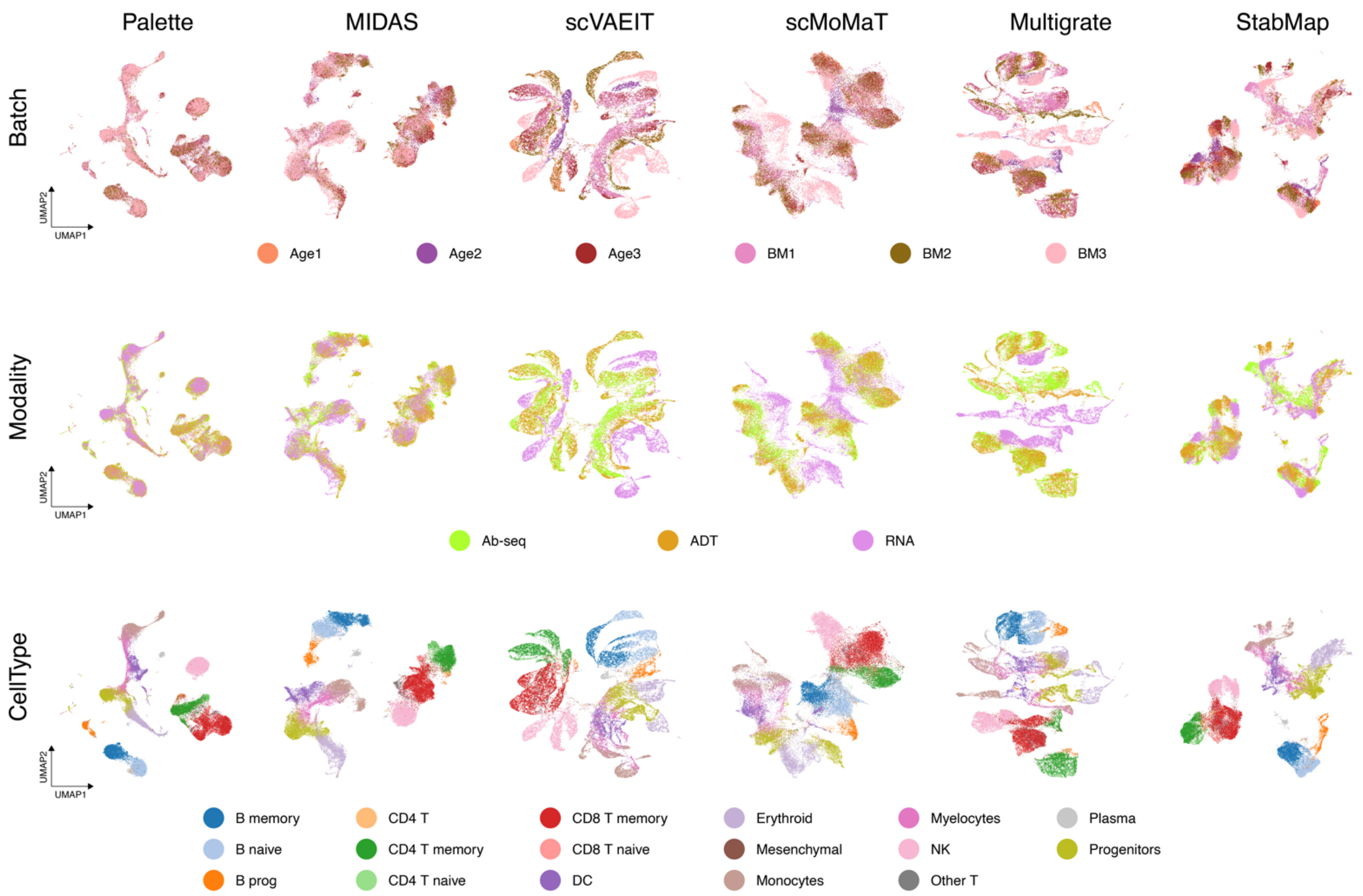

Supplementary Figure 10. UMAP visualizations of integrated cell embeddings generated by Palette and five other integration methods for the first dataset from the Ab-seq scenario. Cells are colored by batch (top row), modality composition (middle row), and cell type (bottom row).

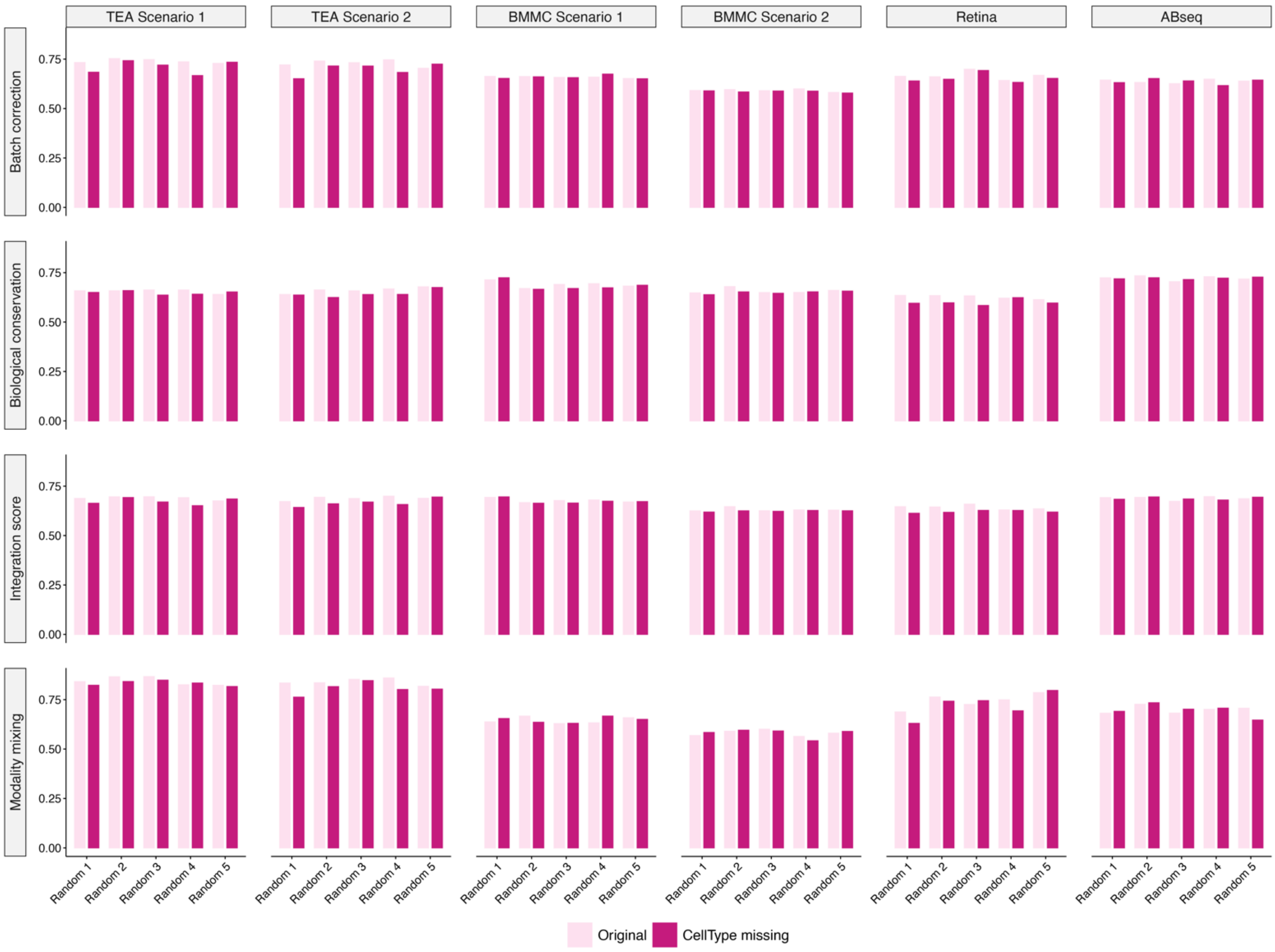

Supplementary Figure 11. Evaluation of Palette's integration performance under imbalanced cell type compositions. Across six integration scenarios and multiple metrics, we compared results on original datasets with those from modified versions in which one cell type was randomly removed from each batch to introduce compositional imbalance. Palette maintained consistently high performance across all scenarios, demonstrating robustness to biological heterogeneity.

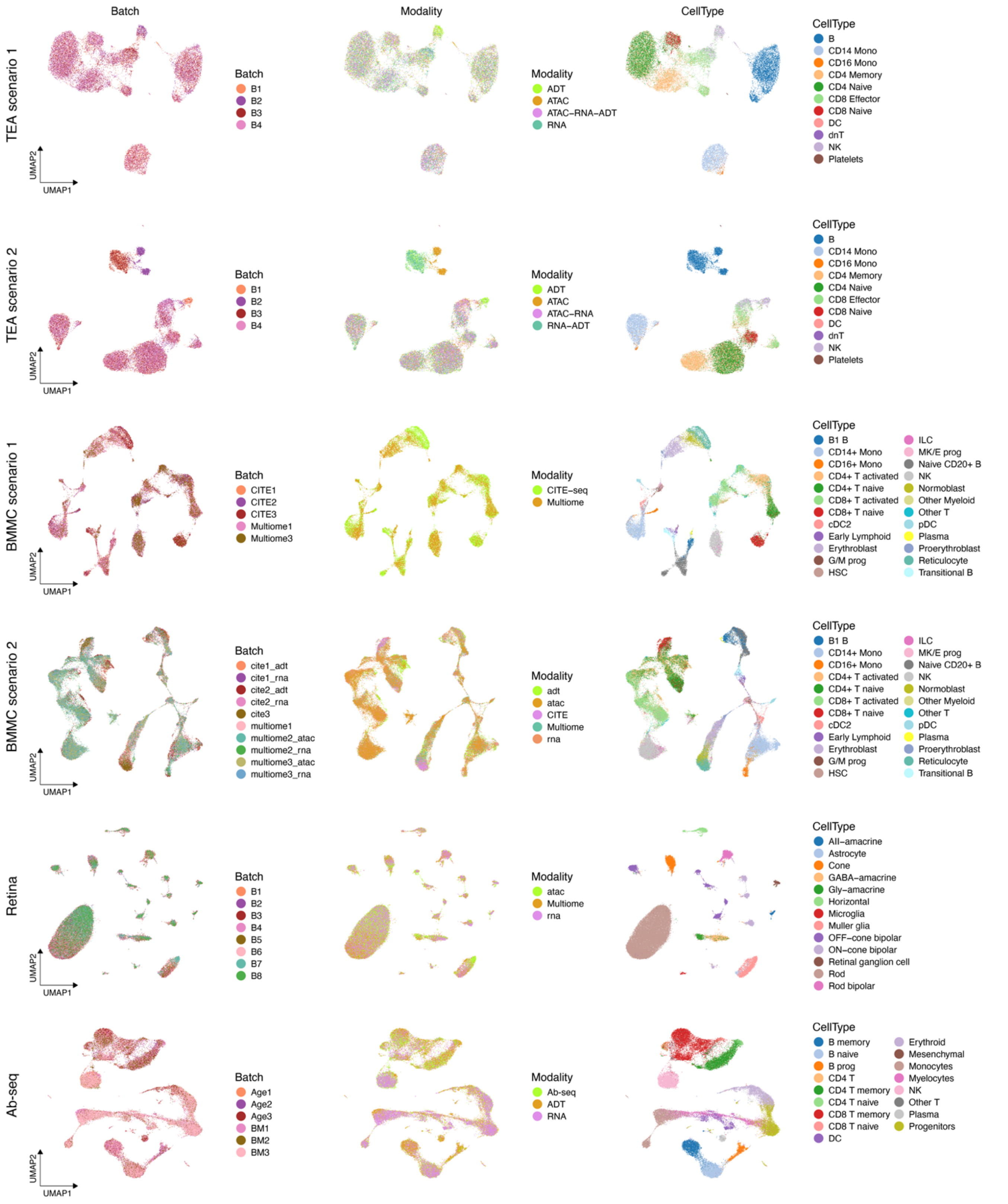

Supplementary Figure 12. UMAP visualizations of integrated cell embeddings generated by Palette on the first dataset of each of six integration scenarios under imbalanced cell type compositions. Cells are colored by batch (left column), modality composition (middle column), and cell type (right column).

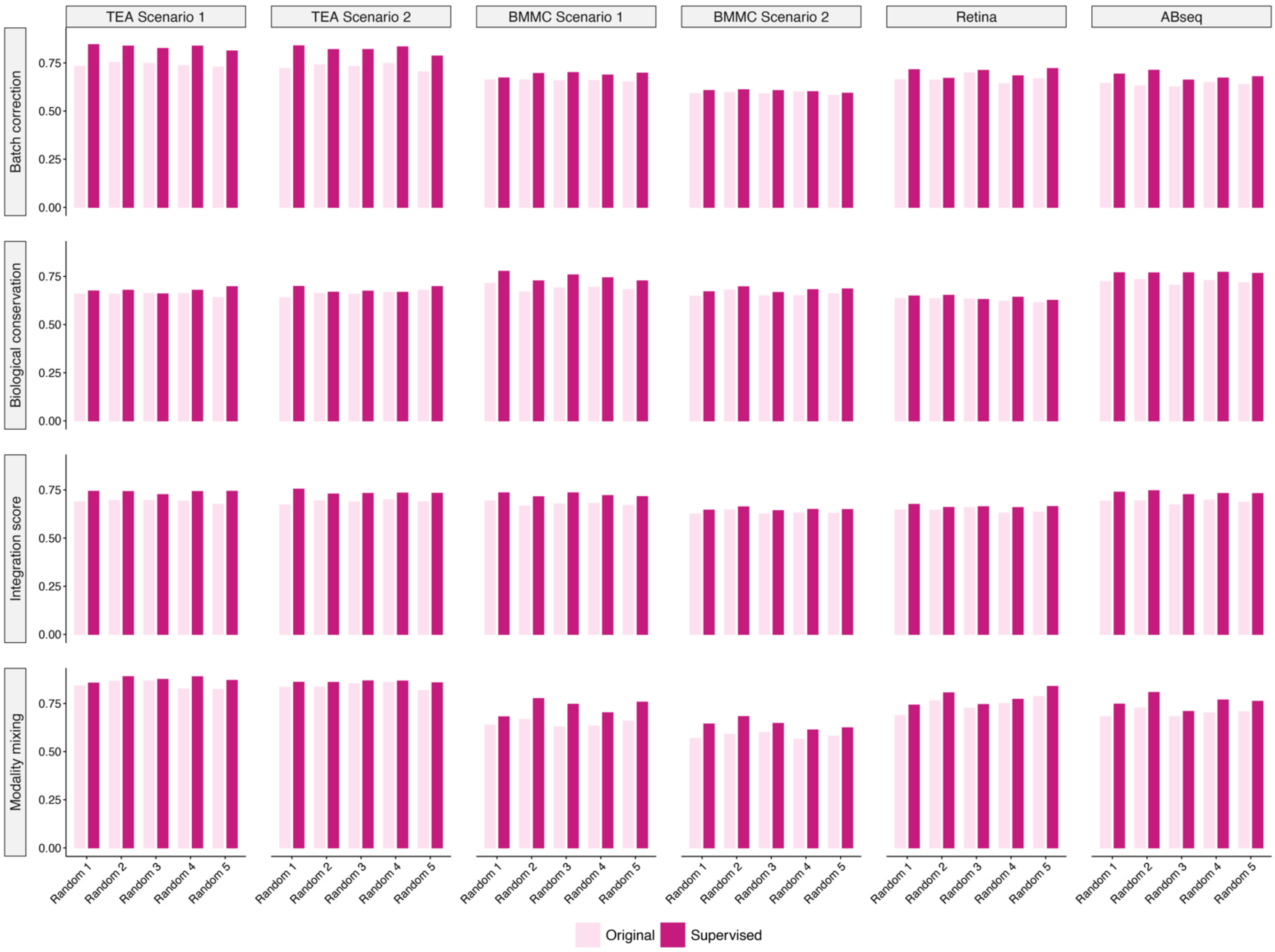

Supplementary Figure 13. Evaluation of Palette's supervised integration performance. Across six integration scenarios and multiple evaluation metrics, we compared Palette's supervised integration mode with its unsupervised counterpart. Supervised integration consistently yielded improved quantitative scores across scenarios.

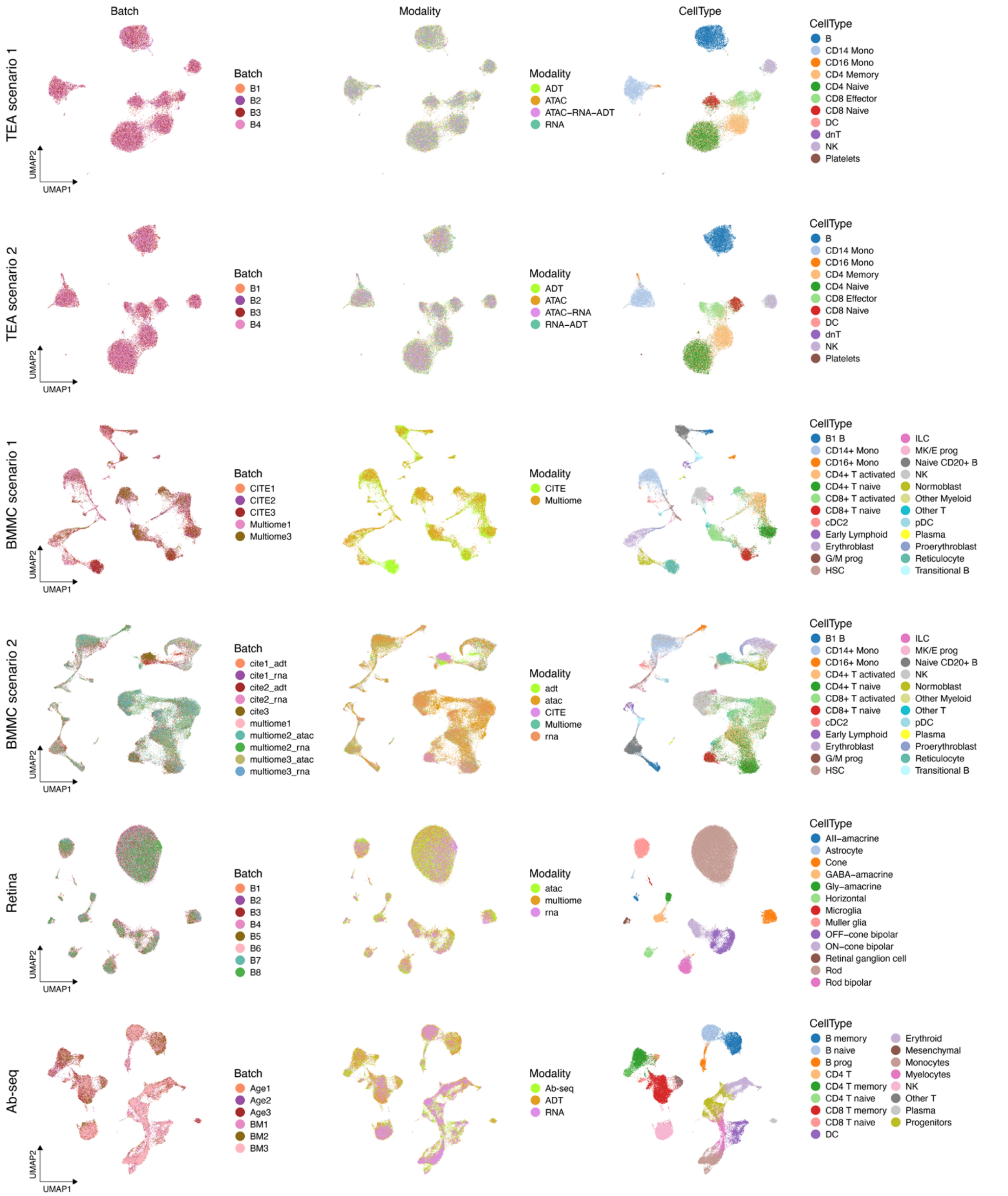

Supplementary Figure 14. UMAP visualizations of integrated cell embeddings generated by Palette in supervised integration mode for the first dataset of each of six integration scenarios. Cells are colored by batch (left column), modality composition (middle column), and cell type (right column).

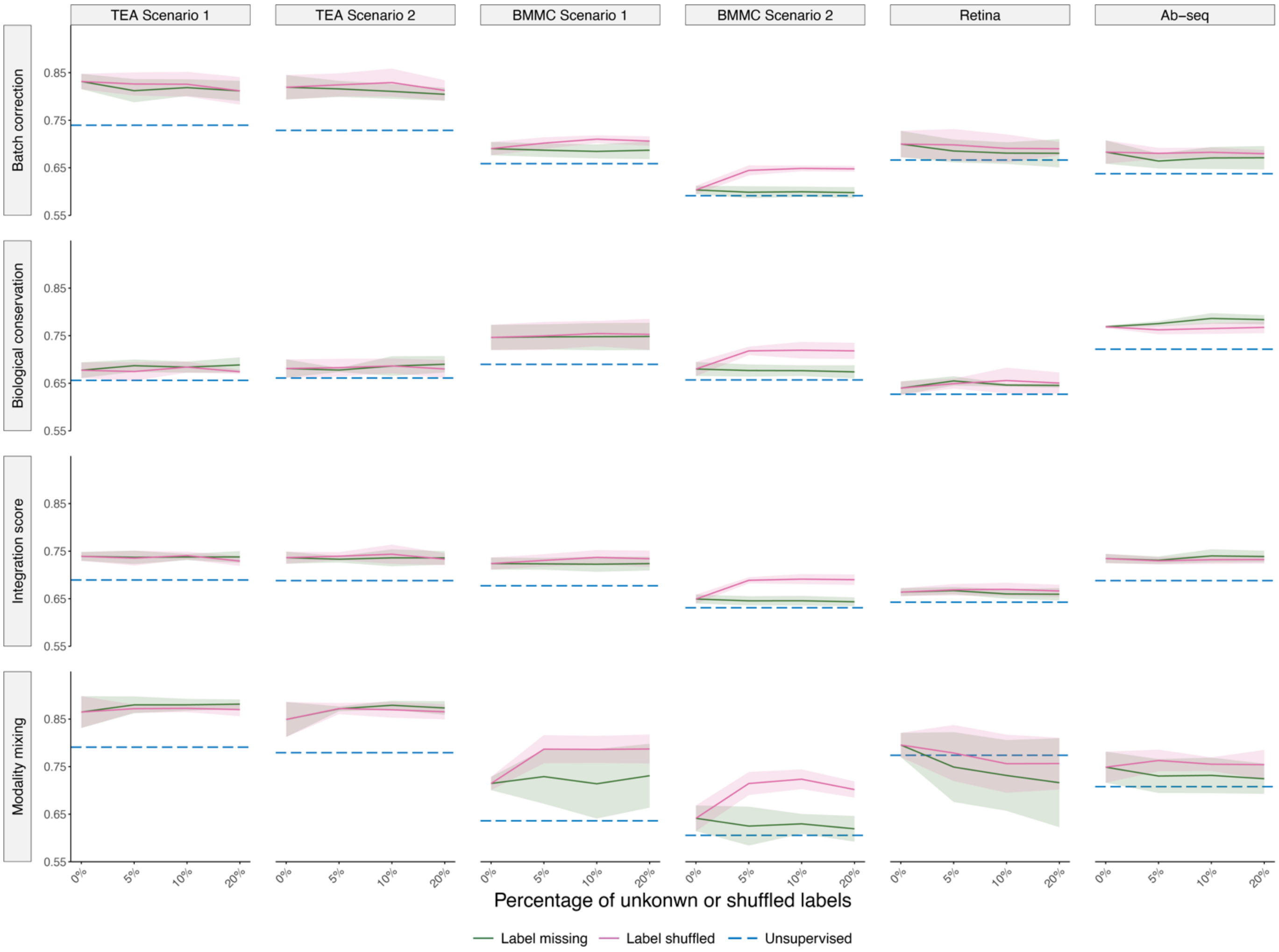

Supplementary Figure 15. Impact of noisy or incompleteness for cell type annotations on the supervised integration performance of Palette. Quantitative evaluation of Palette's supervised integration across multiple datasets under increasing proportions of unknown or shuffled cell type labels. Lines denote mean performance, and shaded regions represent the 95% confidence interval estimated from n = 5 randomly generated sub-experiments.

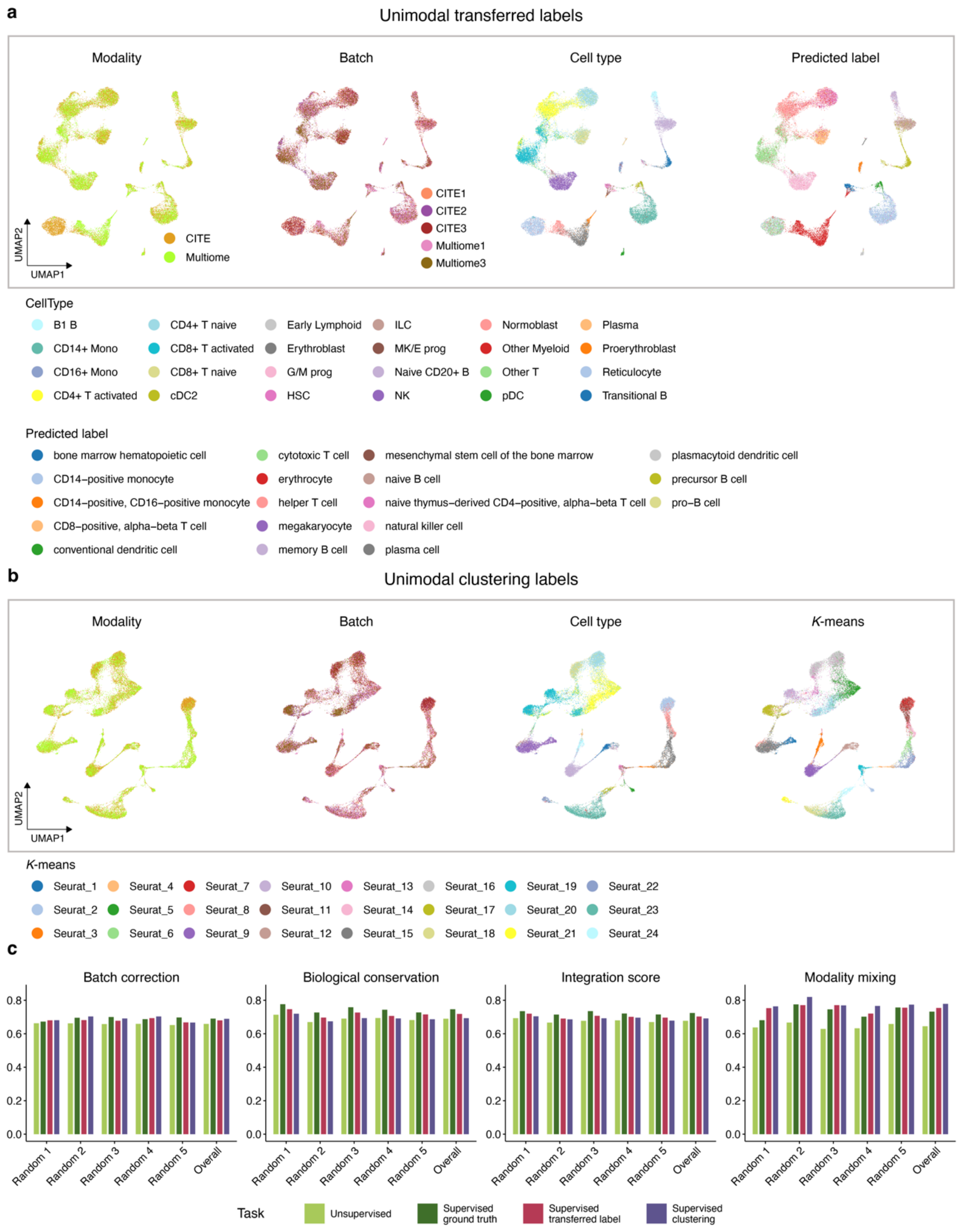

Supplementary Figure 16. Influence of label annotation strategy on the supervised integration performance of Palette. a, b. UMAP visualizations of Palette's supervised integration using two alternative unimodal-derived label sources: RNA-derived transferred labels (**a**) and labels derived from Seurat CCA–integrated transcriptomic embeddings followed by *k*-means clustering (**b**). Cells are colored by modality composition (first column), batch (second column), original labels (third column), and unimodal based labels (fourth column). **c.** Quantitative comparison of Palette's supervised integration under

different annotation conditions, including the two unimodal-derived label sets, the original dataset labels, and an unsupervised setting. For the two unimodal-derived label conditions, integration performance was evaluated using the dataset's original annotations. Performance is presented separately for four evaluation metrics: batch correction, biological conservation, overall integration, and modality mixing. For *k*-means, we specified the number of clusters to match the number of known cell types.

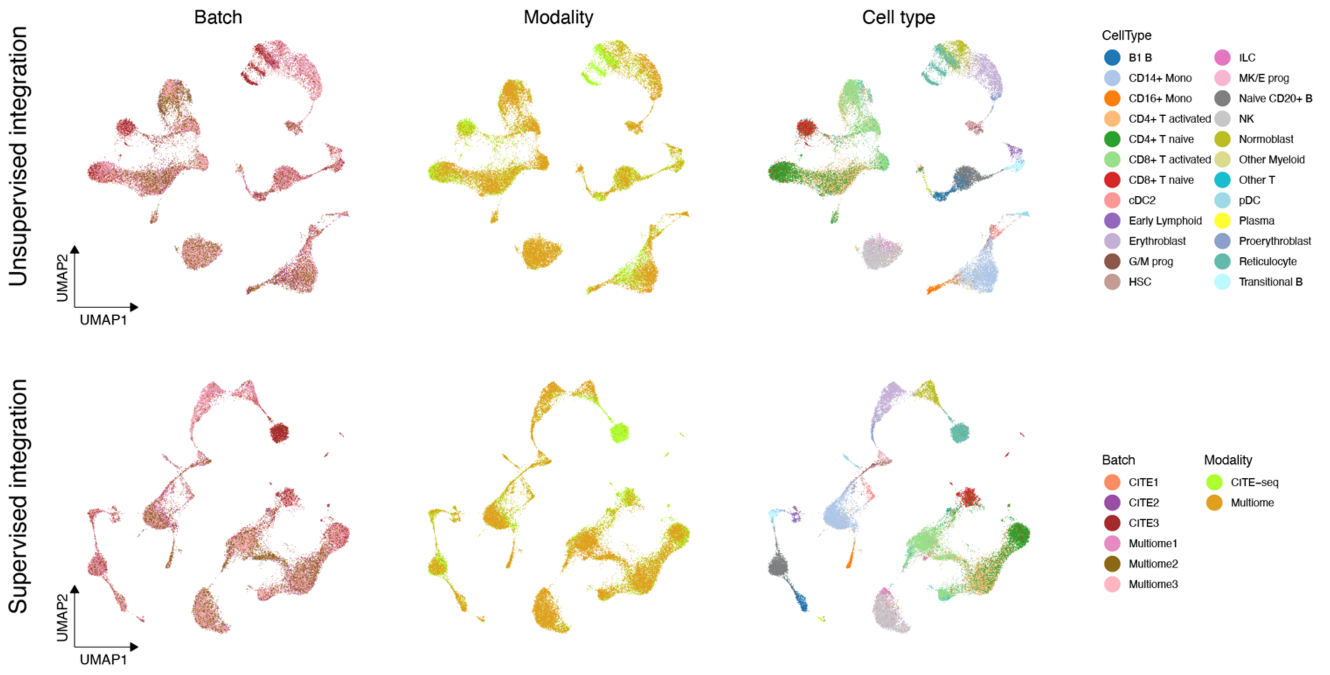

Supplementary Figure 17. UMAP visualizations of human BMMC reference data integrated by Palette in unsupervised (top row) and supervised (bottom row) modes. Cells are colored by batch (left column), modality composition (middle column), and cell type (right column).

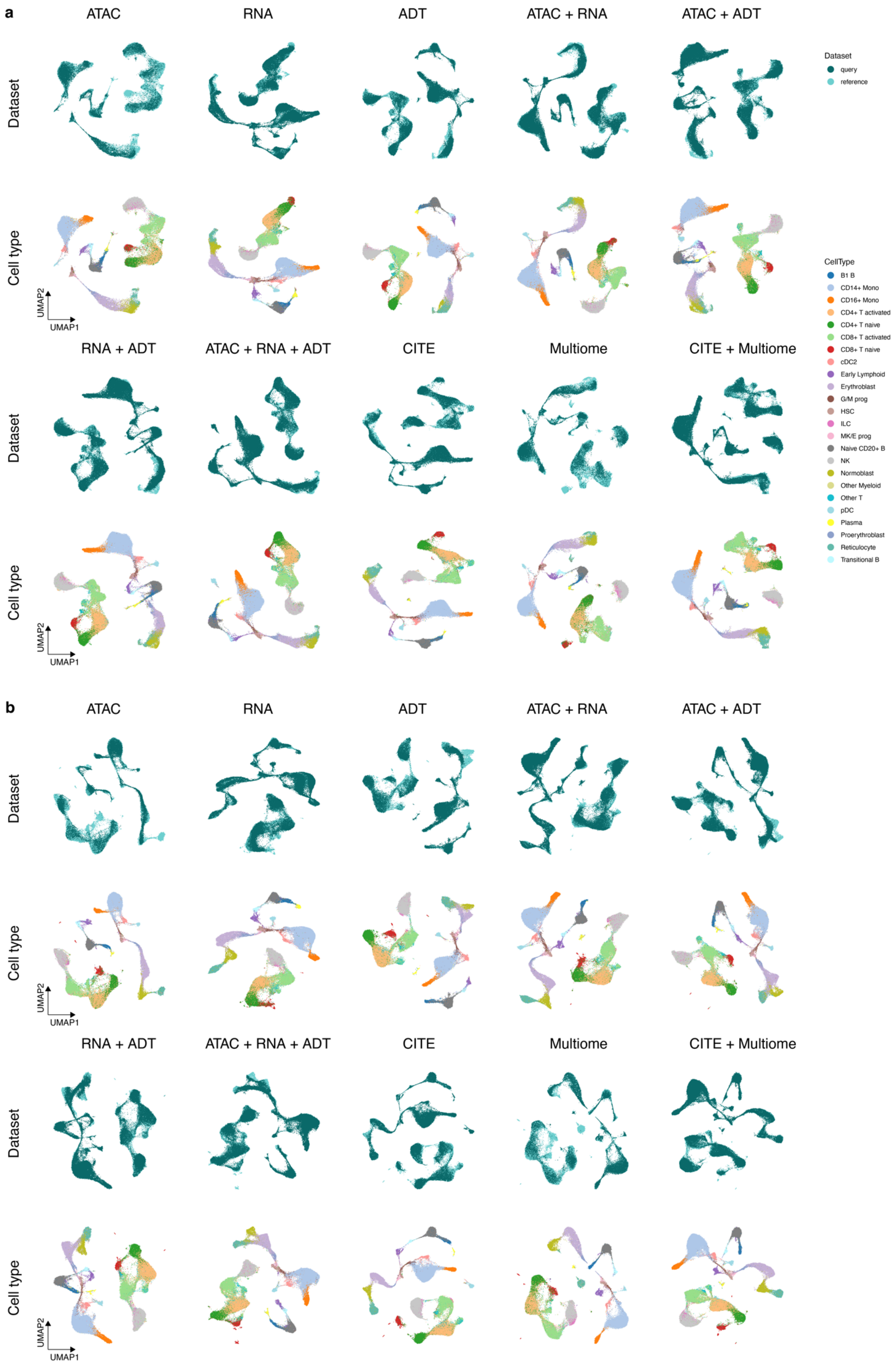
a
ATAC
RNA
ADT
ATAC + RNA
ATAC + ADT
Dataset
Cell type
UMAP2
UMAP1
RNA + ADT
ATAC + RNA + ADT
CITE
Multiome
CITE + Multiome
Dataset
query
reference
CellType
B1 B
CD14+ Mono
CD16+ Mono
CD4+ T activated
CD4+ T naive
CD8+ T activated
CD8+ T naive
cDC2
Early Lymphoid
Erythroblast
G/M prog
HSC
ILC
MK/E prog
Naive CD20+ B
NK
Normoblast
Other Myeloid
Other T
pDC
Plasma
Proerythroblast
Reticulocyte
Transitional B
b

Supplementary Figure 18. UMAP visualizations of query integration results using Palette's reference-based integration, with reference embeddings generated in the unsupervised (a) and supervised (b) modes. Cells are colored by dataset (first and third rows in **a** and **b**) and by cell type (second and fourth rows in **a** and **b**).

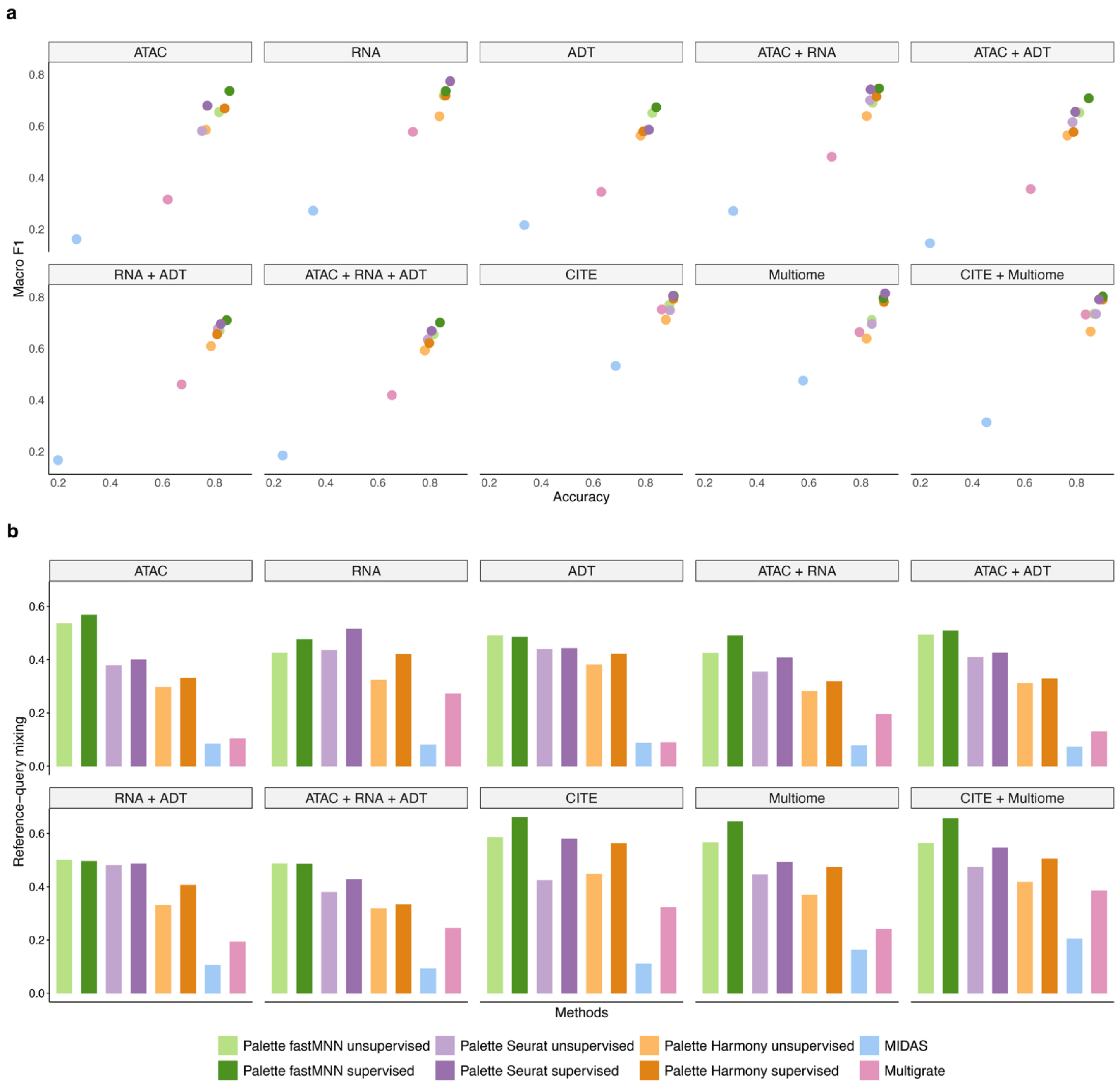

Supplementary Figure 19. Evaluation of cell type label transfer and dataset mixing in reference-based integration across multiple query scenarios. a. Scatter plots showing label transfer performance for each query scenario, measured by accuracy (x-axis) and macro F1 score (y-axis). **b.** Bar plots showing the mixing scores between reference and query cells for each query scenario. In the Palette reference-based integration framework, we incorporated three integration methods (fastMNN, Seurat v3, and Harmony), using fastMNN as the default. Reference embeddings were generated in either unsupervised or supervised mode.

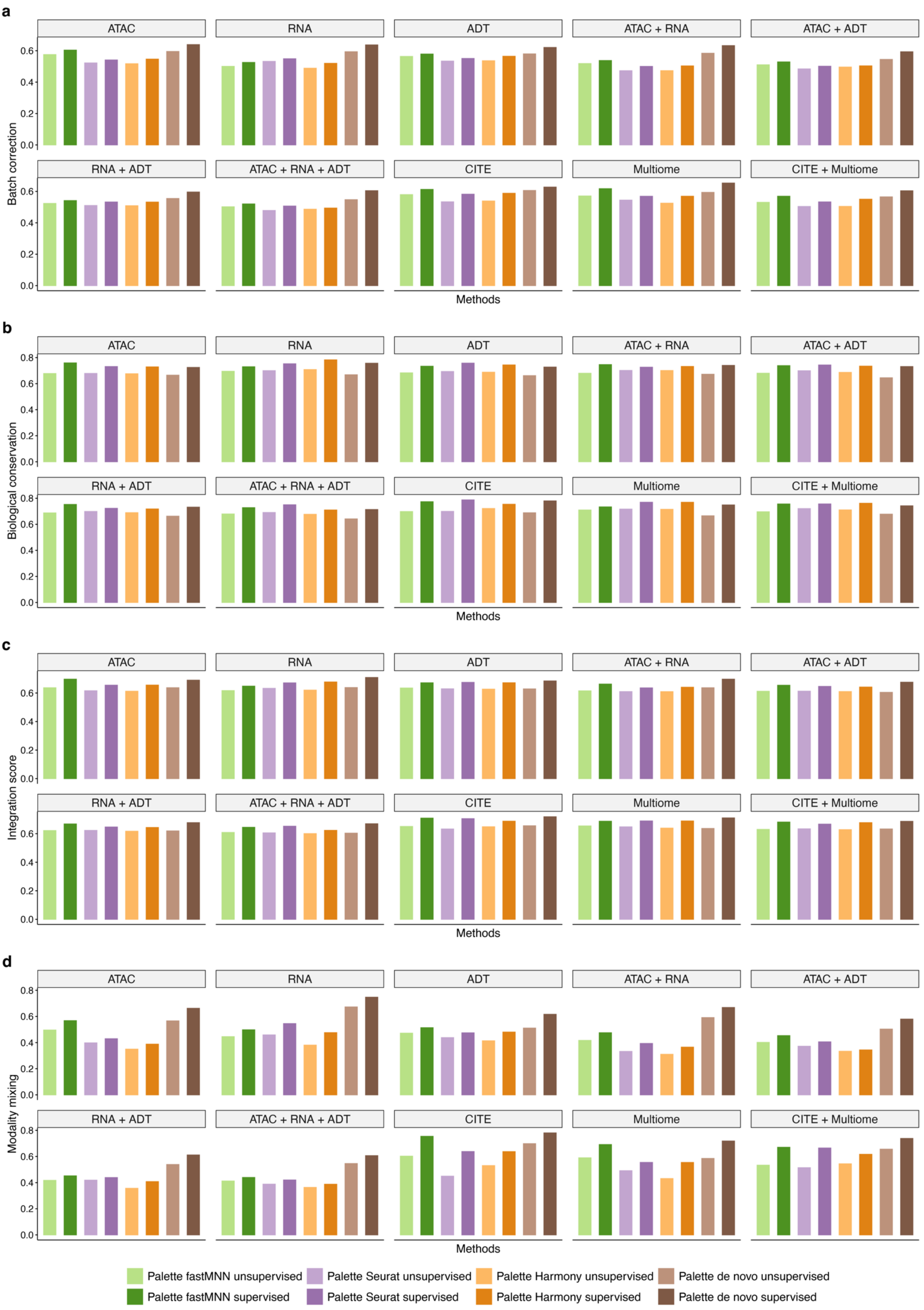

a
b
c
d
ATAC
RNA
ADT
ATAC + RNA
ATAC + ADT
RNA + ADT
ATAC + RNA + ADT
CITE
Multiome
CITE + Multiome
Batch correction
Biological conservation
Integration score
Modality mixing
Methods
0.0
0.2
0.4
0.6
0.8
Palette fastMNN unsupervised
Palette fastMNN supervised
Palette Seurat unsupervised
Palette Seurat supervised
Palette Harmony unsupervised
Palette Harmony supervised
Palette de novo unsupervised
Palette de novo supervised

Supplementary Figure 20. Comparison of integration performance between reference-based and de novo integration across multiple query scenarios. We quantitatively evaluated four integration metrics: (**a**) batch correction score, (**b**) biological conservation score, (**c**) overall integration score, and (**d**) modality mixing score. For supervised de novo integration, cell type labels for each query dataset were obtained by transferring annotations from the reference using reference-based integration with supervised reference embeddings. For evaluation purposes, the ground truth cell type labels of the query datasets were used to compute all metrics.

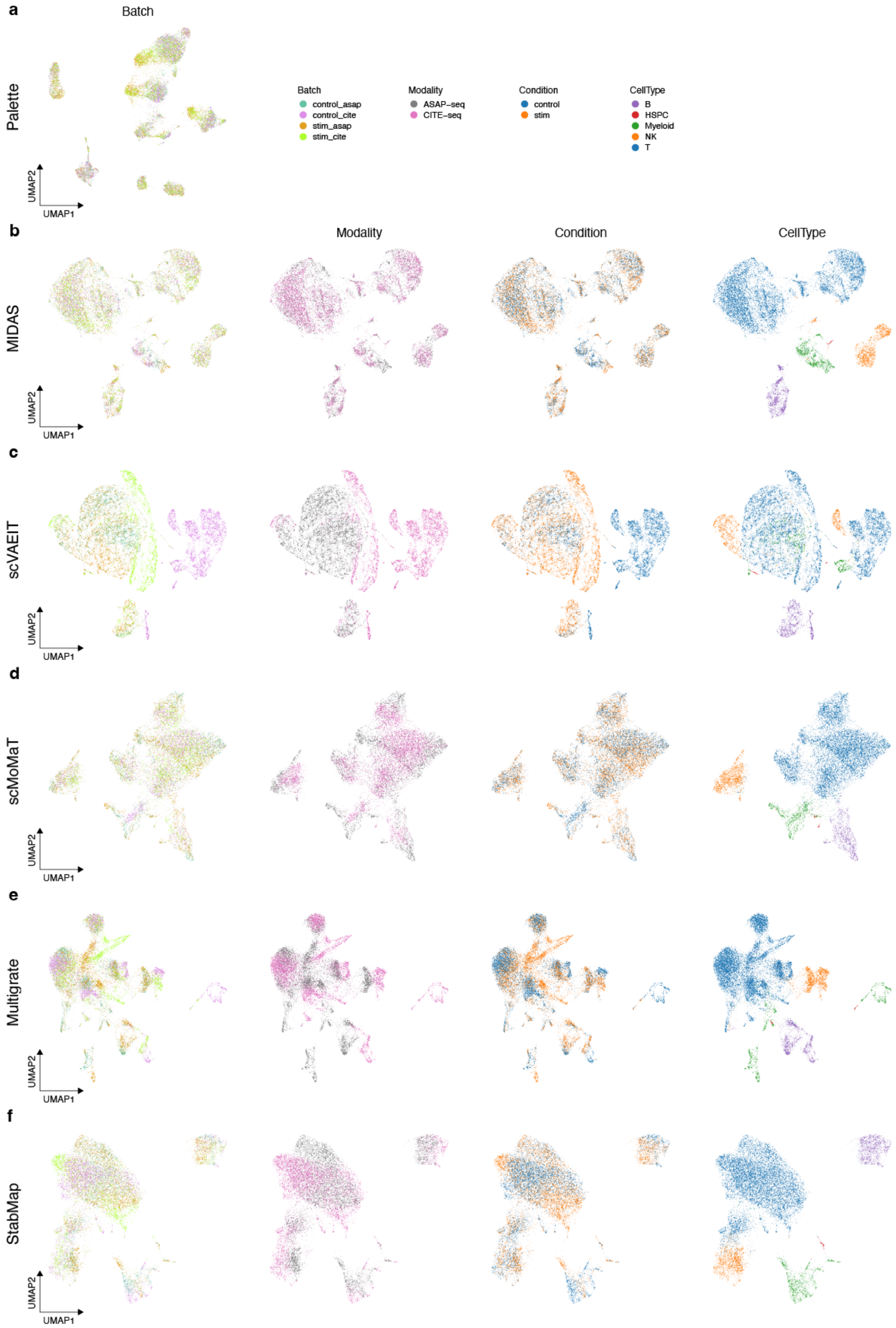

Supplementary Figure 21. UMAP visualizations of integrated cell embeddings generated by Palette and five other integration methods for cross-condition human PBMC data (a–f). Cells are colored by batch (first column), modality composition (second column), condition (third column), and cell type (fourth column).

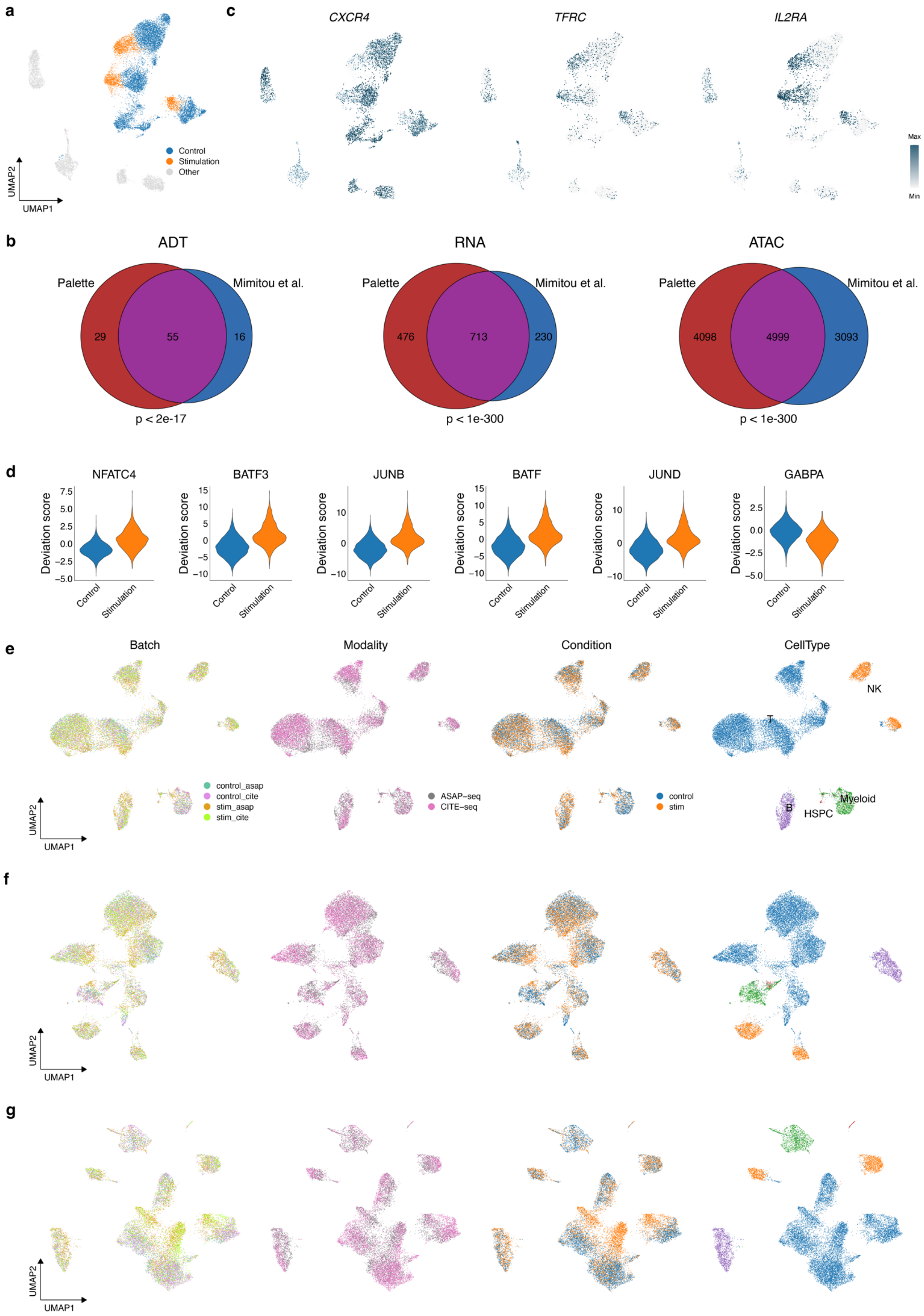
a
Control
Stimulation
Other
UMAP2
UMAP1
c
CXCR4
TFRC
IL2RA
Max
Min
b
ADT
Palette
Mimitou et al.
29
55
16
p < 2e-17
RNA
Palette
Mimitou et al.
476
713
230
p < 1e-300
ATAC
Palette
Mimitou et al.
4098
4999
3093
p < 1e-300
d
NFATC4
BATF3
JUNB
BATF
JUND
GABPA
Deviation score
Control
Stimulation
e
Batch
Modality
Condition
CellType
control_asap
control_cite
stim_asap
stim_cite
ASAP−seq
CITE−seq
control
stim
NK
T
B
HSPC
Myeloid
f
g

Supplementary Figure 22. Palette enables the preservation of condition-specific biological signals during mosaic integration. a. UMAP visualization of clustering results based on integrated cell embeddings generated by Palette. The clusters were divided into a control-enriched group, a stimulation-enriched group, and another group. **b.** Venn diagrams showing the number of shared DEFs identified by both Palette and the original study across three modalities. We observed highly significant agreement, with $p < 2e\text{-}17$ for ADT and $p < 1e\text{-}300$ for RNA and ATAC. **c**. UMAP visualizations of expression patterns of condition-related DE genes. **d.** Violin plots showing the expression of DE transcription factor motifs across conditions. **e–g.** UMAP visualizations of integrated cell embeddings generated by Palette using datasets with DEFs removed: DEFs identified by Palette (**e**), DEFs identified by Mimitou et al. (**f**), and randomly removing the same number of features from each modality as a control (**g**). Cells are colored by batch (first column), modality composition (second column), condition (third column), and cell type (fourth column).

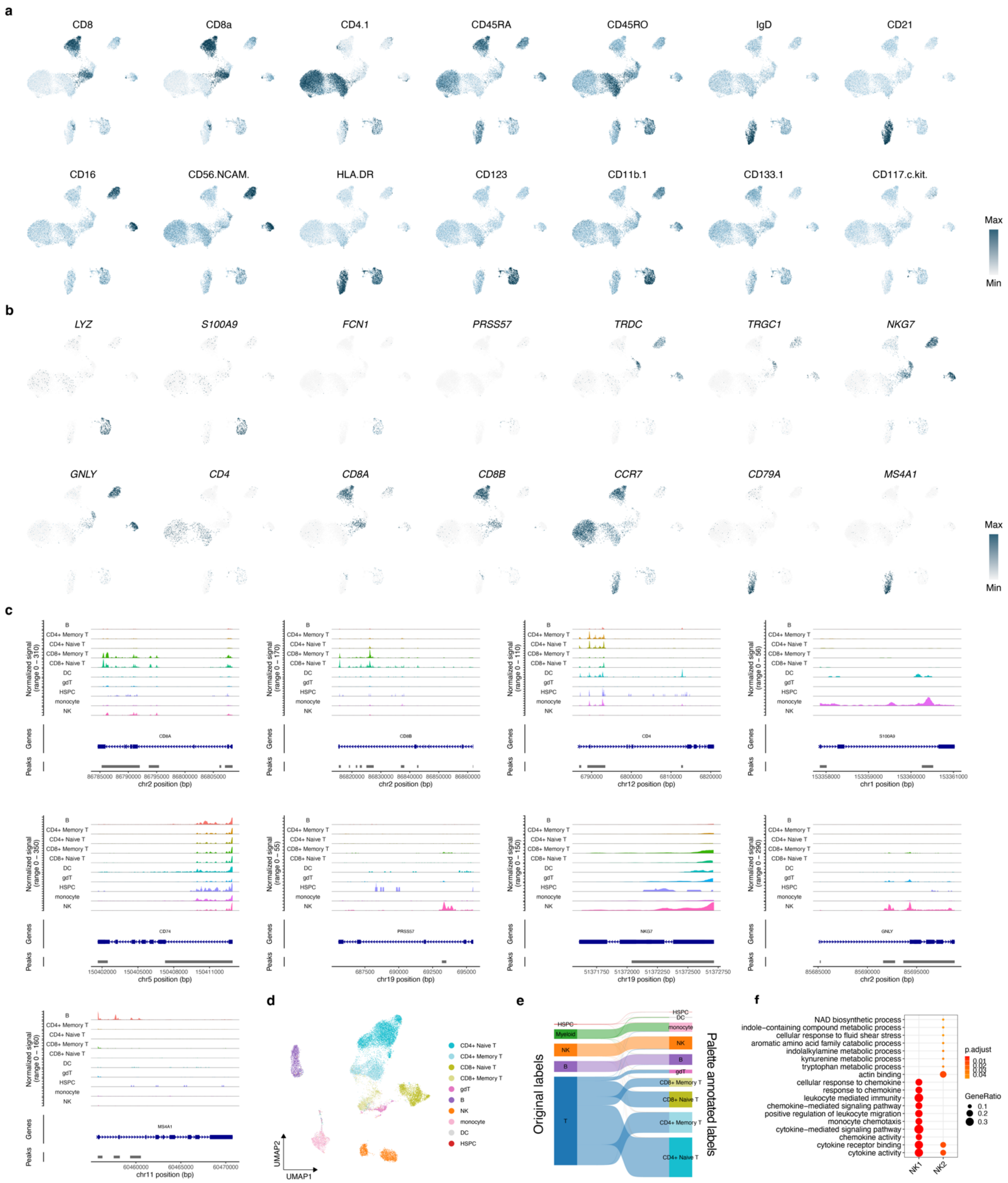

Supplementary Figure 23. Analysis of the Palette integration results after removal of Palette-identified DEFs. **a–c.** Expression patterns of markers for major human PBMC cell types across ADT (**a**), RNA (**b**), and ATAC (**c**). **d.** UMAP visualization of fine-grained cell type distributions based on the original cross-condition human PBMC data, integrated by Palette without DEF removal. **e.** Sankey diagram showing

the correspondence between the original cell type labels and the fine-grained labels. **f.** Top 10 enriched GO terms for DE genes between the two NK cell subpopulations.

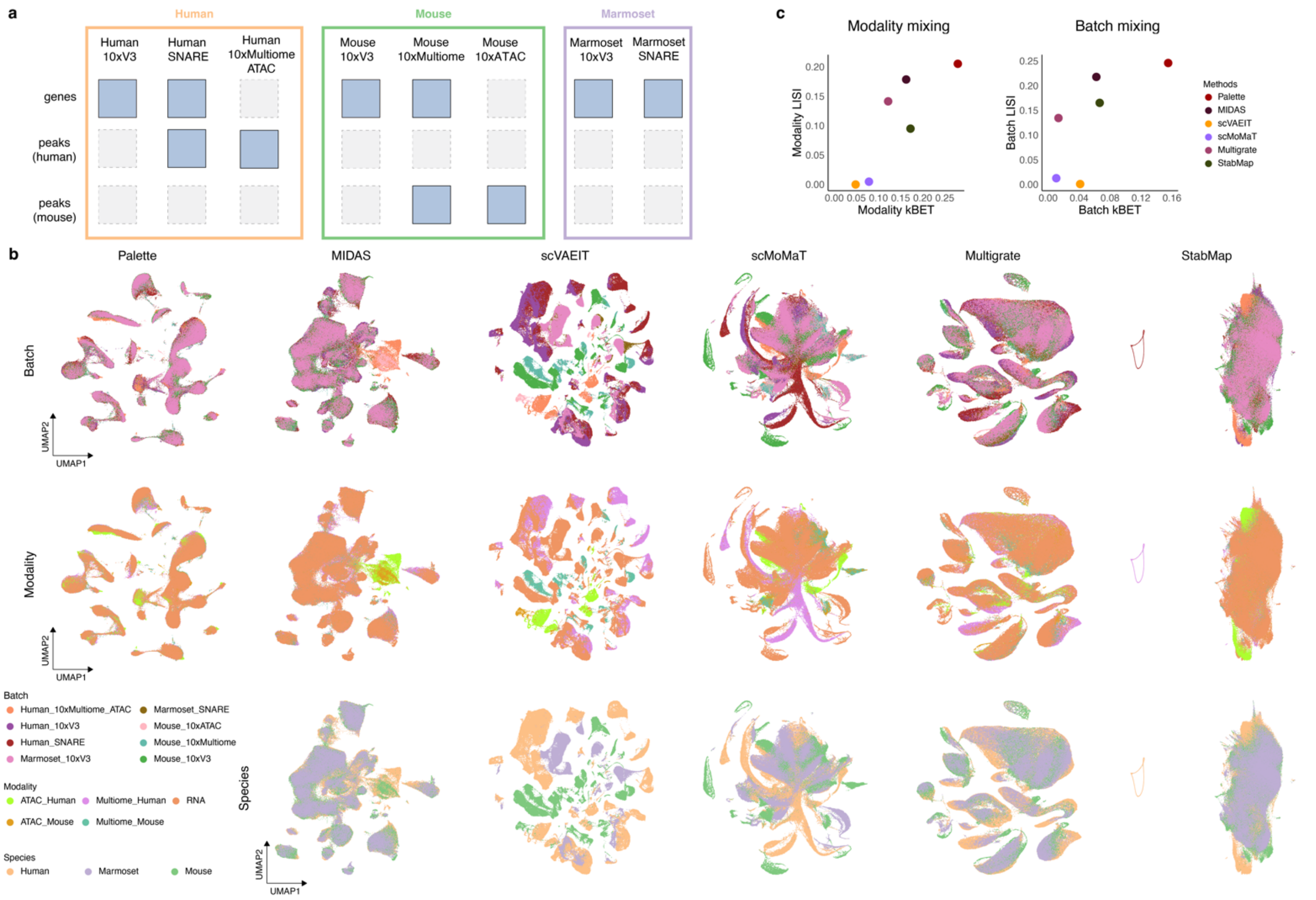

Supplementary Figure 24. Cross-species MOp data integration results. a. Schematic illustration of the modality composition and species of each batch in the cross-species MOp dataset. **b.** UMAP visualizations of integrated cell embeddings generated by Palette and five other integration methods for the cross-species MOp data. Cells are colored by batch (top row), modality composition (middle row), and species (bottom row). **c.** Comparison of modality (left) and batch (right) mixing scores across integration methods. Due to the lack of unified cell type labels, the modality mixing effect of the integrated embeddings was assessed using kBET and LISI, rather than the previously defined modality mixing score.

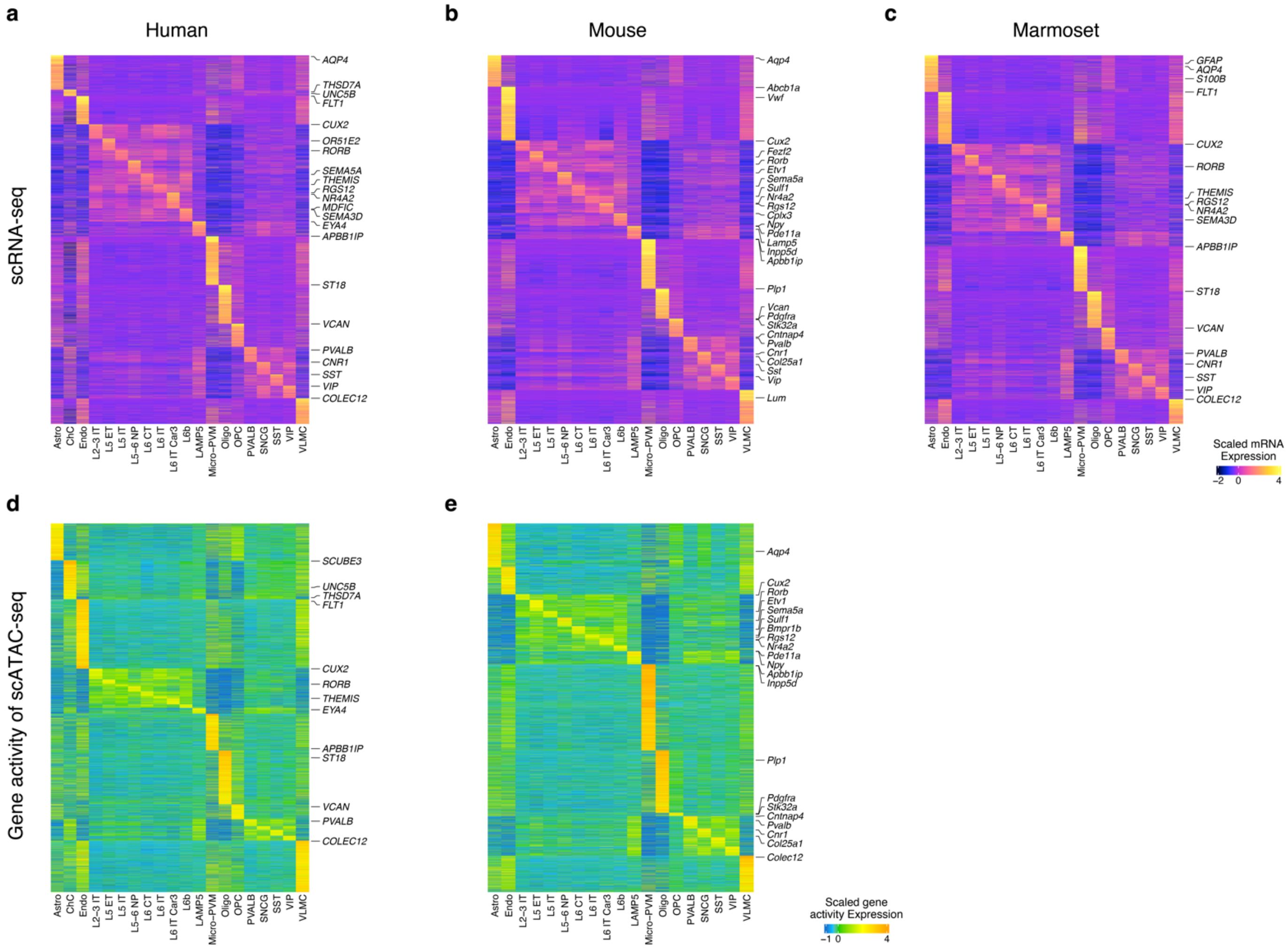

Supplementary Figure 25. Gene expression patterns across cell types and species in the cross-species MOp dataset. Heatmaps showing row-scaled normalized expression values of top DEGs across cell types within each species: human (left column; **a, d**), mouse (middle column; **b, e**), and marmoset (right column; **c**). The top row (**a–c**) shows transcriptomic data, while the bottom row (**d, e**) presents chromatin accessibility data, represented by row-scaled normalized gene activity scores. Representative lineage marker genes are labeled with gene names. DE analysis was performed separately for each species and modality based on cell type annotations using the FindAllMarkers function from the Seurat R package. DE genes were identified using unified criteria: $p < 0.05$ and log fold change > 0.5.

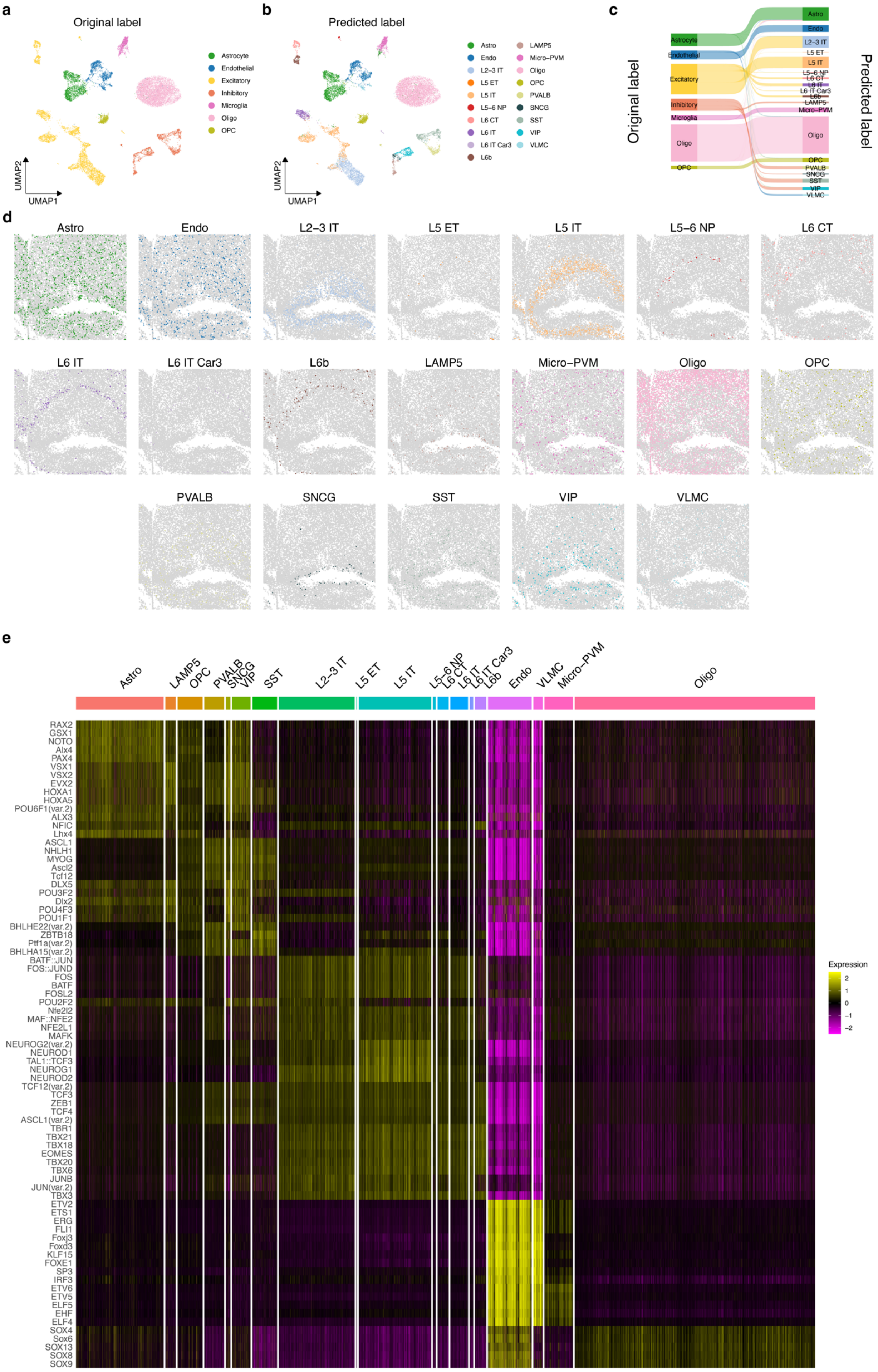

a
Original label
Astrocyte
Endothelial
Excitatory
Inhibitory
Microglia
Oligo
OPC
UMAP1
UMAP2
b
Predicted label
Astro
Endo
L2–3 IT
L5 ET
L5 IT
L5–6 NP
L6 CT
L6 IT
L6 IT Car3
L6b
LAMP5
Micro–PVM
Oligo
OPC
PVALB
SNCG
SST
VIP
VLMC
c
Original label
Predicted label
d
e
Expression

Supplementary Figure 26. Reference-based integration results of human cortex Slide-tags data. a, b. UMAP visualization of cell embeddings from the human cortex Slide-tags data, colored by original (**a**) and predicted (**b**) cell type labels. **c.** Sankey diagram showing the correspondence between original and predicted labels. **d.** Spatial distribution of individual cell types. **e.** Heatmap of transferred motif activity scores for the top five DE transcription factor motifs per cell type. DE analysis was performed across cell types using the FindAllMarkers function from the Seurat R package. DE motifs were identified using unified criteria: $p < 0.05$ and log fold change > 0.3.

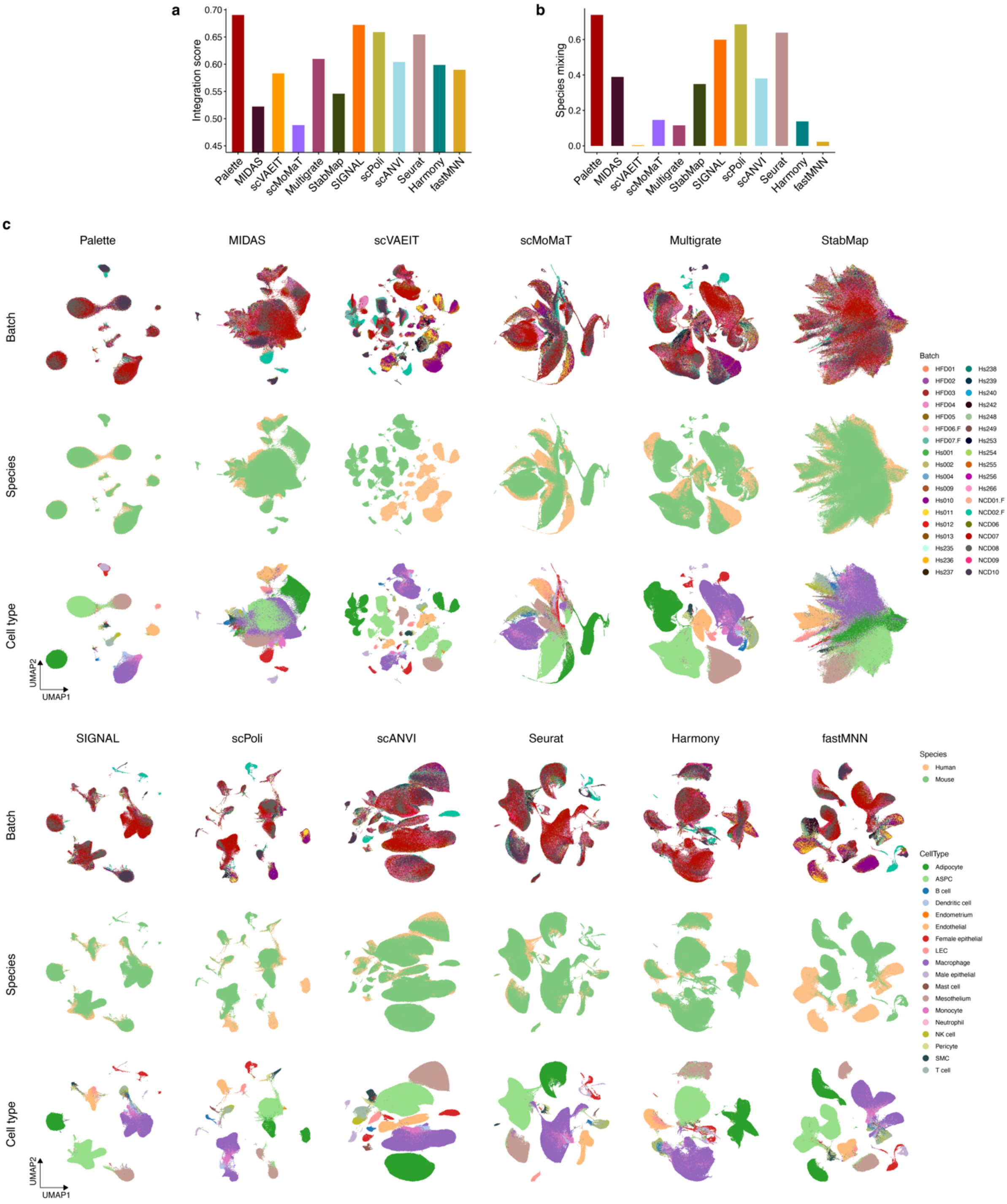

Supplementary Figure 27. Integration results of cross-species WAT data based on orthologous genes. **a, b.** Comparison of overall integration scores (**a**) and species mixing scores (**b**) across different integration methods. **c.** UMAP visualizations of integrated cell embeddings generated by Palette and five other integration methods for the cross-species WAT data. Orthologous genes between human and mouse were used as features for integration. Cells are colored by batch (first and fourth rows), species (second and fifth rows), and cell type (third and sixth rows).

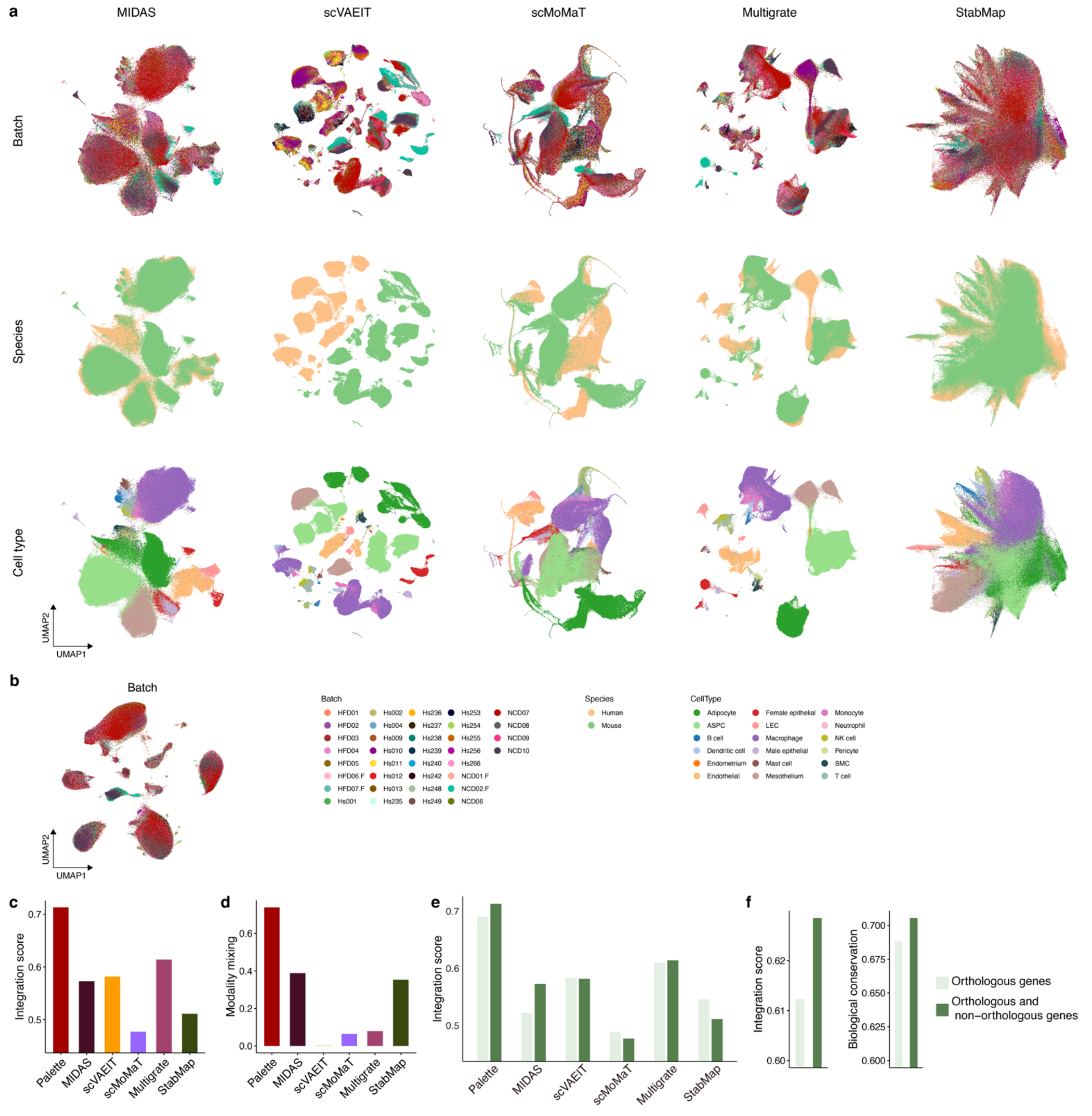

Supplementary Figure 28. Comparison of integration performance with and without inclusion of non-orthologous genes. a. UMAP visualizations of integrated cell embeddings generated by five competing mosaic integration methods for the cross-species WAT dataset, using both orthologous and non-orthologous genes as features. Cells are colored by batch (top row), species (middle row), and cell type (bottom row). **b.** UMAP visualization of integrated cell embeddings generated by Palette under the same feature setting, colored by batch. **c, d.** Comparison of overall integration scores (**c**) and modality mixing

scores (**d**) across methods under the non-orthologous gene inclusion setting. **e.** Bar plot comparing overall integration scores across integration methods using different gene sets. For each method, the left bar indicates performance using only orthologous genes, while the right bar represents performance using both orthologous and non-orthologous genes. **f.** Bar plots comparing overall integration (left) and biological conservation (right) scores for different gene sets in Palette unsupervised integration.

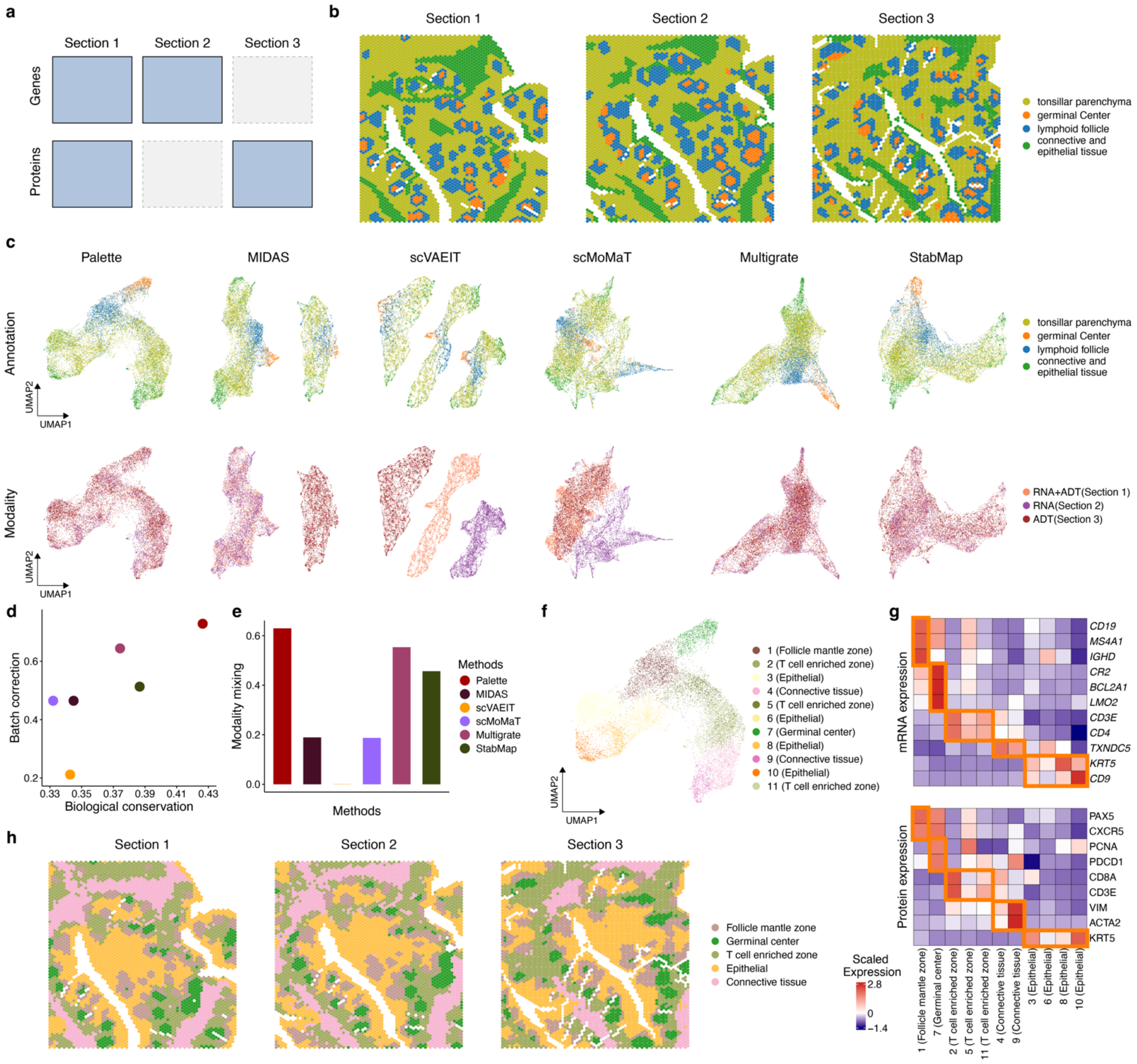

Supplementary Figure 29. Integration results of human tonsil 10x Visium data. a. Schematic illustration of the modality composition for each batch in the human tonsil 10x Visium dataset. **b.** Spatial distribution of original expert annotations. **c.** UMAP visualizations of integrated cell embeddings generated by Palette and five other integration methods. Cells are colored by original expert annotations (top row) and modality (bottom row). **d.** Scatter plot of biological conservation scores versus batch correction scores for different integration methods. **e.** Comparison of modality mixing scores across methods. **f.** UMAP visualization of integrated cell embeddings generated by Palette, colored by Leiden clustering results. **g.**

Expression patterns of selected markers across transcriptomic and proteome layers. **h.** Spatial distribution of annotations derived from Palette-integrated embeddings.

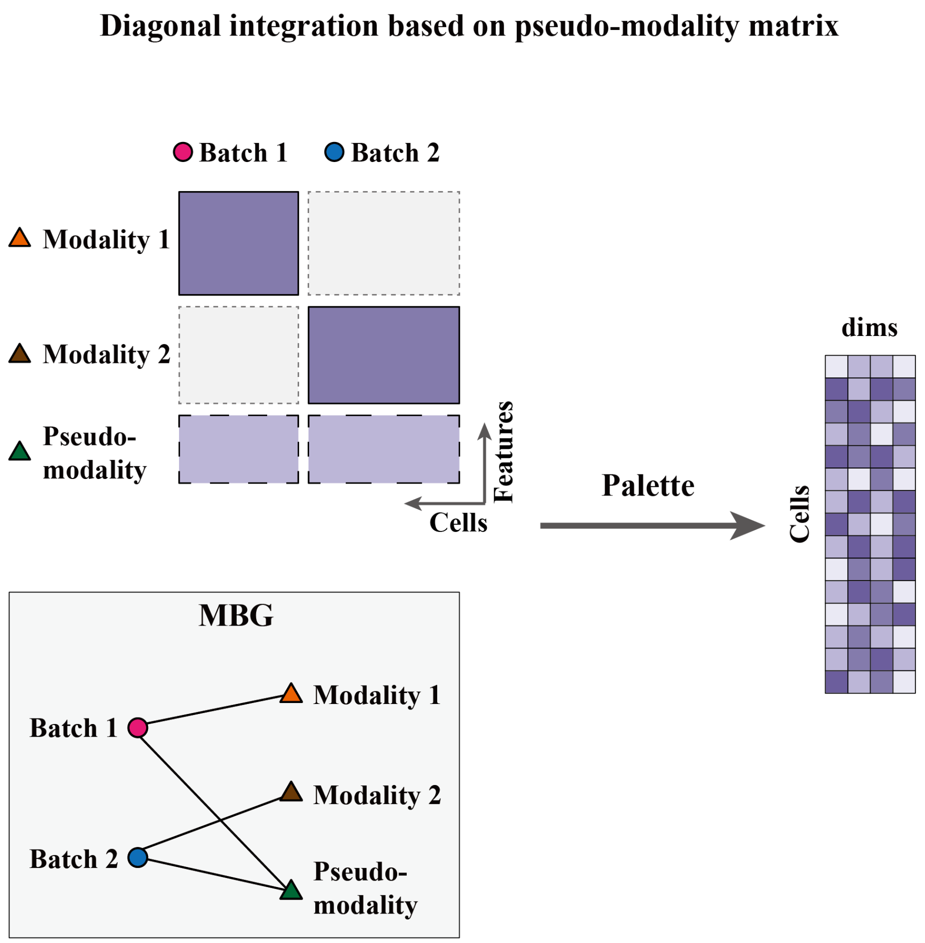

Supplementary Figure 30. Schematic illustration of Palette diagonal integration strategy.

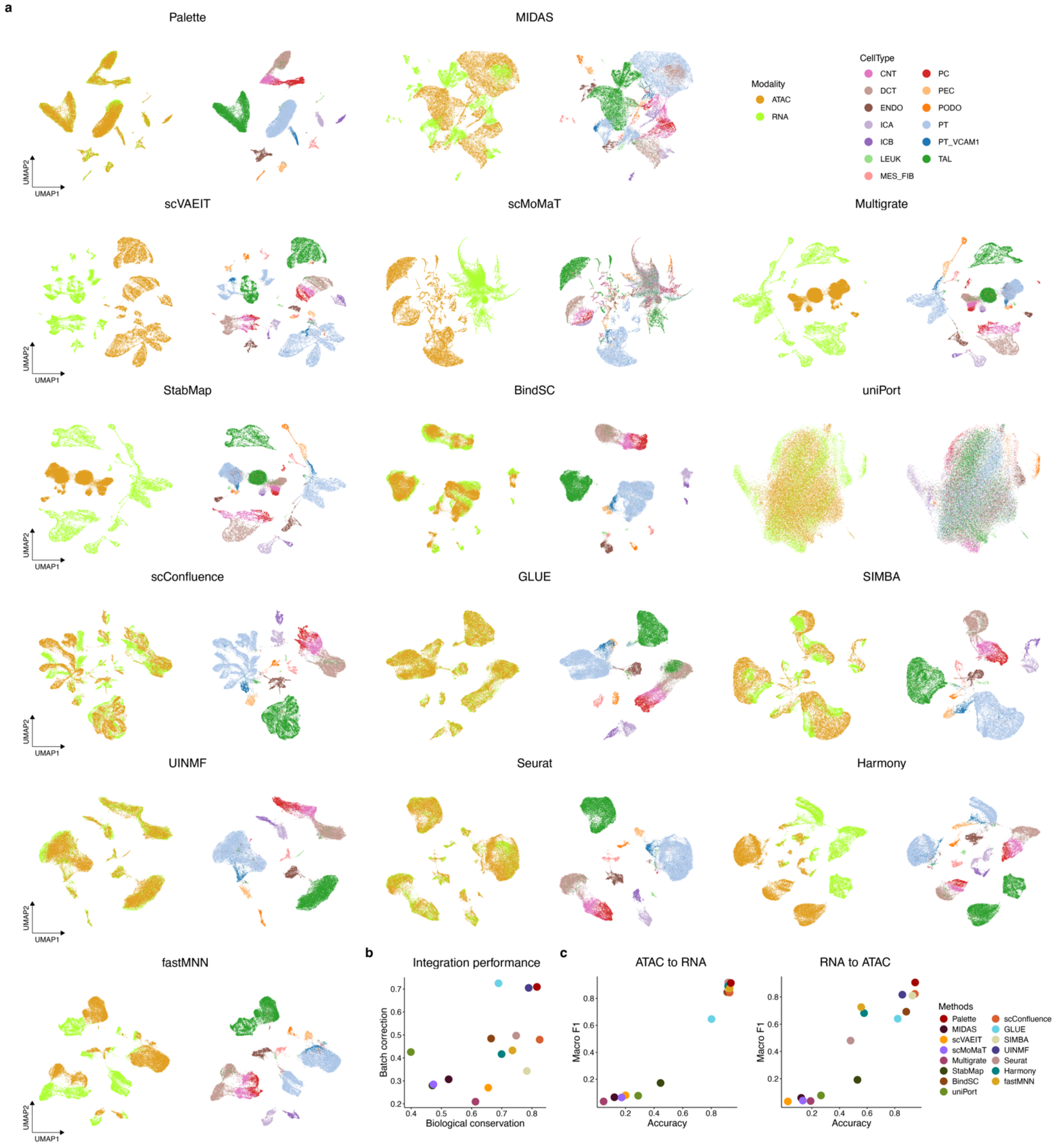

Supplementary Figure 31. Diagonal integration results for the human kidney data. a. UMAP visualizations of integrated cell embeddings generated by Palette and 14 other integration methods. Cells are colored by modality and cell type labels. **b.** Scatter plot of biological conservation scores versus batch correction scores for different integration methods. **c.** Comparison of cross-modality label transfer

performance across methods. Cell type labels from the chromatin accessibility data were transferred to predict the identities of cells in the transcriptome data (left), and vice versa (right).

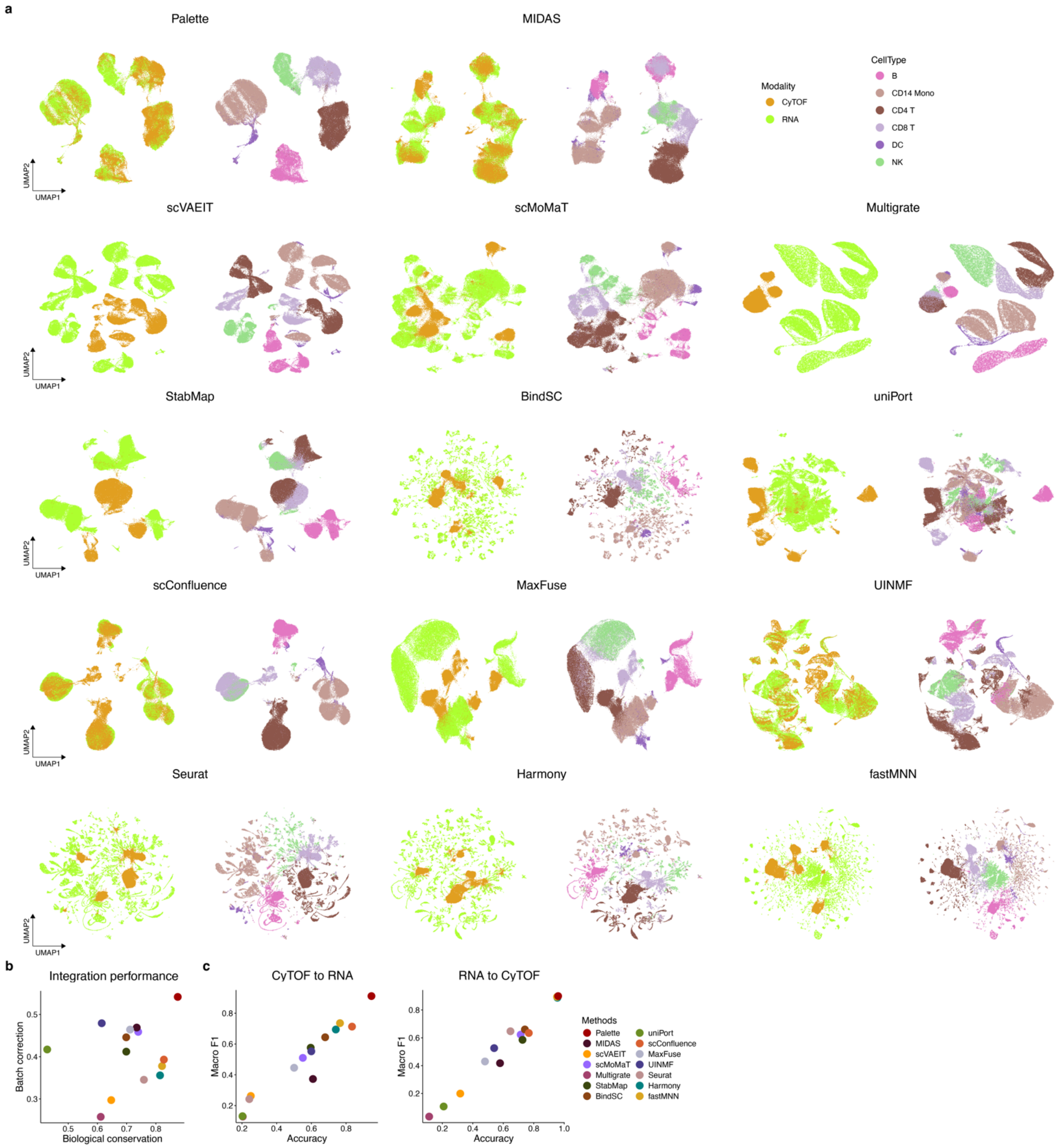

Supplementary Figure 32. Diagonal integration results for the human PBMC data. a. UMAP visualizations of integrated cell embeddings generated by Palette and 13 other integration methods. Cells

are colored by modality and cell type labels. **b.** Scatter plot of biological conservation scores versus batch correction scores for different integration methods. **c.** Comparison of cross-modality label transfer performance across methods. Cell type labels from the proteome data were transferred to predict the identities of cells in the transcriptome data (left), and vice versa (right).

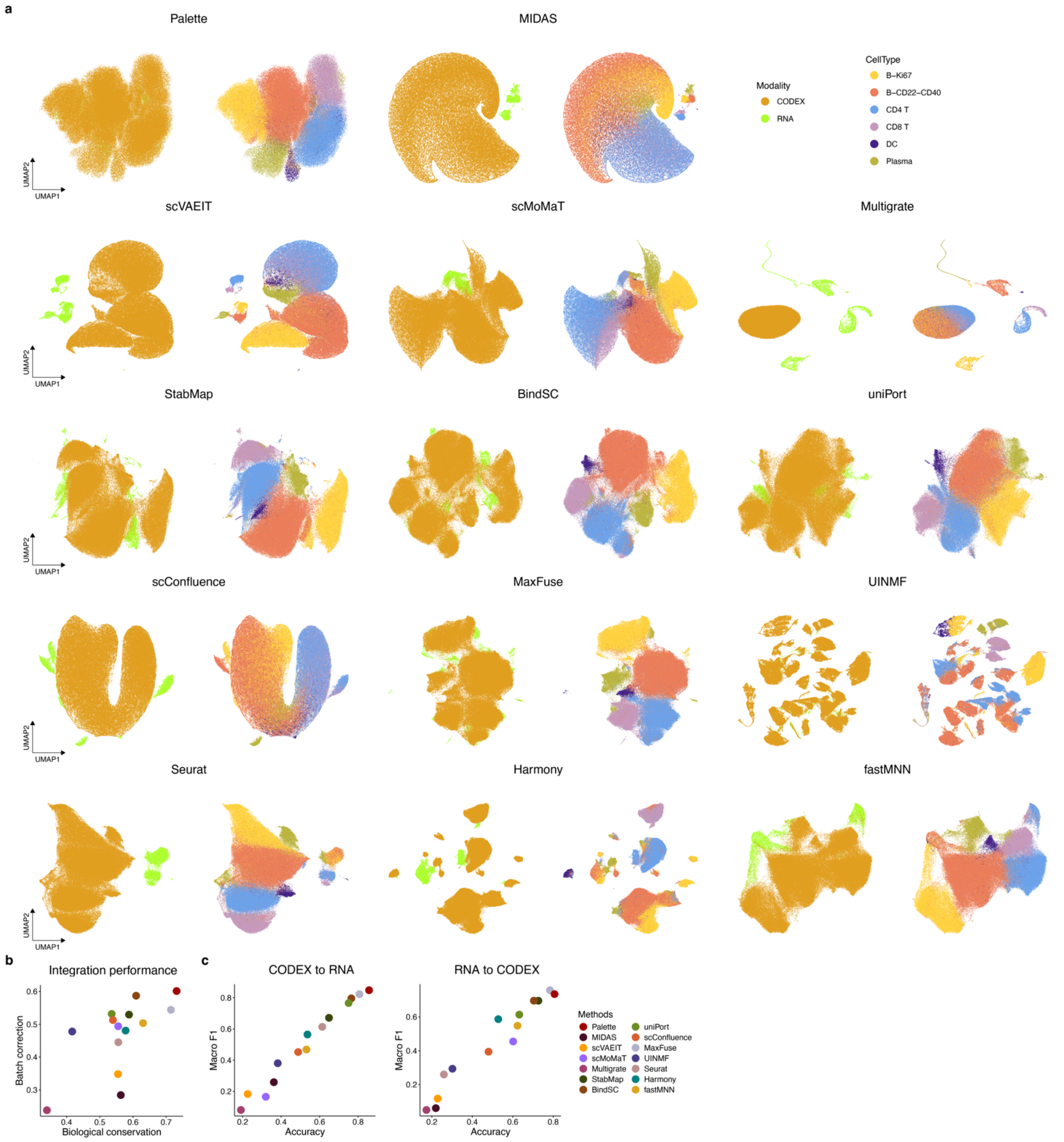

Supplementary Figure 33. Diagonal integration results for the human tonsil data. a. UMAP visualizations of integrated cell embeddings generated by Palette and 13 other integration methods. Cells are colored by modality and cell type labels. **b.** Scatter plot of biological conservation scores versus batch correction scores for different integration methods. **c.** Comparison of cross-modality label transfer performance across methods. Cell type labels from the proteome data were transferred to predict the identities of cells in the transcriptome data (left), and vice versa (right).

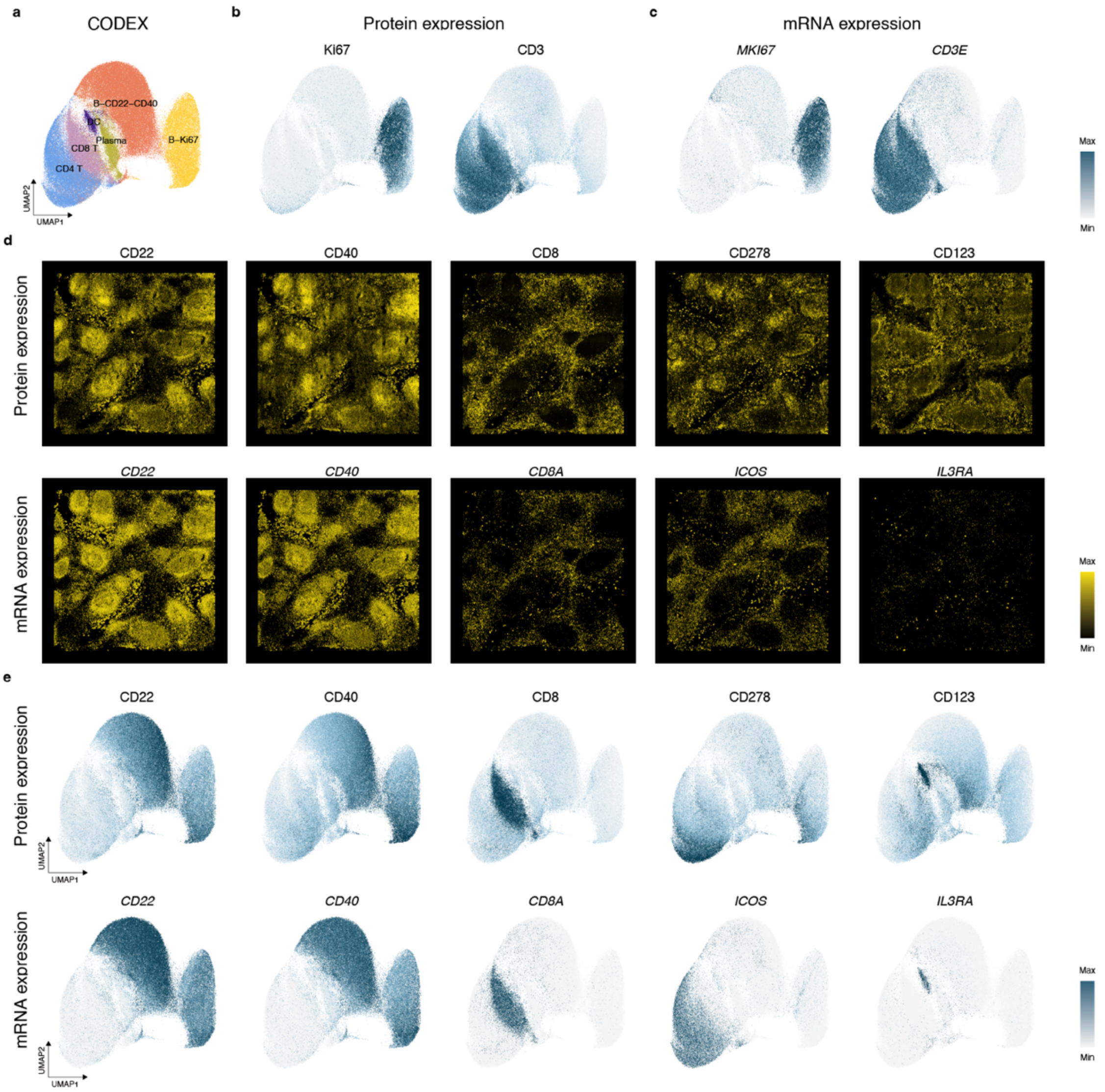

Supplementary Figure 34. Protein expression in CODEX data corresponds to the expression of their coding genes in the inferred transcriptome data. a. UMAP visualization of CODEX cell embeddings, colored by cell type labels. **b, c.** UMAP visualizations of proliferation and T cell markers at the protein (**b**)

and inferred transcriptome (**c**) levels. The corresponding spatial distributions are shown in Fig. 5c. **d, e.** Spatial (**d**) and UMAP (**e**) visualizations of selected markers across the protein (top row) and inferred transcriptome (bottom row) layers, showing consistent expression patterns across modalities.

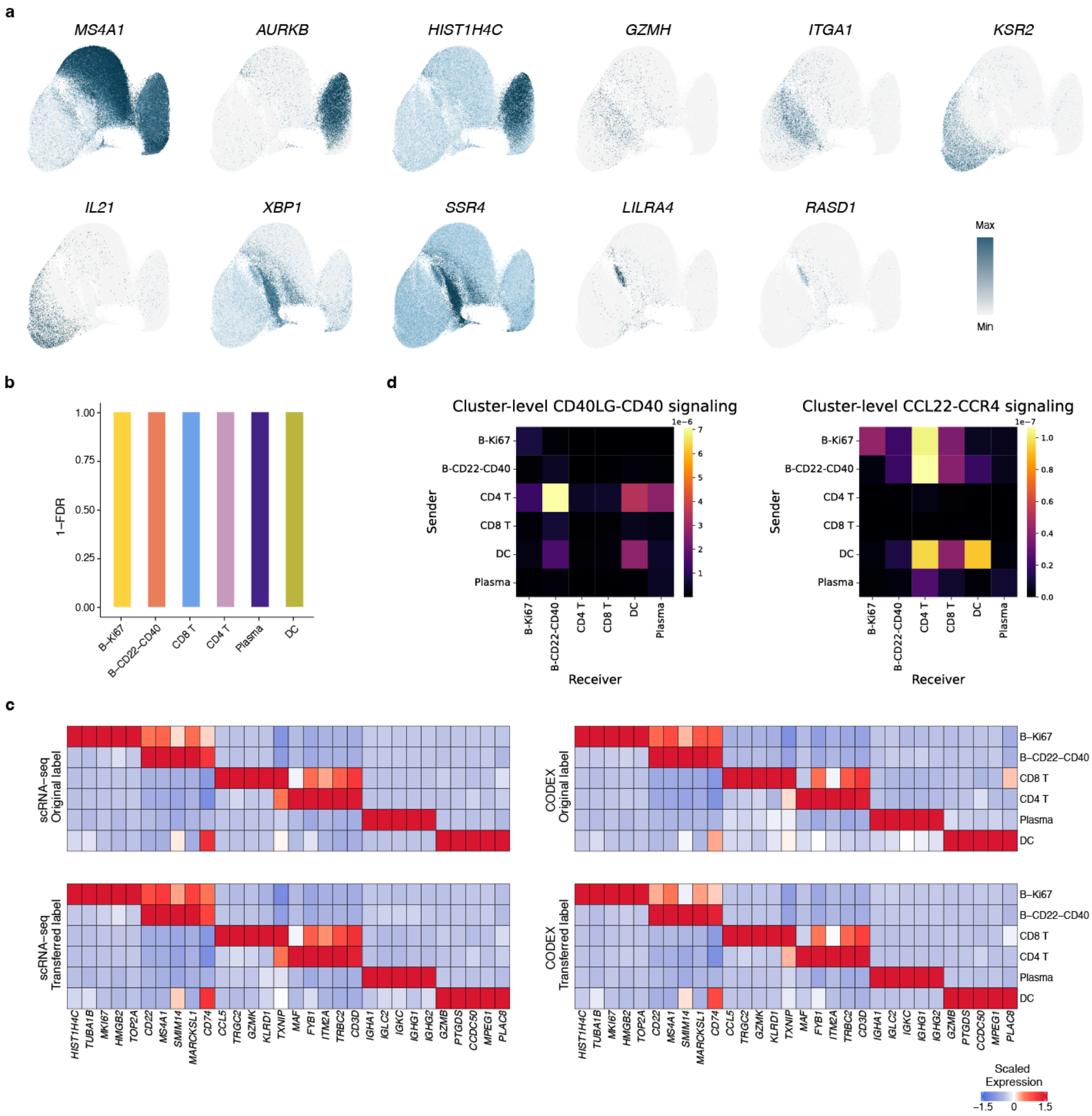

Supplementary Figure 35. Palette accurately transfers knowledge across modalities. a. UMAP visualizations of marker gene expression patterns based on the inferred transcriptome data for CODEX. These genes lack corresponding protein measurements in the CODEX data. **b.** Significance of marker gene overlap for each cell type between original and transferred labels in both scRNA-seq data and the inferred CODEX transcriptome data, assessed using a four-way Fisher's exact test. Across all comparisons, we

observed highly significant overlap, with FDR < 1e-300. **c.** Heatmaps showing expression patterns of top five DE genes under original (top row) and transferred (bottom row) cell type labels for scRNA-seq data (left column) and the inferred CODEX transcriptome data (right column). **d.** Heatmaps of cluster-level cell–cell communication for the CD40LG-CD40 (left) and CCL22-CCR4 (right) signaling pathways.

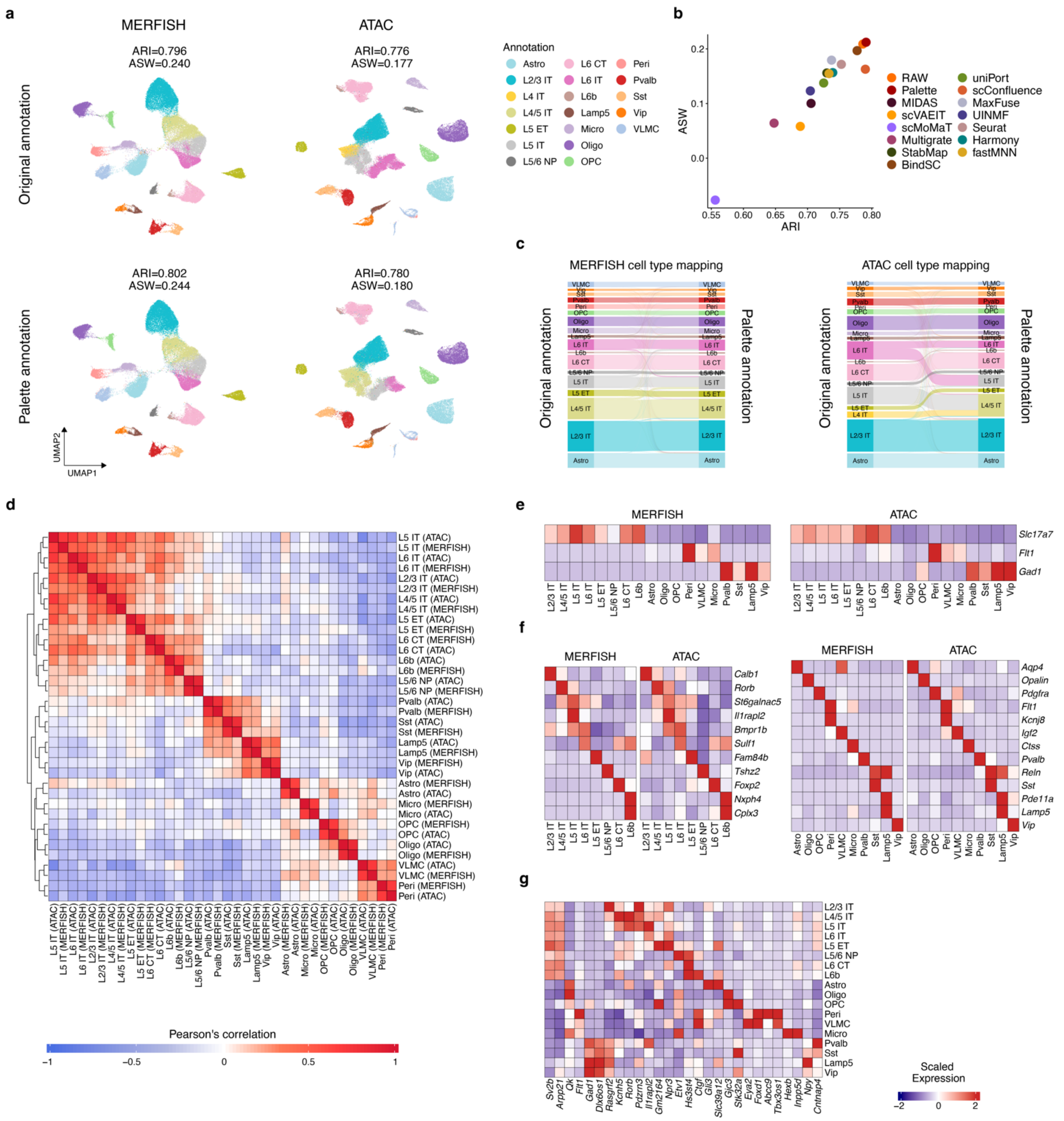

Supplementary Figure 36. Re-annotation of mouse MOp data based on Palette-integrated embeddings. a. UMAP visualizations of cell embeddings from mouse MOp MERFISH (left column) and scATAC-seq (right column) data. Cells are colored by original labels (top row) and re-annotated labels derived from Palette integration (bottom row). Re-annotated labels showing improved performance over the original annotations according to quantitative metrics, including ARI and ASW. **b.** Comparison of ARI and ASW across annotations generated by different integration methods and the original studies. **c.** Sankey diagrams showing the correspondence between original and Palette-based labels for MERFISH (left) and scATAC-seq (right) data. **d.** Correlation heatmap of Palette-based labels. The dendrogram, constructed from the correlation matrix, highlights the hierarchical relationships among re-annotated cell types. Notably, the same cell types across different modalities consistently cluster together at the root nodes of the hierarchy. **e.** Heatmaps showing representative markers expression patterns for coarse-grained cell types shared across modalities, including Glutamatergic, GABAergic, and non-neural populations. **f.** Heatmaps showing fine-grained cell type markers expression patterns corresponding to each coarse category in (**e**). Glutamatergic subtypes are shown on the left, and GABAergic and non-neural subtypes on the right. **g.** Heatmap of marker genes specific to scATAC-seq data.

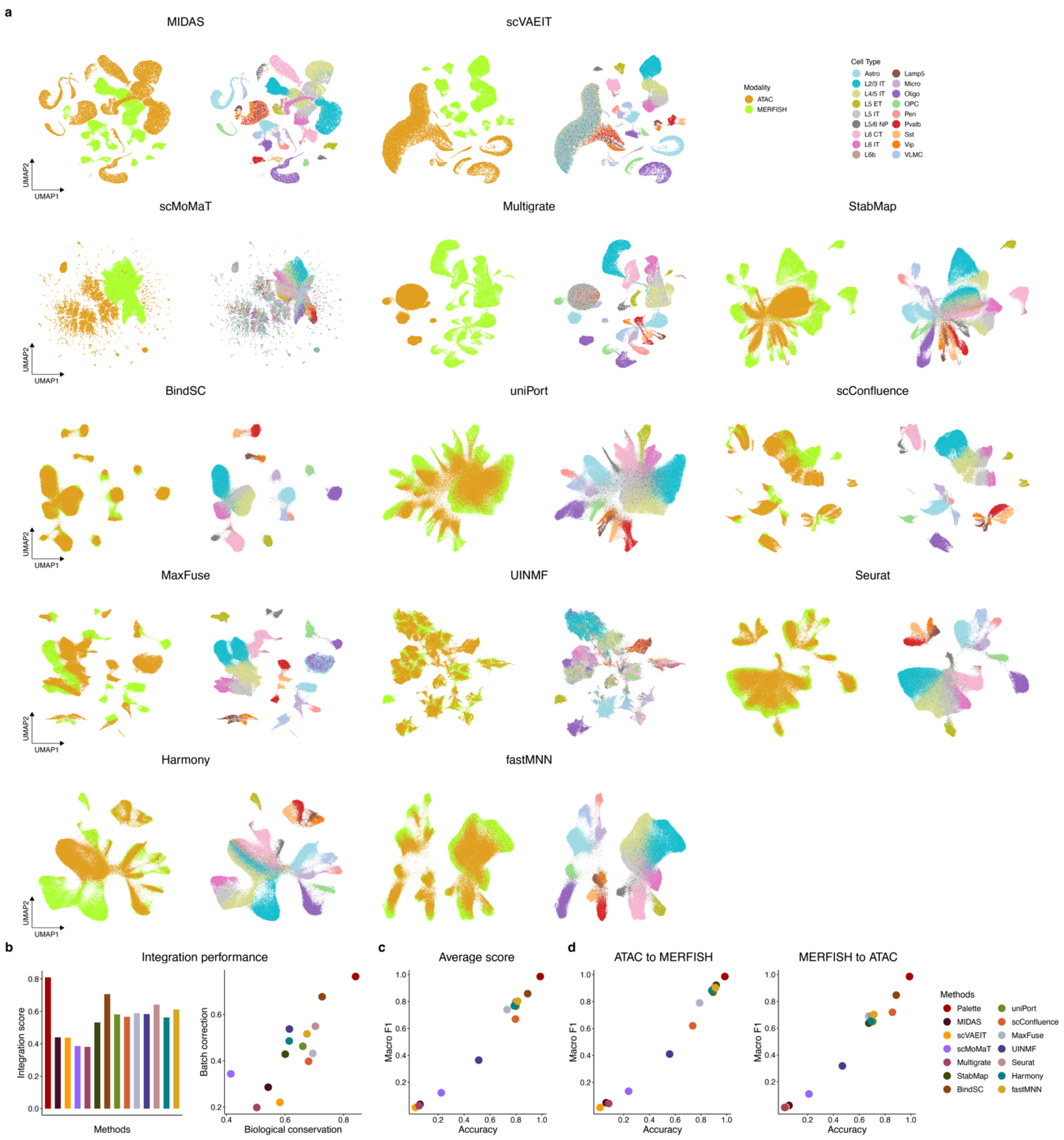

Supplementary Figure 37. Diagonal integration results for the mouse MOp data. a. UMAP visualizations of integrated cell embeddings generated by Palette and 13 other integration methods. Cells are colored by modality and Palette-based cell type labels. **b.** Comparison of integration performance across methods, with overall integration scores shown on the left, and biological conservation scores and batch correction scores on the right. **c.** Scatter plot of mean macro F1 scores versus mean accuracy scores for different integration methods. **d.** Comparison of cross-modality label transfer performance across methods.

Cell type labels from the chromatin accessibility data were transferred to predict the identities of cells in the MERFISH data (left), and vice versa (right).

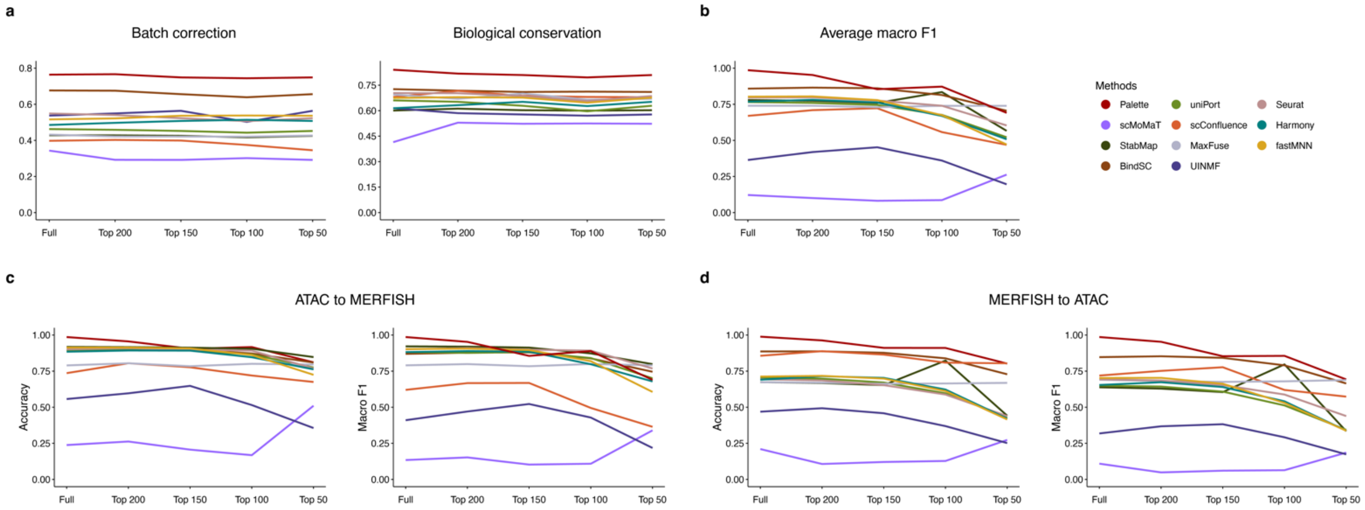

Supplementary Figure 38. Integration and label transfer performance of Palette and other methods on mouse MOp data with reduced feature overlap. a. Line plots showing batch correction (left) and biological conservation (right) scores for each method. **b.** Line plot of average macro F1 scores quantifying overall label transfer performance across methods. **c, d.** Line plots of cross-modality label transfer performance, evaluated using accuracy (left in **c** and **d**) and macro F1 (right in **c** and **d**). Cell type labels from scATAC-seq were transferred to MERFISH (**c**) and vice versa (**d**).

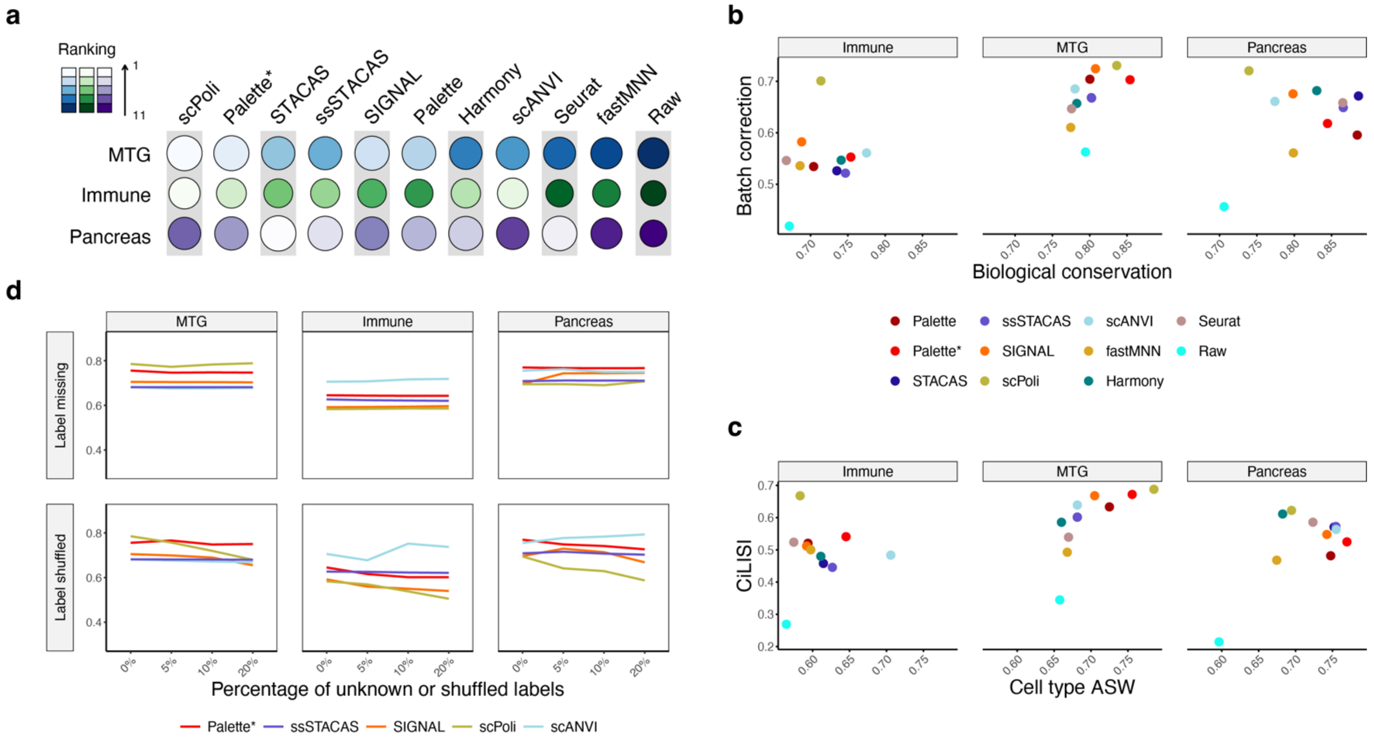

Supplementary Figure 39. Benchmarking of Palette against state-of-the-art integration methods for scRNA-seq data integration. a. Overall integration performance ranking of all methods across multiple datasets. The radius of each circle indicates the overall integration scores, ranging from 0 to 1. **b.** Dot plots comparing biological conservation scores and batch-correction scores for different integration methods across datasets. **c.** Dot plots comparing cell type ASW and CiLISI values for different integration methods across datasets. **d.** Cell type ASW values under increasing proportions of unknown (top row) and shuffled (bottom row) cell-type labels across multiple datasets. The supervised mode of Palette model is denoted as ‘Palette*’, whereas ‘Palette’ refers to the unsupervised mode.

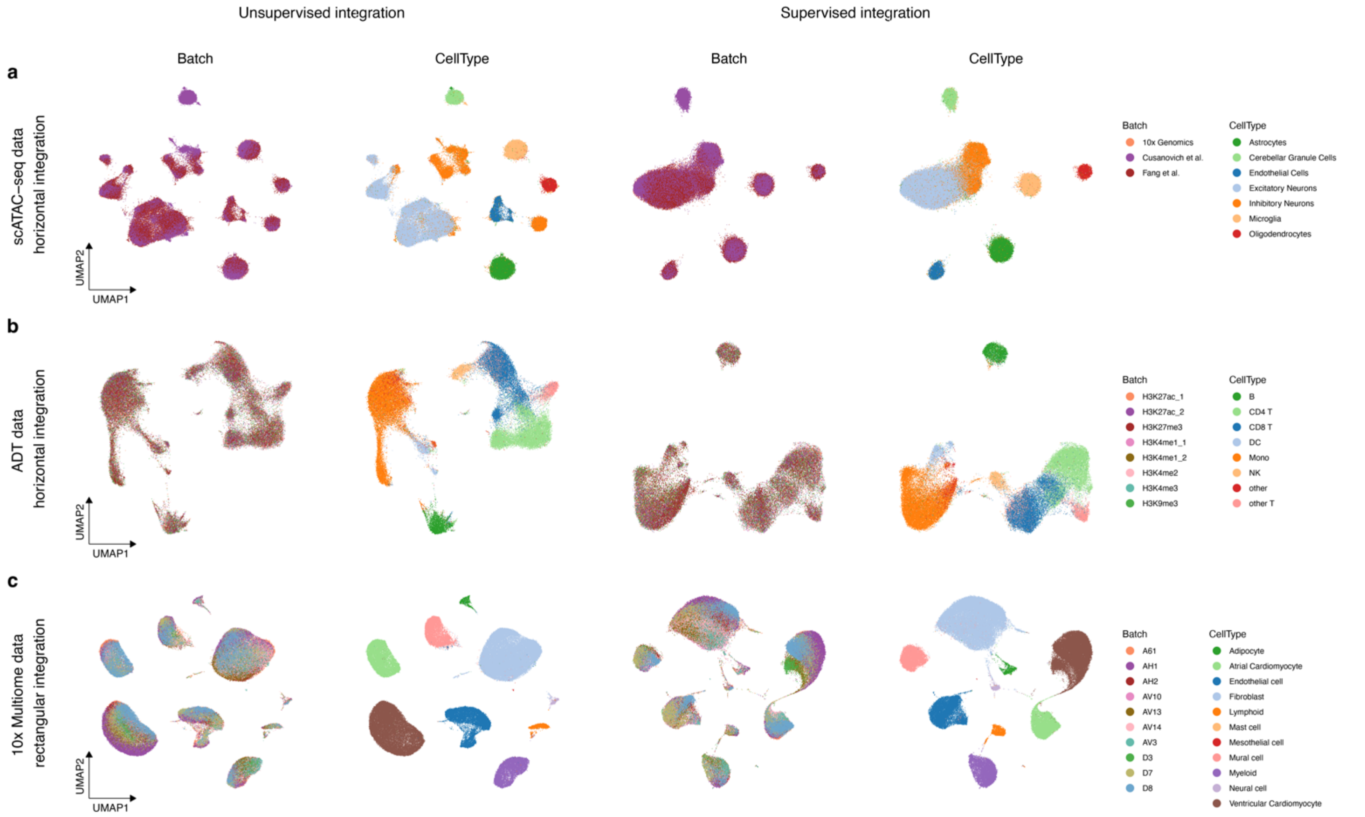

Supplementary Figure 40. Palette can be extended to horizontal and rectangular integration tasks. **a–c.** UMAP visualizations of integrated cell embeddings generated by Palette in unsupervised (left two columns) and supervised (right two columns) modes for mouse brain scATAC-seq data (**a**), human PBMC protein data (**b**), and human heart data (**c**). For each integration mode, cells are colored by batch (first and third columns) and cell type (second and fourth columns), respectively.

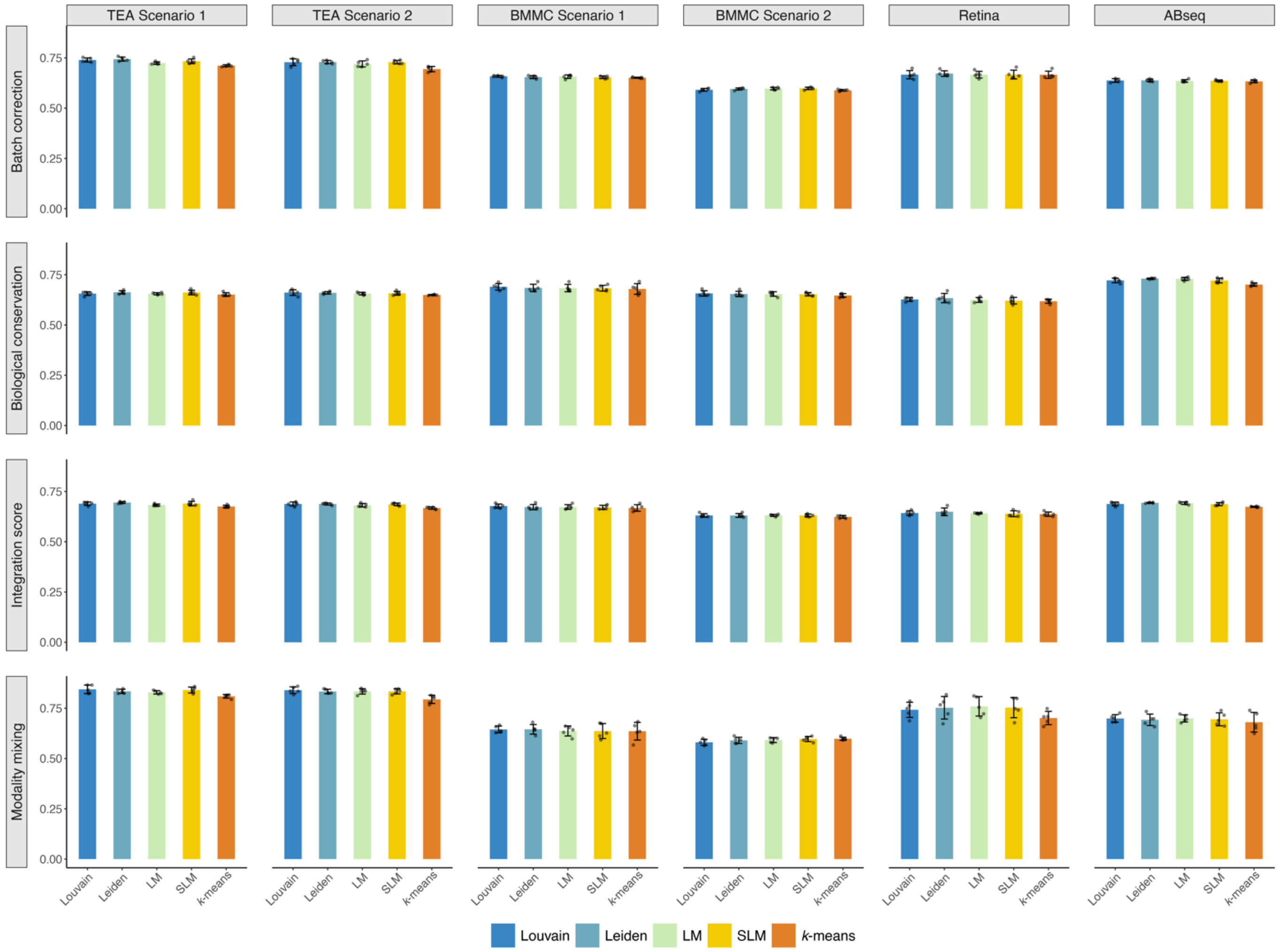

Supplementary Figure 41. Benchmarking clustering algorithms within the unsupervised Palette framework. We evaluated integration performance using multiple quantitative metrics across different clustering strategies combined with Palette: Louvain (default in Palette), Leiden, Louvain with multilevel refinement (LM), smart local moving (SLM), and *k*-means. For Louvain, Leiden, LM, and SLM, the resolution parameter was fixed at 1, consistent with the default setting in Palette. For *k*-means, we specified the number of clusters to match the number of known cell types in each batch. Error bars represent the standard error across evaluated tasks, from $n = 5$ randomly generated sub-experiments.

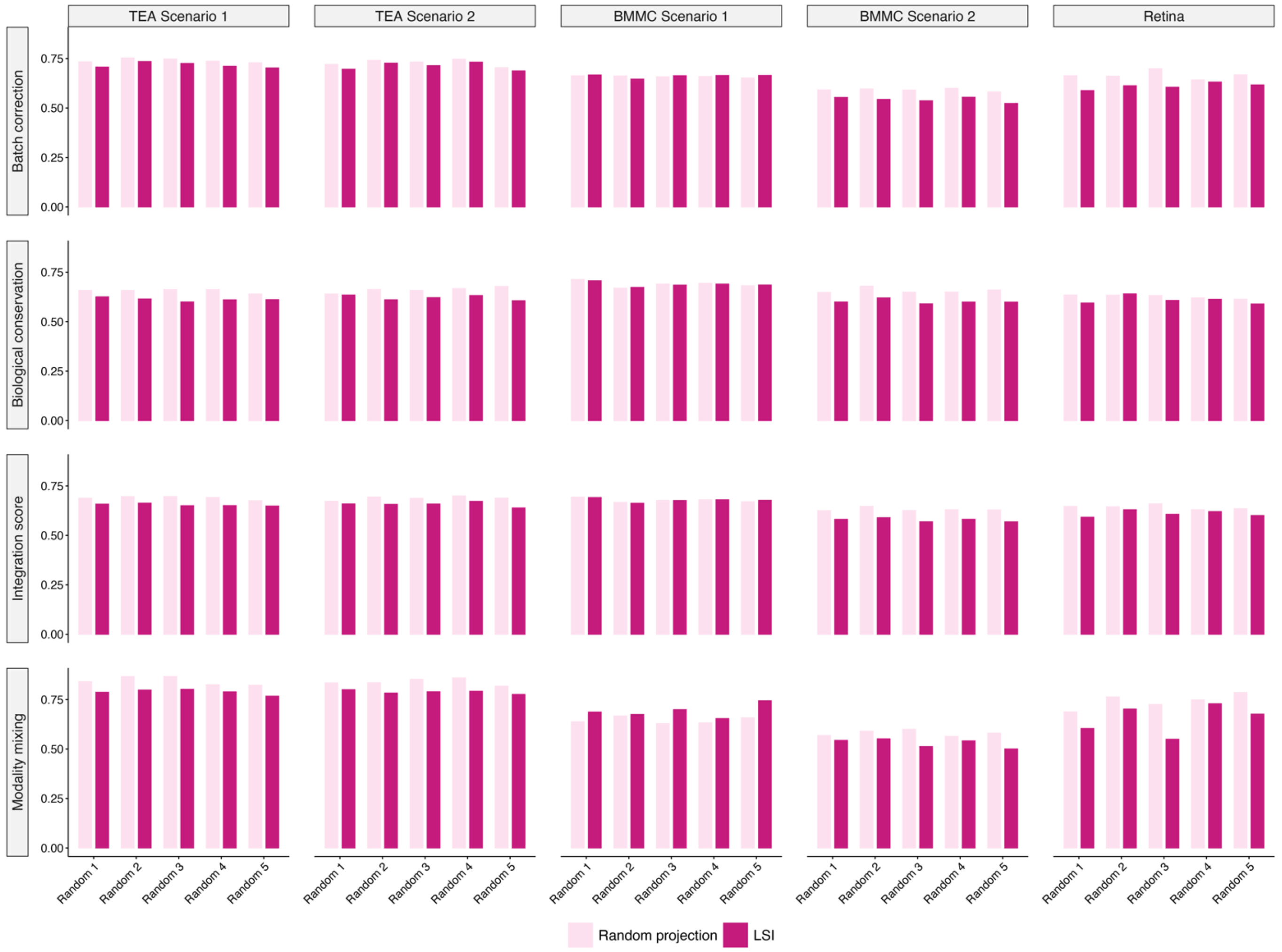

Supplementary Figure 42. Evaluation of Palette's integration performance using different pre-dimensionality reduction methods for chromatin accessibility data. Integration results based on two pre-dimensionality reduction strategies, random projection and LSI, were compared across five integration scenarios using multiple metrics.

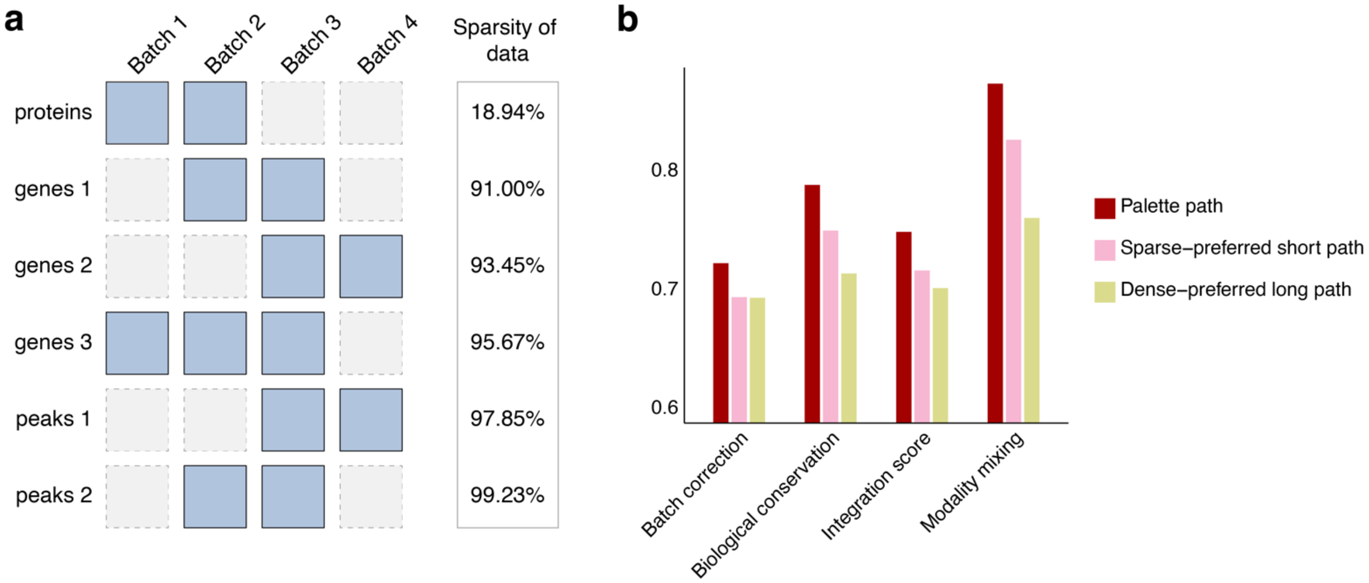

Supplementary Figure 43. Evaluating the trade-off between path length and intermediate-modality sparsity in integration. a. Schematic of the modality composition in the simulated data based on human PBMC TEA-seq dataset. The sparsity levels of each simulated modality are shown on the right. **b.** Quantitative comparison of different path selection strategies, with performance reported separately across four evaluation metrics: batch correction, biological conservation, overall integration, and modality mixing.

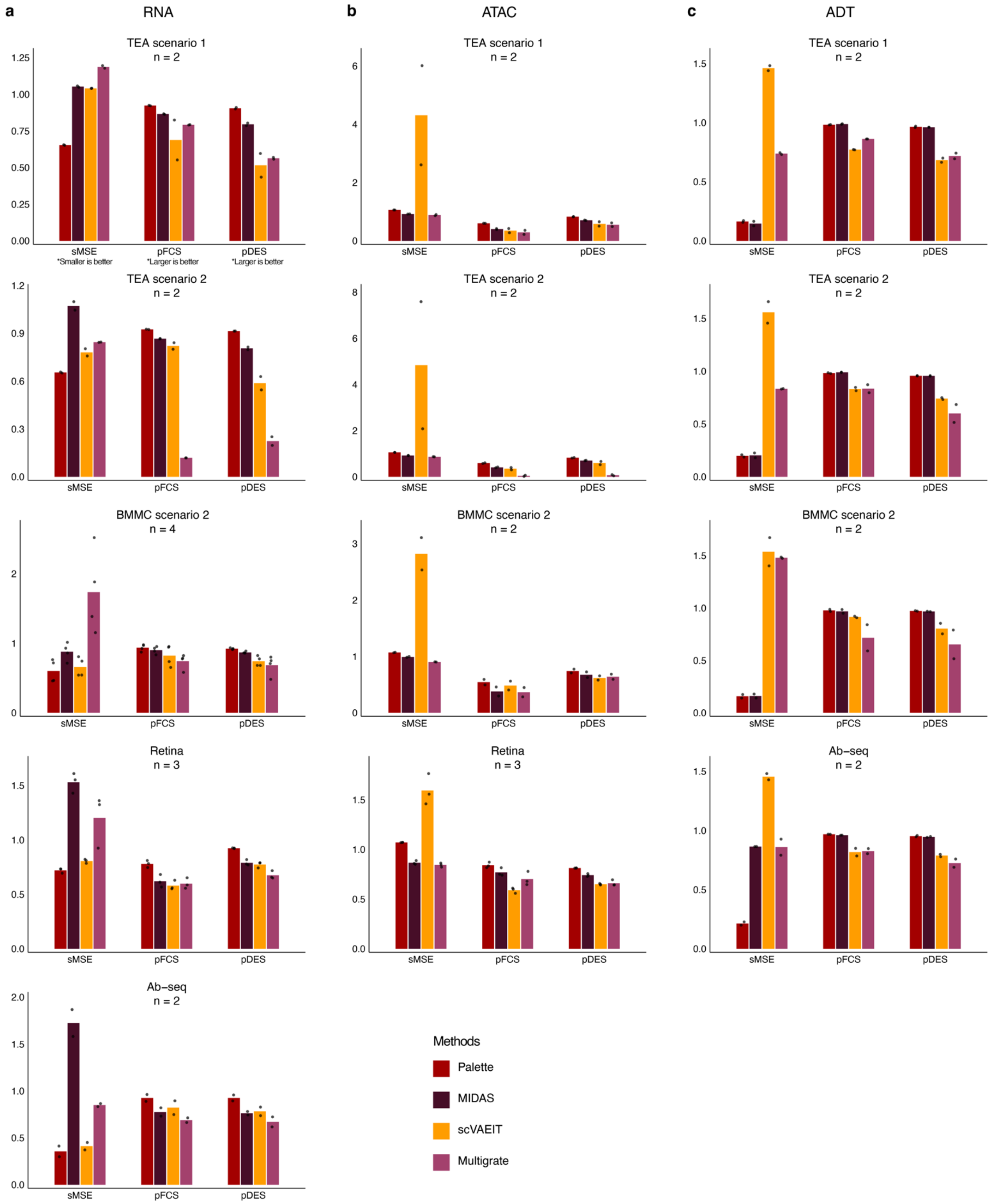

Supplementary Figure 44. Quantitative evaluation of modality inference using data structure metrics.

We compared the modality inference performance of Palette with MIDAS, scVAEIT, and Multigrate across five integration scenarios, using data structure metrics including sMSE, pFCS, and pDES. Performance is

shown for RNA (**a**), ATAC (**b**), and ADT (**c**) in each scenario. For each task and metric, bars represent the mean across inferred batches, and points indicate batch-level measurements. Numbers in each panel denote the number of inferred batches for the corresponding modality and task.

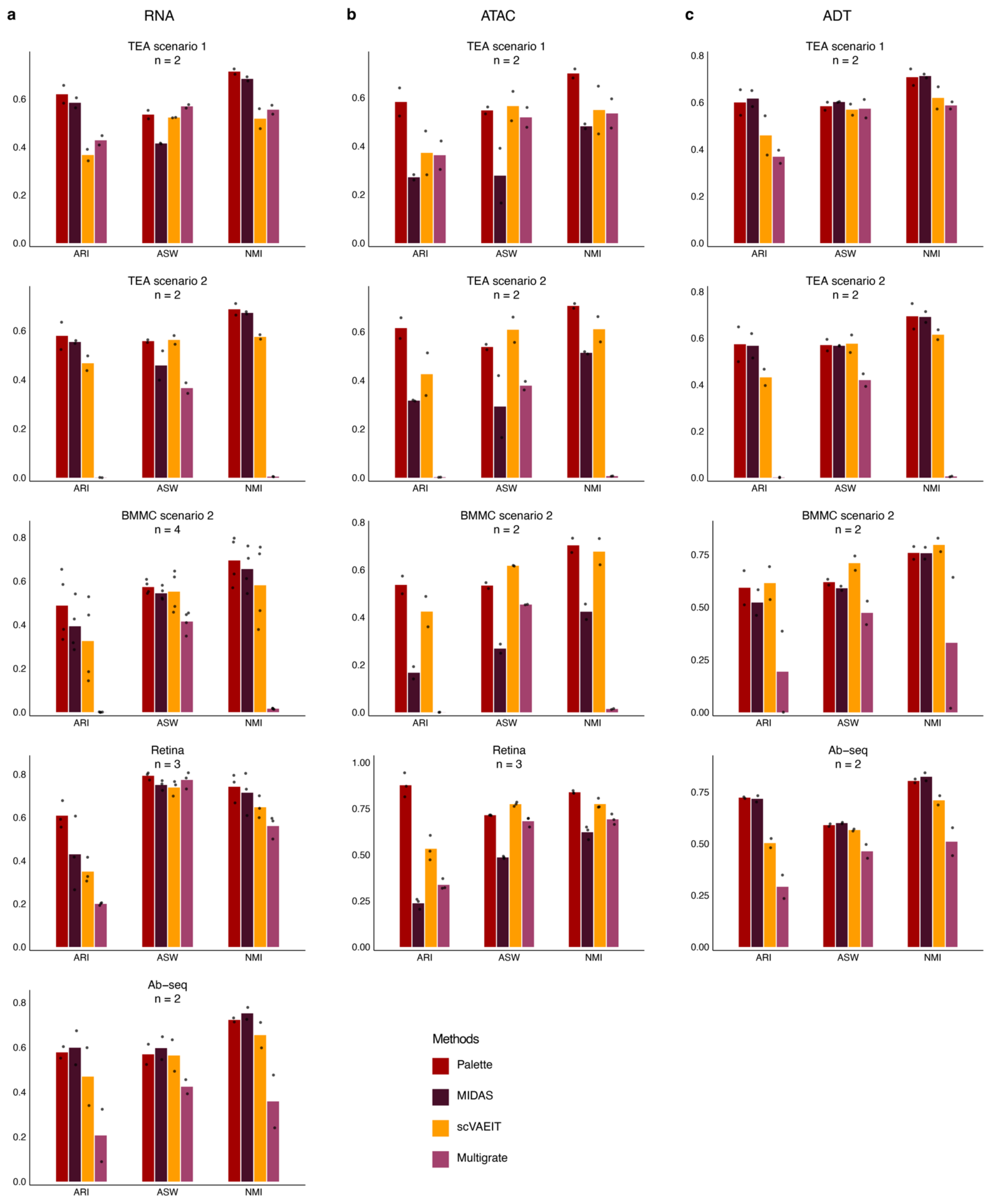

Supplementary Figure 45. Quantitative evaluation of modality inference using clustering-based metrics. We compared the modality inference performance of Palette with MIDAS, scVAEIT, and Multigrate across five integration scenarios, clustering-based metrics including ARI, ASW, and NMI.

Performance is shown for RNA (**a**), ATAC (**b**), and ADT (**c**) in each scenario. For each task and metric, bars represent the mean across inferred batches, and points indicate batch-level measurements. Numbers in each panel denote the number of inferred batches for the corresponding modality and task.

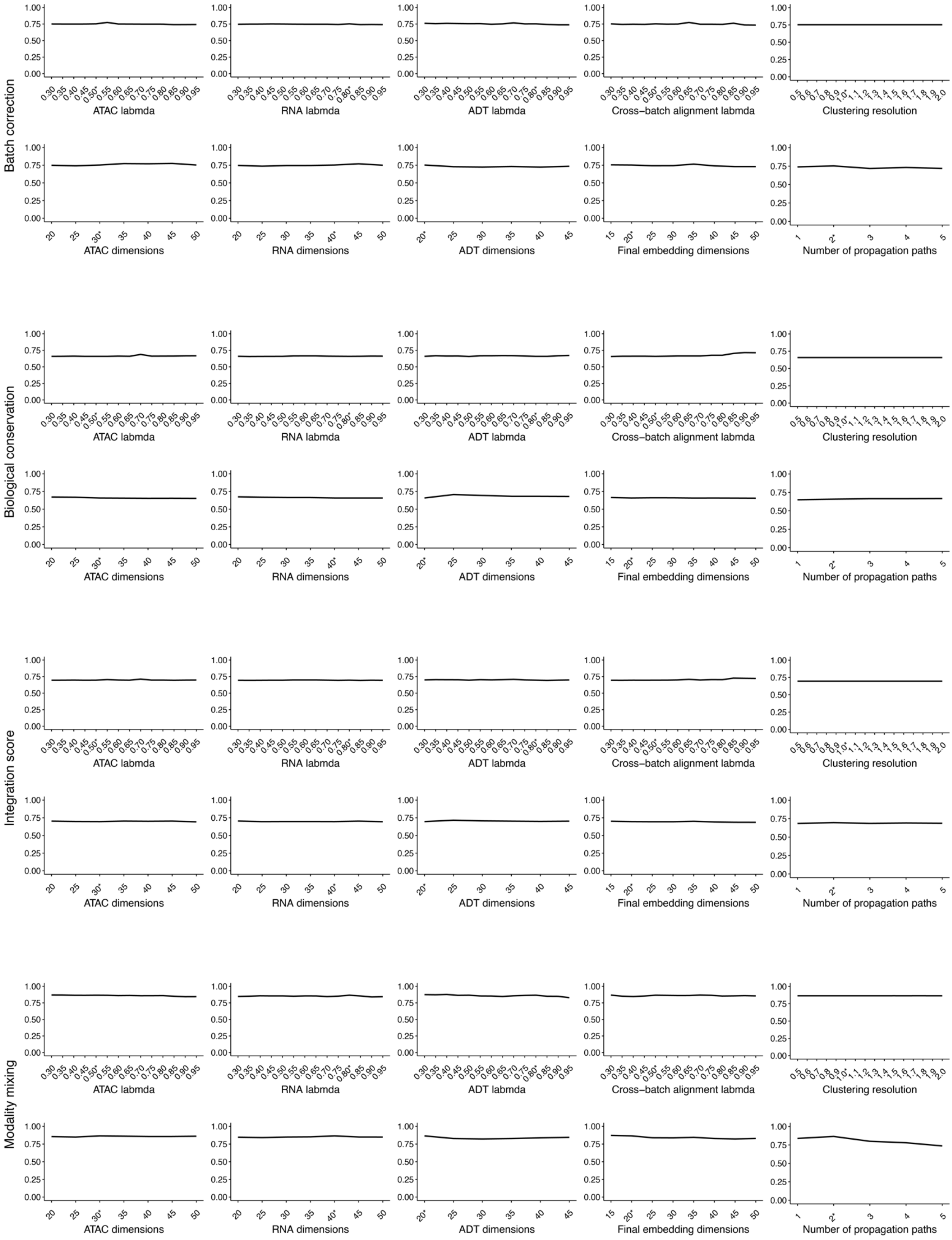

Batch correction
Biological conservation
Integration score
Modality mixing
ATAC labmda
RNA labmda
ADT labmda
Cross−batch alignment labmda
Clustering resolution
ATAC dimensions
RNA dimensions
ADT dimensions
Final embedding dimensions
Number of propagation paths

Supplementary Figure 46. Robustness benchmarking of Palette integration with respect to tuning parameters, using the dataset of the third sub-experiment under the TEA scenario 1. Integration performance was evaluated based on batch correction scores, biological conservation scores, overall integration scores, and modality mixing scores. These metrics were assessed across a range of tuning parameters, including: the Bi-sPCA regularization parameter (λ) used for different modalities and integration stages; clustering resolution for kernel matrix construction in unsupervised mode; dimensionality settings for intra-modal joint reduction and final embedding; and the number of propagation paths. For protein-specific intra-modal dimensionality reduction, the dimension was set to 20 for the human PBMC TEA-seq dataset (which contains 46 ADT features) to ensure sufficient representation across all sub-experiments based on this dataset. In addition, asterisks (*) denote the default parameter settings used by Palette.

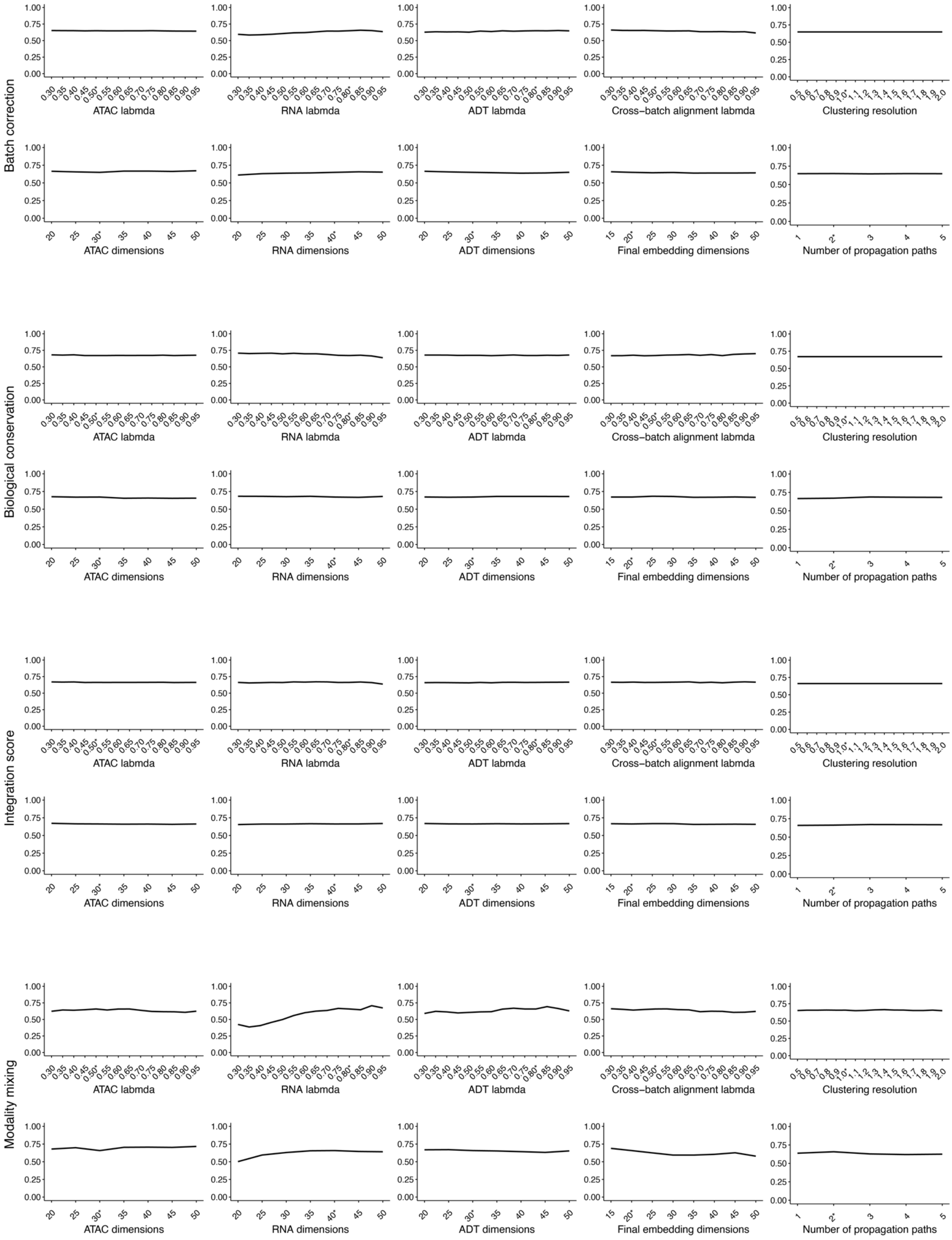
Batch correction
ATAC labmda
RNA labmda
ADT labmda
Cross−batch alignment labmda
Clustering resolution
ATAC dimensions
RNA dimensions
ADT dimensions
Final embedding dimensions
Number of propagation paths
Biological conservation
ATAC labmda
RNA labmda
ADT labmda
Cross−batch alignment labmda
Clustering resolution
ATAC dimensions
RNA dimensions
ADT dimensions
Final embedding dimensions
Number of propagation paths
Integration score
ATAC labmda
RNA labmda
ADT labmda
Cross−batch alignment labmda
Clustering resolution
ATAC dimensions
RNA dimensions
ADT dimensions
Final embedding dimensions
Number of propagation paths
Modality mixing
ATAC labmda
RNA labmda
ADT labmda
Cross−batch alignment labmda
Clustering resolution
ATAC dimensions
RNA dimensions
ADT dimensions
Final embedding dimensions
Number of propagation paths

Supplementary Figure 47. Robustness benchmarking of Palette integration with respect to tuning parameters on the human BMMC CITE-seq and 10x Multiome dataset. Integration performance was evaluated based on batch correction scores, biological conservation scores, overall integration scores, and modality mixing scores. These metrics were assessed across a range of tuning parameters, including: the Bi-sPCA regularization parameter (λ) used for different modalities and integration stages; clustering resolution for kernel matrix construction in unsupervised mode; dimensionality settings for intra-modal joint reduction and final embedding; and the number of propagation paths. An asterisk (*) indicates the default parameter setting used by Palette.

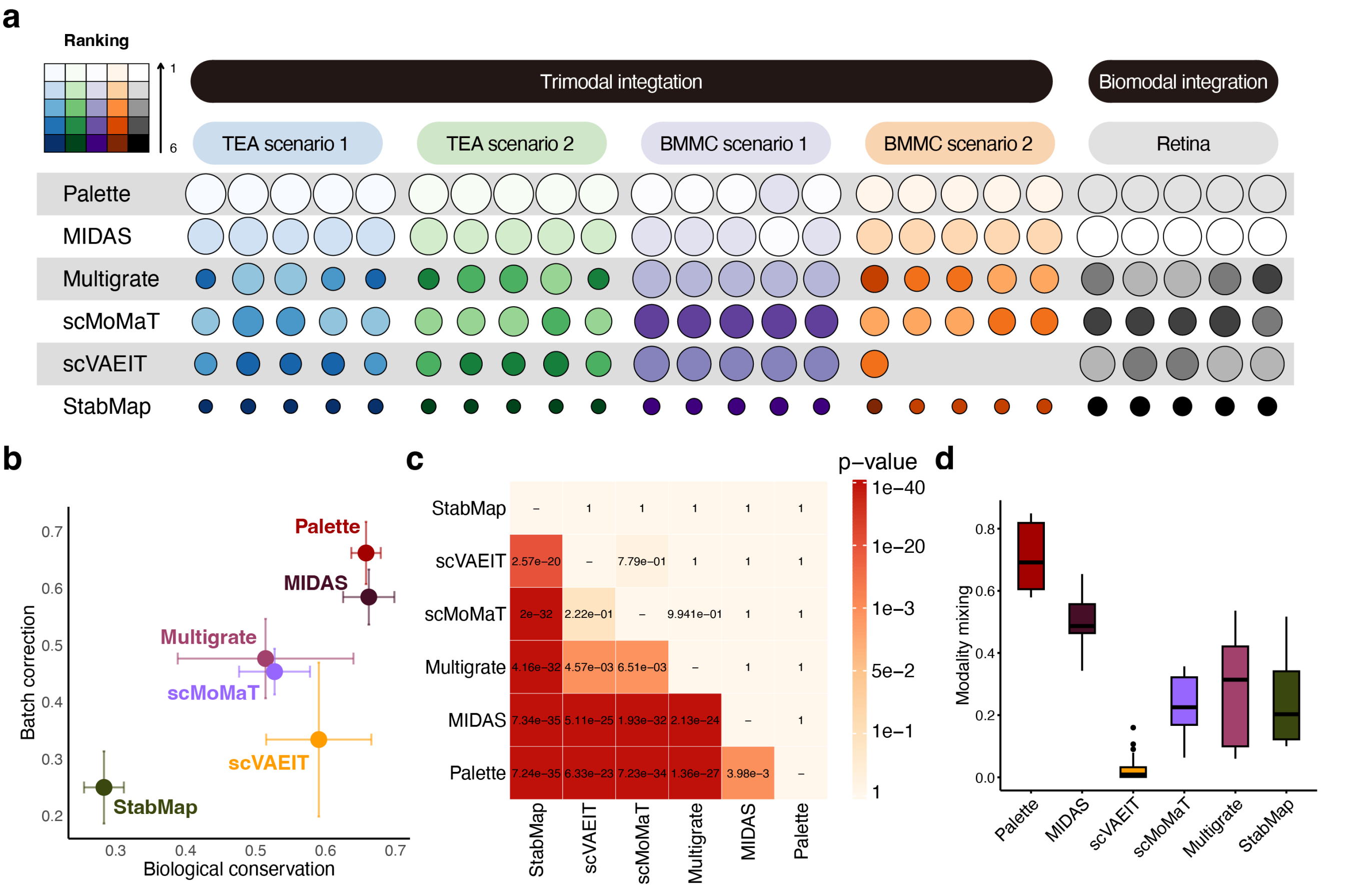

Supplementary Figure 48. Systematic benchmarking of Palette under limited feature size settings. a. Integration ranking across methods for each sub-experiment. The radius of each circle indicates the overall integration scores, ranging from 0 to 1. **b.** Scatter plot of mean biological conservation scores versus mean batch-correction scores for all integration methods. Error bars represent the standard error across the tasks evaluated for each method. **c.** Heatmap of one-sided paired Wilcoxon signed-rank test p-values. **d**. Box plot of overall modality mixing scores across methods. The central lines mark the median values, the boxes show the quartiles, and the whiskers show the rest of the distribution. In this benchmark, Palette was configured to use the minimum number of features required by any method within each scenario. In addition, scVAEIT failed to complete four sub-experiments in the BMMC scenario 2 due to training stagnation. For this method, means, medians, standard errors, and p-values were calculated based on the remaining 21 completed sub-experiments.

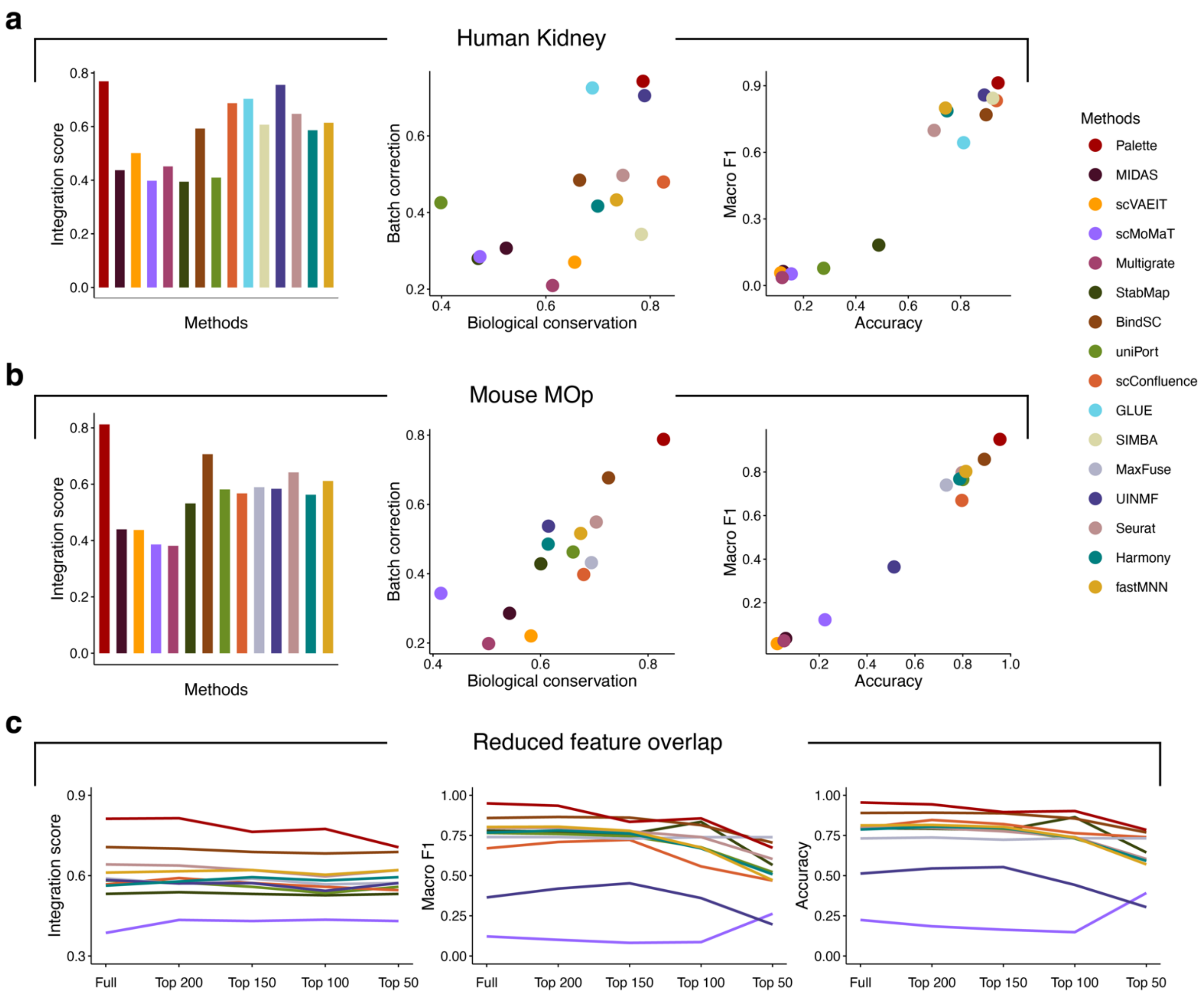

Supplementary Figure 49. Diagonal integration results of Palette under limited feature size settings. **a, b.** Comparison of integration and label transfer performance on human kidney (**a**) and mouse MOp (**b**) datasets across multiple evaluation metrics. **c.** Line plots comparing integration and label transfer performance for different integration methods under reduced feature overlap. In all experiments shown in this figure, Palette was configured to use the minimum number of features required by any method within each scenario.

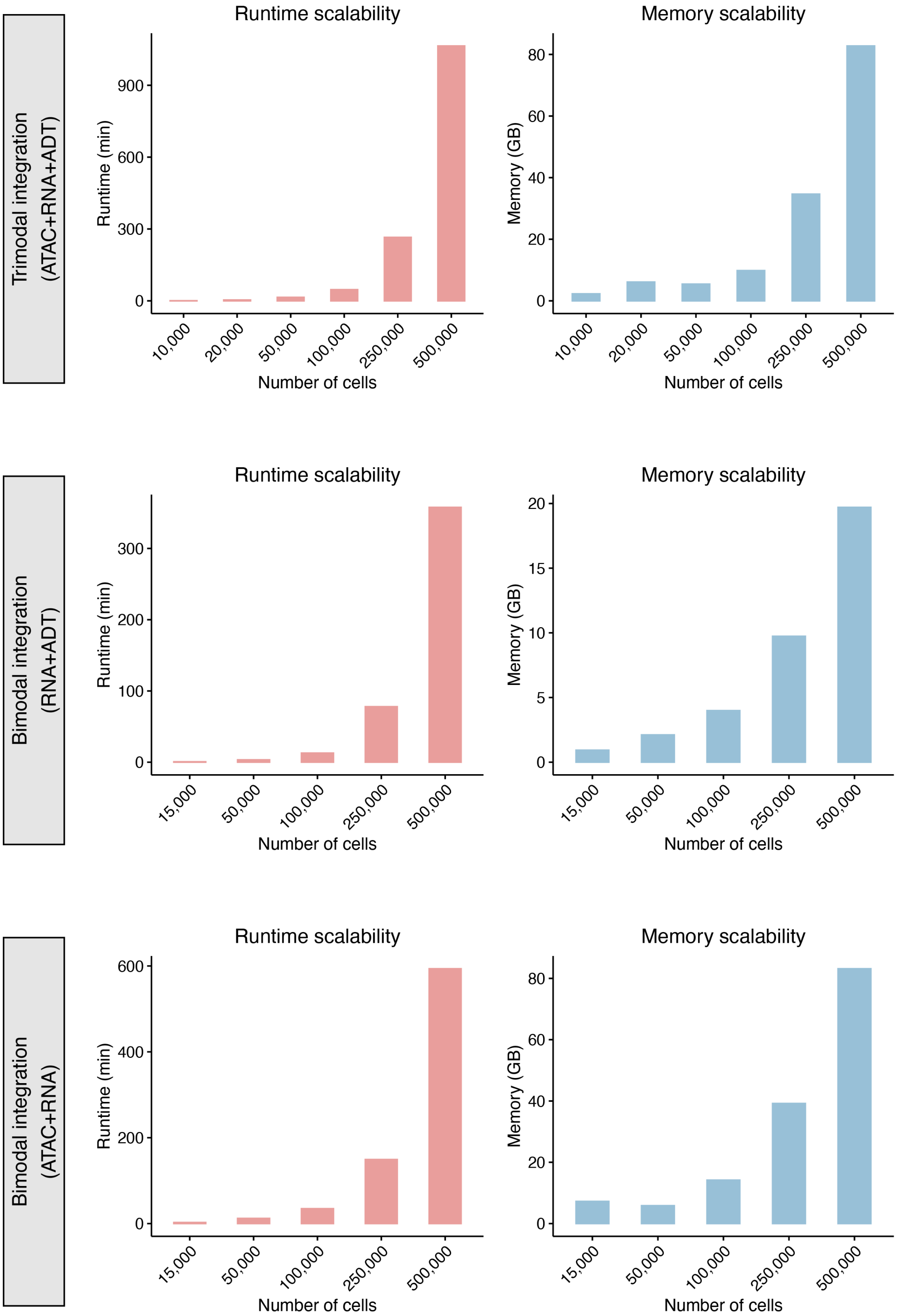

Supplementary Figure 50. Computational scalability of Palette integration. Runtime (left column) and peak RAM (right column) used for Palette integration under increasing cell numbers. Results are shown for trimodal integration (top row) and for two bimodal settings: RNA + ADT (middle row) and ATAC + RNA (bottom row).

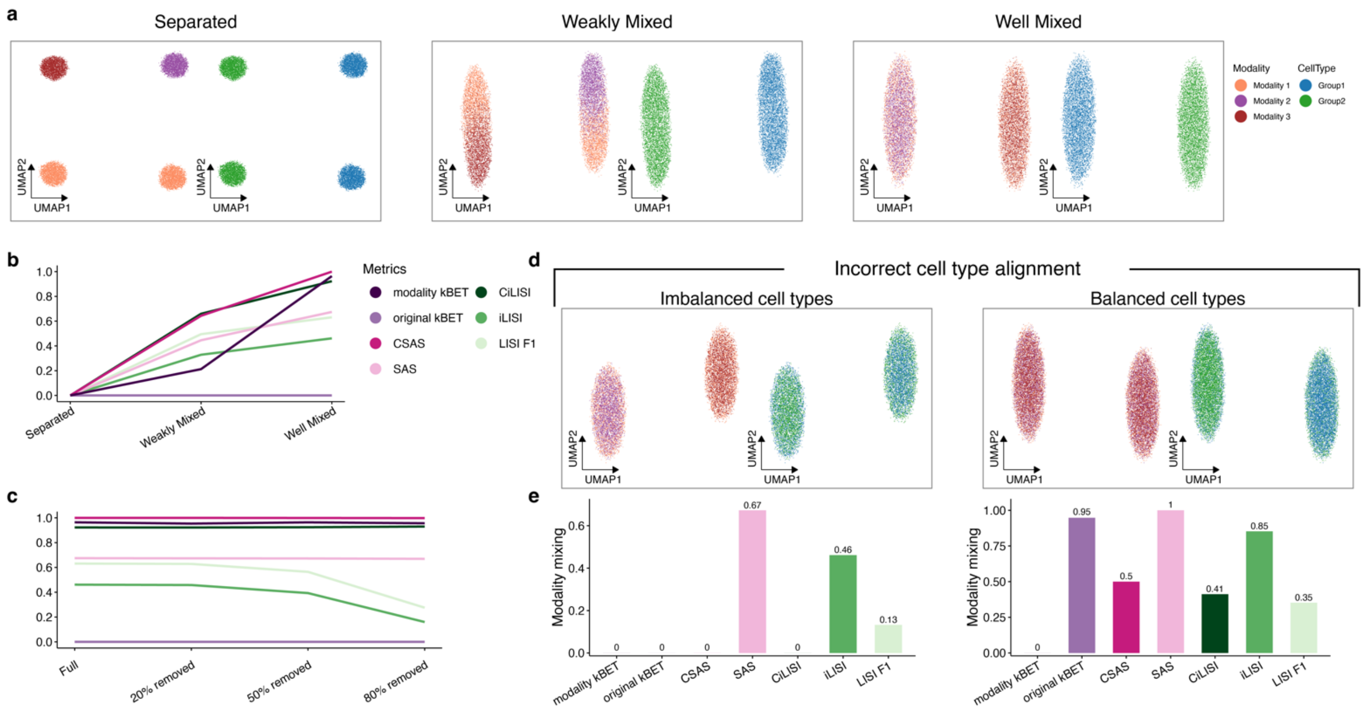

Supplementary Figure 51. Quantitative evaluation of modality mixing metrics across simulated scenarios. a. UMAP visualizations of cell embeddings under three simulated scenarios representing increasing levels of modality mixing. Cells are colored by modality composition (first, third, and fifth columns) and by cell type (second, fourth, and sixth columns). **b.** Quantitative results of seven evaluation metrics under increasing levels of modality mixing. **c.** Line plots showing metric changes under increasing levels of imbalance in sample sizes among different modality compositions. **d.** UMAP visualizations showing embeddings under incorrect cross-modality cell type alignment for datasets with imbalanced (left) and balanced (right) cell type compositions. Cells are colored by modality composition (first and third columns) and by cell type (second and fourth columns). **e.** Bar plots showing metrics performance under incorrect cross-modality cell type alignment for both imbalanced (left) and balanced (right) scenarios.

Supplementary References

1. Li, B. et al. HCA Data Portal: census of immune cells (Human Cell Atlas, 2019).
2. Haghverdi, L., Lun, A. T. L., Morgan, M. D. & Marioni, J. C. Batch effects in single-cell RNA-sequencing data are corrected by matching mutual nearest neighbors. *Nat. Biotechnol.* **36**, 421–427 (2018).
3. Stuart, T. et al. Comprehensive Integration of Single-Cell Data. *Cell* **177**, 1888–1902 (2019).
4. Korsunsky, I. et al. Fast, sensitive and accurate integration of single-cell data with Harmony. *Nat. Methods* **16**, 1289–1296 (2019).
5. Andreatta, M. & Carmona, S. J. STACAS: Sub-type anchor correction for alignment in Seurat to integrate single-cell RNA-seq data. *Bioinformatics* **37**, 882–884 (2021).
6. Xu, C. et al. Probabilistic harmonization and annotation of single-cell transcriptomics data with deep generative models. *Mol. Syst. Biol.* **17**, e9620 (2021).
7. De Donno, C. et al. Population-level integration of single-cell datasets enables multi-scale analysis across samples. *Nat Methods* **20**, 1683–1692 (2023).
8. Zhou, Y., Sheng, Q. & Jin, S. Integrating single-cell data with biological variables. *Proc. Natl Acad. Sci. USA* **122**, e2416516122 (2025).
9. Andreatta, M. et al. Semi-supervised integration of single-cell transcriptomics data. *Nat. Commun.* **15**, 872 (2024).
10. Suresh, H. et al. Comparative single-cell transcriptomic analysis of primate brains highlights human-specific regulatory evolution. *Nat. Ecol. Evol.* **7**, 1930–1943 (2023).
11. Luecken, M. D. et al. Benchmarking atlas-level data integration in single-cell genomics. *Nat. Methods* **19**, 41–50 (2022).
12. Liu, C., Ding, S., Kim, H.J. et al. Multitask benchmarking of single-cell multimodal omics integration methods. *Nat Methods* **22**, 2449–2460 (2025).
