# Supplementary material for: Robust integration of single-cell datasets with imbalanced modality composition
Source: Nat Commun. 2026 May 14;17:6423. doi: 10.1038/s41467-026-72933-4 (PMC13376370; doi:10.1038/s41467-026-72933-4)
Supplement: Supplementary file 3 — Description of Additional Supplementary Files [file 41467_2026_72933_MOESM3_ESM.pdf]

Description of Additional Supplementary Files:

Supplementary Data 1: Public datasets used in this study.

Supplementary Data 2: Benchmark data construction details.

Supplementary Data 3: Cell types removed in each simulation batch.

Supplementary Data 4: Query data construction details.
