# Supplementary material for: Robust integration of single-cell datasets with imbalanced modality composition
Source: Nat Commun. 2026 May 14;17:6423. doi: 10.1038/s41467-026-72933-4 (PMC13376370; doi:10.1038/s41467-026-72933-4)
Supplement: Supplementary file 8 — Reporting Summary [file 41467_2026_72933_MOESM8_ESM.pdf]

Corresponding author(s): Shuilin Jin

Last updated by author(s): Mar 19, 2026

Reporting Summary

Nature Portfolio wishes to improve the reproducibility of the work that we publish. This form provides structure for consistency and transparency in reporting. For further information on Nature Portfolio policies, see our Editorial Policies and the Editorial Policy Checklist.

Statistics

For all statistical analyses, confirm that the following items are present in the figure legend, table legend, main text, or Methods section.

n/a	Confirmed	
☐	☒	The exact sample size (*n*) for each experimental group/condition, given as a discrete number and unit of measurement
☐	☒	A statement on whether measurements were taken from distinct samples or whether the same sample was measured repeatedly
☐	☒	The statistical test(s) used AND whether they are one- or two-sided<br>*Only common tests should be described solely by name; describe more complex techniques in the Methods section.*
☒	☐	A description of all covariates tested
☐	☒	A description of any assumptions or corrections, such as tests of normality and adjustment for multiple comparisons
☐	☒	A full description of the statistical parameters including central tendency (e.g. means) or other basic estimates (e.g. regression coefficient) AND variation (e.g. standard deviation) or associated estimates of uncertainty (e.g. confidence intervals)
☐	☒	For null hypothesis testing, the test statistic (e.g. *F*, *t*, *r*) with confidence intervals, effect sizes, degrees of freedom and *P* value noted<br>*Give P values as exact values whenever suitable.*
☒	☐	For Bayesian analysis, information on the choice of priors and Markov chain Monte Carlo settings
☒	☐	For hierarchical and complex designs, identification of the appropriate level for tests and full reporting of outcomes
☐	☒	Estimates of effect sizes (e.g. Cohen's *d*, Pearson's *r*), indicating how they were calculated

Our web collection on statistics for biologists contains articles on many of the points above.

Software and code

Policy information about availability of computer code

Data collection	All data used in this manuscript are publicly available and no software was used for data collection.
Data analysis	The code used in data analysis in this study is deposited to Zenodo https://doi.org/10.5281/zenodo.18743288<br><br>Python packages used:<br>COMMOT (v0.0.3)<br>MIDAS (https://github.com/labomics/midas/tree/reproducibility/)<br>multigrate (v0.0.2)<br>scmomat (v0.2.2)<br>scarches (v0.5.9)<br>scVAEIT (v1.0.2)<br>scvi (v1.0.4)<br>uniport (v1.3)<br>scconfluence (v0.1.1)<br>scglue (v0.4.0)<br>simba (v1.2)<br>maxfuse (v0.0.2)<br><br>R packages used:

batchelor (v1.14.1)
bindSC (v1.0.0)
clusterProfiler (v4.6.2)
harmony (v1.2.0)
randomForest (v4.7.1.2)
rliger (v2.0.1)
Seurat (v4.4.0)
Signac (v1.9.0)
SIGNAL (v1.0.0)
StabMap (v0.1.8)
stats (v4.2.3)
SuperExactTest (v1.1.0)
caret (v6.0.94)
STACAS (v2.3.0)
limma (v3.54.2)
peakRAM (v1.0.2)

For manuscripts utilizing custom algorithms or software that are central to the research but not yet described in published literature, software must be made available to editors and reviewers. We strongly encourage code deposition in a community repository (e.g. GitHub). See the Nature Portfolio guidelines for submitting code & software for further information.

Data

Policy information about availability of data

All manuscripts must include a data availability statement. This statement should provide the following information, where applicable:

- Accession codes, unique identifiers, or web links for publicly available datasets
- A description of any restrictions on data availability
- For clinical datasets or third party data, please ensure that the statement adheres to our policy

All datasets used in this manuscript are publicly available.
The human PBMC TEA-seq dataset is available in the Gene Expression Omnibus (GEO) database under accession code GSE158013 https://www.ncbi.nlm.nih.gov/geo/query/acc.cgi?acc=GSE158013.
The human BMMC CITE-seq and 10x Multiome datasets we used to construct benchmark data, reference, and query are available in the GEO database under accession code GSE194122 https://www.ncbi.nlm.nih.gov/geo/query/acc.cgi?acc=GSE194122.
The human retina 10x Multiome dataset is available in the GEO database under accession code GSE196235 https://www.ncbi.nlm.nih.gov/geo/query/acc.cgi?acc=GSE196235.
The human BMMC Ab-seq dataset is available at https://figshare.com/projects/Single-cell_proteogenomic_reference_maps_of_the_human_hematopoietic_system/94469.
The HCA BMMC scRNA-seq reference dataset is available at https://explore.data.humancellatlas.org/projects/cc95ff89-2e68-4a08-a234-480eca21ce79.
The cross-condition human PBMC dataset is available in the GEO database under accession code GSE156473 https://www.ncbi.nlm.nih.gov/geo/query/acc.cgi?acc=GSE156473.
Five batches of the cross-species MOp dataset (Human_10xV3, Human_SNARE, Mouse_10xV3, Marmoset_10xV3, and Marmoset_SNARE) are available at https://assets.nemoarchive.org/dat-ek5dbmu.
Two batches of the cross-species MOp dataset (Human_10xMultiome_ATAC and Mouse_10xMultiome) are available in the GEO database under accession code GSE229169 https://www.ncbi.nlm.nih.gov/geo/query/acc.cgi?acc=GSE229169.
One batch of the cross-species MOp dataset (Mouse_10xATAC) is available at https://www.10xgenomics.com/cn/datasets/fresh-cortex-from-adult-mouse-brain-p-50-1-standard-1-1-0.
The human cortex Slide-tags dataset is available at https://singlecell.broadinstitute.org/single_cell/study/SCP2169/slide-tags-snrna-seq-on-human-tonsil.
The cross-species WAT scRNA-seq dataset can be accessed via the CELLxGENE portal at https://cellxgene.cziscience.com/collections/fe0e718d-2ee9-42cc-894b-0b490f437dfd.
The human tonsil 10x Visium dataset is available at https://doi.org/10.5281/zenodo.12654113.
The human kidney scRNA-seq and scATAC-seq datasets can be accessed at https://datasets.cellxgene.cziscience.com/0da9127b-6faf-458c-a124-d04315f4db6e.h5ad and https://datasets.cellxgene.cziscience.com/2d8349bd-3045-4b9a-bbad-301ea3190fef.h5ad, respectively.
The human PBMC scRNA-seq dataset is available in the GEO database under accession code GSE164378 https://www.ncbi.nlm.nih.gov/geo/query/acc.cgi?acc=GSE164378.
The human PBMC CyTOF dataset is available at http://flowrepository.org/id/FR-FCM-Z249.
The human tonsil scRNA-seq dataset is available in the GEO database under accession code GSE165860 https://www.ncbi.nlm.nih.gov/geo/query/acc.cgi?acc=GSE165860.
The human tonsil CODEX dataset is available at https://onlinelibrary.wiley.com/doi/10.1002/eji.202048891.
The mouse MOp MERFISH dataset is available at https://doi.brainimagelibrary.org/doi/10.35077/g.21.
The mouse MOp scATAC-seq dataset is available at https://catlas.org/mousebrain/#!/.
The human MTG dataset can be accessed via the Allen Brain Map portal at https://portal.brain-map.org/atlases-and-data/rnaseq.
The human immune scRNA-seq, the human pancreas scRNA-seq and the mouse brain scATAC-seq dataset can be accessed at https://doi.org/10.6084/m9.figshare.12420968.
The human PBMC ADT dataset is available at https://doi.org/10.5281/zenodo.5504061.
The human heart 10x Multiome dataset is available at https://www.heartcellatlas.org.
More details of the datasets are provided in Supplementary Data 1.
Processed datasets used for the analyses have been deposited in Zenodo at https://doi.org/10.5281/zenodo.18045027.
Source Data are provided with this paper.

Research involving human participants, their data, or biological material

Policy information about studies with human participants or human data. See also policy information about sex, gender (identity/presentation), and sexual orientation and race, ethnicity and racism.

Reporting on sex and gender	NA
Reporting on race, ethnicity, or other socially relevant groupings	NA
Population characteristics	NA
Recruitment	NA
Ethics oversight	NA

Note that full information on the approval of the study protocol must also be provided in the manuscript.

Field-specific reporting

Please select the one below that is the best fit for your research. If you are not sure, read the appropriate sections before making your selection.

[x] Life sciences [ ] Behavioural & social sciences [ ] Ecological, evolutionary & environmental sciences

For a reference copy of the document with all sections, see nature.com/documents/nr-reporting-summary-flat.pdf

Life sciences study design

All studies must disclose on these points even when the disclosure is negative.

Sample size	No sample size calculation was performed. For data used in the paper, sample sizes were chosen based on the availability of public data resources and all available data were included for analysis.
Data exclusions	No data was excluded.
Replication	We provide the code necessary for replicating the results. Different package versions or computational environments might lead to slightly different outputs.
Randomization	For the cross-condition human PMBC mosaic integration analysis, we randomly removed the same number of features as DEFs identified by Palette from each modality.
Blinding	All datasets used here are published in previous research, thus, it is not possible to blind the investigations in reanalyzing the data.

Reporting for specific materials, systems and methods

We require information from authors about some types of materials, experimental systems and methods used in many studies. Here, indicate whether each material, system or method listed is relevant to your study. If you are not sure if a list item applies to your research, read the appropriate section before selecting a response.

Materials & experimental systems

n/a	Involved in the study	
[x]	[ ]	Antibodies
[x]	[ ]	Eukaryotic cell lines
[x]	[ ]	Palaeontology and archaeology
[x]	[ ]	Animals and other organisms
[x]	[ ]	Clinical data
[x]	[ ]	Dual use research of concern
[x]	[ ]	Plants

Methods

n/a	Involved in the study	
[x]	[ ]	ChIP-seq
[x]	[ ]	Flow cytometry
[x]	[ ]	MRI-based neuroimaging

Plants

Seed stocks	NA
Novel plant genotypes	NA
Authentication	NA
