# Supplementary material for: Robust integration of single-cell datasets with imbalanced modality composition
Source: Nat Commun. 2026 May 14;17:6423. doi: 10.1038/s41467-026-72933-4 (PMC13376370; doi:10.1038/s41467-026-72933-4)
Supplement: Supplementary file 2 — Peer Review File [file 41467_2026_72933_MOESM2_ESM.pdf]

nature portfolio

Peer Review File

Robust integration of single-cell datasets with imbalanced modality composition

Corresponding Author: Professor Shuilin Jin

This file contains all reviewer reports in order by version, followed by all author rebuttals in order by version.

Version 0:

Reviewer comments:

Reviewer #1

(Remarks to the Author)
The study presents Palette, a novel computational framework designed to address a critical and timely challenge in single-cell genomics: the mosaic integration of multimodal datasets with imbalanced modality compositions. The authors have invested substantial effort in developing this method and have demonstrated its potential across a wide range of complex scenarios. However, before this manuscript can be considered for publication, several fundamental concerns regarding the experimental design, methodological novelty, evaluation, and comparison with existing techniques must be addressed. The current manuscript fails to provide sufficiently compelling evidence to support its claims and requires substantial additional experimentation and deeper analysis to strengthen its scientific rigor.

1. The central claim of the study is its ability to handle "imbalanced modality composition", yet this is not adequately reflected in the experimental design. In my view, an imbalance in the proportion of batches with different modality compositions (e.g., a 9:1 ratio of complete-modality batches to missing-modality batches) is not the primary challenge; integration may even be easier in such cases due to the abundance of complete information. A more challenging and realistic scenario involves severe imbalances in cell or batch counts associated with different modalities (e.g., integrating nine 'RNA-only' batches with one 'RNA+ATAC' batch) or extensive modality missingness. The current experimental setup fails to simulate these more difficult scenarios, thus weakening the claims about Palette's robustness in handling this critical issue.

2. The proposed "Modality mixing metrics" are intended to evaluate the mixing of cells with different modality compositions, which the authors use to distinguish between batch effects and modality composition heterogeneity. However, the design of this metric is flawed. Because it measures the mixing between batches that have different modality compositions, rather than the alignment of the modalities themselves within shared cell types, this metric is largely coupled with batch correction metrics (e.g., bASW, iLISI). Consequently, better batch alignment will invariably lead to a higher modality mixing score, making this metric unable to independently and effectively assess the quality of modality alignment.

3. The scope of algorithmic comparisons is insufficient to establish the necessity of Palette. For specific integration scenarios, it is crucial to benchmark against the respective state-of-the-art methods. For instance, the spatial mosaic integration analysis should include comparisons with tools like SpatialGlue (Nat Meth 2024), MISO (Nat Meth 2025), MUSE (Nat Biotechnol 2022), and SIMO (Nat Commun 2025). Similarly, the diagonal integration section should be benchmarked against GLUE (Nat Biotechnol 2022), uniPort (Nat Commun 2022), scConfluence (Nat Commun 2024), MaxFuse (Nat Biotechnol 2023), and SIMBA (Nat Meth 2024). Comparing only against general-purpose mosaic integration methods fails to demonstrate that Palette offers a superior or necessary solution in these specialized domains.

4. The manuscript benchmarks Palette against methods like MIDAS and Multigrate in several scenarios (e.g., cross-species, spatial mosaic integration) for which these competitor tools were not originally designed. The authors must provide a detailed description in the Methods section of how the preprocessing pipelines, input data structures, and even model architectures of these competitor algorithms were modified or extended to handle these complex tasks. Without these critical methodological details, the fairness and validity of the comparisons cannot be assessed, and the reliability of the results is questionable.

5. The claim of Palette's simplicity is undermined by its reliance on several key parameters that require manual tuning,

particularly the modality-specific regularization parameter λ for Bi-sPCA. Although robustness analysis is provided, the manuscript lacks a clear explanation of how the default parameters were chosen and fails to offer practical guidance for users on how to select or tune these parameters for new datasets. This ambiguity limits the tool's ease of use and broad applicability.

6. The manuscript does not systematically evaluate Palette's performance on cross-modal prediction (i.e., modality translation) or data imputation, which are key capabilities of many modern multimodal integration methods. A lack of direct comparison with SOTA methods on these tasks makes it difficult for readers to fully assess the functional scope and relative advantages of Palette.

7. It is unclear whether the method can output a corrected data matrix in the original feature space, analogous to the IntegrateData function in Seurat. Such a feature is crucial for many downstream analyses (e.g., differential expression, trajectory inference) and for ensuring compatibility with other single-cell analytical toolkits. If Palette's integration is confined to the low-dimensional embedding space, this would significantly limit its versatility and utility in broader biological analysis workflows.

8. The manuscript needs to clarify how Palette handles inconsistent features within the same modality across different batches (e.g., missing proteins or genes due to different antibody panels or gene annotations). If the approach relies on a simple feature intersection, the number of shared features could decrease dramatically as more datasets are integrated, leading to significant information loss and potentially compromising integration performance. Does Palette employ a more sophisticated strategy to address this common issue?

9. In the reference-based integration strategy, the method appears to use only a subset of the reference atlas—specifically, batches with modality compositions matching the query—as a 'bridge' (as illustrated in Supplementary Fig. 1). This approach may discard a substantial amount of valuable information present in other batches of the reference, potentially reducing the accuracy and robustness of the alignment. The authors should justify this design choice or compare its performance against methods that leverage the entire reference atlas.

10. The theoretical novelty of the core components, Bi-sPCA and the MBG-guided propagation, needs to be more clearly articulated. The authors should position Bi-sPCA more thoroughly within the context of related dimensionality reduction techniques in machine learning (e.g., variants of CCA, dual-view PCA, or other multi-view learning methods). Similarly, the MBG propagation strategy bears conceptual resemblance to other graph-based methods like StabMap. A more detailed discussion is needed to highlight the unique aspects and advantages of Palette's graph construction and information propagation mechanism compared to existing approaches, thereby clarifying its methodological innovation.

11. The fairness of the benchmarking is a concern. The Methods section states that for some competitor methods (e.g., Multigrate, scMoMaT), a reduced number of features (e.g., top 50,000 peaks for scATAC-seq) was used due to memory constraints. The authors must explicitly clarify whether Palette was also restricted to the exact same feature subset in these comparisons. If Palette utilized more input information than its competitors, the performance gains may be partially attributable to this discrepancy, rendering the comparison unfair.

(Remarks on code availability)
To improve reproducibility, please explicitly mapping each analysis in the paper to its corresponding code and parameter settings in the GitHub repository.

Reviewer #2

(Remarks to the Author)
The paper presents Palette, a computational framework for mosaic integration of single-cell multimodal data. Mosaic integration aims to combine datasets that measure different molecular features — such as RNA, proteins, or chromatin accessibility — often from different cells or experiments, to build a unified representation of the cell population. Palette achieves this by using a modified form of principal component analysis and a mosaic bipartite graph, a network that links cells across different data types based on their similarity. Together, this approach aims to separate technical noise from biological variation, allowing information to be shared across missing modalities.
The benchmark on integration performance conducted on bimodal and trimodal datasets shows that Palette outperforms state-of-the-art methods in both biological signal preservation and batch correction. The study encompasses a wide range of datasets, modalities, and technologies. The authors provide also valuable insights into cross-species integration. The work also contributes a benchmarking resource, comparing across integration scenarios and demonstrating robust handling of batch effects beyond cross-modality alignment.
While we appreciate the value of this work, we believe some aspects of the evaluation strategy were not fully convincing.

Major comments

Supervised vs. Unsupervised Integration and Evaluation Metrics
In line 134, the authors report that the supervised version of Palette outperforms the unsupervised mode. However, the manuscript does not provide sufficient detail on how cell type annotation completeness or quality influenced these results. In practical applications, cell-type annotations are often incomplete or partially missing, and therefore the supervised framework should be evaluated under such realistic conditions (e.g., by introducing missing or “NA” labels). This would be feasible given that by default only 25% top-ranked SW cells are selected.

It also remains unclear how the annotations used for supervision were originally obtained — were they derived from a single modality (such as transcriptomic data) or from multi-modal consensus annotations? If annotations were modality-specific, this could introduce bias toward that modality and artificially enhance the apparent performance of supervised integration. It would be important to potential limitations.

In addition, the use of the same cell-type annotations used both for training (in supervised integration or batch correction) and for evaluation, introduces overfitting or validation bias.
This brings also the question of Palette's robustness to errors in cell type labels and to incomplete annotations, a common challenge in real-world single-cell datasets.
These and other challenges of benchmarking performance of supervised integration methods were addressed e.g. in https://www.nature.com/articles/s41467-024-45240-z

"Modality mixing" as an evaluation metric
Modality mixing metrics aim to quantify how well (cells from) datasets with different modality compositions are mixed. The use of modality mixing metrics (such as the Seurat Alignment Score, modality kBET, and modality LISI F1) requires careful interpretation. In particular, the current approach may not provide an unbiased assessment of integration quality because it relies on k-nearest neighbor (kNN) graphs constructed in the integrated latent space, which depend itself on the integration method.
Further, when modality coverage is highly imbalanced, or when the graph is built using closest kNN structures derived from one dominant modality, the resulting "mixing" may reflect technical artifacts rather than true biological alignment. It would be important to discuss these limitations and eg complement these metrics with external biological validations (e.g., preservation of known cell-type markers or cross-modality feature correlations).

Definition and Scope of "Differentially Expressed Features"
In lines 208–211, the authors state that, after removing Palette-identified differentially expressed features (DEFs), they annotated ten immune cell subtypes across transcriptomic, epigenomic, and proteomic layers (Fig. 3h–i). However, it is unclear how these DEFs were obtained. Earlier in the manuscript (Lines 198–207), the DEFs were described as differences between stimulated and control T cells, not across all immune cell types.
The authors should clarify whether the same T cell–specific DEF set was used for this broader immune cell analysis, or whether new DEFs were computed across all immune populations. In particular, it is not evident how removing features derived from a single cell type would impact subtype resolution across multiple immune lineages.
The 2 distinct NK clusters were already apparent before removing Palette-identified DEFs (Fig 3b).

Clarification of Supervised vs. Unsupervised Mode in Spatial Integration
In lines 311–325, the authors evaluate Palette on a mosaic integration task using low-resolution spatial multimodal data from human tonsil sections. However, it is not specified whether the analysis was performed using the supervised or unsupervised mode of Palette.
If supervised integration was conducted, which cell type labels were used?

Single-modality Palette integration performance
The authors demonstrate Palette's performance in cross-species single-cell RNA integration using both orthologous and non-orthologous genes, showing strong results compared to state-of-the-art methods. However, since Palette employs Bi-sPCA for intra-modality batch correction and dimensionality reduction, it would be valuable to explicitly evaluate how Palette performs as a standalone unimodal batch correction method for RNA data outside the scope of cross-species integration (Suppl Fig 25). Including this benchmark against established single-modality tools would strengthen the paper by showing whether Palette also contributes meaningfully to RNA-only batch correction tasks, beyond its multimodal integration capabilities, or whether users should use a different method for RNA-only integration.

Code review and reproducibility
The provided R package (https://github.com/qiongyusheng/Palette) is not readily installable, as it has issues with BiocManager and lacks the required biocViews field in the package description. In addition, the data needed to run the tutorials is only available upon request, limiting transparency and accessibility for independent validation. The accompanying reproducibility repository (https://github.com/qiongyusheng/Palette_reproducibility) contains incomplete code and no accessible datasets, preventing full replication of the published results. The authors should ensure that all code and data required to reproduce the main figures and benchmarks are publicly available and executable without additional data requests.

Minor comments

Supplementary Figure 20: No inclusion of plot c) in foot note.

(Remarks on code availability)
The provided R package (https://github.com/qiongyusheng/Palette) is not readily installable, as it has issues with

BiocManager and lacks the required biocViews field in the package description. In addition, the data needed to run the tutorials is only available upon request, limiting transparency and accessibility for independent validation. The accompanying reproducibility repository (https://github.com/qiongyusheng/Palette_reproducibility) contains incomplete code and no accessible datasets, preventing full replication of the published results

Reviewer #3

(Remarks to the Author)
I co-reviewed this manuscript with one of the reviewers who provided the listed reports. This is part of the Nature Communications initiative to facilitate training in peer review and to provide appropriate recognition for Early Career Researchers who co-review manuscripts.

(Remarks on code availability)
R package: https://github.com/qiongyusheng/Palette.
- Not readily installation, issues with BiocManager: not `biocViews` included in description.
- Data for taking the tutorials only upon request.

Results reproducibility: https://github.com/qiongyusheng/Palette_reproducibility.
- Incomplete code and no available data to reproduce results.

Reviewer #4

(Remarks to the Author)
Sheng et al present Palette, a computational tool for mosaic integration of multi-modal single-cell datasets. They provide a detailed description of the mathematical principles behind the method, benchmark it against existing methods, and demonstrate its applicability in a variety of scenarios. Overall, the manuscript is very strong and the tool addresses an important problem in the field of single cell genomics. However there are a few concerns, addressing which would further strengthen this work:

Could the authors provide data on how computational resource requirements and runtime change with dataset size?

Could the authors include a discussion on the tradeoff between the length of propagation path and the sparsity of modalities that lie on a path? E.g. is it better to follow a longer path if the shortest path means traversing through one or more extremely sparse data modalities?

Could the authors provide their rationale for choosing specific values of T1, theta-m, lambda-m and other important, modality specific parameters? This will especially be helpful for users who wish to use this for new single-cell modalities.

Could the authors provide quantitative assessment of inference accuracy for missing modalities by holding out specific modalities, in cases where multi-omic data is available. Does this performance vary significantly by modality?

Could authors clarify the abbreviation TEA where it is first mentioned in the results and also explain what “randomly generated dataset” means (line 703). This will help simplify the text for readers.

(Remarks on code availability)
The authors provide code on github to install their package and tutorials to sufficiently guide data analysis.

Version 1:

Reviewer comments:

Reviewer #1

(Remarks to the Author)
In their revised manuscript, the authors have now addressed my initial concerns regarding the validation of their method, its comparative benchmarking, and its overall methodological contribution.

(Remarks on code availability)
The authors have now mapped the paper's analyses to the corresponding code and parameters, making the results reproducible.

Reviewer #2

(Remarks to the Author)
The authors have carefully addressed all our comments, providing a thorough and well-structured rebuttal and revising the manuscript accordingly.

The expanded evaluation strategy includes experiments under perturbed annotation settings, robustness and sensitivity

analyses of modality mixing metrics, and single-modality integration benchmarks.

The revised manuscript now convincingly presents Palette as a valuable new tool for multi-modal dataset integration.

A minor point remains for clarification:

Modified Methods (lines 885–891):
In the description of the horizontal and rectangular integration benchmarks, different numbers of highly variable genes (HVGs) are selected across datasets (4,000 HVGs for the MTG dataset versus 2,000 HVGs for the immune and pancreas datasets). It would be important to explain the rationale for using different number of HVG across these benchmarks. A brief justification (e.g., differences in dataset size, complexity, or consistency with prior studies) would help avoid potential confusion for readers.

(Remarks on code availability)
The package Palette is now installable and the tutorials are clear

Reviewer #3

(Remarks to the Author)
I co-reviewed this manuscript with one of the reviewers who provided the listed reports. This is part of the Nature Communications initiative to facilitate training in peer review and to provide appropriate recognition for Early Career Researchers who co-review manuscripts.

(Remarks on code availability)

Reviewer #4

(Remarks to the Author)
The authors have sufficiently addressed my comments, good luck to the team with the remaining publication process!

(Remarks on code availability)
The authors provide code on github to install their package and tutorials to sufficiently guide data analysis.

Open Access This Peer Review File is licensed under a Creative Commons Attribution 4.0 International License, which permits use, sharing, adaptation, distribution and reproduction in any medium or format, as long as you give appropriate credit to the original author(s) and the source, provide a link to the Creative Commons license, and indicate if changes were made.
In cases where reviewers are anonymous, credit should be given to 'Anonymous Referee' and the source.
The images or other third party material in this Peer Review File are included in the article’s Creative Commons license,

unless indicated otherwise in a credit line to the material. If material is not included in the article's Creative Commons license and your intended use is not permitted by statutory regulation or exceeds the permitted use, you will need to obtain permission directly from the copyright holder.
To view a copy of this license, visit https://creativecommons.org/licenses/by/4.0/

Point-by-point responses to the comments of Reviewer #1

Comment: The study presents Palette, a novel computational framework designed to address a critical and timely challenge in single-cell genomics: the mosaic integration of multimodal datasets with imbalanced modality compositions. The authors have invested substantial effort in developing this method and have demonstrated its potential across a wide range of complex scenarios. However, before this manuscript can be considered for publication, several fundamental concerns regarding the experimental design, methodological novelty, evaluation, and comparison with existing techniques must be addressed. The current manuscript fails to provide sufficiently compelling evidence to support its claims and requires substantial additional experimentation and deeper analysis to strengthen its scientific rigor.

Response: We thank the reviewer for the thoughtful comments and constructive suggestions regarding our work. In response, we have carefully revised the manuscript and conducted extensive additional analyses and benchmarking experiments to systematically address these fundamental concerns and to strengthen the overall scientific rigor of the study. The major revisions prompted by the reviewer's comments are summarized below.

(1) We have expanded the methodological description to more clearly articulate the novelty of Palette, the design rationale of the proposed framework, and the role of core model components. We have also added detailed justification for parameter choices and practical guidance for new modalities.

(2) We have revised the modality mixing metrics to assess the quality of alignment between different modality compositions more effectively.

(3) We have extended the diagonal integration benchmarking to include additional representative methods, including GLUE, scConfluence, MaxFuse, and SIMBA.

(4) We have added a benchmarking analysis for missing modality prediction (imputation).

(5) For reference-based integration, Multigrate, a method that utilizes the entire reference atlas, has been included for benchmarking.

(6) We have added additional benchmarking results for both mosaic and diagonal integration using restricted feature sets for Palette.

For more details, please see our point-by-point response below. For the reviewer's convenience, the corresponding revised manuscript is attached at the end of each response and is underlined to facilitate distinction from the response text.

Comment: 1. The central claim of the study is its ability to handle "imbalanced modality composition", yet this is not adequately reflected in the experimental design. In my view, an imbalance in the proportion of batches with different modality compositions (e.g., a 9:1 ratio of complete-modality batches to missing-modality batches) is not the primary challenge; integration may even be easier in such cases due to the abundance of complete information. A more challenging and realistic scenario involves severe imbalances in cell or batch counts associated with different modalities (e.g., integrating nine 'RNA-only' batches with one 'RNA+ATAC' batch) or extensive modality missingness. The current experimental setup fails to simulate these more difficult scenarios, thus weakening the claims about Palette's robustness in handling this critical issue.

Response: We thank the reviewer for this insightful comment. In the original manuscript, the integration scenarios mentioned above are already taken into consideration. Specifically, in the original benchmark experiments, three integration scenarios, including TEA scenarios 1 and 2, and BMMC scenario 2, exhibit extensive modality missingness (Supplementary Figs. 2a, 2d, and 3d in the revised manuscript). In addition, in the original cross-species MOp mosaic integration experiment, the mouse scATAC-seq batch ('Mouse 10xATAC' in Supplementary Fig. 24a of the revised manuscript) contains only 3,512 cells (approximately 1.36% of the total 257,943 cells), constituting a severe imbalance in modality-associated cell counts. We have clarified the challenging characteristics of these experiments more clearly in the revised manuscript (*lines 114-116* and *256-258*).

Moreover, to directly evaluate the Palette's robustness in the reviewer's specific difficult example, we constructed the scenario of integrating nine "RNA-only" batches with one "RNA+ATAC" batch using human heart 10x Multiome data (10 batches of paired snRNA-seq and snATAC-seq) by masking the snATAC-seq profiles from nine batches. As shown in Reviewer Fig. 1, Palette maintains strong performance under this extreme configuration, demonstrating its robustness to scenarios with substantial modality imbalance and limited cross-modality overlap.

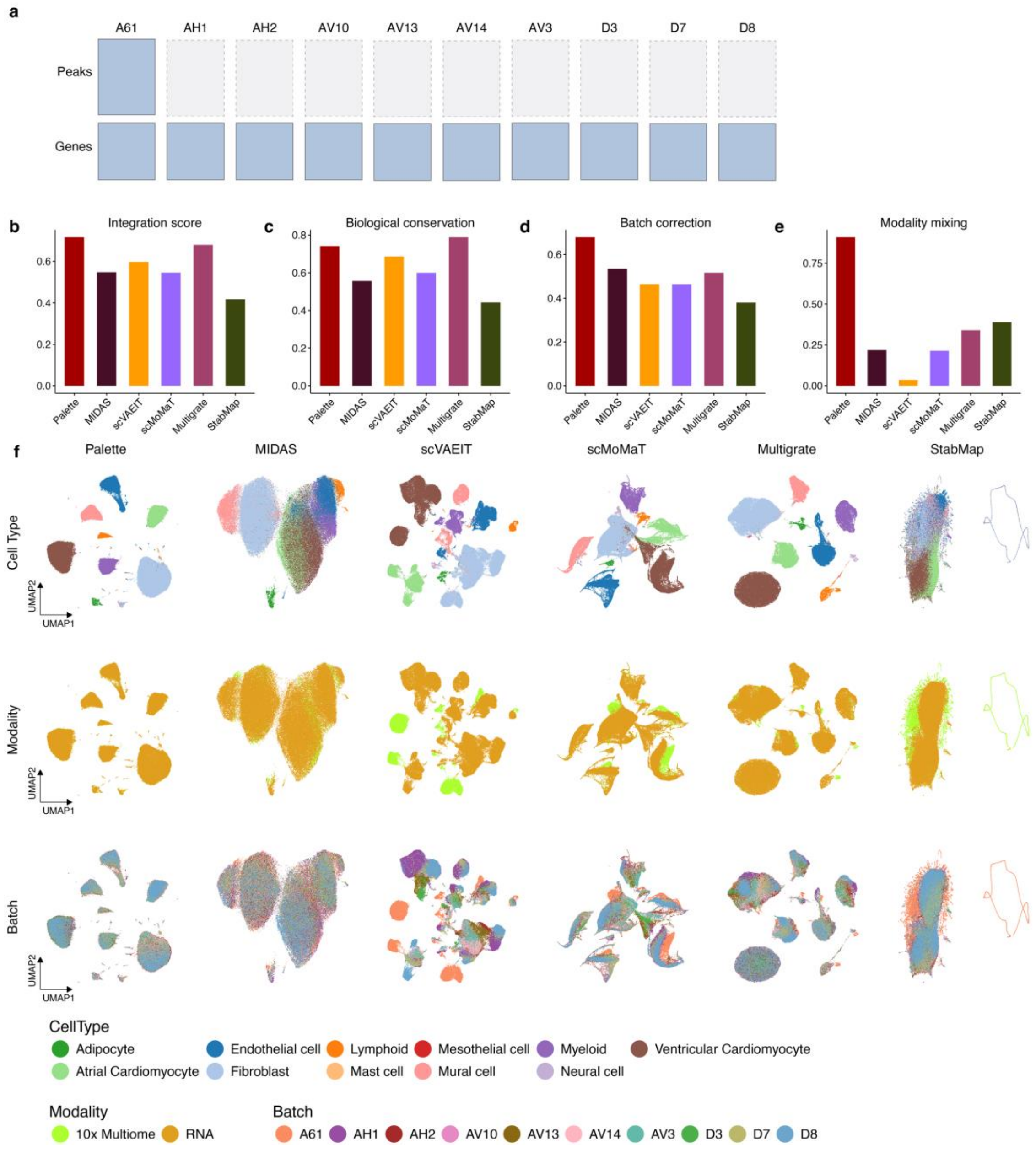

Reviewer Figure 1. Mosaic integration results on the human heart 10x Multiome dataset. a. Schematic of the modality composition of each batch in the human heart 10x Multiome dataset. **b–e.** Comparison of integration performance across methods, including overall integration (**b**), biological conservation (**c**), batch correction (**d**), and modality mixing scores (**e**). **f.** UMAP visualizations of integrated cell embeddings generated by Palette and five other integration methods for the mosaic integration of human heart 10x Multiome dataset. Cells are colored by batch (top row), modality composition (middle row), and cell type (bottom row).

Modified Results:

Lines 109-116: Specifically, for trimodal integration, we designed a full-modality single-hop and a multi-hop scenario using the TEA-seq technology data[9], corresponding to TEA scenarios 1 and 2,

respectively. To evaluate integration across different sequencing technologies, we also created two scenarios of single-hop and multi-hop, referred to as BMMC scenarios 1 and 2, respectively, by combining two different technologies of the human bone marrow mononuclear cells (BMMCs) dataset. Additionally, except for BMMC scenario 2, the other three trimodal integration scenarios are designed to handle extensive modality missingness.

Lines 254-258: We further assessed Palette's ability to perform cross-species mosaic integration. We assembled a primary motor cortex (MOp) dataset comprising eight batches from human, marmoset, and mouse, spanning both transcriptome and chromatin accessibility modalities[47,48]. The dataset exhibits a pronounced imbalance in cell numbers across modality compositions (Supplementary Table 1), which further complicates coherent cross-modality and cross-species alignment.

Comment: 2. The proposed "Modality mixing metrics" are intended to evaluate the mixing of cells with different modality compositions, which the authors use to distinguish between batch effects and modality composition heterogeneity. However, the design of this metric is flawed. Because it measures the mixing between batches that have different modality compositions, rather than the alignment of the modalities themselves within shared cell types, this metric is largely coupled with batch correction metrics (e.g., bASW, iLISI). Consequently, better batch alignment will invariably lead to a higher modality mixing score, making this metric unable to independently and effectively assess the quality of modality alignment.

Response: We thank the reviewer for this constructive comment. In the revised manuscript, we refined the metrics for modality mixing. Specifically, we compute the Seurat Alignment Score, kBET, and normalized iLISI (referred to as CSAS, modality kBET, and CiLISI in the revised manuscript, respectively) within each cell type using modality composition labels, ensuring that the metric evaluates modality composition alignment within biologically comparable populations rather than being influenced by differences in cell-type composition. Under this refined evaluation, Palette maintains consistently strong performance. Moreover, the revised results showed that methods with stronger batch mixing do not always exhibit better modality mixing.

We further note that, in the mosaic integration setting, modality-level alignment cannot be assessed directly by aligning of modality-specific embeddings, as each cell is represented by a single unified latent embedding rather than separate modality-specific representations. This limitation is inherent to the mosaic problem formulation and is shared by existing mosaic integration frameworks. Accordingly, we focus on quantifying the alignment of different modality compositions within the unified latent space, which directly investigates whether modality composition heterogeneity induces segregation among cells.

We have updated the **Methods** section in the revised manuscript (*lines 1083–1115*) to clarify the revised definition and computation of the modality mixing metrics.

Modified Figures:

d

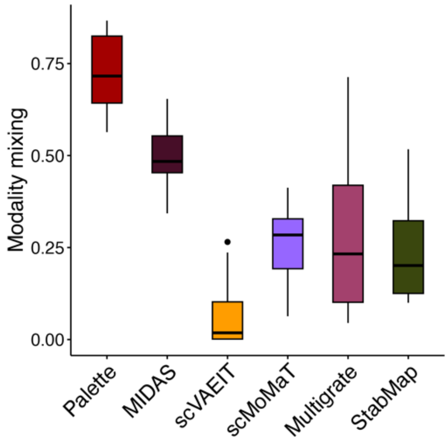

Figure 2. Systematic benchmarking of Palette against state-of-the-art integration methods. d. Box plot of overall modality mixing scores across methods. The central lines mark the median values, the boxes show the quartiles, and the whiskers show the rest of the distribution.

c

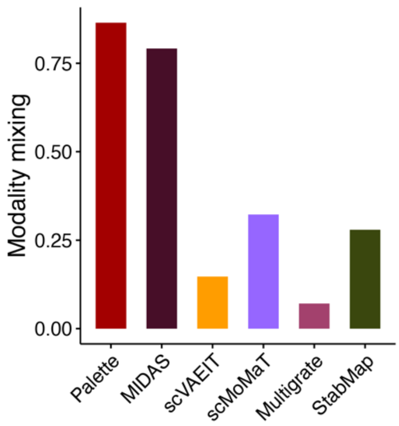

Figure 3. Results on the cross-condition human PBMC dataset. c. Comparison of modality mixing score for different integration methods.

Modified Supplementary Figures:

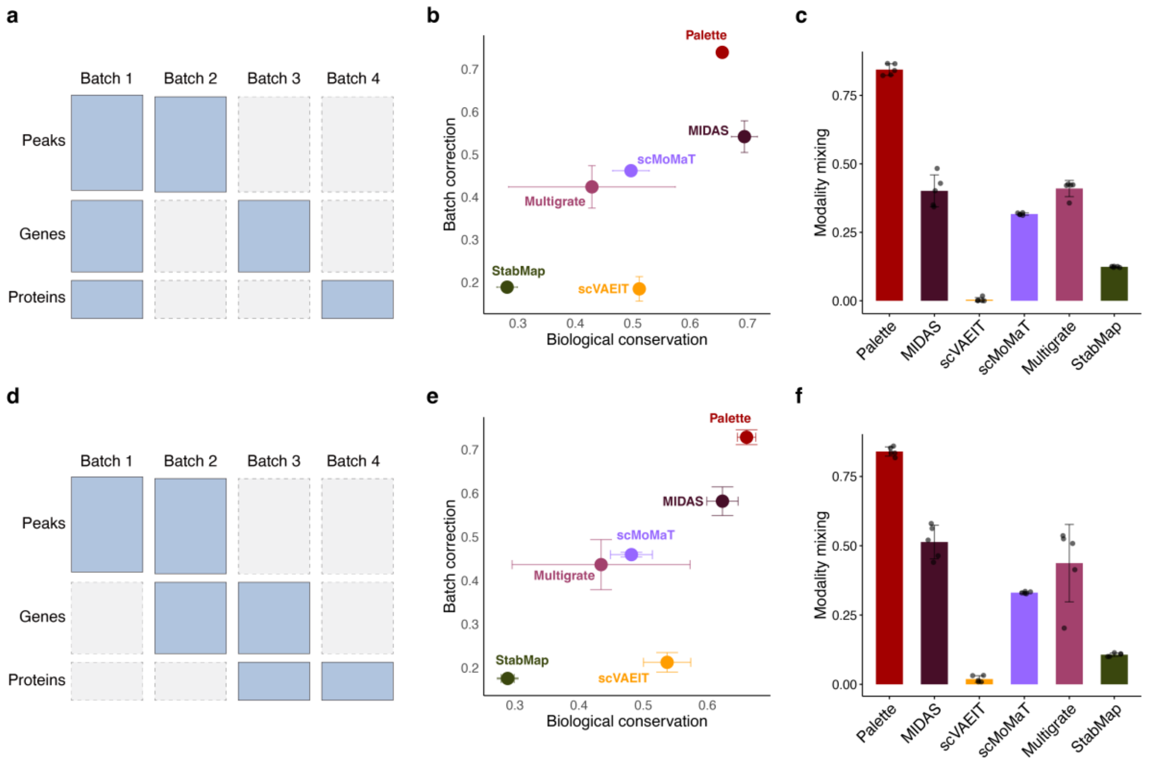

Supplementary Figure 2. Benchmarking of Palette and other integration methods on the TEA scenarios 1 and 2. The top row (**a**–**c**) summarizes results for TEA scenario 1: (**a**) modality composition of each batch; (**b**) comparison of mean biological conservation versus mean batch correction across methods; and (**c**) mean modality mixing scores. The bottom row (**d**–**f**) presents the corresponding results for TEA scenario 2. Error bars represent the standard error across evaluated tasks, from $n = 5$ randomly generated sub-experiments.

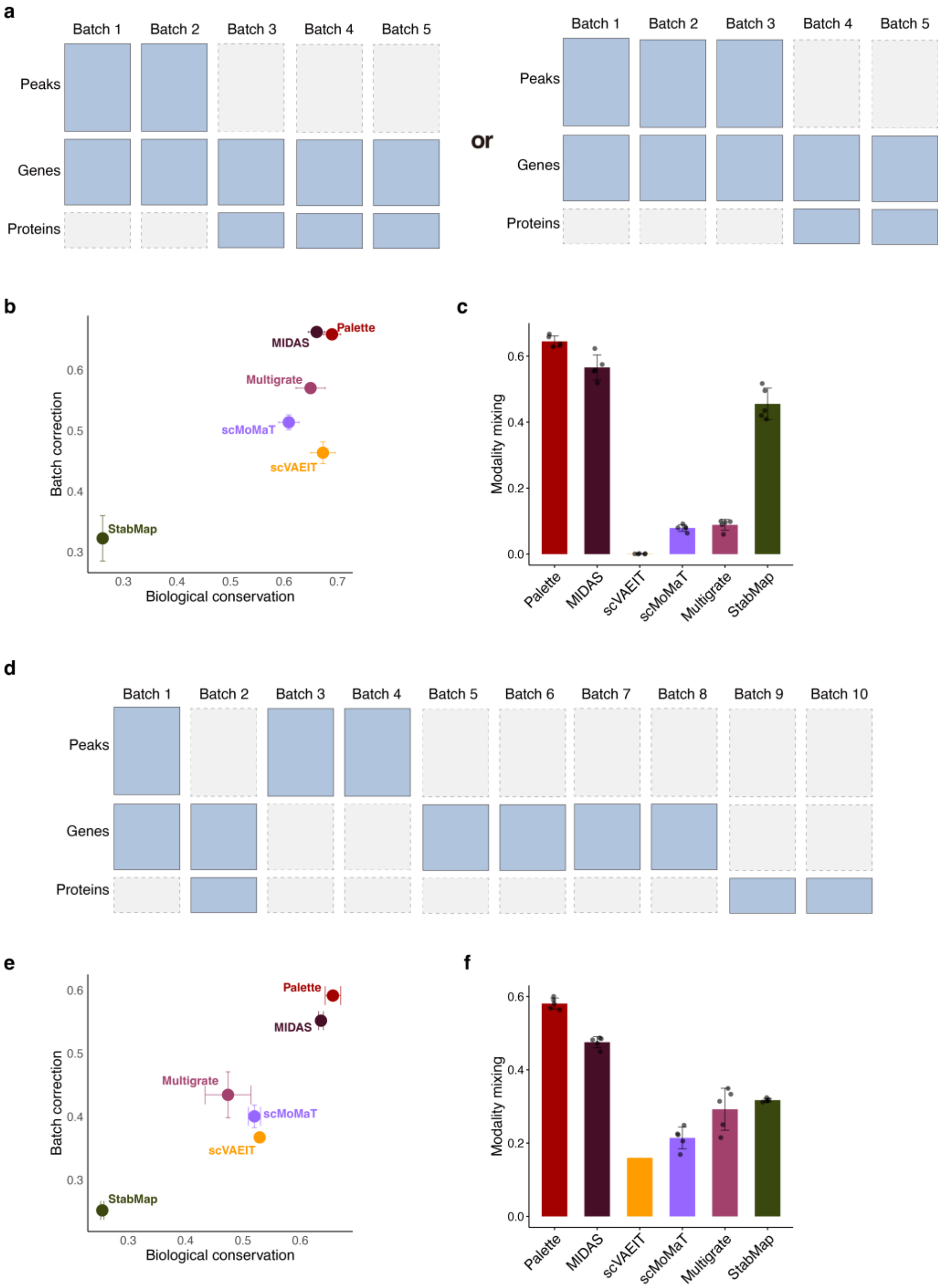

Supplementary Figure 3. Benchmarking of Palette and other integration methods on the BMMC scenarios 1 and 2. The top two rows (**a**–**c**) summarize results for BMMC scenario 1: (**a**) modality composition of each batch; (**b**) mean biological conservation versus batch correction across methods; and (**c**) mean modality mixing scores. The bottom two rows (**d**–**f**) present the corresponding results for BMMC scenario 2. Error bars represent the standard error across evaluated tasks, from $n = 5$ randomly generated sub-experiments. For BMMC scenario 2, scVAEIT completed only one sub-experiment, so no error bars are shown for this method.

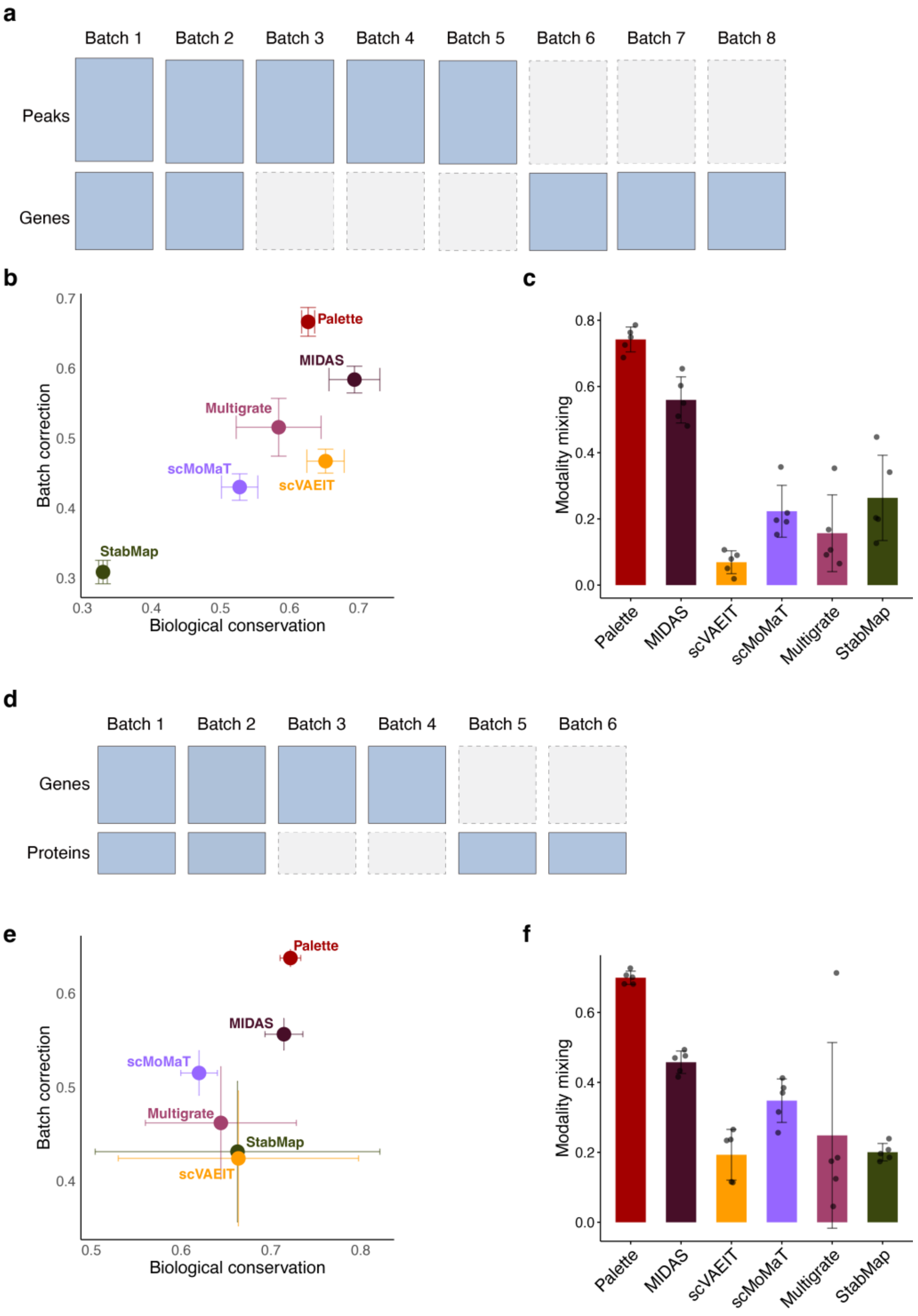

Supplementary Figure 4. Benchmarking of Palette and other integration methods on the Retina and Ab-seq datasets. The top two rows (**a**–**c**) summarize results for the Retina dataset: (**a**) modality composition of each batch; (**b**) comparison of mean biological conservation versus mean batch correction across methods; and (**c**) mean modality mixing scores. The bottom two rows (**d**–**f**) present the corresponding results for the Ab-seq dataset. Error bars indicate the standard error across evaluated tasks, from *n* = 5 randomly generated sub-experiments.

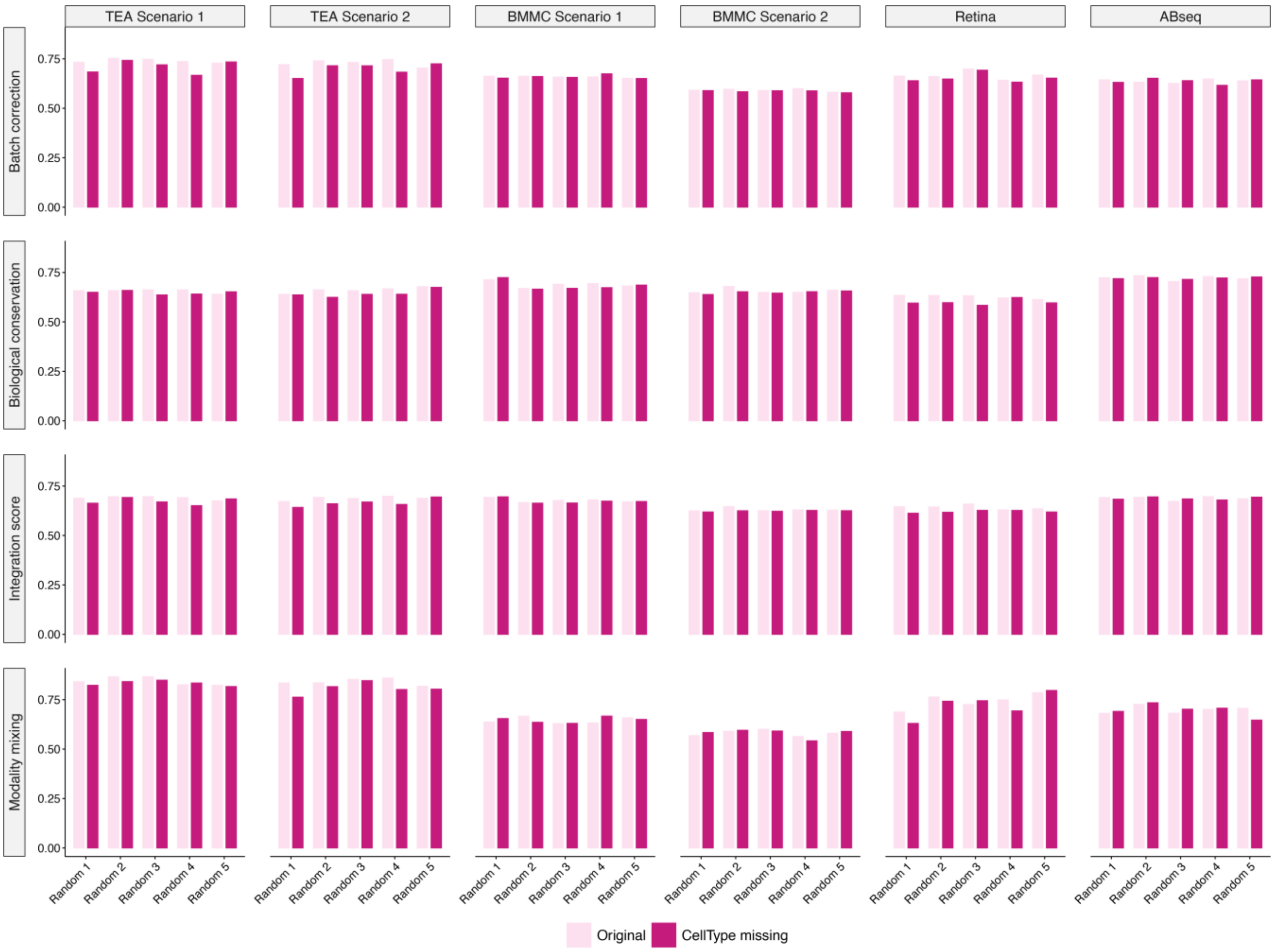

Supplementary Figure 11. Evaluation of Palette's integration performance under imbalanced cell type compositions. Across six integration scenarios and multiple metrics, we compared results on original datasets with those from modified versions in which one cell type was randomly removed from each batch to introduce compositional imbalance. Palette maintained consistently high performance across all scenarios, demonstrating robustness to biological heterogeneity.

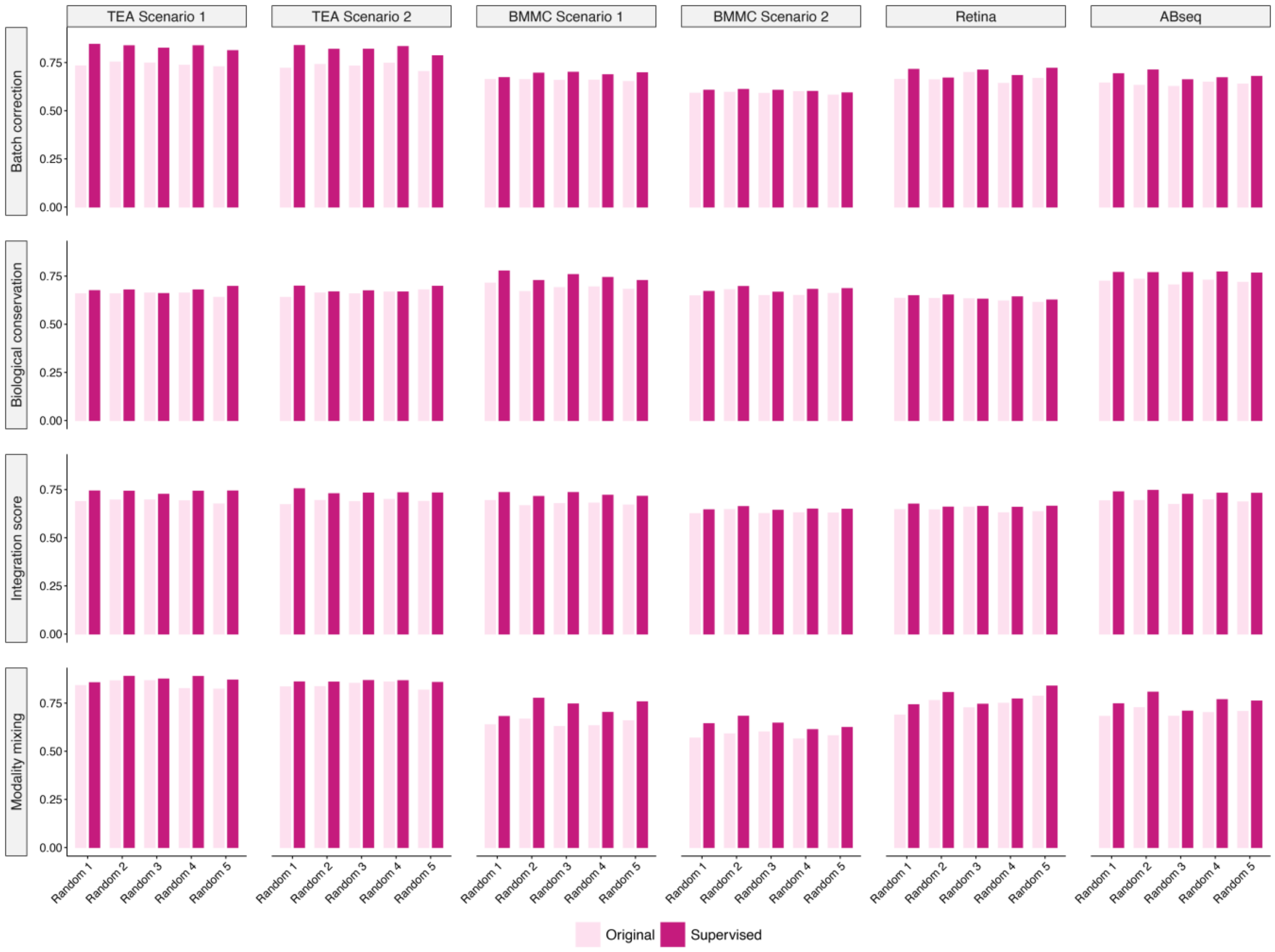

Supplementary Figure 13. Evaluation of Palette's supervised integration performance. Across six integration scenarios and multiple evaluation metrics, we compared Palette's supervised integration mode with its unsupervised counterpart. Supervised integration consistently yielded improved quantitative scores across scenarios.

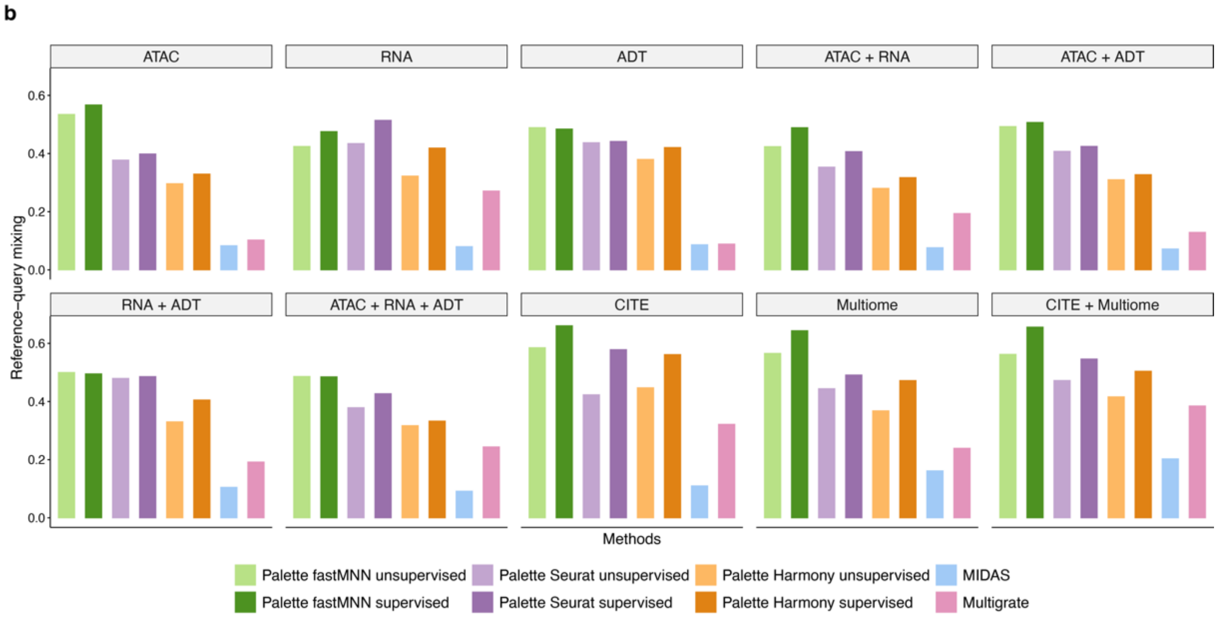

Supplementary Figure 19. Evaluation of cell type label transfer and dataset mixing in reference-based integration across multiple query scenarios. b. Bar plots showing the mixing scores between reference and query cells for each query scenario.

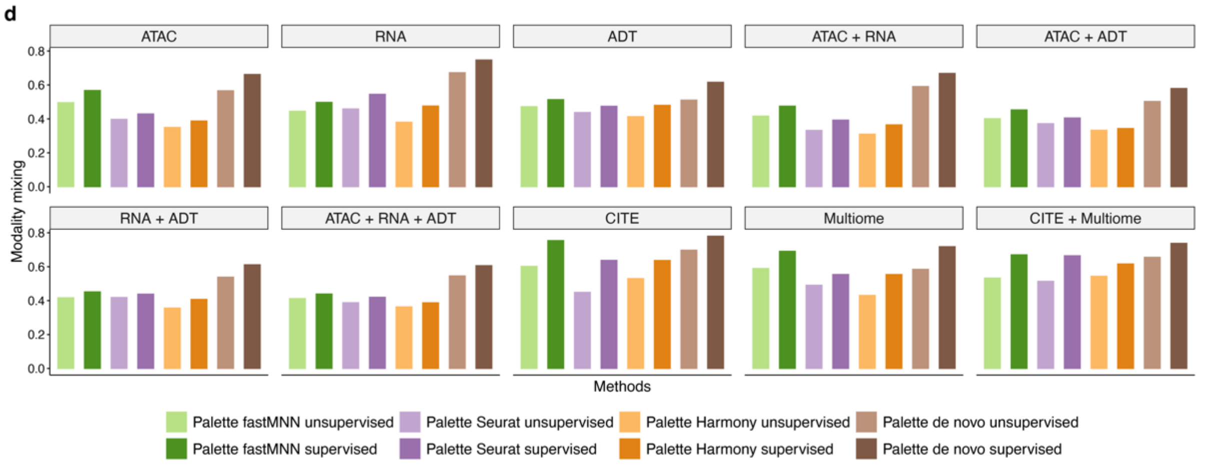

Supplementary Figure 20. Comparison of integration performance between reference-based and de novo integration across multiple query scenarios. We quantitatively evaluated four integration metrics: (**a**) batch correction score, (**b**) biological conservation score, (**c**) overall integration score, and (**d**) modality mixing score. For supervised de novo integration, cell type labels for each query dataset were obtained by transferring annotations from the reference using reference-based integration with supervised reference embeddings. For evaluation purposes, the ground truth cell type labels of the query datasets were used to compute all metrics.

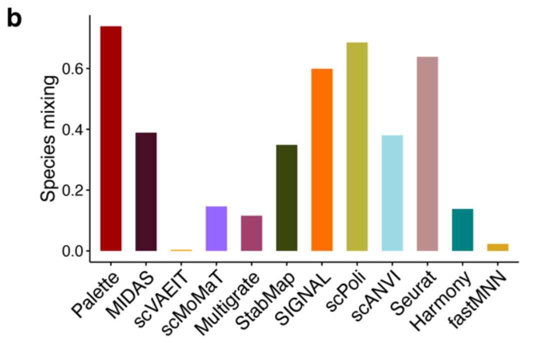

Supplementary Figure 27. Integration results of cross-species WAT data based on orthologous genes. a, b. Comparison of overall integration scores (**a**) and species mixing scores (**b**) across different integration methods.

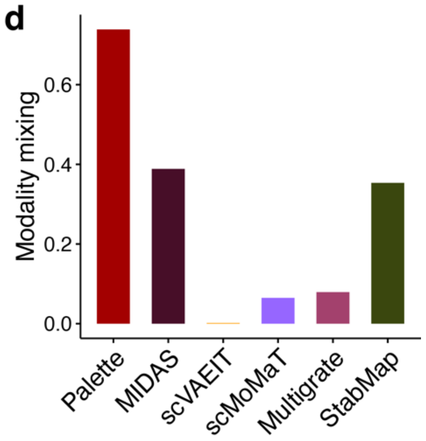

Supplementary Figure 28. Comparison of integration performance with and without inclusion of non-orthologous genes. c, d. Comparison of overall integration scores (**c**) and modality mixing scores (**d**) across methods under the non-orthologous gene inclusion setting.

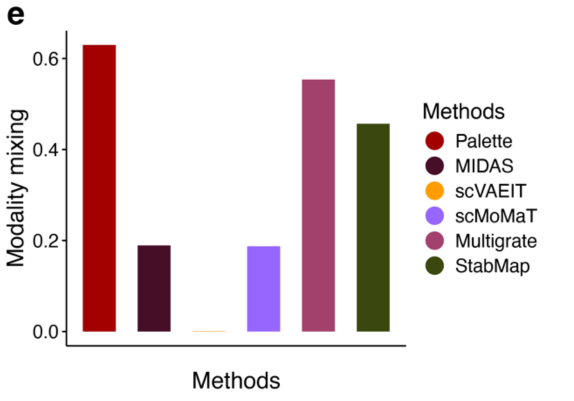

Supplementary Figure 29. Integration results of human tonsil 10x Visium data. e. Comparison of modality mixing scores across methods.

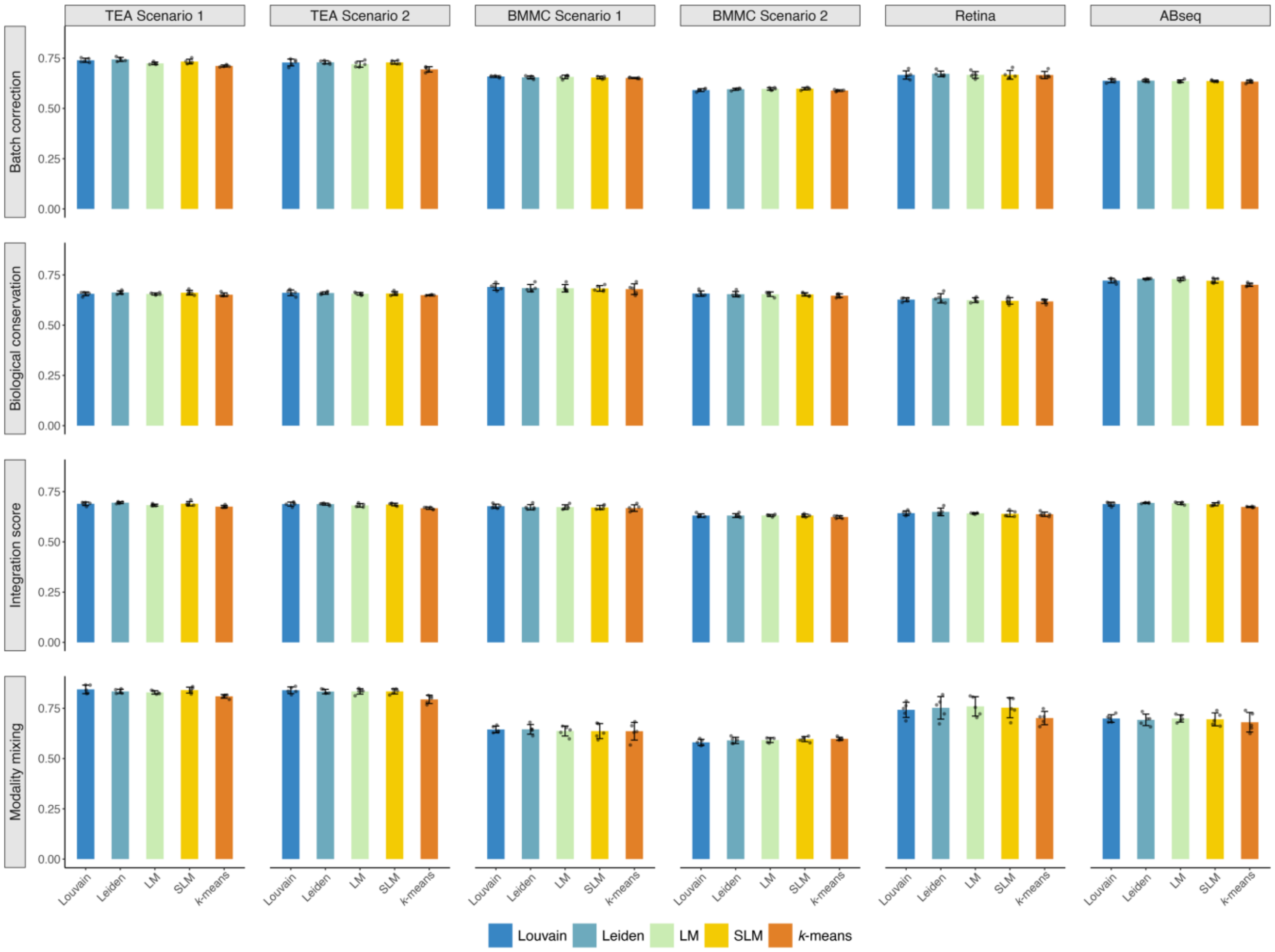

Supplementary Figure 41. Benchmarking clustering algorithms within the unsupervised Palette framework. We evaluated integration performance using multiple quantitative metrics across different clustering strategies combined with Palette: Louvain (default in Palette), Leiden, Louvain with multilevel refinement (LM), smart local moving (SLM), and *k*-means. For Louvain, Leiden, LM, and SLM, the resolution parameter was fixed at 1, consistent with the default setting in Palette. For *k*-means, we specified the number of clusters to match the number of known cell types in each batch. Error bars represent the standard error across evaluated tasks, from *n* = 5 randomly generated sub-experiments.

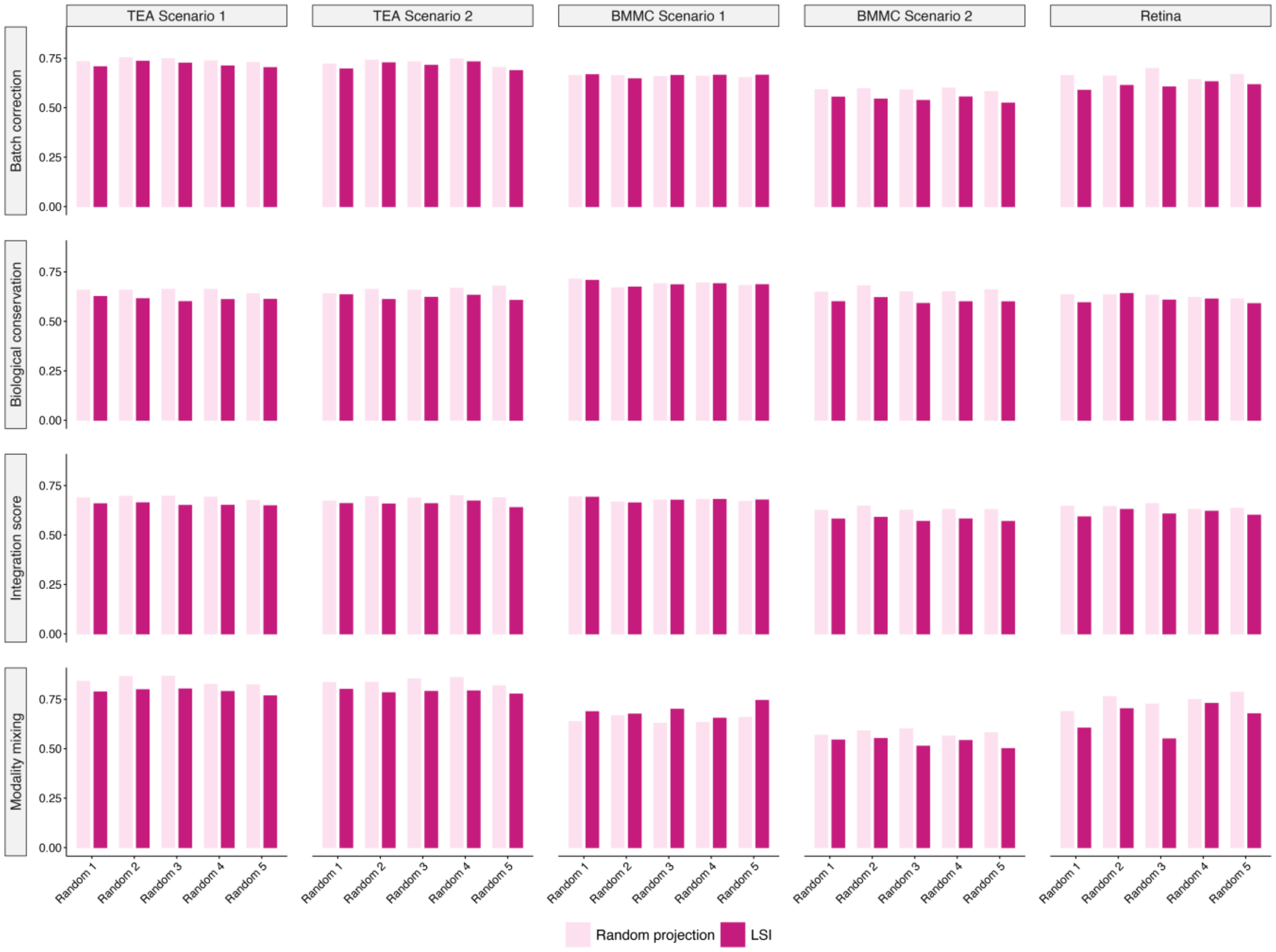

Supplementary Figure 42. Evaluation of Palette's integration performance using different pre-dimensionality reduction methods for chromatin accessibility data. Integration results based on two pre-dimensionality reduction strategies, random projection and LSI, were compared across five integration scenarios using multiple metrics.

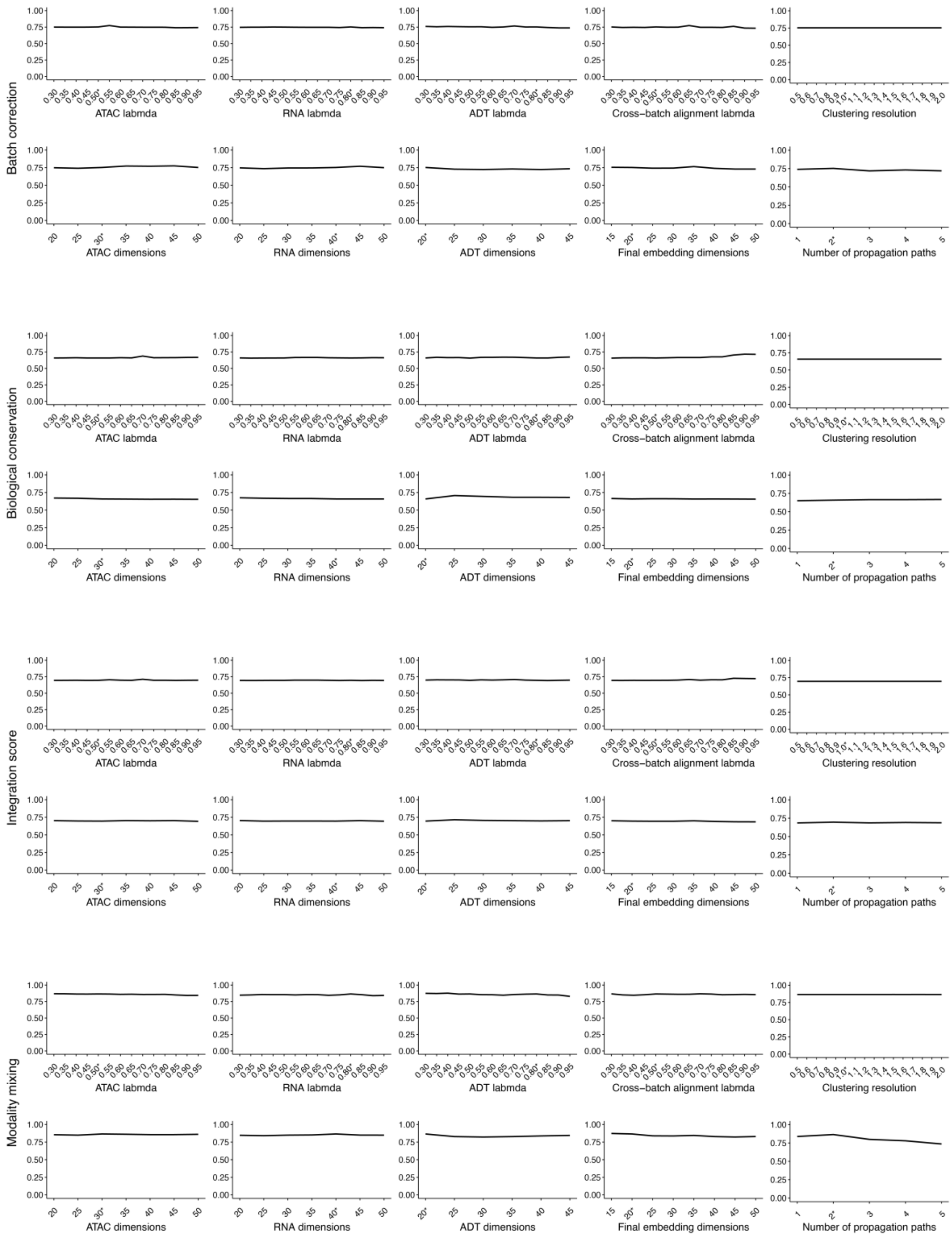

Supplementary Figure 46. Robustness benchmarking of Palette integration with respect to tuning parameters, using the dataset of the third sub-experiment under the TEA scenario 1. Integration performance was evaluated based on batch correction scores, biological conservation scores, overall integration scores, and modality mixing scores. These metrics were assessed across a range of tuning parameters, including: the Bi-sPCA regularization parameter (λ) used for different modalities and integration stages; clustering resolution for kernel matrix construction in unsupervised

mode; dimensionality settings for intra-modal joint reduction and final embedding; and the number of propagation paths. For protein-specific intra-modal dimensionality reduction, the dimension was set to 20 for the human PBMC TEA-seq dataset (which contains 46 ADT features) to ensure sufficient representation across all sub-experiments based on this dataset. In addition, asterisks (*) denote the default parameter settings used by Palette.

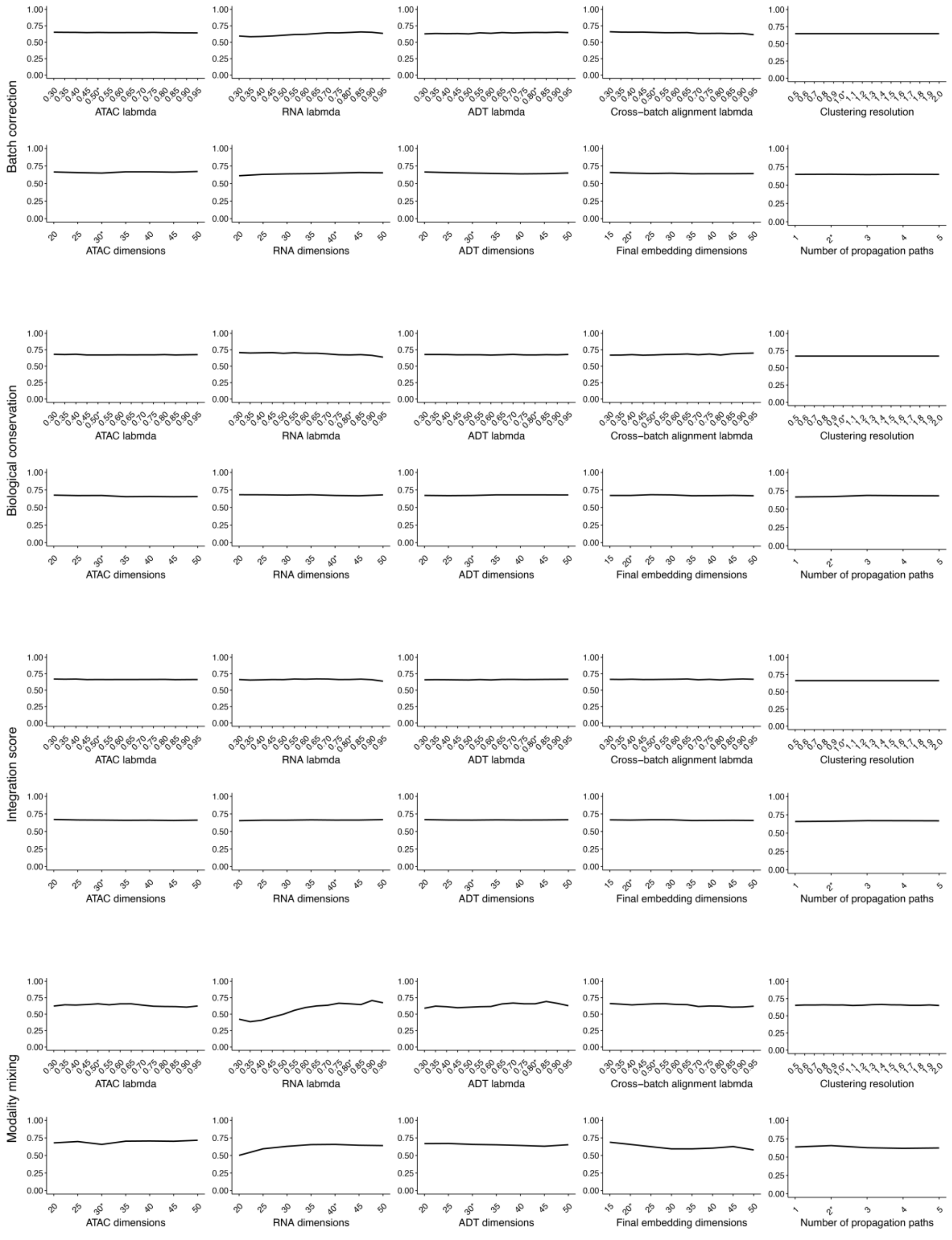

Supplementary Figure 47. Robustness benchmarking of Palette integration with respect to tuning parameters on the human BMMC CITE-seq and 10x Multiome dataset. Integration performance was evaluated based on batch correction scores, biological conservation scores, overall integration scores, and modality mixing scores. These metrics were assessed across a range of tuning parameters, including: the Bi-sPCA regularization parameter (λ) used for different modalities and integration stages; clustering resolution for kernel matrix construction in unsupervised mode; dimensionality settings for intra-modal joint reduction and final embedding; and the number of propagation paths. An asterisk (*) indicates the default parameter setting used by Palette.

Modified Methods (lines 1083-1115):

Modality mixing metrics. To quantify the mixing of cells originating from different modality compositions, we employed three metrics: the cell-type–stratified Seurat alignment score (CSAS), modality kBET, and CiLISI[31]. These metrics characterize the extent of modality mixing in mosaic integration settings but are not intended to serve as standalone measures of overall integration quality. It should therefore be evaluated together with additional biological metrics. In this study, integration quality was assessed by combining the modality mixing metric with the biological conservation and batch correction metrics.

CSAS. The Seurat alignment score (SAS), originally introduced by Butler et al.[104], measures how well datasets are aligned in a shared embedding space. To account for heterogeneity across cell types, we computed SAS separately within each cell type and reported the median value as CSAS. For a given cell type c, datasets representing different modality compositions were first downsampled to match the smallest group. SAS was then computed on the kNN graph as

$$SAS_c = 1 - \frac{\bar{x}_c - \frac{K_c}{N_c}}{K_c - \frac{K_c}{N_c}} ,$$

where N_c is the number of modality compositions present in cell type c, and $\bar{x}_c$ is the average number of neighbors sharing the same modality composition. Following Butler et al.[104], K_c was set to 1% of the downsampled cell count. As with SAS, CSAS ranges from 0 to 1, with higher values indicating stronger modality mixing.

Modality kBET. To evaluate mixing using local neighborhood homogeneity, we adapted kBET by treating modality composition labels as the grouping variable. kBET was computed within each cell type, and the median rejection rate across cell types was used as the modality kBET score.

CiLISI. CiLISI, originally proposed by Andreatta et al.[31], evaluates batch mixing by computing iLISI within individual cell types. To account for variability in cell type abundance and the differing modality compositions, we implemented a modified version. Within each cell type, datasets were downsampled to the smallest modality group, and iLISI was calculated on the downsampled cells. The

resulting values were normalized to the range 0 to 1, and the median across all cell types was reported as CiLISI.

Modality mixing score. The modality mixing score is defined as the mean value of CSAS, modality kBET, and CiLISI. We formulize this metric as follows:

$$S_{\mathrm{mod}} = (\mathrm{CSAS} + \mathrm{kBET}_{\mathrm{mod}} + \mathrm{CiLISI})/3,$$

where S_{mod} indicates the modality mixing score, and $\mathrm{kBET}_{\mathrm{mod}}$ denotes modality kBET, respectively. We further tested the behavior of these three metrics against four additional metrics using a suite of simulated scenarios designed to probe their sensitivity and robustness (Supplementary Fig. 51 and Supplementary Note 5).

Added Reference:

31. Andreatta, M. et al. Semi-supervised integration of single-cell transcriptomics data. *Nat. Commun.* **15,** 872 (2024).

Comment: 3. The scope of algorithmic comparisons is insufficient to establish the necessity of Palette. For specific integration scenarios, it is crucial to benchmark against the respective state-of-the-art methods. For instance, the spatial mosaic integration analysis should include comparisons with tools like SpatialGlue (Nat Meth 2024), MISO (Nat Meth 2025), MUSE (Nat Biotechnol 2022), and SIMO (Nat Commun 2025). Similarly, the diagonal integration section should be benchmarked against GLUE (Nat Biotechnol 2022), uniPort (Nat Commun 2022), scConfluence (Nat Commun 2024), MaxFuse (Nat Biotechnol 2023), and SIMBA (Nat Meth 2024). Comparing only against general-purpose mosaic integration methods fails to demonstrate that Palette offers a superior or necessary solution in these specialized domains.

Response: We thank the reviewer for this helpful comment. In response, we have expanded our benchmarking test to include more advanced and targeted methods.

For spatial mosaic integration task, to date, we are not aware of a published method that explicitly targets this setting. The methods mentioned by the reviewer are therefore not directly applicable without altering the problem definition or violating their modeling assumptions. Specifically, SpatialGlue and MISO perform vertical integration of multiple omics measured on the same spatial slice; MUSE integrates spatial transcriptomics with image morphology; and SIMO aligns spatial transcriptomics with single-cell data. None of these frameworks accepts mosaic-style inputs in which modality compositions differ across spatial batches, which is the defining characteristic of spatial mosaic integration in our study.

For diagonal integration, we expanded the benchmarking in the revised manuscript by incorporating additional state-of-the-art methods according to their applicable scenarios. Specifically, GLUE and SIMBA were included for the human kidney scRNA-seq and scATAC-seq integration task, as both

methods rely on strong feature correspondences. MaxFuse, which is designed for weak feature relationships, was evaluated on the PBMC, human tonsil, and human MOp diagonal integration tasks. scConfluence, which is applicable under both strong and weak correspondences, was included across all diagonal integration experiments. uniPort was already part of our original comparisons. These additions enable Palette to be evaluated alongside state-of-the-art methods specifically designed for diagonal integration. The results showed that Palette consistently outperformed other methods, achieving the highest performance (Fig. 5a and Supplementary Figs. 31–38).

We have added the new diagonal integration results to the **Results** section of the revised manuscript (*lines 375–380, 417, and 428*).

Modified Figure:

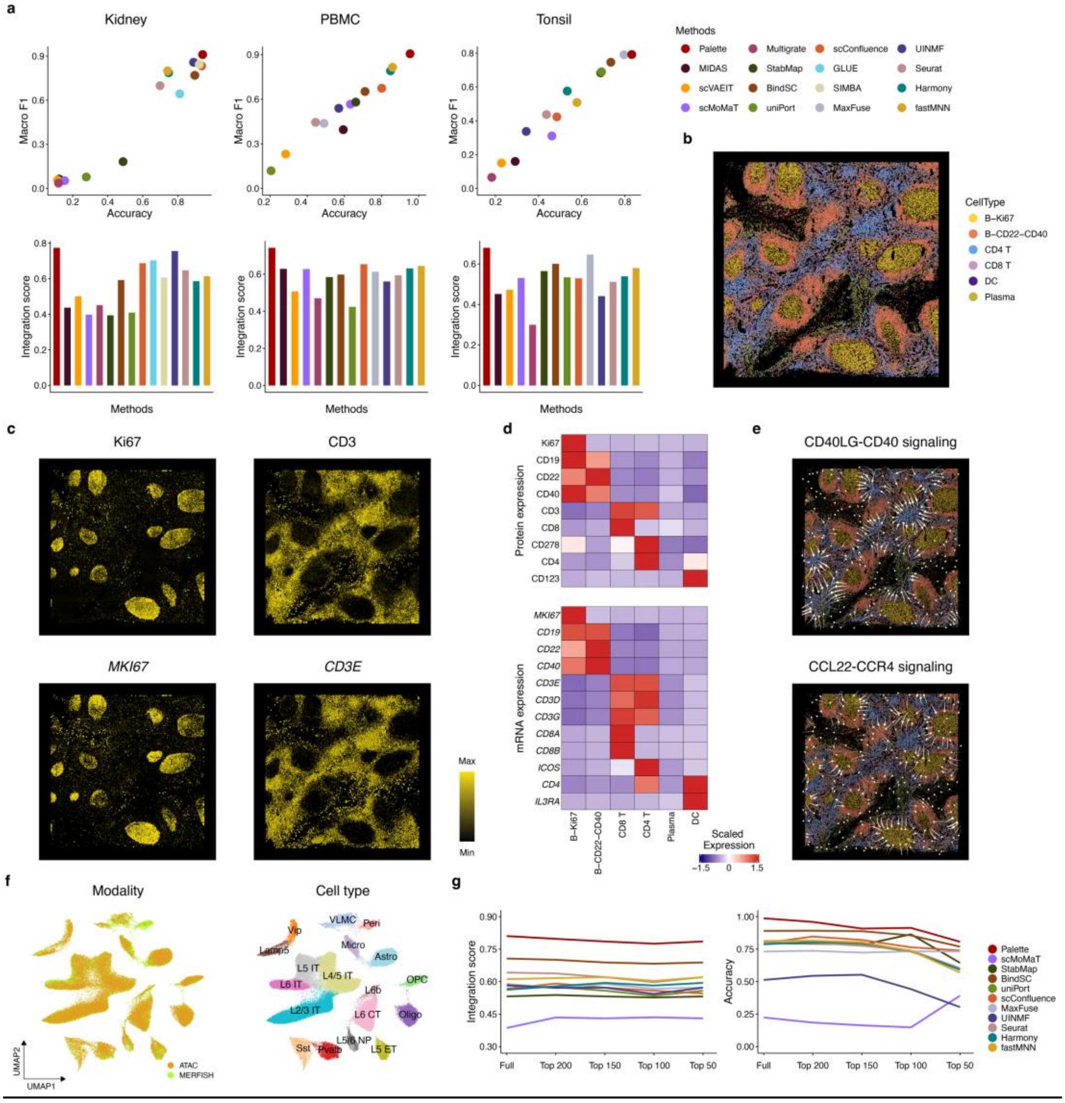

Figure 5. Diagonal integration results. a. Comparison of label transfer metrics (top row) and overall integration scores (bottom row) across three tasks for different integration methods. **b.** Overview of the CODEX human tonsil dataset. **c.** Spatial expression patterns of representative markers: protein (top

row; Ki-67) and inferred mRNA expression (bottom row; *MKI67*) for a proliferation marker (left); protein (top row; CD3) and inferred mRNA expression (bottom row; *CD3E*) for a T cell marker (right). **d.** Expression patterns of selected cell type markers across the protein and inferred mRNA layers. **e.** Signaling directionality of CD40LG-CD40 (top row) and CCL22-CCR4 (bottom row) interactions. **f.** UMAP visualizations of integrated cell embeddings in the mouse MOp dataset, colored by modality (left) and re-annotated cell type (right). **g.** Line plots comparing overall integration scores (left) and label transfer accuracy (right) for different integration methods under reduced feature overlap.

Modified supplementary Figures:

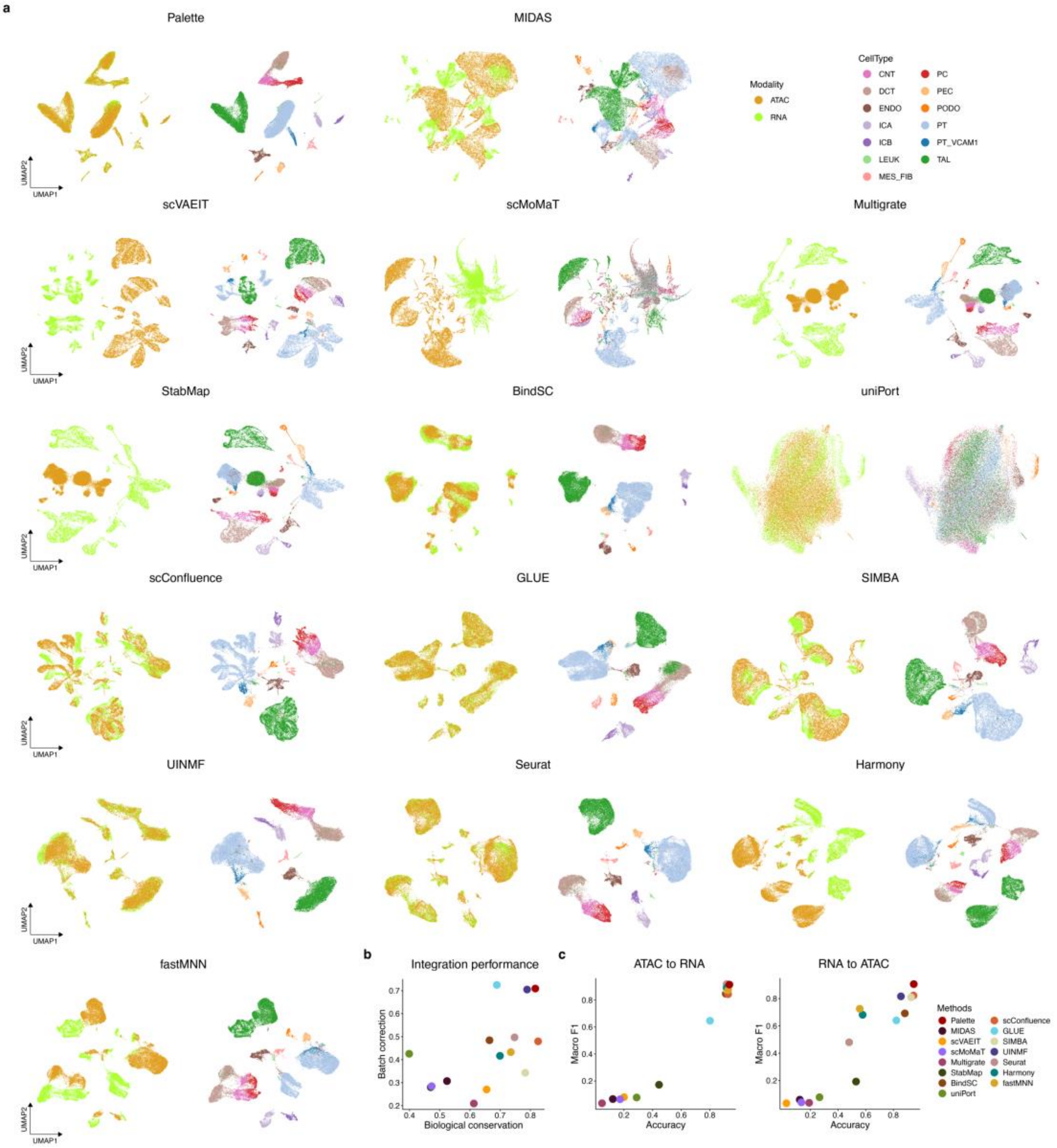

Supplementary Figure 31. Diagonal integration results for the human kidney data. a. UMAP visualizations of integrated cell embeddings generated by Palette and 14 other integration methods. Cells are colored by modality and cell type labels. **b.** Scatter plot of biological conservation scores versus batch correction scores for different integration methods. **c.** Comparison of cross-modality label

transfer performance across methods. Cell type labels from the chromatin accessibility data were transferred to predict the identities of cells in the transcriptome data (left), and vice versa (right).

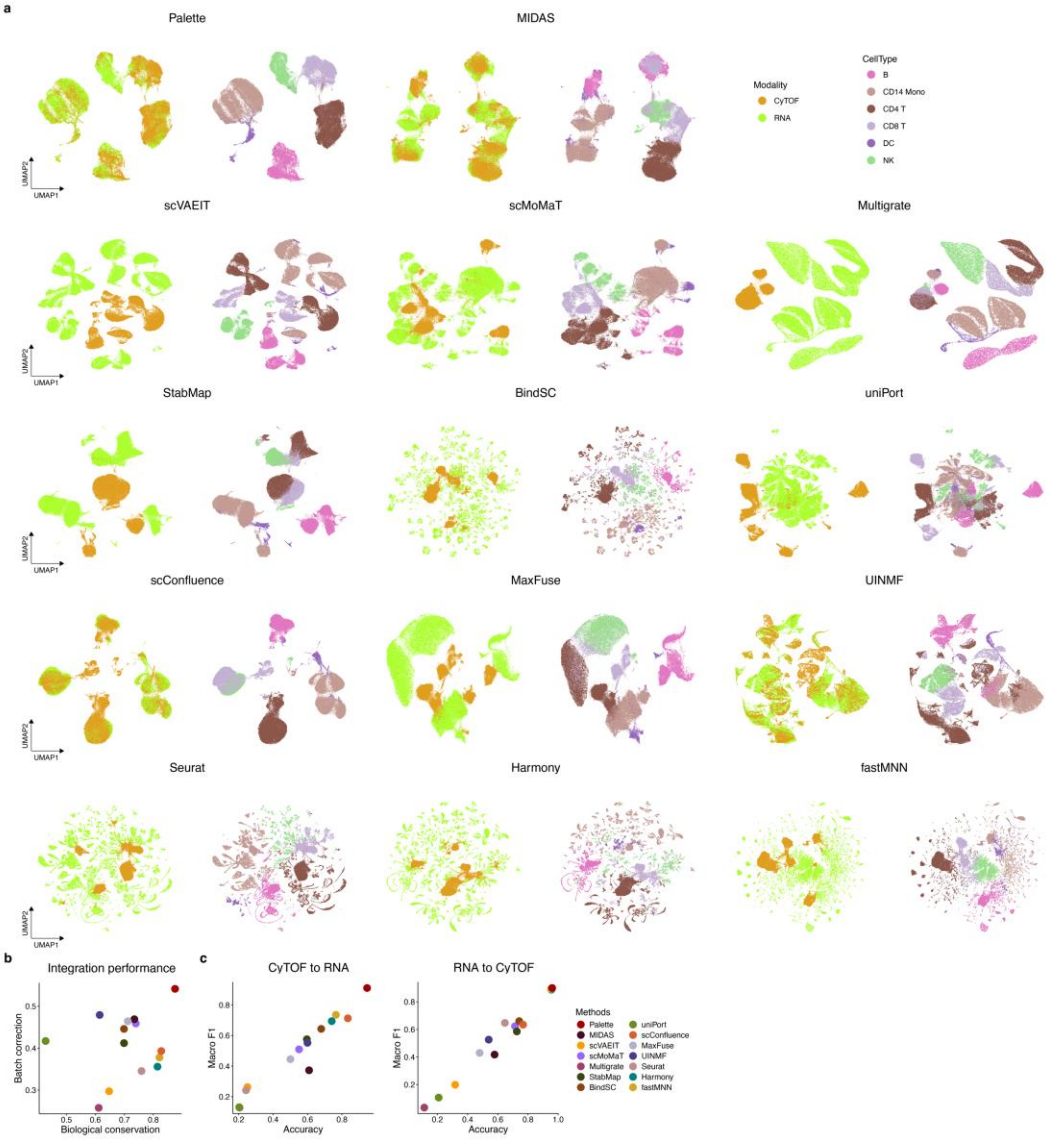

Supplementary Figure 32. Diagonal integration results for the human PBMC data. a. UMAP visualizations of integrated cell embeddings generated by Palette and 13 other integration methods. Cells are colored by modality and cell type labels. **b.** Scatter plot of biological conservation scores versus batch correction scores for different integration methods. **c.** Comparison of cross-modality label transfer performance across methods. Cell type labels from the proteome data were transferred to predict the identities of cells in the transcriptome data (left), and vice versa (right).

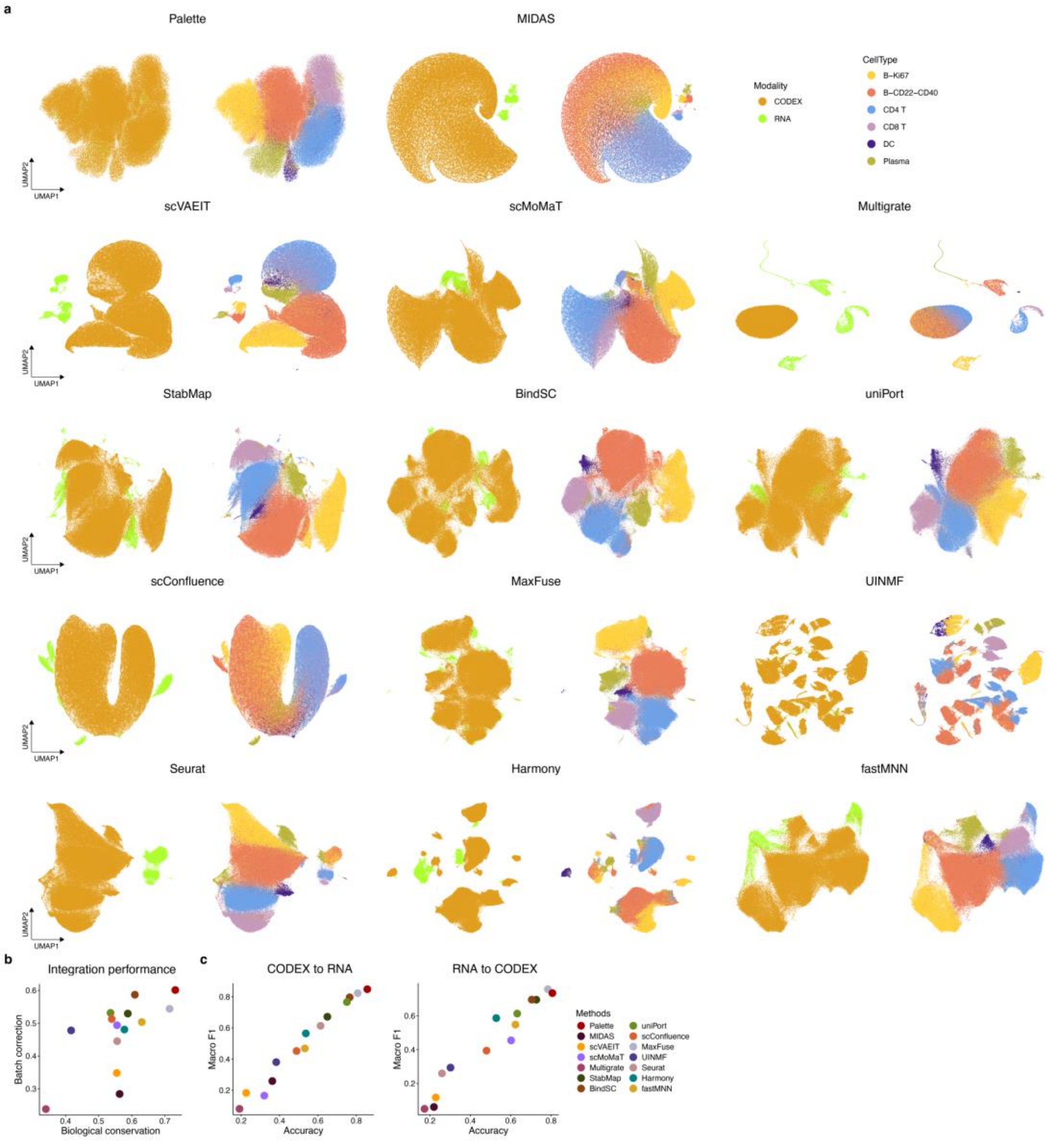

Supplementary Figure 33. Diagonal integration results for the human tonsil data. a. UMAP visualizations of integrated cell embeddings generated by Palette and 13 other integration methods. Cells are colored by modality and cell type labels. **b.** Scatter plot of biological conservation scores versus batch correction scores for different integration methods. **c.** Comparison of cross-modality label transfer performance across methods. Cell type labels from the proteome data were transferred to predict the identities of cells in the transcriptome data (left), and vice versa (right).

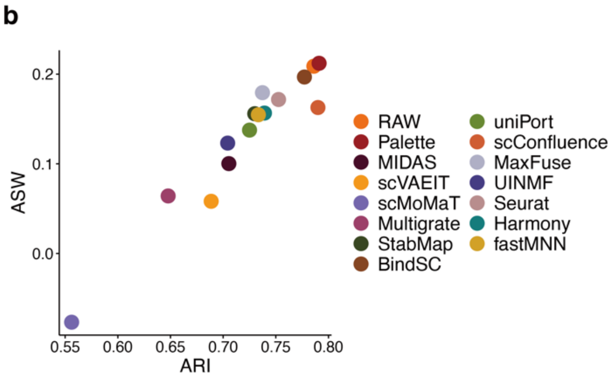

Supplementary Figure 36. Re-annotation of mouse MOp data based on Palette-integrated embeddings. b. Comparison of ARI and ASW across annotations generated by different integration methods and the original studies.

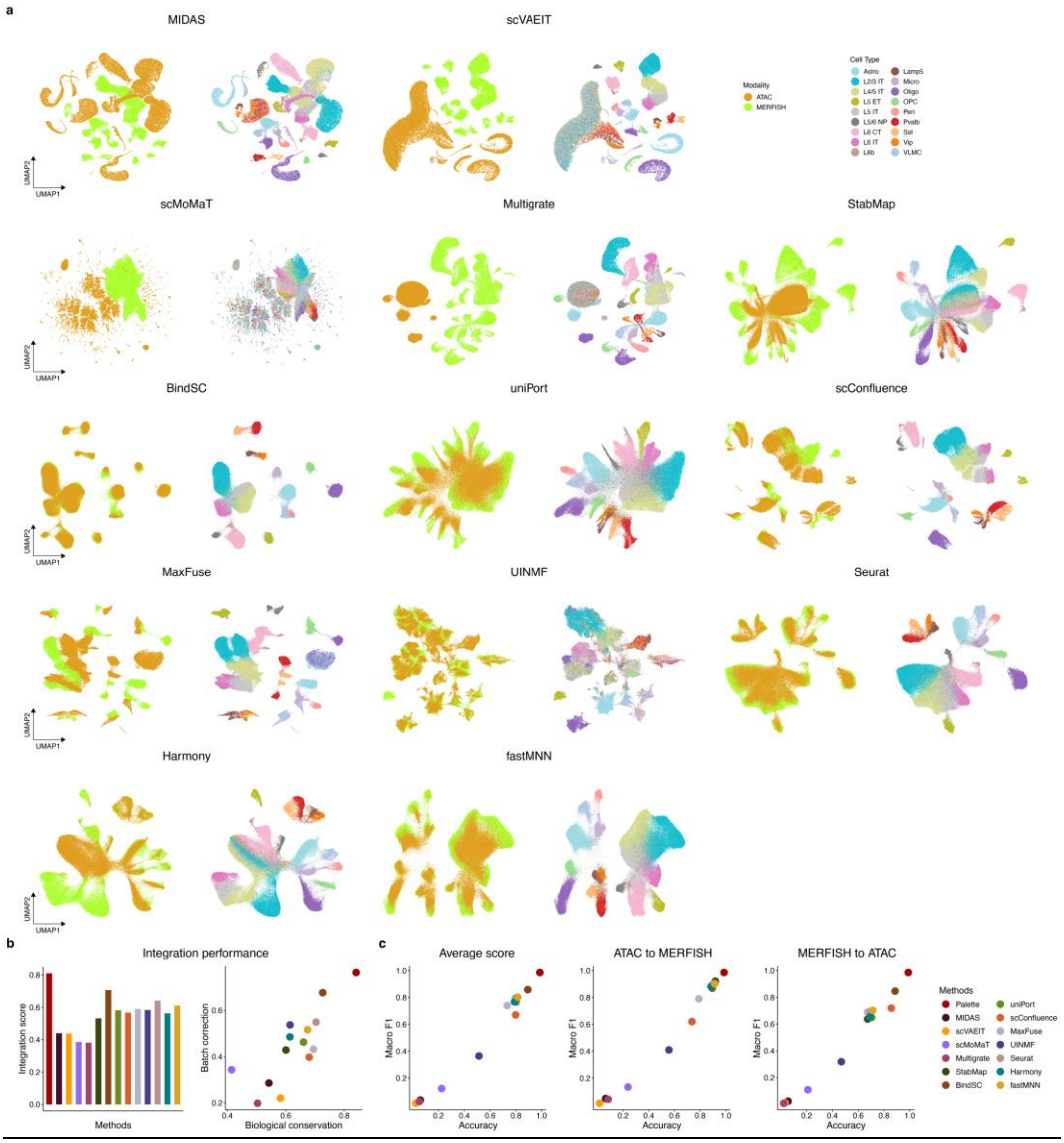

Supplementary Figure 37. Diagonal integration results for the mouse MOp data. a. UMAP visualizations of integrated cell embeddings generated by Palette and 13 other integration methods. Cells are colored by modality and Palette-based cell type labels. **b.** Comparison of integration performance across methods, with overall integration scores shown on the left, and biological conservation scores and batch correction scores on the right. **c.** Scatter plot of mean macro F1 scores versus mean accuracy scores for different integration methods. **d.** Comparison of cross-modality label transfer performance across methods. Cell type labels from the chromatin accessibility data were transferred to predict the identities of cells in the MERFISH data (left), and vice versa (right).

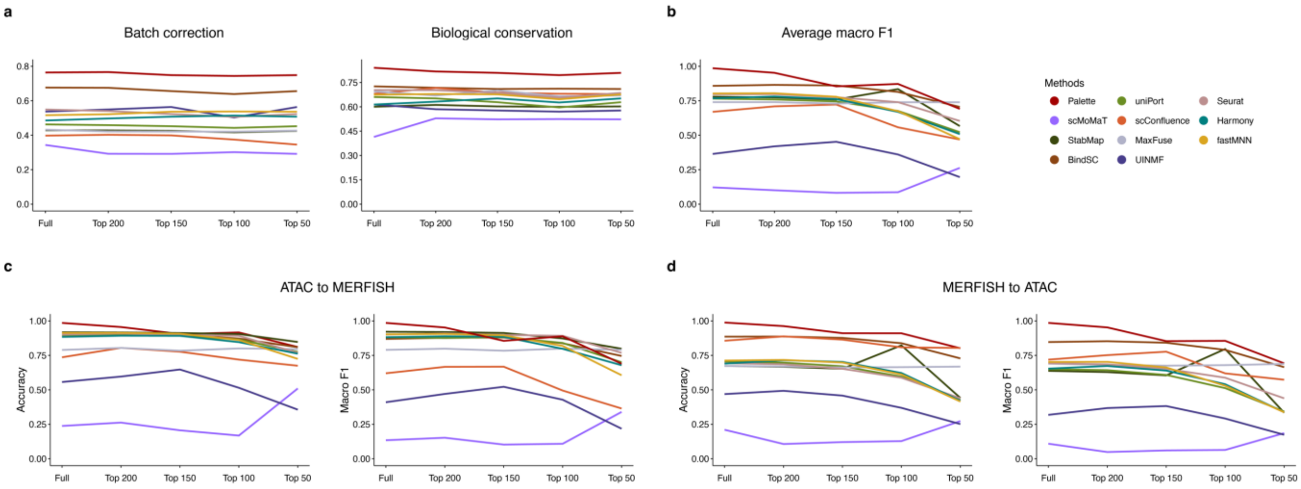

Supplementary Figure 38. Integration and label transfer performance of Palette and other methods on mouse MOp data with reduced feature overlap. a. Line plots showing batch correction (left) and biological conservation (right) scores for each method. **b.** Line plot of average macro F1 scores quantifying overall label transfer performance across methods. **c, d.** Line plots of cross-modality label transfer performance, evaluated using accuracy (left in **c** and **d**) and macro F1 (right in **c** and **d**). Cell type labels from scATAC-seq were transferred to MERFISH (**c**) and vice versa (**d**).

Modified Results:

Lines 371-383: We evaluated this strategy across three diagonal integration tasks, each involving a pair of unshared modalities from six datasets: (1) scRNA-seq and scATAC-seq data from human kidney[72], (2) scRNA-seq data[73] and cytometry by time of flight (CyTOF) proteomics data (30 markers)[74] from human PBMC, and (3) scRNA-seq data[75] and co-detection by indexing (CODEX) spatial proteomics data (46 markers)[15] from human tonsil. We benchmarked Palette against mosaic integration methods as well as six diagonal integration algorithms: (1) the general-purpose methods BindSC[76], uniPort[77], and scConfluence[78]; (2) GLUE[79] and SIMBA[80], which are designed for strongly linked modalities such as scRNA-seq and scATAC-seq; (3) and MaxFuse[70], which targets weakly linked modalities such as scRNA-seq and proteomic data. We also included three batch integration

methods (fastMNN, Seurat v3, and Harmony) and UINMF[81], which requires globally shared features. Integration performance was assessed by cross-modality label transfer accuracy and overall integration performance. Across all three tasks, Palette consistently outperformed other methods, achieving the highest performance (Fig. 5a and Supplementary Figs.31–33).

Lines 408-419: Finally, we collected scATAC-seq data[93] and spatially resolved multiplexed error-robust fluorescence in situ hybridization (MERFISH) data (254 genes)[94] from mouse MOp. Using the gene activity matrix from scATAC-seq and the corresponding expression profiles from MERFISH, we constructed pseudo-modality data comprising 253 linked features shared across both modalities. Palette successfully aligned the modalities, but slight discrepancies exist in the original cell type labels. To standardize labels, we computed silhouette widths (SWs) for MERFISH-profiled cells in the Palette-integrated embedding, treated cells with SW > 0 as high-confidence anchors, and assigned labels to the remaining cells using k-nearest neighbors (kNN) in the integrated embedding (Fig. 5f and Supplementary Fig. 36c). For comparison, we applied the same label assignment process to the other 13 algorithms. Across all methods, Palette-based reannotation achieved the highest adjusted Rand index (ARI) and average silhouette width (ASW) in the low-dimensional space of each modality, surpassing those of the original labels (Supplementary Fig. 36a, b).

Lines 425-431: To assess the robustness of Palette under conditions with limited linked features, we ranked the 253 linked features according to their importance for differentiating cell types (Methods). We then generated pseudo-modality data using the top 200, 150, 100, and 50 most informative genes, and applied 11 integration methods that rely on cross-modality feature linkage. Notably, Palette maintained superior performance across all feature sets, demonstrating robustness to the reduction in linked features (Fig. 5g and Supplementary Fig. 38).

Added References:

78. Samaran, J., Peyré, G. & Cantini, L. scConfluence: single-cell diagonal integration with regularized inverse optimal transport on weakly connected features. *Nat. Commun.* **15,** 7762 (2024).

79. Cao, Z. J. & Gao, G. Multi-omics single-cell data integration and regulatory inference with graph-linked embedding. *Nat. Biotechnol.* **40,** 1458–1466 (2022).

80. Chen, H., Ryu, J., Vinyard, M. E., Lerer, A. & Pinello, L. SIMBA: single-cell embedding along with features. *Nat. Methods* **21,** 1003–1013 (2023).

Comment: 4. The manuscript benchmarks Palette against methods like MIDAS and Multigrate in several scenarios (e.g., cross-species, spatial mosaic integration) for which these competitor tools were not originally designed. The authors must provide a detailed description in the Methods section of how

the preprocessing pipelines, input data structures, and even model architectures of these competitor algorithms were modified or extended to handle these complex tasks. Without these critical methodological details, the fairness and validity of the comparisons cannot be assessed, and the reliability of the results is questionable.

Response: We thank the reviewer for this valuable comment. In the revised manuscript, we added more methodological details for such methods in these integration scenarios. For the preprocessing pipelines, we have already described in the Methods section of the original manuscript (in 'Cross-species multimodal MOp dataset', 'Cross-species WAT scRNA-seq dataset', 'Human tonsil 10x Visium dataset', and 'Data pre-processing' subsection; lines 819-826, 832-837, 838-842, and 905-923 in the revised manuscript). Importantly, we did not modify the model architecture of any competing method. All methods were executed following the procedures of the respective original publications, which we described in the Methods section of the original manuscript (in 'Benchmark data integration methods' subsection; lines 938-963 in the revised manuscript). For input data structures, when a method did not natively support a particular modality configuration, we ran it under the closest supported configuration consistent with its intended use. These input modifications were introduced solely to enable the method to run without altering its algorithmic components.

We have added a detailed description of the method-specific input modifications for the cross-species MOp, cross-species WAT, and low-resolution spatial analyses to the **Methods** section in the revised manuscript (*lines 976-998*).

Added Methods (lines 976-998):

Cross-species multimodal MOp integration. We integrated cross-species MOp multimodal data using Palette and five competing methods under a unified preprocessing workflow. We did not modify the model architecture of any method. For methods that cannot jointly ingest multiple epigenomic modalities (MIDAS and scVAEIT), we provided an epigenomic representation in their closest supported configuration by using a gene activity matrix (generated using the GeneActivity function in the Signac R package (v1.9.0) with default parameters) as a proxy. In contrast, Palette, scMoMaT, Multigrate, and StabMap can accommodate arbitrary modality combinations and were provided the modality matrices in their native supported formats. No architecture modifications or method-specific hyperparameter tuning were introduced.

Cross-species WAT scRNA-seq integration. For tasks relying solely on orthologous genes, all methods received identical input matrices. For tasks incorporating non-orthologous genes, input configurations were adapted based on method capabilities. MIDAS, scVAEIT, and StabMap natively support features not shared across all batches. For these methods, non-orthologous genes were supplied as non-globally shared features within the scRNA-seq modality. Palette, scMoMaT, and Multigrate

typically expect globally shared feature sets within a modality. To incorporate non-orthologous genes without altering the source code, we supplied these genes as a separate, additional input modality. This strategy allows the utilization of species-specific information while adhering to the algorithmic constraints of the methods.

Low-resolution spatial multimodal integration. We treated low-resolution spatial mosaic datasets mathematically identically to single-cell mosaic data, with the primary distinction being resolution (spot vs. cell). No model architectures were modified. All methods received identically preprocessed data and identical feature sets. They were executed following the standard procedures described in their original publications, utilizing their native supported data formats.

Comment: 5. The claim of Palette's simplicity is undermined by its reliance on several key parameters that require manual tuning, particularly the modality-specific regularization parameter λ for Bi-sPCA. Although robustness analysis is provided, the manuscript lacks a clear explanation of how the default parameters were chosen and fails to offer practical guidance for users on how to select or tune these parameters for new datasets. This ambiguity limits the tool's ease of use and broad applicability.

Response: We thank the reviewer for this constructive comment. We agree that our previous description of Palette as “simple” may have been inappropriate. In response, we have removed the corresponding description from the revised manuscript.

Regarding key parameters in the Palette framework, we have added explicit explanations of the rationale behind the default parameter settings and provided practical guidance for selecting appropriate values when applying Palette to new single-cell modalities. These parameters include the quantile threshold for selecting representative cells, the thresholds T_1 and T_2 for identifying similar clusters across batches, and the Bi-sPCA regularization parameter λ used at different stages of the integration workflow. Specifically, we have described how the representative-cell quantile threshold trades off computational cost against robustness to noisy cells, how the cluster-matching thresholds T_1 and T_2 control the stringency of cross-batch cluster pairing in the unsupervised setting, and how the Bi-sPCA regularization parameter λ modulates the balance between suppressing unwanted technical variation and preserving biologically meaningful structure.

We have revised the description of Palette in the **Introduction** section of the revised manuscript (*lines 54-55*). Moreover, we have added explicit explanations of the role and practical considerations behind the default parameter settings used in this study, together with the guidance for adapting them to new datasets, in the **Supplementary Methods**.

Modified Introduction (lines 54-55):

Here, we present Palette, a flexible computational framework for mosaic integration that operates without relying on explicit biological assumptions.

Added Supplementary Methods:

An explanation of parameter selection in Palette. Palette integration involves several parameters that are specified at different stages of the workflow, including the quantile threshold for selecting representative cells, the thresholds T_1 and T_2 for identifying similar clusters across batches, and the Bi-sPCA regularization parameter λ used at different integration stages. Here, we explain the rationale behind the default settings of these parameters and provide practical guidance for selecting appropriate values when applying Palette to new data modalities.

Quantile threshold for representative cell selection. The quantile threshold determines the proportion of representative cells selected within each cell type or cluster, based on silhouette width (SW) rankings as described earlier. Higher quantile thresholds correspond to selecting fewer cells. This design serves two purposes: it reduces computational complexity and improves robustness to noisy or potentially mislabeled cells. For modalities where measurements are sparse and cell–cell separability is weak, using a higher quantile threshold (i.e., retaining fewer, more representative cells) can reduce the influence of ambiguous observations on downstream similarity estimation. For large-scale datasets, higher thresholds can also reduce runtime.

Global modality-specific thresholds T_1 and T_2. The thresholds T_1 and T_2 are jointly used to filter similar clusters across batches in the unsupervised setting. Specifically, T_1 acts as a global, modality-specific lower bound on cluster similarity, whereas T_2 is the cosine value of an adaptive angular constraint that accounts for within-cluster variability through the combination of the median cluster angle θ_c and a modality-specific offset θ_m. A cluster-pair similarity is retained only if it exceeds $T = \max\{T_1, T_2\}$. These two thresholds play complementary roles: T_1 controls a global similarity baseline across batches, while T_2 adjusts the similarity requirement according to the intrinsic structure of each cluster. In practice, when within-dataset separability and cross-batch consistency are strong, users may consider slightly more stringent settings (higher T_1, smaller θ_m); conversely, when signals are weak or sparse, more permissive settings can help avoid discarding potentially related clusters.

Bi-sPCA regularization parameter λ. The Bi-sPCA framework includes a regularization parameter that controls the balance between suppressing unwanted technical variation and preserving biologically meaningful signals. In Palette, λ is used in two distinct contexts.

During the joint dimensionality reduction stage, λ^m is specified in a modality-specific manner to account for differences in dimensionality and sparsity across modalities. Modalities with relatively dense feature-by-cell matrices and strong cell–cell separability, such as scRNA-seq and ADT, often tolerate moderately larger λ^m, whereas sparse modalities, such as scATAC-seq, may require more

conservative settings to reduce the risk of over-correction. Pseudo-modality matrices may also require slightly stronger regularization in some settings, and we therefore recommend starting from a conservative baseline and refining if needed.

In the subsequent cross-batch alignment stage, a single global λ parameter is used to regulate the suppression of batch-associated variation across modalities. While this parameter serves a different role from λ^m, its selection follows a similar principle: stronger regularization is appropriate when technical heterogeneity across batches is pronounced, while monitoring potential loss of biological structure.

Practical guidance for parameter selection on new modalities. While Palette provides robust default parameters for established modalities (ADT, scRNA-seq, and scATAC-seq), which were used for all benchmarks in this study, users may encounter novel or domain-specific modalities. When applying Palette to a new modality, we recommend a principled "warm-start then refine" strategy. If the new modality shares similar characteristics, including signal-to-noise ratio, dimensionality, and sparsity patterns, with an established modality, its default parameters should serve as the initial baseline. Otherwise, we suggest the following systematic procedure.

Quantile threshold for representative cell selection. In most cases, the default quantile threshold for representative cell selection is sufficient. For extremely large datasets or modalities with very sparse measurements and weak separability, this threshold can be moderately increased to stabilize representative cell selection.

Global modality-specific thresholds T_1 and T_2. If no established modality defaults are applicable, we recommend using the pseudo-modality defaults (described in the Methods). This configuration provides a permissive filtering regime intended to reduce the risk of over-filtering potentially informative cross-batch similarity signals for integration. Note that these parameters are effectively bypassed in supervised integration modes.

Bi-sPCA regularization parameter λ. If cell-type labels are available, tuning should be guided by the interplay between batch correction and biological conservation metrics (both described in the Methods). An observation of high batch correction scores coupled with low biological conservation may suggest over-correction, necessitating a decrease in λ^m. Conversely, persistent batch separation despite preserved biological structure suggests under-correction, warranting an increase in λ^m. In the absence of labels, we recommend using batch-wise clustering consistency, such as mean ASW for each batch on the integrated embedding, as a proxy for biological conservation.

Finally, for the global cross-batch alignment parameter λ, while the default value is designed to be conservative and robust, it can be further refined following the same dual-criterion principle as λ^m, balancing alignment strength against biological conservation. When labels are available, users can

optimize λ by monitoring the modality mixing score (as defined in the Results) instead of the batch mixing score. Otherwise, the default setting generally provides a robust and conservative choice.

Comment: 6. The manuscript does not systematically evaluate Palette's performance on cross-modal prediction (i.e., modality translation) or data imputation, which are key capabilities of many modern multimodal integration methods. A lack of direct comparison with SOTA methods on these tasks makes it difficult for readers to fully assess the functional scope and relative advantages of Palette.

Response: We thank the reviewer for this constructive comment. To assess the effectiveness of Palette's predicting (imputing) missing modalities, we have conducted a systematic evaluation of Palette's modality inference performance. Specifically, we benchmarked Palette on five benchmark tasks that support missing modality prediction and compared its performance with three state-of-the-art methods: MIDAS, scVAEIT, and Multigrate. Following a recent comprehensive benchmark study (added Supplementary ref. 12), we evaluated inference quality using six established metrics that capture both data structure preservation and clustering consistency.

Across all evaluated tasks, Palette successfully reconstructed missing modalities and demonstrated competitive and stable performance relative to existing methods (Supplementary Figs. 44 and 45). Detailed experimental setups, evaluation protocols, and metric definitions have provided in **Supplementary Note 4** and **Supplementary Methods** of the revised Supplementary Information. These results demonstrate that Palette can provide accurate and reliable missing modality prediction across diverse multimodal settings.

Added Supplementary Figures:

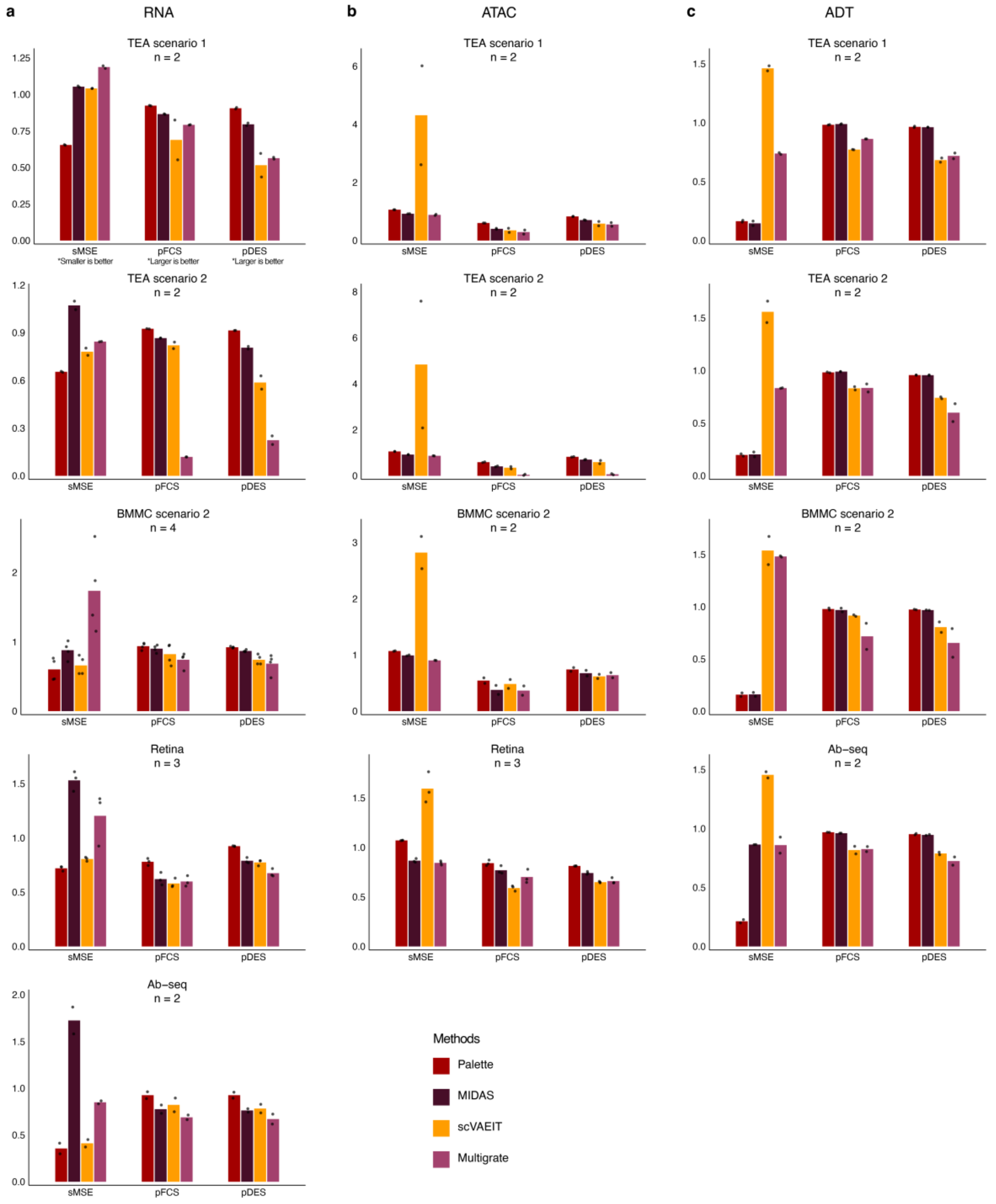

Supplementary Figure 44. Quantitative evaluation of inferred modality data using data structure metrics. Across five integration scenarios, we compared the modality inference performance of Palette with MIDAS, scVAEIT, and Multigrate using data structure metrics, including sMSE, pFCS, and pDES. For each task and each metric, bars indicate the mean performance across inferred batches, and individual points represent batch-level measurements. In addition, the number annotated in each panel denotes the number of batches inferred for the corresponding modality and task.

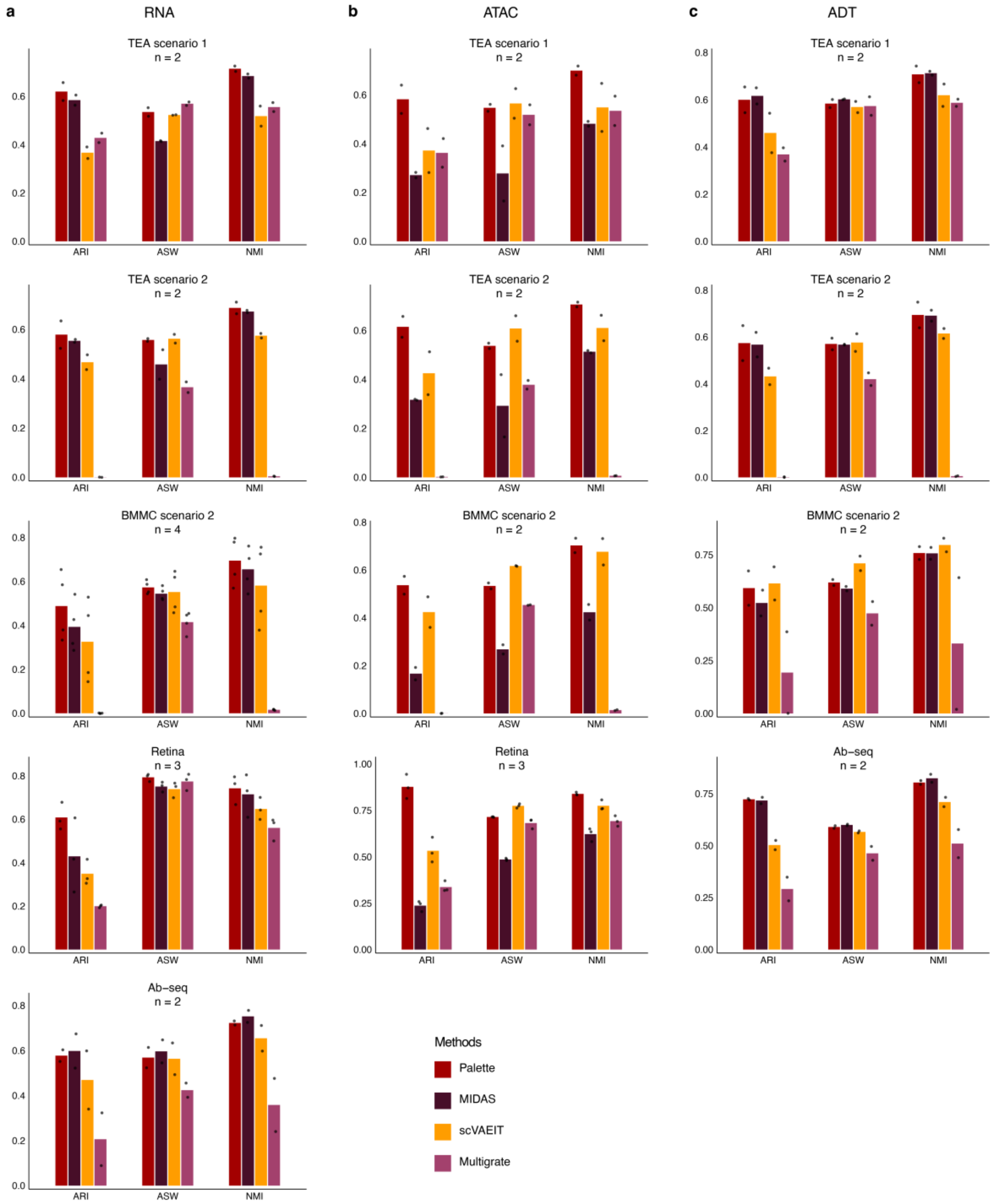

Supplementary Figure 45. Quantitative evaluation of inferred modality data using clustering-based metrics. Across five integration scenarios, we assessed modality inference performance for Palette, MIDAS, scVAEIT, and Multigrate using clustering-based metrics, including ARI, ASW, and NMI. For each task and each metric, bars indicate the mean performance across inferred batches, and individual points represent batch-level measurements. In addition, the number annotated in each panel denotes the number of batches inferred for the corresponding modality and task.

Added Supplementary Note:

Supplementary Note 4. Benchmarking modality inference performance

We evaluated missing-modality inference using five benchmark scenarios. BMMC Scenario 1 was excluded because the missing modality lacks real measurements and therefore cannot be quantitatively assessed. For each remaining scenario, one sub-experiment was selected for inference (Supplementary Methods). Palette was compared with three methods capable of performing missing-modality inference: MIDAS, Multigrate, and scVAEIT.

Inference performance was assessed in a modality-specific manner within each task using data structure and clustering-based metrics following a recent benchmark study[12] (Supplementary Methods). Across all scenarios and nearly all metrics, Palette either achieved the highest performance scores or exhibited performance comparable to the top-performing methods. An exception was observed for the standardized mean squared error (sMSE) metric on scATAC-seq data, where Palette did not rank among the top-performing methods (Supplementary Figs. 44 and 45). Overall, these results indicate that Palette enables accurate and reliable missing modality inference across diverse multimodal settings.

Added Supplementary Methods:

Modality inference analysis. We conducted missing-modality inference experiments using five of the six benchmark tasks that support this evaluation. For each task, one sub-experiment was randomly selected for inference (TEA Scenario 1: 2; TEA Scenario 2: 3; BMMC Scenario 2: 1; Retina: 3; Ab-seq: 4). Palette was compared with three methods designed for missing-modality inference: MIDAS, scVAEIT, and Multigrate. To ensure consistency across inferred outputs, we used the top 50,000 highly variable scATAC-seq peaks as input for all tasks except Ab-seq. For a given modality within a task, inference was performed across multiple batches. Performance was first evaluated separately for each batch using the corresponding metrics, and batch-level values were then averaged to obtain a single modality-level performance score used for comparison across methods and tasks.

Modality inference metrics. We quantitatively evaluated modality inference performance using the evaluation metrics described by Liu et al.[12], which cover both data structure metrics and clustering-based evaluation metrics. The data structure metrics include sMSE relative to the ground-truth data, preservation of feature correlation structure (pFCS), and preservation of differential expression statistics (pDES).

sMSE. We computed the standardized mean squared error between the inferred and ground-truth matrices, with lower values indicating closer agreement.

pFCS. For the inferred and ground-truth data, we independently selected highly variable features (100 for scRNA-seq and ADT, and 1,000 for scATAC-seq). Using each dataset's own feature set, we

computed cell–cell correlation matrices and quantified preservation as the mean Pearson correlation between the two matrices.

pDES. For each cell type, DE analysis was performed separately on the inferred and ground-truth data using limma (v3.54.2) to obtain DE feature sets. Cell–cell correlation matrices were then computed from each dataset using its corresponding DE features, and preservation was measured as the mean Pearson correlation between the two matrices.

For clustering-based evaluation, we used ARI, ASW, and NMI, as introduced earlier (Methods), to quantify the agreement between the Louvain-derived cluster labels and the ground-truth labels.

Added Supplementary Reference:

12. Liu, C., Ding, S., Kim, H.J. et al. Multitask benchmarking of single-cell multimodal omics integration methods. *Nat Methods* **22**, 2449–2460 (2025).

Comment: 7. It is unclear whether the method can output a corrected data matrix in the original feature space, analogous to the IntegrateData function in Seurat. Such a feature is crucial for many downstream analyses (e.g., differential expression, trajectory inference) and for ensuring compatibility with other single-cell analytical toolkits. If Palette's integration is confined to the low-dimensional embedding space, this would significantly limit its versatility and utility in broader biological analysis workflows.

Response: We thank the reviewer for this thoughtful comment. Palette does not output a globally corrected expression matrix in the original feature space. In practice, mosaic multimodal integration methods primarily rely on a shared latent representation or a neighborhood graph for downstream analyses, rather than on corrected expression matrices. In mosaic integration settings, different batches observe different combinations of modalities and therefore do not share a globally consistent feature space at the measurement level. Constructing a single "global" corrected expression matrix would require additionally imputing missing modalities across batches. For this reason, corrected expression matrices are generally of limited practical utility for downstream biological analyses in mosaic settings.

This design choice is consistent with other mosaic integration methods, such as scMoMaT and StabMap, which similarly operate at the latent or embedding level rather than producing globally corrected expression matrices. The shared representation learned by Palette supports the core downstream analyses typically performed in integrative single-cell studies, including joint clustering, visualization, cell type annotation, neighborhood-based analyses, trajectory inference, and cross-dataset comparison.

For differential expression analysis, consistent with current best practices[1,2], differential testing is typically performed on the original (raw or normalized) data rather than on corrected matrices, which are neither intended nor required for differential testing.

We have clarified the output of Palette in the **Methods** section of the revised manuscript (*lines 495–497*) to explicitly state that Palette learns a shared latent representation rather than producing a globally corrected expression matrix.

References:

1. Luecken, M. D. & Theis, F. J. Current best practices in single-cell RNA-seq analysis: a tutorial. *Mol. Syst. Biol.* **15**, e8746 (2019).
2. Nguyen, H.C.T., Baik, B., Yoon, S. et al. Benchmarking integration of single-cell differential expression. *Nat Commun.* **14,** 1570 (2023).

Modified Methods (lines 490-497):

Overview. Palette takes as input appropriately preprocessed mosaic datasets with diverse modality compositions across batches, accompanied by batch annotations and, optionally, cell type annotations for each cell. The mosaic integration workflow includes three key steps: (1) intra-modal joint dimensionality reduction, (2) MBG-guided inferring of missing modality matrices, and (3) cross-batch alignment. Moreover, Palette enables reference-based integration, allowing newly acquired mosaic data to align with an already integrated multimodal reference. As output, Palette learns a shared latent representation across batches and modalities, rather than a globally corrected expression matrix in the original feature space.

Comment: 8. The manuscript needs to clarify how Palette handles inconsistent features within the same modality across different batches (e.g., missing proteins or genes due to different antibody panels or gene annotations). If the approach relies on a simple feature intersection, the number of shared features could decrease dramatically as more datasets are integrated, leading to significant information loss and potentially compromising integration performance. Does Palette employ a more sophisticated strategy to address this common issue?

Response: We thank the reviewer for this thoughtful comment. Palette generally requires a common feature space within a modality by intersecting the features observed across batches. However, for inconsistent features within a modality, Palette can incorporate them by setting these inconsistent feature matrices as new modality matrices. In the original manuscript, we demonstrated how to include non-globally shared features in the cross-species white adipose tissue analysis. Specifically, we treat orthologous features (globally shared features) as a shared modality and non-orthologous features (non-globally shared features) as species-specific modalities, constructing a mosaic integration task (Fig. 4i and j, and Supplementary Figs. 27 and 28 in the revised manuscript). The results illustrated that, compared to integration solely using orthologous features, Palette can benefit from incorporating non-orthologous features.

We have added clarifications in the **Methods** section of the revised manuscript (*lines 525–529*) to describe how heterogeneous feature sets are handled in Palette.

Modified Methods (lines 525-529):

Intra-modal joint dimensionality reduction. Palette first performs dimensionality reduction independently for each modality on the modality-specific common feature space. When non-globally shared features correspond to distinct biological measurements, Palette allows them to be modeled separately as additional modalities, rather than being forced into the intersected feature space.

Comment: 9. In the reference-based integration strategy, the method appears to use only a subset of the reference atlas—specifically, batches with modality compositions matching the query—as a 'bridge' (as illustrated in Supplementary Fig. 1). This approach may discard a substantial amount of valuable information present in other batches of the reference, potentially reducing the accuracy and robustness of the alignment. The authors should justify this design choice or compare its performance against methods that leverage the entire reference atlas.

Response: We thank the reviewer for this thoughtful comment. This design choice is imposed to ensure that harmonization is carried out only between datasets that are comparable in terms of available modalities. Performing harmonization between the query and reference batches containing modalities absent from the query would be ill-defined and may introduce spurious corrections driven by modality-specific signals that the query does not possess. Importantly, this design choice does not imply that other reference batches are excluded from the integration. After harmonization using the modality-matched reference subset, the query is further aligned and embedded into the latent space learned from the entire reference atlas, allowing it to leverage the global biological structure captured across batches and modalities.

Additionally, we compared Palette performance against methods that utilize the entire reference atlas. Specifically, two methods, MIDAS and Multigrate, are incorporated into comparing under multiple scenarios described in the original manuscript (lines 150-153 in the revised manuscript). Palette consistently achieved superior integration quality and label transfer accuracy (Supplementary Fig. 19), demonstrating the effectiveness of the reference-based integration strategy for Palette.

We have modified both the **Results** and **Discussion** sections in the revised manuscript (*lines 159-166 and 469-475*) to update the experimental result and clarify the design rationale of the reference-based integration strategy.

Modified Supplementary Figure:

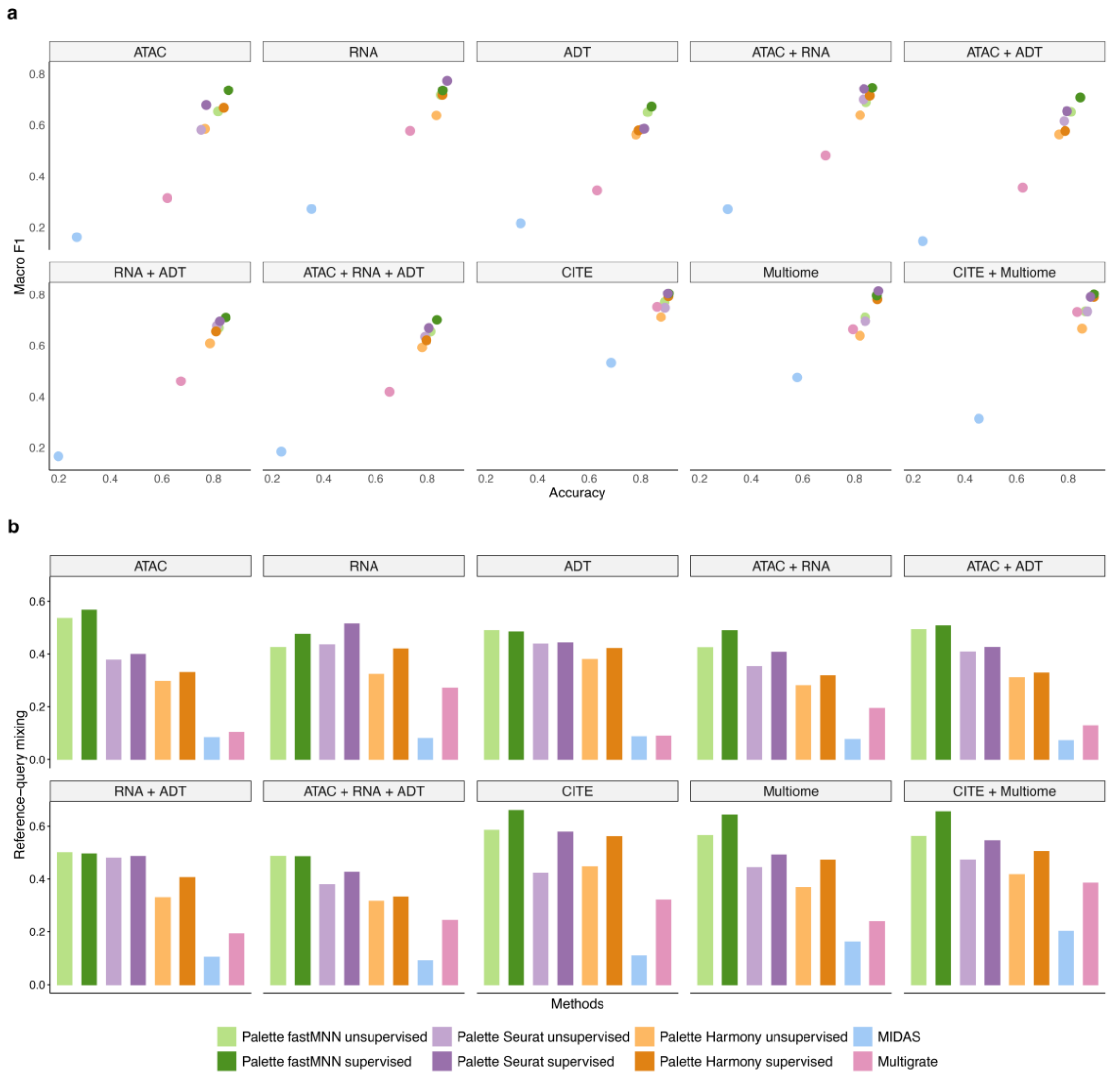

Supplementary Figure 19. Evaluation of cell type label transfer and dataset mixing in reference-based integration across multiple query scenarios. a. Scatter plots showing label transfer performance for each query scenario, measured by accuracy (x-axis) and macro F1 score (y-axis). **b.** Bar plots showing the mixing scores between reference and query cells for each query scenario. In the Palette reference-based integration framework, we incorporated three integration methods (fastMNN, Seurat v3, and Harmony), using fastMNN as the default. Reference embeddings were generated in either unsupervised or supervised mode.

Modified Results (lines 159-166):

We compared the performance of Palette with MIDAS and Multigrate. Quantitative results demonstrate that Palette consistently outperformed MIDAS and Multigrate in label transfer accuracy (Supplementary Fig. 19a), particularly excelling in scenarios involving novel modality compositions in the query data, an area where MIDAS and Multigrate showed performance degradation. In contrast, Palette demonstrated stable and high performance across different modality compositions.

Quantitative analysis of reference-query mixing further confirmed Palette's superiority (Supplementary Fig. 19b).

Modified Discussion (lines 469-475):

For scenarios involving integrated reference and unintegrated query, Palette exploits shared modalities as bridges to perform reference-based integration. This approach prevents modality composition imbalance between the reference and query datasets, ensuring alignment in a comparable feature space. Through the shared bridge modality, Palette maps the query data into the space of the globally integrated reference, allowing it to inherit the biological structure captured across batches and modalities. In addition, the strategy progressively incorporates query datasets into the reference, effectively constructing an expanded embedding.

Comment: 10. The theoretical novelty of the core components, Bi-sPCA and the MBG-guided propagation, needs to be more clearly articulated. The authors should position Bi-sPCA more thoroughly within the context of related dimensionality reduction techniques in machine learning (e.g., variants of CCA, dual-view PCA, or other multi-view learning methods). Similarly, the MBG propagation strategy bears conceptual resemblance to other graph-based methods like StabMap. A more detailed discussion is needed to highlight the unique aspects and advantages of Palette's graph construction and information propagation mechanism compared to existing approaches, thereby clarifying its methodological innovation.

Response: We thank the reviewer for this thoughtful comment. Bi-sPCA is formulated to operate on a unified data representation and to regulate dependence on two external variables through kernelized supervision. In contrast, CCA-based approaches aim to maximize correlation between multiple data views treated symmetrically. Moreover, joint PCA-based methods focus on capturing shared variance across views. The objective of Bi-sPCA is to modulate the influence of biologically meaningful and unwanted sources of variation within a single dimensionality reduction framework, rather than align views or model joint variance, which are the objectives of CCA-based or PCA-based methods, respectively.

For the MBG-guided propagation strategy, although both Palette and StabMap exploit mosaic data structure for mosaic integration, they differ fundamentally in graph construction and algorithmic role. Briefly, StabMap relies on a weighted batch–batch graph to guide reference-based mapping along predefined paths. In contrast, Palette constructs a mosaic bipartite graph whose nodes represent both batches and modalities. This design enables Palette to perform adaptive, path-conditioned, cell-level propagation and infer missing modality representations, rather than adhering to a fixed, batch-level mapping strategy.

We have added more details to the **Supplementary Methods** section in the revised Supplementary Information to clarify the novelty and advantages of the two core components of Palette.

Added Supplementary Methods:

Comparison between Bi-sPCA and existing dimensionality reduction methods. Bi-sPCA is designed to address dimensionality reduction problems in which learned representations are expected to be associated with a variable of interest while being as independent as possible of an unwanted source of variation. The multi-view learning methods, such as canonical correlation analysis (CCA) and its variants, aim to identify projections that maximize correlation between two data views, typically treating the views symmetrically. In contrast, Bi-sPCA operates on a single data matrix and uses kernelized supervision to regulate dependence on multiple external variables. Its objective is not to align two views, but to learn the low-dimensional representation with respect to predefined factors.

Bi-sPCA is also distinct from multi-view or joint PCA approaches, which seek to learn a shared low-dimensional subspace across multiple views by minimizing reconstruction error or enforcing low-rank structure. Such methods primarily focus on capturing shared variance across views and do not explicitly model competing sources of dependence. By contrast, Bi-sPCA is formulated to disentangle variation associated with different external variables, which act on the same data matrix, making it particularly suitable for integration scenarios where supervision and confounder control are both required.

Comparison between the MBG-guided propagation strategy and StabMap's strategy. The MBG-guided propagation strategy in Palette and the approach used in StabMap both exploit predefined mosaic data structures to guide integration, but they differ fundamentally in graph construction and algorithmic role.

Specifically, StabMap relies on a Mosaic Data Topology (MDT), a weighted graph whose nodes correspond to batches and whose edge weights are determined by the number of shared features between batches. StabMap integration is reference-based: one or multiple reference batches are specified, and all other batches are mapped to the reference embedding along paths determined by MDT edge weights, resulting in a fixed, batch-level mapping strategy. In contrast, Palette constructs an unweighted Mosaic Bipartite Graph (MBG) whose nodes represent both batches and modalities, with edges encoding only the presence of a modality in a batch. The MBG does not impose a reference batch or a predefined mapping order. Instead, it defines admissible paths for information transfer and is used to infer low-dimensional representations of missing modalities. Propagation in Palette is path-conditioned and cell-specific, with optimal paths selected dynamically based on nearest-neighbor distances and multiple candidate paths fused through distance-based weighting.

Thus, although both MDT and MBG encode mosaic data structure, MDT in StabMap guides reference-based batch mapping through weighted batch–batch relationships, whereas MBG in Palette serves as a structural prior for adaptive, path-conditioned inference of missing modalities. The MDT- and MBG-based strategies address distinct integration objectives and are not interchangeable.

Comment: 11. The fairness of the benchmarking is a concern. The Methods section states that for some competitor methods (e.g., Multigrate, scMoMaT), a reduced number of features (e.g., top 50,000 peaks for scATAC-seq) was used due to memory constraints. The authors must explicitly clarify whether Palette was also restricted to the exact same feature subset in these comparisons. If Palette utilized more input information than its competitors, the performance gains may be partially attributable to this discrepancy, rendering the comparison unfair.

Response: We thank the reviewer for this thoughtful comment. In the revised manuscript, we performed an additional, conservative analysis in which Palette was constrained to use the minimum number of feature sets used by any method within each evaluation scenario. This limited feature size setting places Palette under the most restrictive input condition while leaving all other components of the integration pipeline unchanged. Across both mosaic integration and diagonal integration tasks, Palette consistently remained the top-performing method under this constraint (Supplementary Figs. 48 and 49).

Added Supplementary Figures:

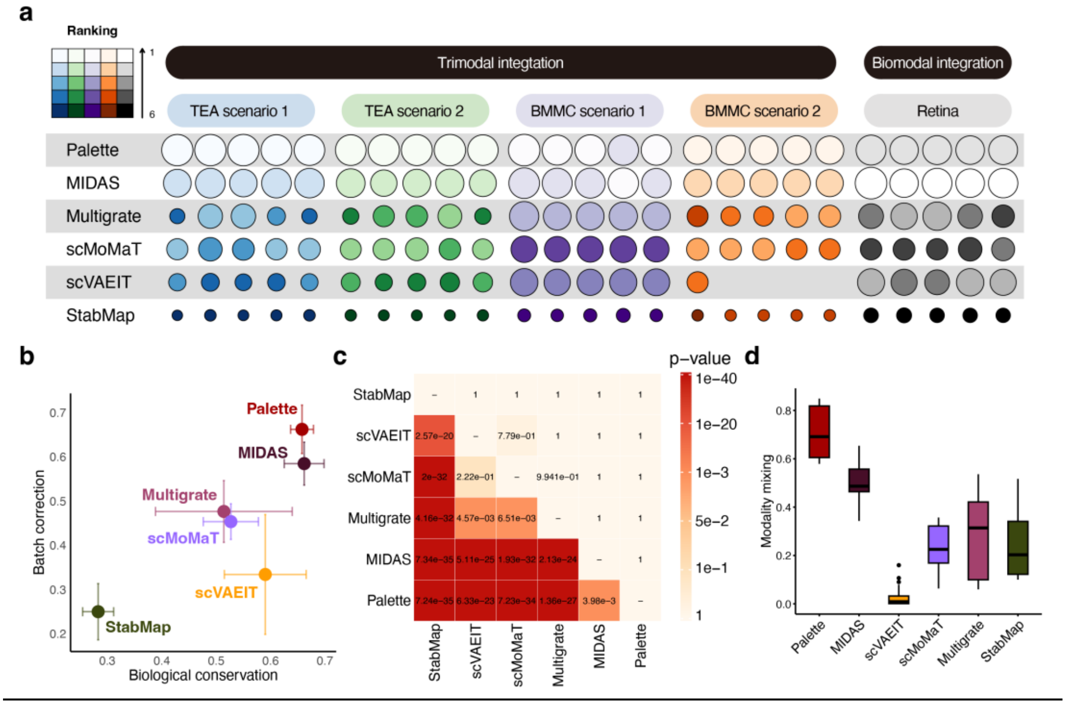

Supplementary Figure 48. Systematic benchmarking of Palette under limited feature size settings. a. Integration ranking across methods for each sub-experiment. The radius of each circle indicates the overall integration scores, ranging from 0 to 1. **b.** Scatter plot of mean biological conservation scores versus mean batch-correction scores for all integration methods. Error bars represent the standard error across the tasks evaluated for each method. **c.** Heatmap of one-sided paired Wilcoxon signed-rank test p-values. **d**. Box plot of overall modality mixing scores across methods. The central lines mark the median values, the boxes show the quartiles, and the whiskers show the rest of the distribution. In this benchmark, Palette was configured to use the minimum number of features required by any method within each scenario. In addition, scVAEIT failed to complete four sub-experiments in the BMMC scenario 2 due to training stagnation. For this method, means, medians, standard errors, and p-values were calculated based on the remaining 21 completed sub-experiments.

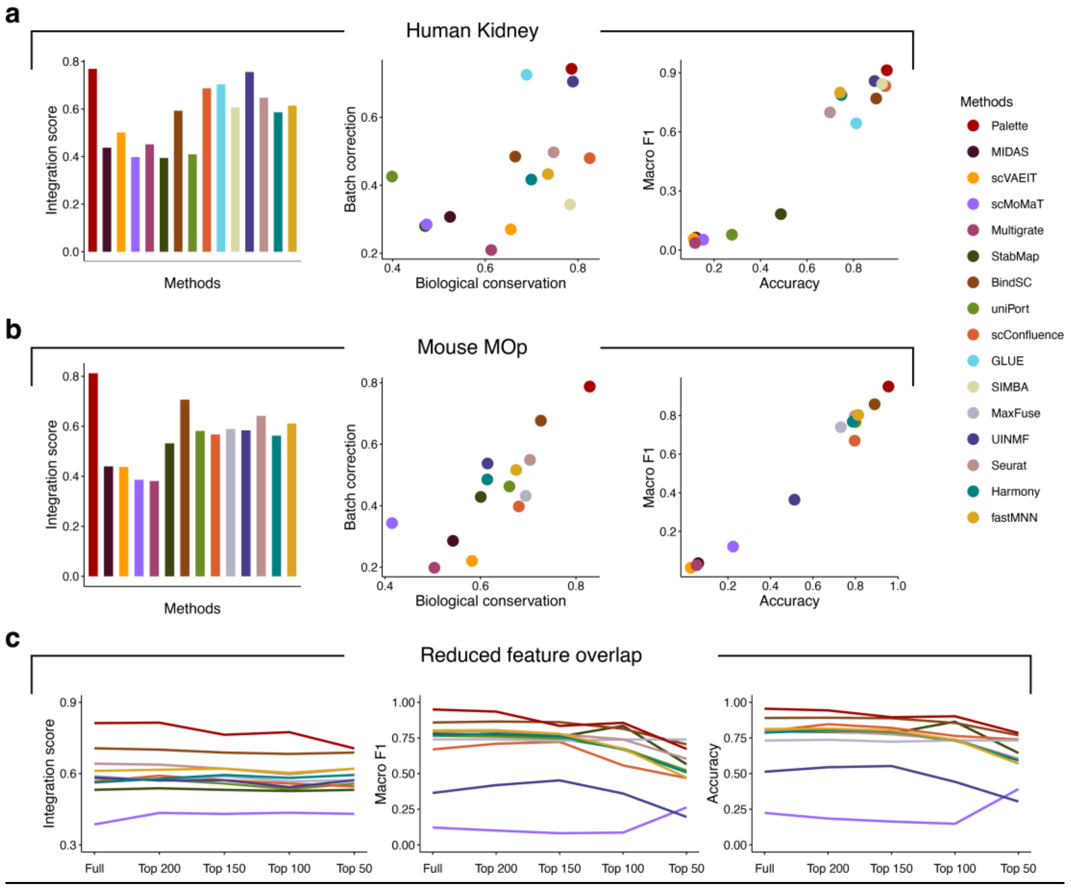

Supplementary Figure 49. Diagonal integration results of Palette under limited feature size settings. a, b. Comparison of integration and label transfer performance on human kidney (a) and mouse MOp (**b**) datasets across multiple evaluation metrics. **c.** Line plots comparing integration and label transfer performance for different integration methods under reduced feature overlap. In all experiments shown in this figure, Palette was configured to use the minimum number of features required by any method within each scenario.

Added Methods (lines 958-960):

Moreover, we evaluated Palette under a limited feature size setting, in which the number of input features was restricted to the minimum used within each evaluation scenario (Supplementary Figs. 48 and 49).

Comment: (Remarks on code availability) To improve reproducibility, please explicitly mapping each analysis in the paper to its corresponding code and parameter settings in the GitHub repository.

Response: We thank the reviewer for this thoughtful comment. In response, we have updated the reproducibility repository to explicitly document the correspondence between the analyses reported in the manuscript and the released code. Specifically, the README file now provides an experiment-level mapping in which each major analysis (corresponding to a main or supplementary figure in the paper) is linked to a dedicated folder containing the scripts required to reproduce the reported results. Within each analysis-specific folder, the scripts used to generate the results are provided, and all parameter settings used in the paper are explicitly defined within the corresponding scripts, consistent with those described in the Methods section of the manuscript. The reproducibility repository is available at: https://github.com/qiongyusheng/Palette_reproducibility.

Point-by-point responses to the comments of Reviewer #2

Comment: The paper presents Palette, a computational framework for mosaic integration of single-cell multimodal data. Mosaic integration aims to combine datasets that measure different molecular features — such as RNA, proteins, or chromatin accessibility — often from different cells or experiments, to build a unified representation of the cell population. Palette achieves this by using a modified form of principal component analysis and a mosaic bipartite graph, a network that links cells across different data types based on their similarity. Together, this approach aims to separate technical noise from biological variation, allowing information to be shared across missing modalities.

The benchmark on integration performance conducted on bimodal and trimodal datasets shows that Palette outperforms state-of-the-art methods in both biological signal preservation and batch correction. The study encompasses a wide range of datasets, modalities, and technologies. The authors provide also valuable insights into cross-species integration. The work also contributes a benchmarking resource, comparing across integration scenarios and demonstrating robust handling of batch effects beyond cross-modality alignment.

While we appreciate the value of this work, we believe some aspects of the evaluation strategy were not fully convincing.

Response: We thank the reviewer for the thoughtful comments and constructive suggestions regarding our work. In response, we have carefully revised the manuscript to address all the points raised. In particular, to strengthen the rigor and credibility of the evaluation strategy, we revised the evaluation metrics and expanded the evaluation scenarios to assess robustness and practical applicability better. The major revisions prompted by the reviewer's comments are summarized below.

(1) We have evaluated the performance of the supervised mosaic integration for Palette under multiple perturbed annotation settings.

(2) We have revised the modality mixing metrics and explicitly clarified their applicability boundaries. Additionally, a robustness and sensitivity analysis of the revised modality mixing metrics has been added.

(3) We have added a single-modality batch integration benchmarking using multiple scRNA-seq datasets.

(4) We have revised the manuscript to clarify the scope of the differentially expressed features in the cross-condition human PBMC data analysis and the integration mode in the spatial multimodal data mosaic integration.

(5) We have resolved the reported R package installation issues, expanded the reproducibility codebase, and ensured that the datasets used in this study are publicly accessible.

For more details, please see our point-by-point response below. For the reviewer's convenience, the corresponding revised manuscript is attached at the end of each response and is underlined to facilitate distinction from the response text.

Comment: Major comments

Supervised vs. Unsupervised Integration and Evaluation Metrics

In line 134, the authors report that the supervised version of Palette outperforms the unsupervised mode. However, the manuscript does not provide sufficient detail on how cell type annotation completeness or quality influenced these results. In practical applications, cell-type annotations are often incomplete or partially missing, and therefore the supervised framework should be evaluated under such realistic conditions (e.g., by introducing missing or "NA" labels). This would be feasible given that by default only 25% top-ranked SW cells are selected.

It also remains unclear how the annotations used for supervision were originally obtained — were they derived from a single modality (such as transcriptomic data) or from multi-modal consensus annotations? If annotations were modality-specific, this could introduce bias toward that modality and artificially enhance the apparent performance of supervised integration. It would be important to potential limitations.

In addition, the use of the same cell-type annotations used both for training (in supervised integration or batch correction) and for evaluation, introduces overfitting or validation bias.

This brings also the question of Palette's robustness to errors in cell type labels and to incomplete annotations, a common challenge in real-world single-cell datasets.

Response: We thank the reviewer for this constructive comment. To address these concerns, we conducted a unified set of analyses to assess the sensitivity and stability of supervised integration under perturbed annotation conditions.

To assess how annotation completeness and quality influence supervised integration, we evaluated Palette on six benchmark integration tasks under progressively perturbed label conditions. Specifically, we randomly masked or shuffled 5%, 10%, and 20% of the cell type labels to simulate missing or erroneous annotations. Using these modified labels, we re-ran supervised integration under six scenarios. Across datasets and metrics, performance showed limited sensitivity to increasing levels of label perturbation, and supervised integration continued to outperform the unsupervised setting in most cases (Supplementary Fig. 15).

In our benchmarks, labels used for supervision were taken directly from the original studies, which, except for the human retina 10x Multiome dataset (annotated based on scRNA-seq and subsequently validated using scATAC-seq), were derived using multimodal consensus strategies. In the revised manuscript, we examined whether different annotation strategies could bias supervised integration in

multimodal settings. Specifically, we re-annotated the human BMMC CITE-seq and 10x Multiome datasets using transcriptome-only information via two data-driven approaches: (1) reference-based label transfer and (2) k-means clustering on Seurat-integrated embeddings. Using these unimodal-derived labels, we re-ran supervised integration for the BMMC scenario 1 task. Performance changed moderately relative to multimodal annotations but remained consistently higher than unsupervised integration (Supplementary Fig. 16).

Additionally, in all experiments described above, the perturbed labels were used for supervised training, while all quantitative evaluations were performed using the original, unperturbed annotations. This design explicitly avoids using the same cell type annotations for both supervised training and evaluation, eliminating overfitting or validation bias.

These analyses showed that while supervised integration performance can be influenced by annotation quality and annotation strategy, Palette's supervised mode exhibits stable performance under realistic annotation perturbations and consistently outperforms the unsupervised alternative, without being overly sensitive to the provided supervision labels.

We have discussed the above scenarios in **Supplementary Note 1** of the revised Supplementary Information and provided the details of the original annotation strategies in the **Methods** section of the revised manuscript (*lines 757-761*).

Added Supplementary Figures:

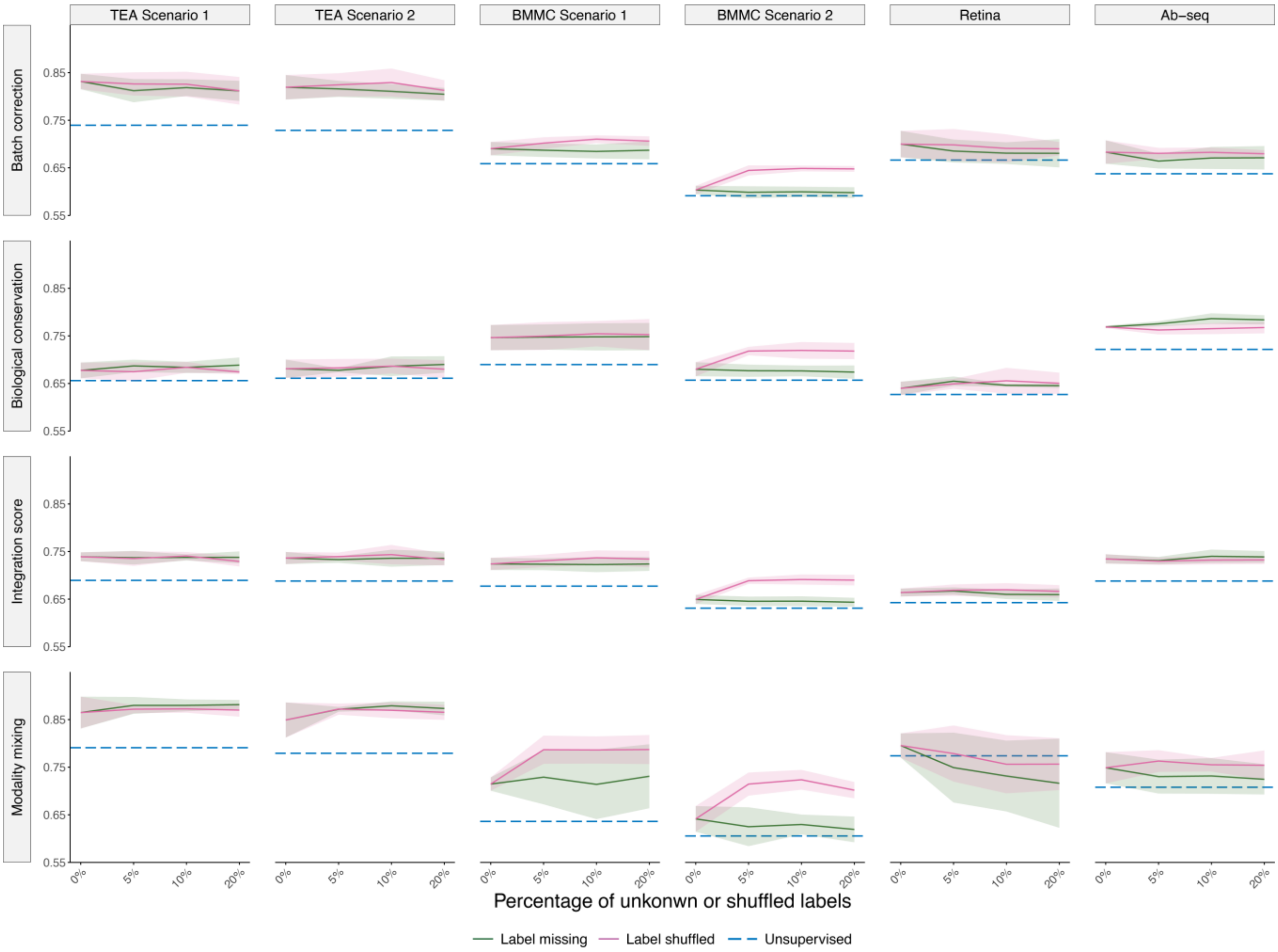

Supplementary Figure 15. Impact of noisy or incompleteness for cell type annotations on the supervised integration performance of Palette. Quantitative evaluation of Palette's supervised integration across multiple datasets under increasing proportions of unknown or shuffled cell type labels. Lines denote mean performance, and shaded regions represent the 95% confidence interval estimated from n = 5 randomly generated sub-experiments.

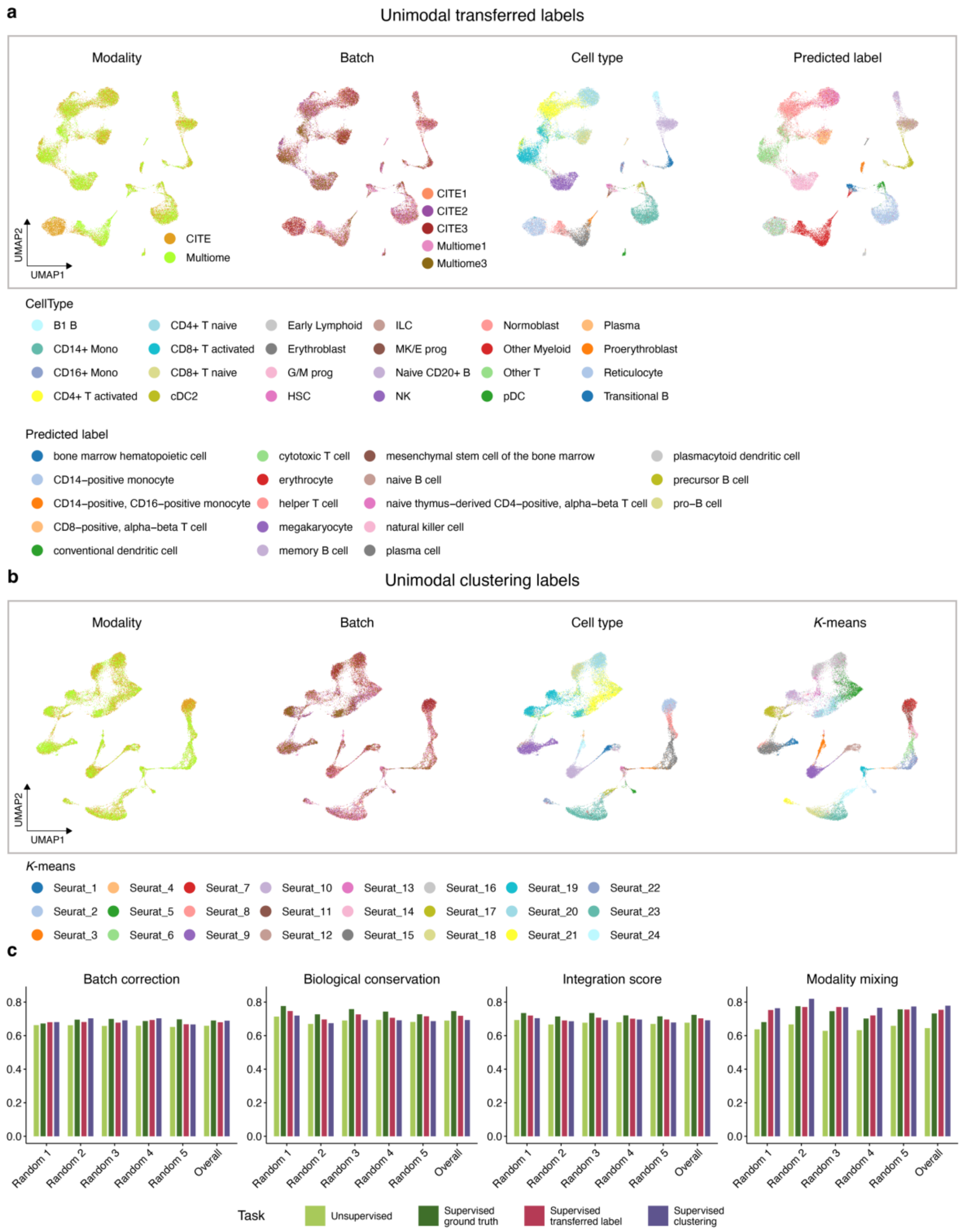

Supplementary Figure 16. Influence of label annotation strategy on the supervised integration performance of Palette. a, b. UMAP visualizations of Palette's supervised integration using two alternative unimodal-derived label sources: RNA-derived transferred labels (**a**) and labels derived from Seurat CCA–integrated transcriptomic embeddings followed by *k*-means clustering (**b**). Cells are colored by modality composition (first column), batch (second column), original labels (third column), and unimodal based labels (fourth column). **c.** Quantitative comparison of Palette's supervised integration under different annotation conditions, including the two unimodal-derived label sets, the

original dataset labels, and an unsupervised setting. For the two unimodal-derived label conditions, integration performance was evaluated using the dataset's original annotations. Performance is presented separately for four evaluation metrics: batch correction, biological conservation, overall integration, and modality mixing. For *k*-means, we specified the number of clusters to match the number of known cell types.

Added Supplementary Note:

Supplementary Note 1. Evaluation of Palette supervised integration performance under variable cell type annotation conditions

To assess how the quality and completeness of cell type labels influence Palette's supervised integration, we simulated label sets of varying reliability by randomly shuffling or masking 5%, 10%, and 20% of the labels. Palette was then re-run in supervised mode using these perturbed labels, and the resulting integrations were quantitatively evaluated (Supplementary Fig. 15). In nearly all cases, supervised integration with perturbed labels continued to outperform the unsupervised alternative, indicating that supervised integration remains effective even when labels are partially disrupted. Moreover, across all integration tasks, most evaluation metrics showed limited sensitivity to increasing noise or missingness, suggesting that Palette is stable with respect to label quality and completeness.

Considering annotation strategies used for multimodal datasets may also influence integration performance, we next evaluated the effect of annotation modality on integration performance. The human BMMC datasets generated by CITE-seq and 10x Multiome, whose original labels were derived from multimodal consensus, were re-annotated using transcriptome-based unimodal information. To avoid introducing human-driven biases, we employed two fully data-driven Seurat-based procedures: label transfer from the HCA BMMC scRNA-seq reference[1] and batch integration followed by k-means clustering to generate cluster labels. Using these two unimodal annotation schemes, we re-ran Palette supervised integration for the BMMC scenario 1 task (Supplementary Fig. 16a and b). Compared with multimodal consensus labels, unimodal annotations led to moderately lower scores for batch correction, biological conservation, and overall integration, whereas modality mixing performance was comparable or slightly improved (Supplementary Fig. 16c). Nevertheless, supervised integration under unimodal annotations consistently outperformed the unsupervised setting.

Collectively, these analyses indicate that Palette's supervised integration exhibits stable behavior across a range of realistic annotation perturbations, including incomplete labels, noisy labels, and differences in annotation strategy.

Modified Results (lines 136-146):

Moreover, we evaluated supervised mosaic integration by incorporating cell type labels as prior information. The supervised mode of Palette consistently outperformed the unsupervised mode across

multiple metrics (Supplementary Figs. 13 and 14), underscoring the benefits of leveraging manual annotations, an aspect often overlooked by existing methods. In addition, recent work shows that supervised integration approaches often exhibit sensitivity to variation in the quality or completeness of cell type labels[31]. We therefore systematically examined how annotation perturbations influence supervised integration performance. Quantitative results indicate Palette exhibits stable performance across a range of realistic annotation conditions, including incomplete or noisy labels and different annotation strategies, while maintaining a consistent advantage over unsupervised integration (Supplementary Figs. 15 and 16, and Supplementary Note 1).

Modified Methods (lines 754-761):

Mosaic integration benchmark datasets. We benchmarked mosaic integration using five multimodal single-cell datasets[9,28,29]: the human PBMC TEA-seq dataset, the human BMMC CITE-seq dataset, the human BMMC 10x Multiome dataset, the human BMMC Ab-seq dataset, and the human retina 10x Multiome dataset. Cell type annotations provided in the original studies were used as ground truth. Except for the human retina 10x Multiome dataset, whose cell type annotations were assigned based on transcriptomic profiles and further validated using the chromatin accessibility modality, all other datasets were annotated using multimodal consensus–based cell type labels.

Added Supplementary Reference:

1. Li, B. et al. HCA Data Portal: census of immune cells (Human Cell Atlas,2019).

Comment: These and other challenges of benchmarking performance of supervised integration methods were addressed e.g. in https://www.nature.com/articles/s41467-024-45240-z

Response: We thank the reviewer for pointing out this relevant study, which presents a novel semi-supervised batch integration framework called ssSTACAS and evaluates multiple metrics for quantifying integration quality. We have now cited this work in the revised manuscript.

In addition, we have also included ssSTACAS and its unsupervised version STACAS in our newly added single-modality integration benchmark study, following the reviewer's comment.

Added Reference:

31. Andreatta, M. et al. Semi-supervised integration of single-cell transcriptomics data. *Nat. Commun.* **15**, 872 (2024).

Comment: "Modality mixing" as an evaluation metric

Modality mixing metrics aim to quantify how well (cells from) datasets with different modality compositions are mixed. The use of modality mixing metrics (such as the Seurat Alignment Score, modality kBET, and modality LISI F1) requires careful interpretation. In particular, the current

approach may not provide an unbiased assessment of integration quality because it relies on k-nearest neighbor (kNN) graphs constructed in the integrated latent space, which depend itself on the integration method.

Further, when modality coverage is highly imbalanced, or when the graph is built using closest kNN structures derived from one dominant modality, the resulting “mixing” may reflect technical artifacts rather than true biological alignment. It would be important to discuss these limitations and eg complement these metrics with external biological validations (e.g., preservation of known cell-type markers or cross-modality feature correlations).

***Response*:** We thank the reviewer for this constructive comment. We agree that modality mixing metrics should be interpreted with care because they are computed on a kNN graph in the integrated latent space and without any prior biological information combined. In current practice, the assessment of single-cell multimodal mosaic integration quality is typically conducted along two complementary dimensions: batch correction and biological conservation[1-3]. In this study, we follow this established evaluation paradigm and further introduce modality mixing metrics to quantitatively assess the central focus of our work, the integration of single-cell datasets with imbalanced modality composition. Importantly, modality mixing metrics are not used as standalone or decisive measures, but are interpreted jointly with batch correction and biological conservation metrics to provide a more comprehensive evaluation of integration quality.

We acknowledge the reviewer’s concern that modality mixing metrics may be inflated by erroneous biological alignment across datasets with different modality compositions. To alleviate this issue, we have refined the modality mixing metrics by computing them within each cell type rather than at the dataset level. Specifically, we compute the Seurat Alignment Score, kBET, and normalized iLISI (referred to as CSAS, modality kBET, and CiLISI in the revised manuscript, respectively) within each cell type using modality composition labels. This evaluation strategy reduces confounding effects arising from differences in cell type composition, enabling the assessment of modality mixing among biologically comparable cells. Palette still demonstrated consistent superiority for the modified modality mixing metrics. However, as with other mixing metrics, modality mixing alone does not distinguish desirable alignment from overcorrection. Therefore, it should be interpreted jointly with biological-related metrics, which provide the necessary constraint for identifying biologically meaningful structure preserved in the integrated representation. We have emphasized this applicability boundary in the revised manuscript.

In addition, we conducted systematic simulation studies to test the robustness and sensitivity of these modality mixing metrics under controlled settings (Supplementary Note 5 and Supplementary Fig. 49). These simulations were designed to characterize the behavior of different modality mixing metrics under varying degrees of modality mixing, unequal data sizes across modality compositions, and

mismatched cell type compositions. The results show that the modality mixing metrics remain sensitive to increasing degrees of true modality mixing while being robust to data size imbalance and cell type composition mismatch, in contrast to several dataset-level metrics.

Regarding the external biological validations suggested by the reviewer, such as preservation of known cell-type markers or cross-modality feature correlations, we agree that these are conceptually valuable criteria. However, under the mosaic integration setting considered here, there is currently no principled and modality-general strategy that can be applied fairly across different integration frameworks without introducing additional assumptions. First, several mosaic integration methods, including scMoMaT, StabMap, and Palette, do not output corrected expression matrices but instead provide only a shared latent representation. As a result, expression-level marker preservation cannot be evaluated in a unified and method-agnostic manner. Second, quantifying cross-modality feature correlations requires well-defined a priori biological correspondences between modalities. While such correspondences exist between transcriptomic and proteomic features, they are not directly available for transcriptomic and chromatin accessibility data. In the case of scATAC-seq data, the relationship between peaks and genes is indirect, cell-type–specific, context-dependent, and typically inferred computationally rather than known beforehand[4]. Consequently, defining a systematic, objective, and generally applicable cross-modality feature correlation metric is not feasible in this setting. We therefore focus on internally consistent and method-comparable evaluation metrics, and explicitly discuss the scope and limitations of modality mixing metrics in the revised manuscript.

We have updated the **Methods** section in the revised manuscript (*lines 1083–1115*) to clarify the revised definition and computation of the modality mixing metrics, as well as emphasized the applicability boundary. Moreover, we have added details about the simulation study of modality mixing metrics in **Supplementary Note 5** and **Supplementary Methods** of the revised Supplementary Information.

References:

1. Lotfollahi, M., Litinetskaya, A. & Theis, F.J. Multigrate: single-cell multi-omic data integration. Preprint at *bioRxiv* https://doi.org/10.1101/2022.03.16.484643 (2022).
2. He, Z. et al. Mosaic integration and knowledge transfer of single-cell multimodal data with MIDAS. *Nat. Biotechnol.* **42,** 1594–1605 (2024).
3. Zhang, Z. et al. scMoMaT jointly performs single cell mosaic integration and multi-modal bio-marker detection. *Nat. Commun.* **14,** 384 (2023).
4. Pliner, H. A. et al. Cicero predicts cis-regulatory DNA interactions from single-cell chromatin accessibility data. *Mol. Cell* **71,** 858–871.e8 (2018).

Modified Figures:

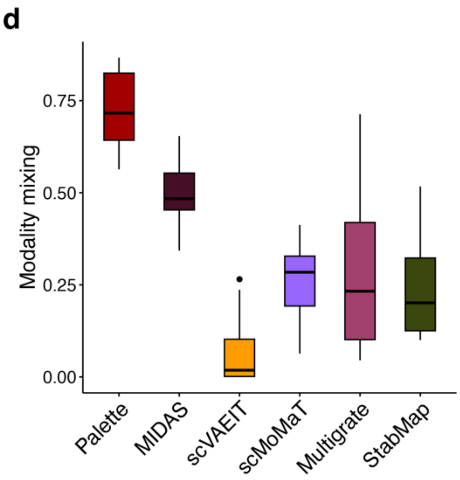

Figure 2. Systematic benchmarking of Palette against state-of-the-art integration methods. d. Box plot of overall modality mixing scores across methods. The central lines mark the median values, the boxes show the quartiles, and the whiskers show the rest of the distribution.

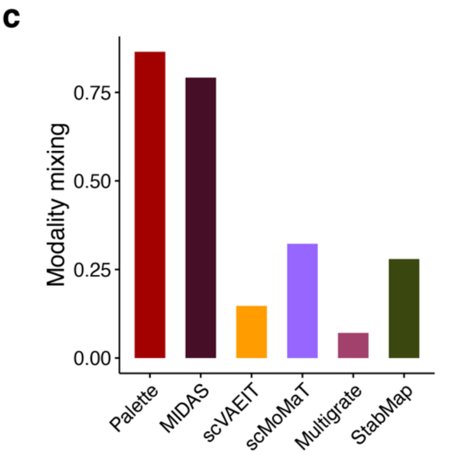

Figure 3. Results on the cross-condition human PBMC dataset. c. Comparison of modality mixing score for different integration methods.

Modified Supplementary Figures:

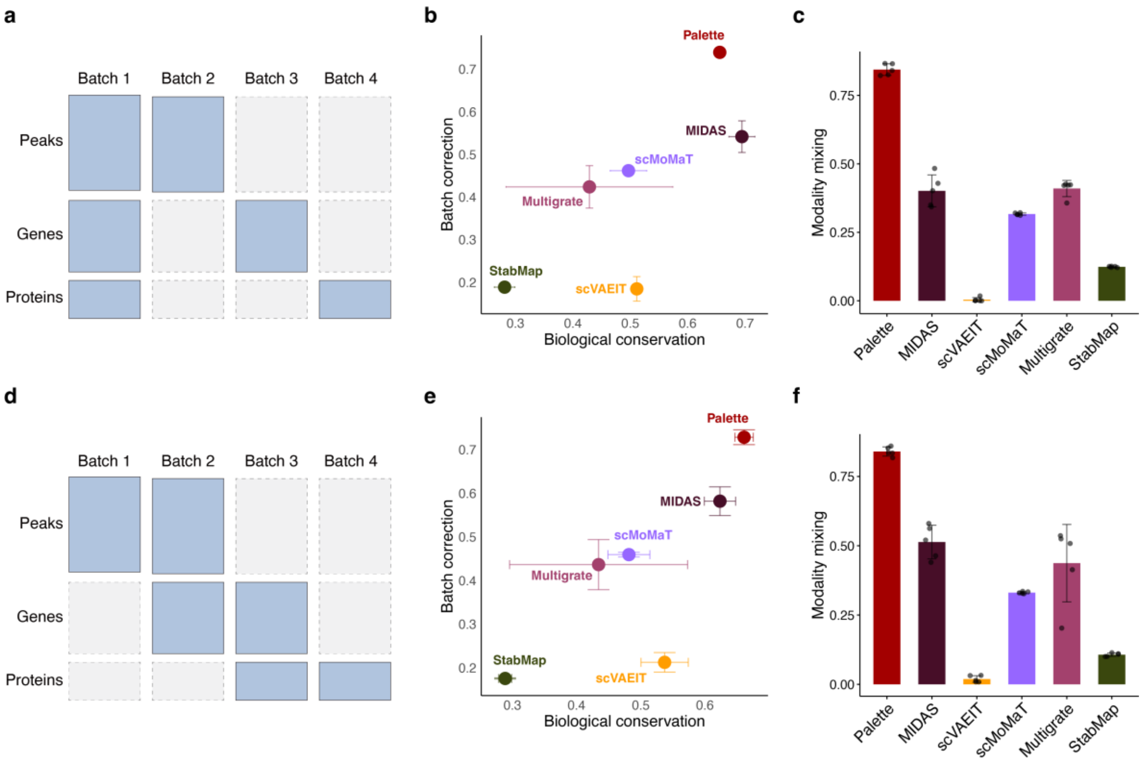

Supplementary Figure 2. Benchmarking of Palette and other integration methods on the TEA scenarios 1 and 2. The top row (**a**–**c**) summarizes results for TEA scenario 1: (**a**) modality composition of each batch; (**b**) comparison of mean biological conservation versus mean batch correction across methods; and (**c**) mean modality mixing scores. The bottom row (**d**–**f**) presents the corresponding results for TEA scenario 2. Error bars represent the standard error across evaluated tasks, from $n = 5$ randomly generated sub-experiments.

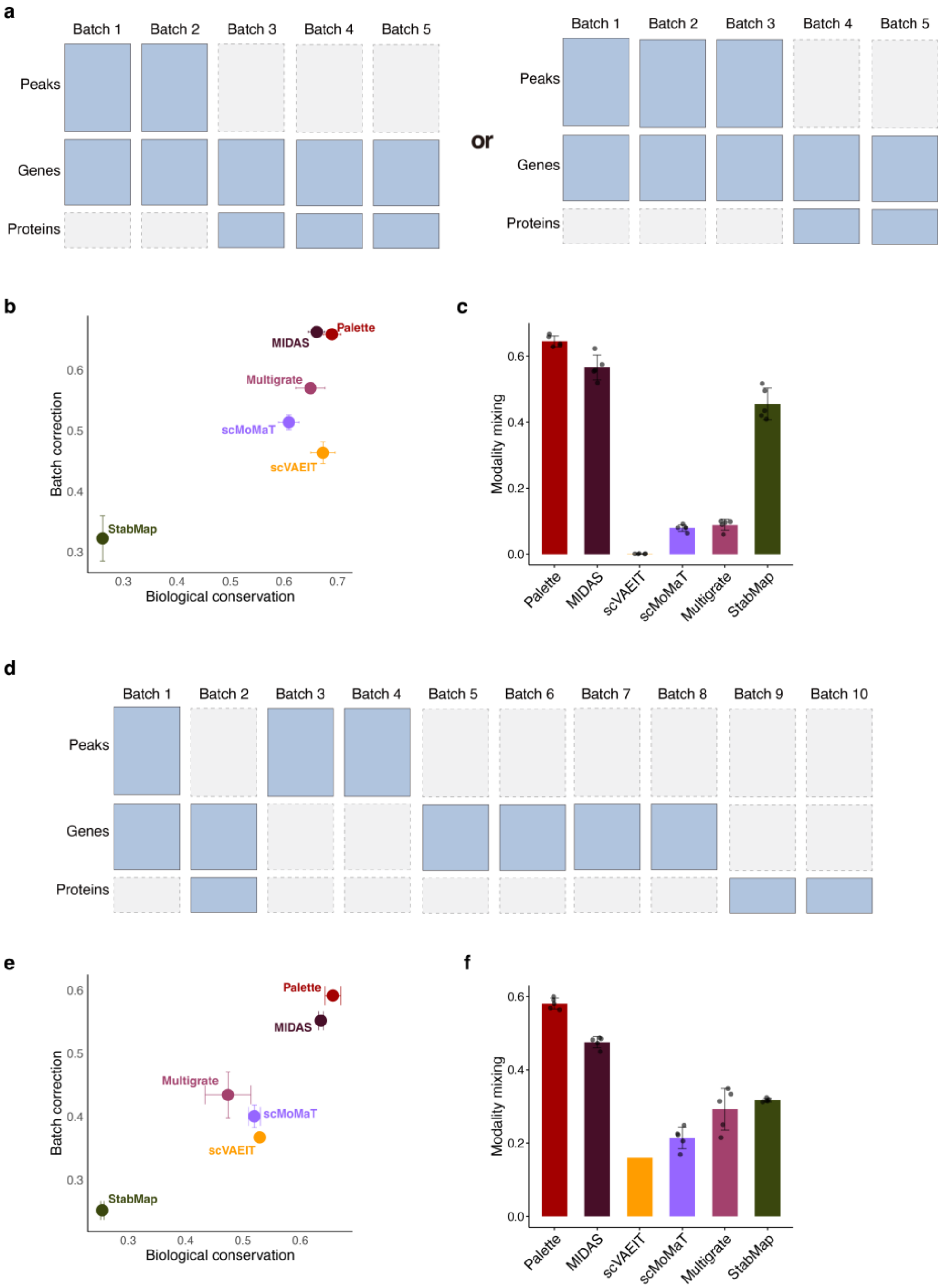

Supplementary Figure 3. Benchmarking of Palette and other integration methods on the BMMC scenarios 1 and 2. The top two rows (**a**–**c**) summarize results for BMMC scenario 1: (**a**) modality composition of each batch; (**b**) mean biological conservation versus batch correction across methods; and (**c**) mean modality mixing scores. The bottom two rows (**d**–**f**) present the corresponding results for BMMC scenario 2. Error bars represent the standard error across evaluated tasks, from *n* = 5 randomly generated sub-experiments. For BMMC scenario 2, scVAEIT completed only one sub-experiment, so no error bars are shown for this method.

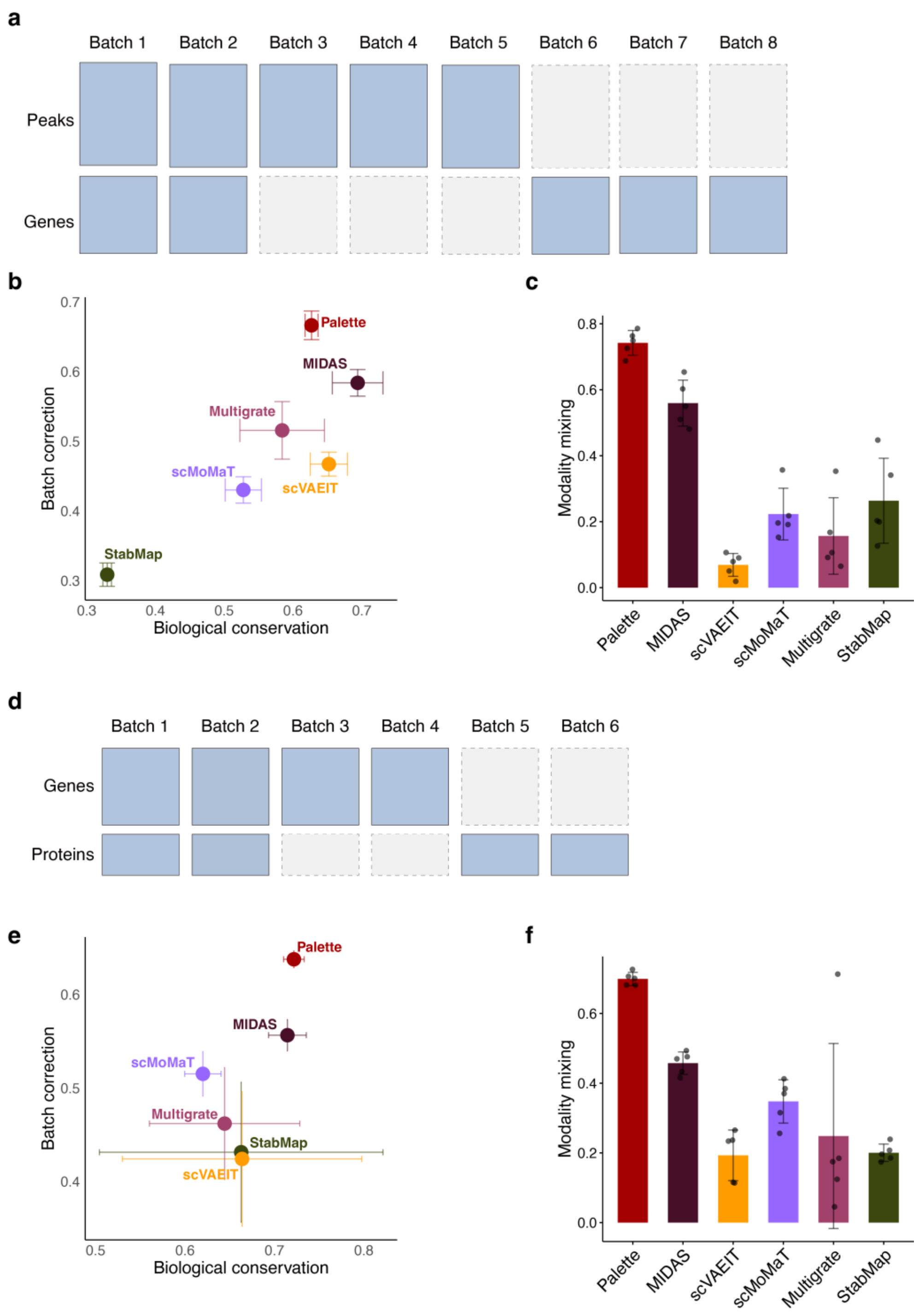

Supplementary Figure 4. Benchmarking of Palette and other integration methods on the Retina and Ab-seq datasets. The top two rows (**a**–**c**) summarize results for the Retina dataset: (**a**) modality composition of each batch; (**b**) comparison of mean biological conservation versus mean batch correction across methods; and (**c**) mean modality mixing scores. The bottom two rows (**d**–**f**) present the corresponding results for the Ab-seq dataset. Error bars indicate the standard error across evaluated tasks, from *n* = 5 randomly generated sub-experiments.

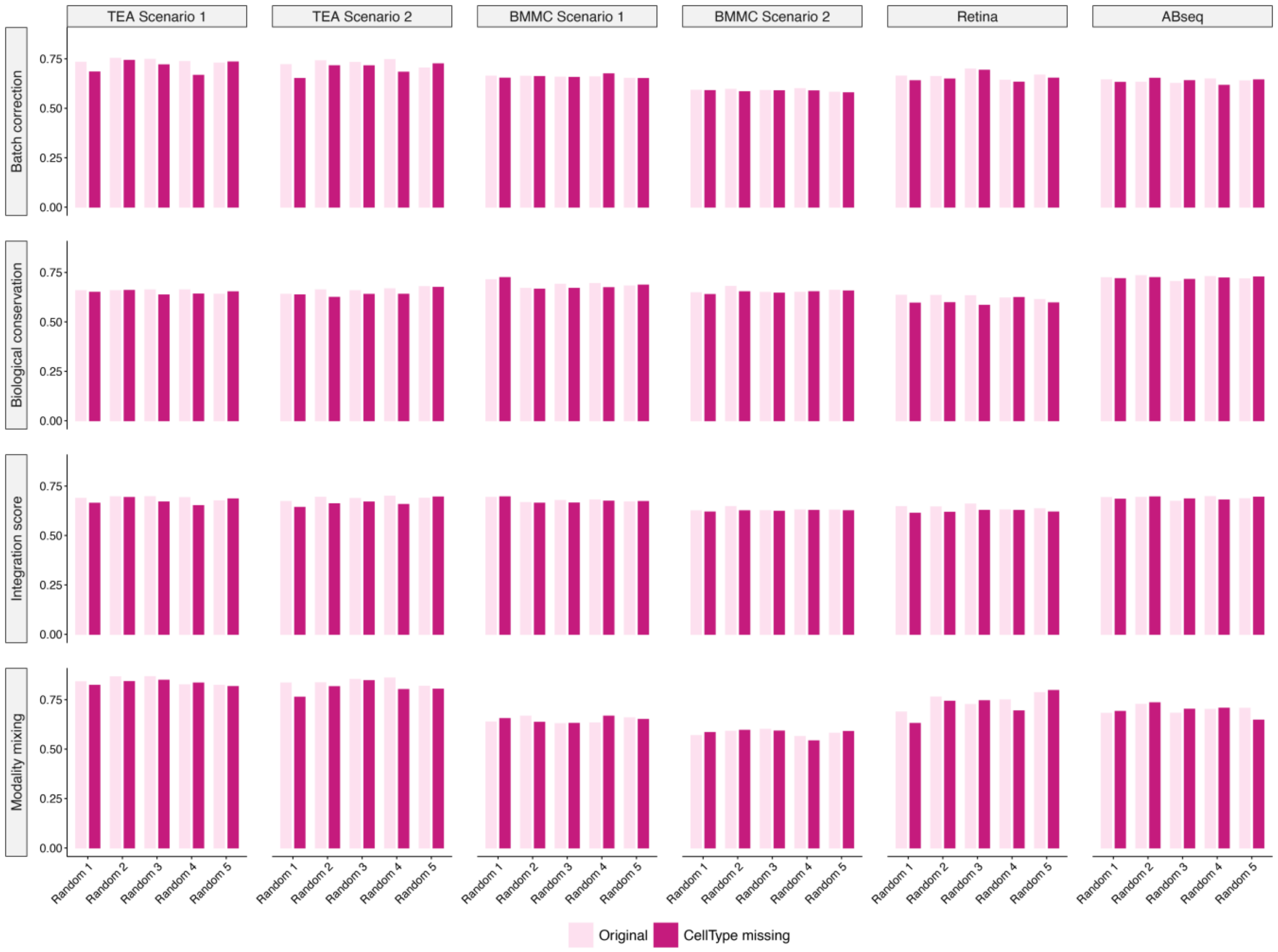

Supplementary Figure 11. Evaluation of Palette's integration performance under imbalanced cell type compositions. Across six integration scenarios and multiple metrics, we compared results on original datasets with those from modified versions in which one cell type was randomly removed from each batch to introduce compositional imbalance. Palette maintained consistently high performance across all scenarios, demonstrating robustness to biological heterogeneity.

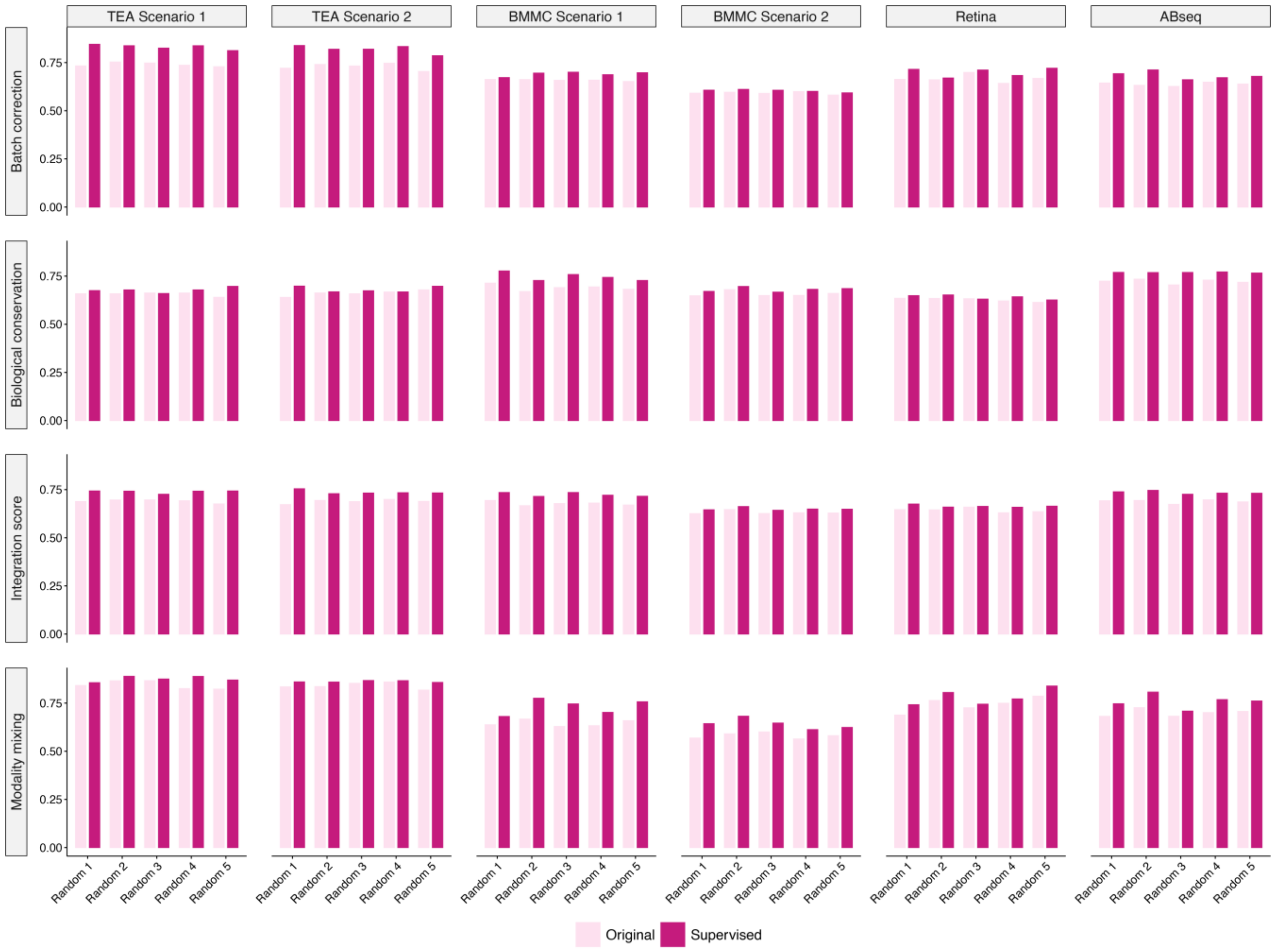

Supplementary Figure 13. Evaluation of Palette's supervised integration performance. Across six integration scenarios and multiple evaluation metrics, we compared Palette's supervised integration mode with its unsupervised counterpart. Supervised integration consistently yielded improved quantitative scores across scenarios.

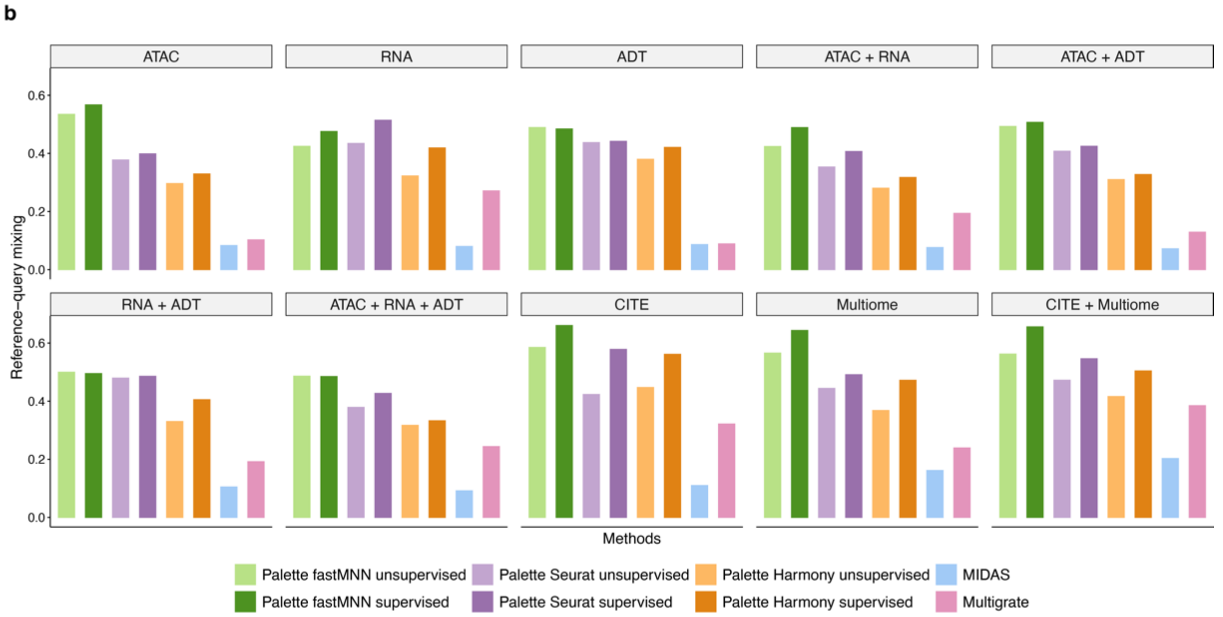

<u>Supplementary Figure 19. Evaluation of cell type label transfer and dataset mixing in reference-based integration across multiple query scenarios. b.</u> <u>Bar plots showing the mixing scores between reference and query cells for each query scenario.</u>

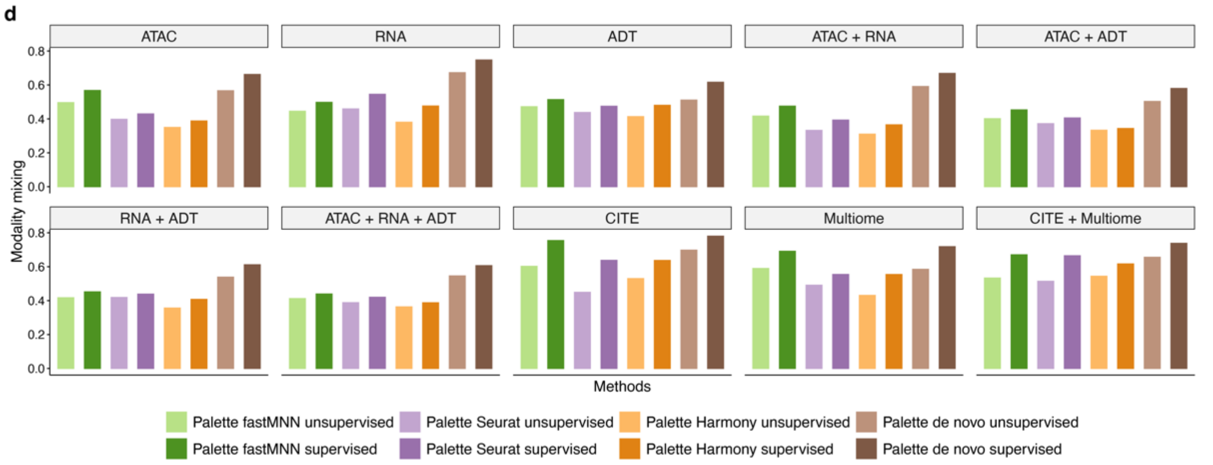

<u>Supplementary Figure 20. Comparison of integration performance between reference-based and de novo integration across multiple query scenarios.</u> <u>We quantitatively evaluated four integration metrics: (**a**) batch correction score, (**b**) biological conservation score, (**c**) overall integration score, and (**d**) modality mixing score. For supervised de novo integration, cell type labels for each query dataset were obtained by transferring annotations from the reference using reference-based integration with supervised reference embeddings. For evaluation purposes, the ground truth cell type labels of the query datasets were used to compute all metrics.</u>

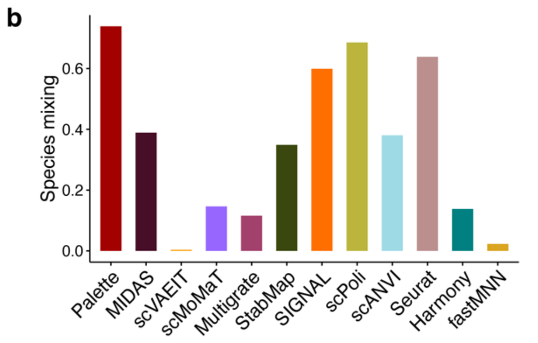

Supplementary Figure 27. Integration results of cross-species WAT data based on orthologous genes. a, b. Comparison of overall integration scores (**a**) and species mixing scores (**b**) across different integration methods.

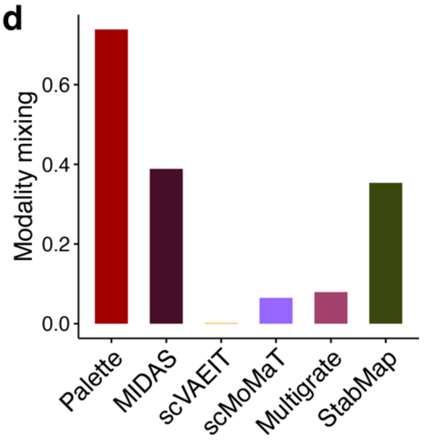

Supplementary Figure 28. Comparison of integration performance with and without inclusion of non-orthologous genes. c, d. Comparison of overall integration scores (**c**) and modality mixing scores (**d**) across methods under the non-orthologous gene inclusion setting.

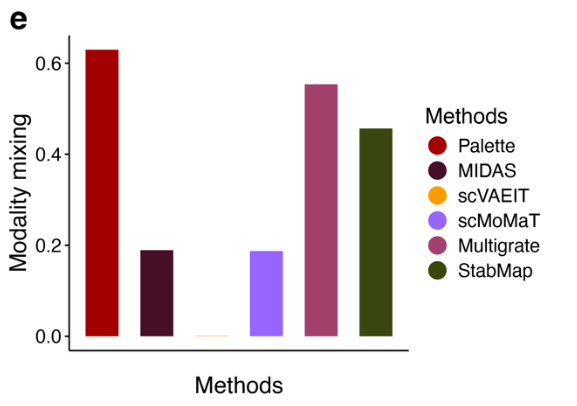

Supplementary Figure 29. Integration results of human tonsil 10x Visium data. e. Comparison of modality mixing scores across methods.

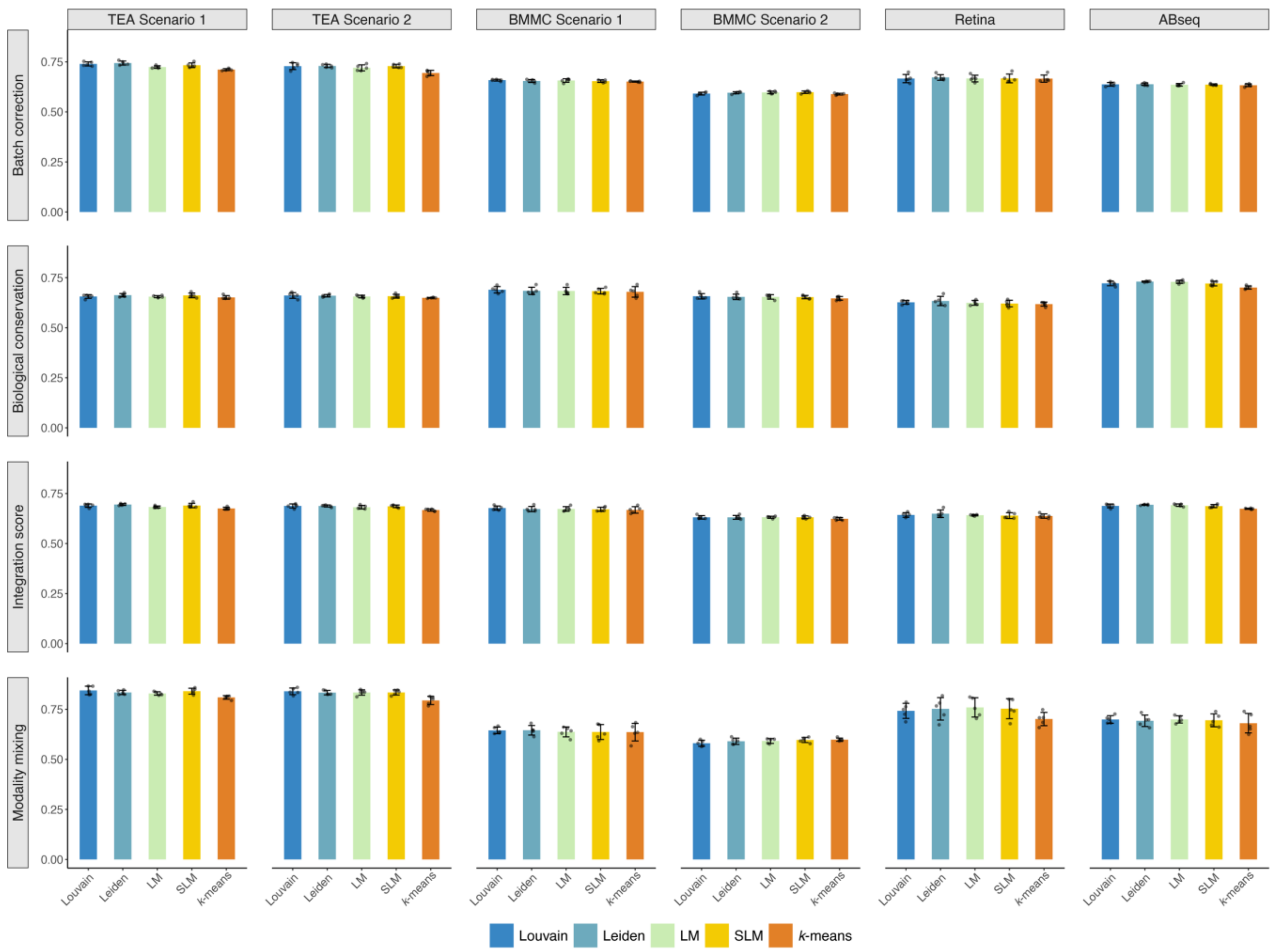

Supplementary Figure 41. Benchmarking clustering algorithms within the unsupervised Palette framework. We evaluated integration performance using multiple quantitative metrics across different clustering strategies combined with Palette: Louvain (default in Palette), Leiden, Louvain with multilevel refinement (LM), smart local moving (SLM), and *k*-means. For Louvain, Leiden, LM, and SLM, the resolution parameter was fixed at 1, consistent with the default setting in Palette. For *k*-means, we specified the number of clusters to match the number of known cell types in each batch. Error bars represent the standard error across evaluated tasks, from n = 5 randomly generated sub-experiments.

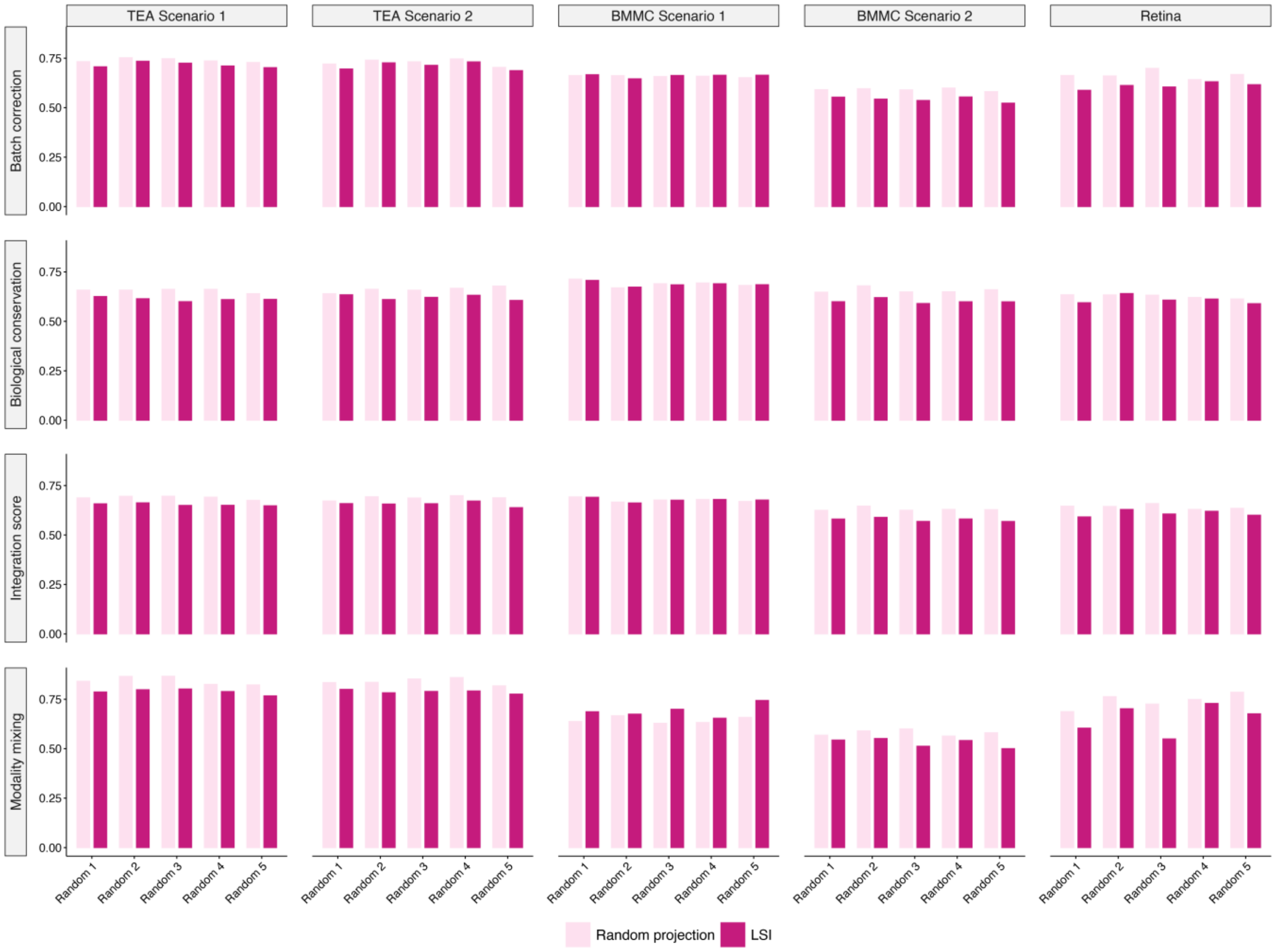

Supplementary Figure 42. Evaluation of Palette's integration performance using different pre-dimensionality reduction methods for chromatin accessibility data. Integration results based on two pre-dimensionality reduction strategies, random projection and LSI, were compared across five integration scenarios using multiple metrics.

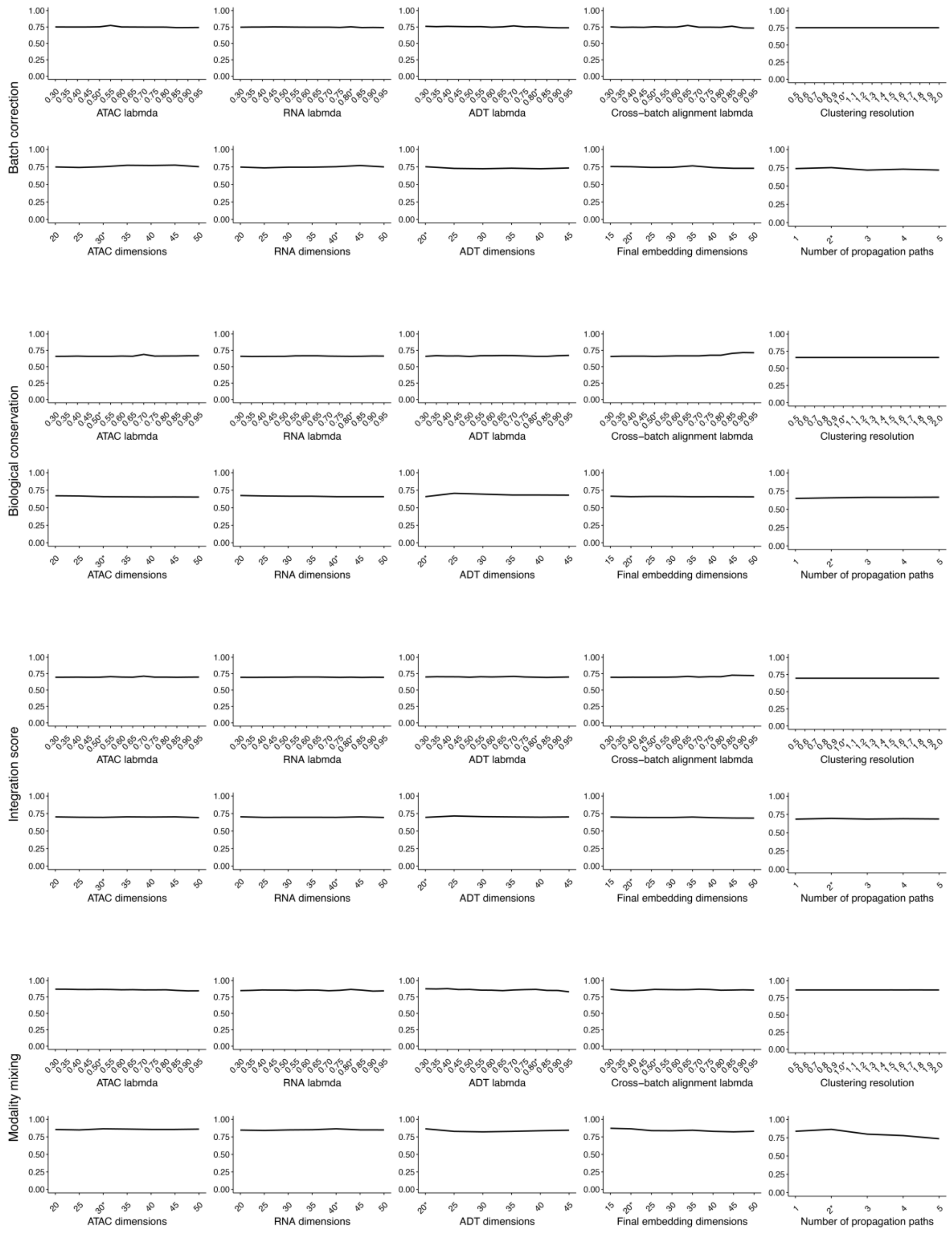

Supplementary Figure 46. Robustness benchmarking of Palette integration with respect to tuning parameters, using the dataset of the third sub-experiment under the TEA scenario 1. Integration performance was evaluated based on batch correction scores, biological conservation scores, overall integration scores, and modality mixing scores. These metrics were assessed across a range of tuning parameters, including: the Bi-sPCA regularization parameter (λ) used for different modalities and integration stages; clustering resolution for kernel matrix construction in unsupervised

mode; dimensionality settings for intra-modal joint reduction and final embedding; and the number of propagation paths. For protein-specific intra-modal dimensionality reduction, the dimension was set to 20 for the human PBMC TEA-seq dataset (which contains 46 ADT features) to ensure sufficient representation across all sub-experiments based on this dataset. In addition, asterisks (*) denote the default parameter settings used by Palette.

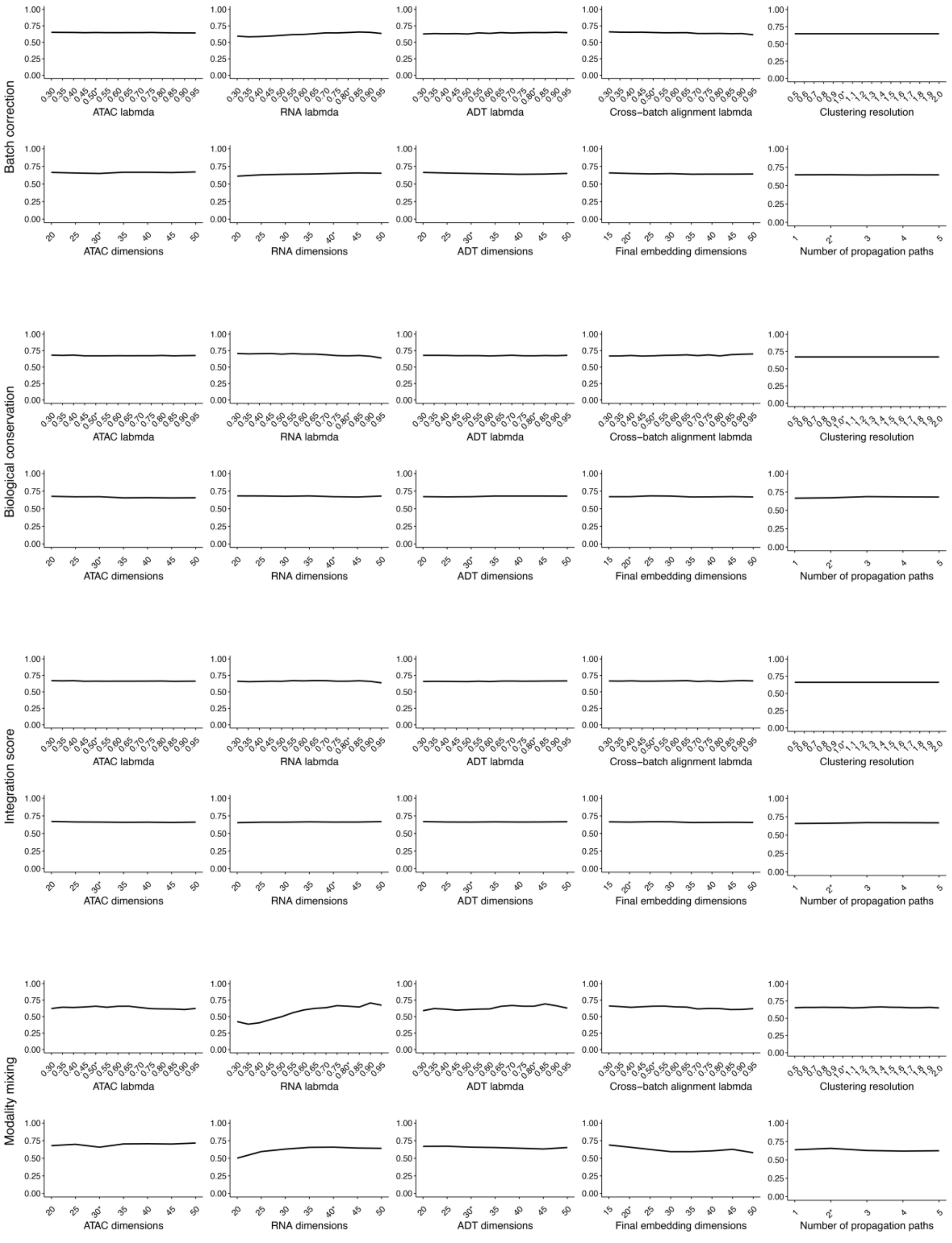

Supplementary Figure 47. Robustness benchmarking of Palette integration with respect to tuning parameters on the human BMMC CITE-seq and 10x Multiome dataset. Integration performance was evaluated based on batch correction scores, biological conservation scores, overall integration scores, and modality mixing scores. These metrics were assessed across a range of tuning parameters, including: the Bi-sPCA regularization parameter (λ) used for different modalities and integration stages; clustering resolution for kernel matrix construction in unsupervised mode; dimensionality settings for intra-modal joint reduction and final embedding; and the number of propagation paths. An asterisk (*) indicates the default parameter setting used by Palette.

Modified Methods (lines 1083-1115):

Modality mixing metrics. To quantify the mixing of cells originating from different modality compositions, we employed three metrics: the cell-type–stratified Seurat alignment score (CSAS), modality kBET, and CiLISI[31]. These metrics characterize the extent of modality mixing in mosaic integration settings but are not intended to serve as standalone measures of overall integration quality. It should therefore be evaluated together with additional biological metrics. In this study, integration quality was assessed by combining the modality mixing metric with the biological conservation and batch correction metrics.

CSAS. The Seurat alignment score (SAS), originally introduced by Butler et al.[104], measures how well datasets are aligned in a shared embedding space. To account for heterogeneity across cell types, we computed SAS separately within each cell type and reported the median value as CSAS. For a given cell type c, datasets representing different modality compositions were first downsampled to match the smallest group. SAS was then computed on the kNN graph as

$$SAS_c = 1 - \frac{\bar{x}_c - \frac{K_c}{N_c}}{K_c - \frac{K_c}{N_c}} \ ,$$

where N_c is the number of modality compositions present in cell type c, and $\bar{x}_c$ is the average number of neighbors sharing the same modality composition. Following Butler et al.[104], K_c was set to 1% of the downsampled cell count. As with SAS, CSAS ranges from 0 to 1, with higher values indicating stronger modality mixing.

Modality kBET. To evaluate mixing using local neighborhood homogeneity, we adapted kBET by treating modality composition labels as the grouping variable. kBET was computed within each cell type, and the median rejection rate across cell types was used as the modality kBET score.

CiLISI. CiLISI, originally proposed by Andreatta et al.[31], evaluates batch mixing by computing iLISI within individual cell types. To account for variability in cell type abundance and the differing modality compositions, we implemented a modified version. Within each cell type, datasets were downsampled to the smallest modality group, and iLISI was calculated on the downsampled cells. The

resulting values were normalized to the range 0 to 1, and the median across all cell types was reported as CiLISI.

Modality mixing score. The modality mixing score is defined as the mean value of CSAS, modality kBET, and CiLISI. We formulize this metric as follows:

$$S_{\mathrm{mod}} = (\mathrm{CSAS} + \mathrm{kBET}_{\mathrm{mod}} + \mathrm{CiLISI})/3,$$

where S_{mod} indicates the modality mixing score, and $\mathrm{kBET}_{\mathrm{mod}}$ denotes modality kBET, respectively. We further tested the behavior of these three metrics against four additional metrics using a suite of simulated scenarios designed to probe their sensitivity and robustness (Supplementary Fig. 51 and Supplementary Note 5).

Added Supplementary Note:

Supplementary Note 5. Evaluation of modality mixing metrics

To evaluate the behavior of modality mixing metrics, we assessed seven metrics using a suite of simulated scenarios. These metrics include three measures (details of metric definitions are provided in the Methods), CSAS, modality kBET, and CiLISI, which are computed separately within each cell type, as well as four additional metrics computed at the dataset level, including the original kBET, SAS, iLISI, and LISI F1.

We first simulated datasets with varying degrees of modality mixing across modality compositions (Supplementary Fig. 51a and Supplementary Methods). As the mixing level increased, all metrics except the original kBET exhibited a consistent upward trend (Supplementary Fig. 51b). The reduced sensitivity of the original kBET in this setting can be attributed to the presence of modality-specific cell types, which violate its assumption of shared label distributions across datasets. Under perfect mixing, only modality kBET, CSAS, and CiLISI reached values close to 1, whereas the remaining metrics remained comparatively low.

We next examined the effect of imbalanced data sizes across modality compositions by progressively removing cells from Modality 1 under the Well Mixed setting (Supplementary Methods). In this scenario, iLISI and LISI F1 decreased as the degree of imbalance increased, while all other metrics remained largely stable (Supplementary Fig. 51c). This behavior indicates that most metrics are robust to unequal data sizes across modality compositions, whereas iLISI and LISI F1 are more sensitive to data size imbalance.

Finally, we evaluated scenarios with mismatched cell type compositions across modality compositions. We distinguished between balanced settings, in which all modality compositions share identical cell type compositions, and imbalanced settings, in which specific cell types are present in only a subset of modality compositions (Supplementary Fig. 51d and Supplementary Methods). In the imbalanced setting, SAS and iLISI achieved high scores (Supplementary Fig. 51e). In contrast, under

balanced settings, the original kBET, SAS, and iLISI produced inflated values, reflecting their insensitivity to cell type mismatches.

Taken together, these results show that the metrics quantifying within cell types, modality kBET, CSAS, and CiLISI, are robust to imbalanced cell type distributions and unequal data sizes across modality compositions, while remaining sensitive to true modality mixing. We therefore selected these three metrics as the primary measures of modality mixing throughout this study.

Added Supplementary Figure:

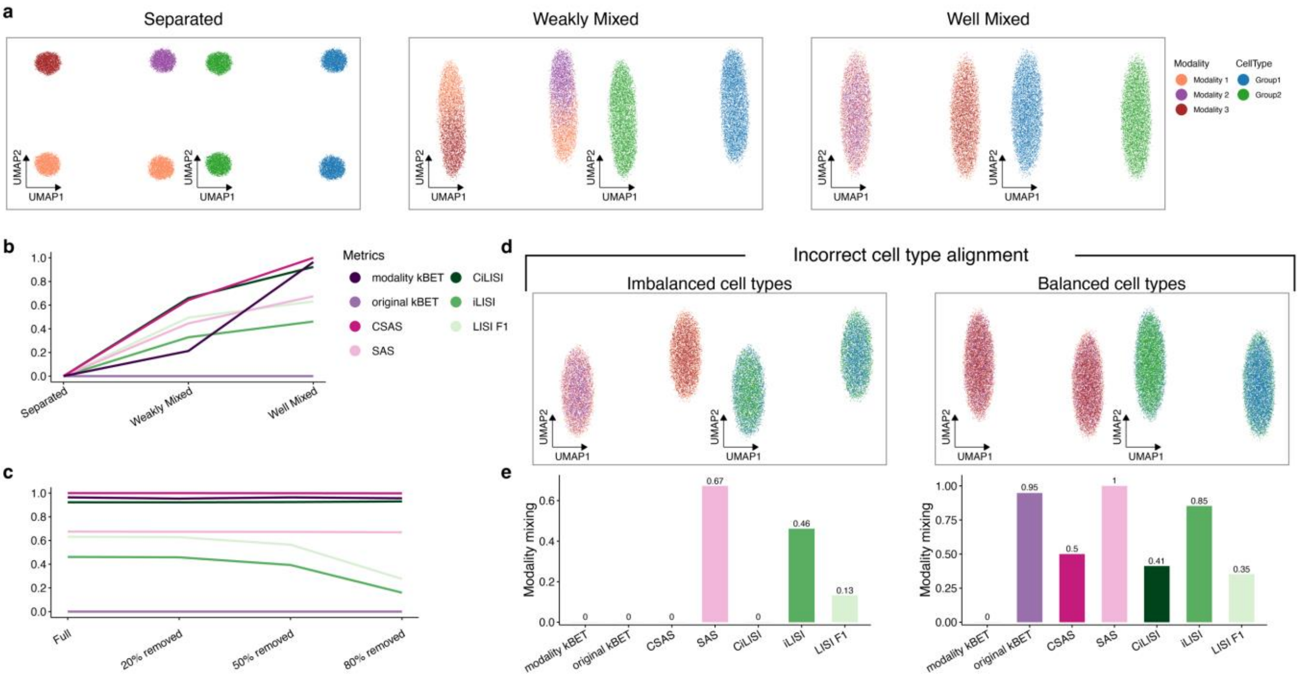

Supplementary Figure 51. Quantitative evaluation of modality mixing metrics across simulated scenarios. a. UMAP visualizations of cell embeddings under three simulated scenarios representing increasing levels of modality mixing. Cells are colored by modality composition (first, third, and fifth columns) and by cell type (second, fourth, and sixth columns). **b.** Quantitative results of seven evaluation metrics under increasing levels of modality mixing. **c.** Line plots showing metric changes under increasing levels of imbalance in sample sizes among different modality compositions. **d.** UMAP visualizations showing embeddings under incorrect cross-modality cell type alignment for datasets with imbalanced (left) and balanced (right) cell type compositions. Cells are colored by modality composition (first and third columns) and by cell type (second and fourth columns). **e.** Bar plots showing metrics performance under incorrect cross-modality cell type alignment for both imbalanced (left) and balanced (right) scenarios.

Added Supplementary Methods:

Data simulation. We generated simulated datasets with different degrees of modality mixing across modality compositions using the splatter R package (v1.22.1). Specifically, three baseline scenarios

were generated, referred to as Separated, Weakly Mixed, and Well Mixed, by jointly setting the parameters batch.facScale and batch.facLoc to 0.1, 0.03, and 0, respectively. Each simulated dataset consisted of three modality compositions (denoted as Modality 1, Modality 2, and Modality 3) and a total of 6,000 cells. Two cell types, termed Group 1 and Group 2, were present in equal proportions. To construct datasets used for evaluating modality mixing under the presence of modality-specific cell types, we applied an additional filtering step to all three baseline scenarios. Specifically, Group 1 cells were removed from Modality 3, and Group 2 cells were removed from Modality 2. The resulting datasets, which exhibit imbalanced cell type distributions across modality compositions, were used for assessing metric sensitivity to varying degrees of modality mixing. To simulate an imbalance in cell numbers across modality compositions, we focused on the filtered Well Mixed dataset described above. From this dataset, we randomly removed 20%, 50%, and 80% of cells from Modality 1, thereby generating three levels of increasing cell number imbalance across modality compositions. These datasets were used for evaluating metric robustness to unequal data sizes. To simulate incorrect cell type alignment across modality compositions, we considered two settings based on the Well Mixed scenario. In the balanced setting, cell type labels in Modality 1 of the original Well Mixed dataset were swapped, such that cells originally labeled as Group 1 were relabeled as Group 2, and vice versa. In this case, all modality compositions retained identical cell type compositions despite the label permutation. In the imbalanced setting, the same label swapping was applied to Modality 1 of the filtered Well Mixed dataset, in which Group 1 had been removed from Modality 3 and Group 2 from Modality 2. This procedure resulted in mismatched cell type compositions across modality compositions and was used to evaluate metric behavior under cell type misalignment.

Comment: Definition and Scope of "Differentially Expressed Features"
In lines 208–211, the authors state that, after removing Palette-identified differentially expressed features (DEFs), they annotated ten immune cell subtypes across transcriptomic, epigenomic, and proteomic layers (Fig. 3h–i). However, it is unclear how these DEFs were obtained. Earlier in the manuscript (Lines 198–207), the DEFs were described as differences between stimulated and control T cells, not across all immune cell types.
The authors should clarify whether the same T cell–specific DEF set was used for this broader immune cell analysis, or whether new DEFs were computed across all immune populations. In particular, it is not evident how removing features derived from a single cell type would impact subtype resolution across multiple immune lineages.

Response: We thank the reviewer for highlighting the ambiguity in our description of differentially expressed features (DEFs). In the cross-condition human PBMC dataset analysis, the features removed before re-annotation were exclusively the T cell–specific DEFs, defined as features that are

differentially expressed between stimulated and control cells within the T cell compartment. We did not compute a new "global" DEF set across all immune populations for the feature-removal step. After removing these T cell–specific DEFs, we performed a standard annotation workflow. Briefly, we clustered the Palette-integrated embedding and then identified cluster markers across modalities to support subtype labeling. Importantly, these cluster markers were used only for annotation and interpretation. They were not used for additional feature removal and are therefore not referred to as DEFs.

In addition, the purpose of removing T cell–specific DEFs is to assess whether Palette can disentangle stimulation-associated T cell state variation from lineage-defining biological structure. As described in the original study by Mimitou et al., the PBMCs were stimulated under a T cell–activating condition, and stimulation-dependent changes were reported predominantly within T cells. Consistent with the intended scope of feature removal, eliminating T cell–specific DEFs increased mixing between stimulated and control T cells, whereas non–T cell populations retained clear and stable clustering patterns, and the refined annotations remained highly concordant with the original labels at the level of major immune lineages (Fig. 3g–i and Supplementary Fig. 23).

We have revised the **Results** section (*lines 187, 210-211, 212, 216-219, and 221-228*) to clarify the description of the biological condition, explicitly define T cell–specific DEFs, and clarify the scope and interpretation of the subsequent subtype refinement.

Modified Results (lines 182-228):

Integrating mosaic datasets with batches from different biological conditions is a challenging scenario, as the inherent variation of mosaic integration is confounded with the biological variation introduced by conditions. To test the capability of Palette in such integration, we employed a well-controlled human peripheral blood mononuclear cells (PBMCs) dataset from Mimitou et al.[10] (Methods), comprising two CITE-seq and two ASAP-seq batches, with each technology containing one control and one T cell stimulated condition (Fig. 3a). Among all tested methods, Palette, MIDAS, and scMoMaT yielded notably better modality mixing in the uniform manifold approximation and projection (UMAP) embeddings, while the remaining methods struggled with batch alignment (Fig. 3b and Supplementary Fig. 21). Quantitatively, Palette achieved the highest modality mixing scores (Fig. 3c).

Palette-integrated result revealed a subtle but consistent stimulation-associated aggregation within T cells (Fig. 3b), suggestive of underlying condition-specific biological variation. To determine whether this pattern reflects true biological effects, we performed unsupervised clustering followed by differential expression (DE) analysis on T cells across all three modalities (Supplementary Fig. 22a and Methods). This analysis identified 84 DE proteins, 1,189 DE genes, and 9,097 DE peaks, showing concordance with the original study (Fisher's exact test[35], $p < 2e\text{-}17$; Supplementary Fig. 22b and

Methods). Notably, both Palette and Mimitou et al. consistently identified key molecular signatures at the proteomics level, including the upregulation of canonical T cell activation markers (CD69, CD25, CD71, CD278 proteins) in stimulation-enriched T cells and elevated expression of CD3 and CD28 proteins in control-enriched cells[36-39] (Fig. 3d). At the transcriptome level, genes such as *CXCR4*, *TFRC*, and *IL2RA* were consistently detected by both analyses (Supplementary Fig. 22c). Gene Ontology (GO) enrichment analysis of genes upregulated in the stimulation-enriched cluster revealed overrepresentation of pathways associated with cytokine signaling, immune effector regulation, and cell proliferation, consistent with previous studies[40,41] (Fig. 3e). Motif enrichment analysis of differentially accessible chromatin regions identified significant enrichment for binding motifs of the AP-1 and FOS transcription factor families, along with their dimeric complexes (Fig. 3f), corroborating previous reports[42,43]. Moreover, the motif-level DE patterns strongly agreed with those from the original study (Supplementary Fig. 22d).

To test whether the observed condition-specific T cell clustering was driven by these T cell–specific differentially expressed features (DEFs), we performed a feature ablation study. Specifically, we independently removed an equal number of T cell–specific DEFs identified by Palette and by Mimitou et al. from each modality. As a control, we also randomly removed the same number of features from each modality. We then integrated these three modified datasets using Palette and evaluated condition mixing of T cells using both k-nearest-neighbor batch effect test (kBET)[44] and local inverse Simpson's Index (LISI)[34] (Supplementary Fig. 22e–g). Removal of T cell–specific DEFs, particularly those identified by Palette, led to an increase in mixing between stimulated and control T cells (Fig. 3g), indicating that these features encode condition-associated T cell state variation. These results indicate that Palette not only supports effective modality alignment but also preserves fine-grained, condition-specific biological variation.

We further investigated whether Palette integration could disentangle condition-associated and condition-shared biological signals. After Palette-identified T cell–specific DEFs removal, we expect the primary effect of feature removal to be confined to T cell stimulation-derived state variation, while major lineage structure among immune populations should remain stable. For this purpose, we annotated T cells into five finer subtypes, with other immune populations largely preserved (Fig. 3h, i, and Supplementary Fig. 23a–c), resulting in a total of ten immune cell subtypes and high correspondence with the original annotations at the level of major immune lineages (Supplementary Fig. 23d, e).

Comment: The 2 distinct NK clusters were already apparent before removing Palette-identified DEFs (Fig 3b).

Response: We thank the reviewer for this helpful comment. We agree that the NK1 and NK2 subclusters are already visible before T cell–specific DEF removal (Fig. 3b). Our goal was not to suggest that these NK subclusters emerge as a result of feature removal, but to show that their separability is preserved after removing T cell–specific DEFs. This preservation indicates that Palette-identified T cell–specific DEFs primarily capture stimulation-driven variation within T cells, while retaining intrinsic heterogeneity in other immune populations. We have revised the **Results** section accordingly to clarify this point (*lines 228–230*).

Modified Results (lines 228-230):

Notably, the two natural killer (NK) cell subclusters (NK1 and NK2) remained clearly separable after removing T cell–specific DEFs (Fig. 3j), indicating that lineage-intrinsic heterogeneity unrelated to the stimulation condition was retained.

Comment: Clarification of Supervised vs. Unsupervised Mode in Spatial Integration

In lines 311–325, the authors evaluate Palette on a mosaic integration task using low-resolution spatial multimodal data from human tonsil sections. However, it is not specified whether the analysis was performed using the supervised or unsupervised mode of Palette.

If supervised integration was conducted, which cell type labels were used?

Response: We thank the reviewer for this constructive comment. We clarify that the low-resolution spatial multimodal data experiment was performed using the unsupervised mode of Palette. In this setting, the original expert manual histological annotations were used exclusively for quantitative evaluation and were not provided to Palette during integration. We have explicitly clarified the use of the unsupervised mode for Palette in the **Results** section of the revised manuscript (*line 331*).

Modified Results (lines 331-332):

We then evaluated the performance of Palette on the unsupervised mosaic integration scenario for the low-resolution spatial multimodal dataset.

Comment: Single-modality Palette integration performance

The authors demonstrate Palette's performance in cross-species single-cell RNA integration using both orthologous and non-orthologous genes, showing strong results compared to state-of-the-art methods. However, since Palette employs Bi-sPCA for intra-modality batch correction and dimensionality reduction, it would be valuable to explicitly evaluate how Palette performs as a standalone unimodal batch correction method for RNA data outside the scope of cross-species integration (Suppl Fig 25). Including this benchmark against established single-modality tools would strengthen the paper by showing whether Palette also contributes meaningfully to RNA-only batch correction tasks, beyond

its multimodal integration capabilities, or whether users should use a different method for RNA-only integration.

Response: We thank the reviewer for this constructive suggestion. In response, we performed a systematic benchmark to evaluate Palette as a standalone unimodal batch correction method for scRNA-seq data, outside the context of cross-species integration.

Specifically, we compared Palette (in both supervised and unsupervised modes) with the state-of-the-art single-modality integration methods, including unsupervised (STACAS, Seurat v3, fastMNN, and Harmony), supervised (scPoli, scANVI, and SIGNAL), and semi-supervised (ssSTACAS) approaches, across three real scRNA-seq datasets (MTG, immune, and pancreas). Quantitative results show that Palette achieves competitive performance in RNA-only integration tasks (Supplementary Fig. 39). Briefly, both the supervised and unsupervised modes of Palette perform on par with corresponding leading methods across multiple integration metrics. These results demonstrate that, beyond its multimodal integration capabilities, Palette also provides a viable and competitive solution for RNA-only batch correction.

The full benchmarking results have reported in **Supplementary Note 2** of the revised Supplementary Information.

Added Supplementary Figure:

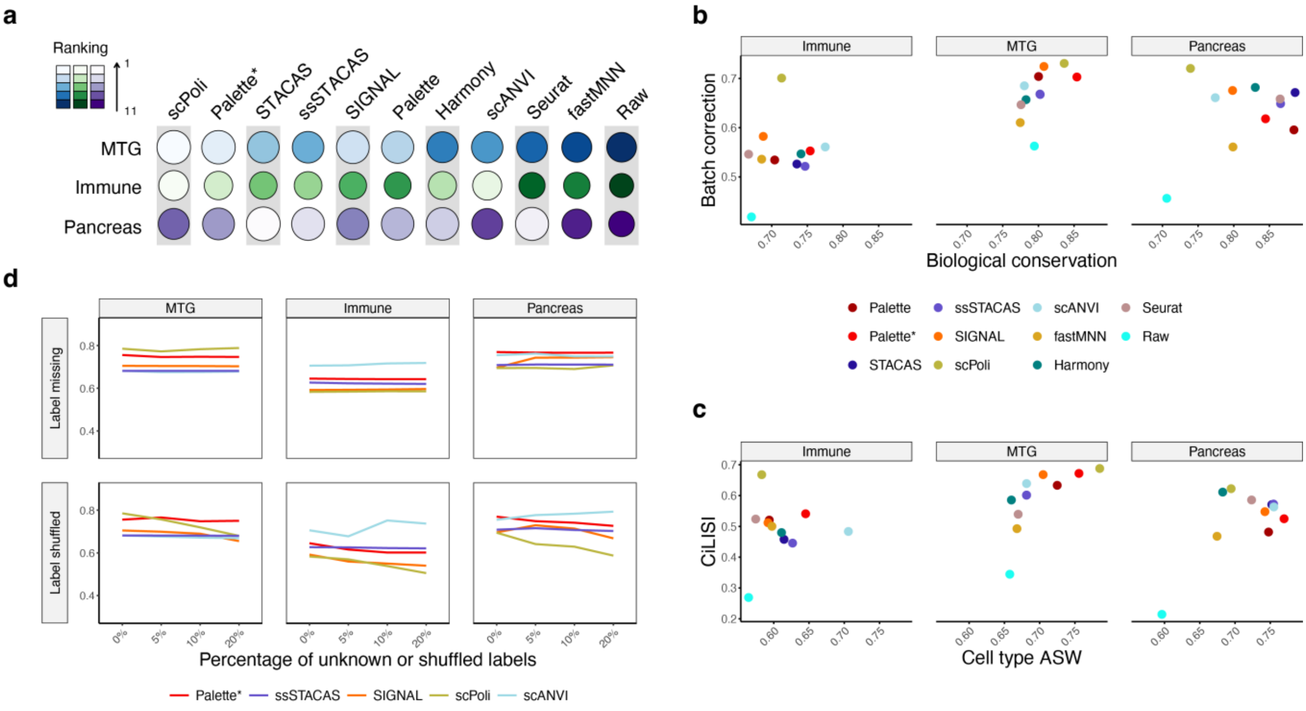

Supplementary Figure 39. Benchmarking of Palette against state-of-the-art integration methods for scRNA-seq data integration. a. Overall integration performance ranking of all methods across multiple datasets. The radius of each circle indicates the overall integration scores, ranging from 0 to

1. **b.** Dot plots comparing biological conservation scores and batch-correction scores for different integration methods across datasets. **c.** Dot plots comparing cell type ASW and CiLISI values for different integration methods across datasets. **d.** Cell type ASW values under increasing proportions of unknown (top row) and shuffled (bottom row) cell-type labels across multiple datasets. The supervised mode of Palette model is denoted as 'Palette*', whereas 'Palette' refers to the unsupervised mode.

Added Supplementary Note:

Supplementary Note 2. Evaluation of Palette horizontal integration on scRNA-seq data

To evaluate Palette's horizontal integration performance, we compared it with representative state-of-the-art single-cell integration algorithms. These included unsupervised methods (fastMNN[2], Seurat v3[3], Harmony[4], and STACAS[5]), supervised methods (scANVI[6], scPoli[7], and SIGNAL[8]), and the semi-supervised method ssSTACAS[9]. Since scRNA-seq data remains the most widely used modality for horizontal integration, we benchmarked all algorithms, and with Palette in both supervised and unsupervised modes, across three real scRNA-seq datasets (MTG, immune, and pancreas)[10,11].

Overall integration performance showed that scPoli, Palette supervised mode, and STACAS were the top-performing methods across the three tasks (Supplementary Fig. 39a). Among the unsupervised approaches, Palette unsupervised mode ranked second. We also observed that both modes of Palette achieved high biological conservation scores (Supplementary Fig. 39b). Following Andreatta et al.[9], we further examined CiLISI and cell type ASW, two metrics considered more suitable for evaluating batch correction and biological conservation. Using these metrics, Palette still maintained high biological conservation (cell type ASW) scores across datasets (Supplementary Fig. 39c).

We next assessed the sensitivity of supervised and semi-supervised methods to the quality and completeness of cell type labels. To this end, we perturbed labels by randomly shuffling or masking 5%, 10%, and 20% of the cell type annotations, and re-ran the supervised and semi-supervised algorithms using these modified labels. ssSTACAS showed the highest robustness in both perturbation scenarios, maintaining stable cell type ASW scores under increasing label noise and missing (Supplementary Fig. 39d). Palette preserved stable performance under increasing levels of label missing, with slightly higher sensitivity to label shuffling compared to ssSTACAS.

Added Supplementary Methods:

Transcriptomics data horizontal integration methods. We benchmarked Palette with both unsupervised and supervised integration mode against eight data integration methods. scANVI[6] and scPoli[7] were executed using the Python packages 'scvi' (v1.0.4) and 'scarches' (v0.5.9), respectively. Seurat v3[3], Harmony[4], fastMNN[2], and SIGNAL[8] were executed using the R packages 'Seurat' (v4.4.0), 'harmony' (v1.2.0), 'batchelor' (v1.14.1), and 'SIGNAL' (v1.0.0), respectively. Both ssSTACAS[9] and

STACAS[5] were run using the 'STACAS' (v2.3.0) R package. All methods were benchmarked using 20-dimensional integrated embeddings. Except where noted, all methods were run with their default integration parameters.

Modified Discussion (lines 458-463):

Benefiting from its modular design, Palette extends beyond mosaic integration to support horizontal, rectangular, and diagonal integration tasks. We first benchmarked Palette against state-of-the-art unsupervised and supervised horizontal integration methods across diverse scRNA-seq datasets[30,96] (Supplementary Fig. 39, and Supplementary Note 2). As part of this evaluation, the semi-supervised algorithm ssSTACAS and its unsupervised counterpart STACAS[97] were also included.

Modified Methods (lines 885-891):

Horizontal and rectangular integration datasets. We compiled three datasets for single-cell scRNA-seq data integration benchmarking from two studies. The human MTG scRNA-seq dataset from Suresh et al.[96], comprising five batches and a total of 137,303 cells. We selected 4,000 HVGs as features and retained all cells without additional quality control. The human immune and pancreas datasets from Luecken et al.[30], comprising ten batches with 33,506 cells and nine batches with 16,382, respectively. For both datasets, we selected 2,000 HVGs as features and retained all cells without additional quality control.

Added Reference:

97. Andreatta, M. & Carmona, S. J. STACAS: Sub-type anchor correction for alignment in Seurat to integrate single-cell RNA-seq data. *Bioinformatics* **37,** 882–884 (2021).

Comment: Code review and reproducibility

The provided R package (https://github.com/qiongyusheng/Palette) is not readily installable, as it has issues with BiocManager and lacks the required biocViews field in the package description. In addition, the data needed to run the tutorials is only available upon request, limiting transparency and accessibility for independent validation. The accompanying reproducibility repository (https://github.com/qiongyusheng/Palette_reproducibility) contains incomplete code and no accessible datasets, preventing full replication of the published results. The authors should ensure that all code and data required to reproduce the main figures and benchmarks are publicly available and executable without additional data requests.

Response: We thank the reviewer for carefully evaluating the code availability and reproducibility of our work. In response, we have improved both the accessibility and completeness of the released code and data.

First, we have fixed the installation issues of the R package (https://github.com/qiongyusheng/Palette), and the package installation procedure is now clearly documented in the GitHub README.

Second, all data required to run the tutorials is now publicly available and no longer requires individual requests. The corresponding data access links are provided in the GitHub README.

Third, we have updated and expanded the accompanying reproducibility repository (https://github.com/qiongyusheng/Palette_reproducibility) to include the code corresponding to the major analyses reported in the manuscript, together with a detailed description of the code structure in the GitHub README.

Finally, the processed datasets used in the study have been deposited in Zenodo and are publicly accessible (https://doi.org/10.5281/zenodo.18045027), enabling independent reproduction of the main figures and benchmarking results without additional data requests.

Comment: Minor comments

Supplementary Figure 20: No inclusion of plot c) in foot note.

Response: We thank the reviewer for pointing this out. We have revised the caption (footnote) of Supplementary Figure 20 (Supplementary Figure 22 in the revised Supplementary Information) to include panel c), and all panels are now consistently described in the revised Supplementary Information.

Revised Supplementary Figure caption:

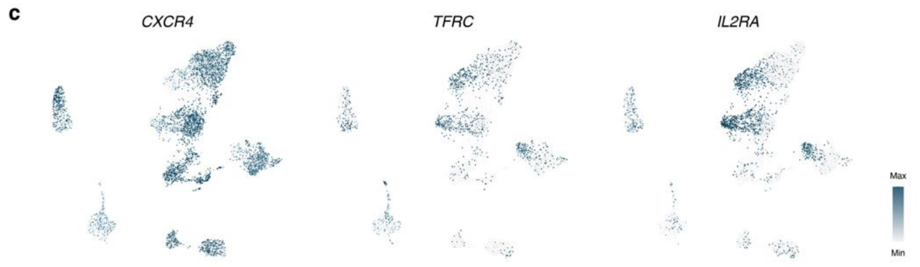

Supplementary Figure 22. Palette enables the preservation of condition-specific biological signals during mosaic integration. c. UMAP visualizations of expression patterns of condition-related DE genes.

Comment: (Remarks on code availability)

The provided R package (https://github.com/qiongyusheng/Palette) is not readily installable, as it has issues with BiocManager and lacks the required biocViews field in the package description. In addition, the data needed to run the tutorials is only available upon request, limiting transparency and accessibility for independent validation. The accompanying reproducibility repository

(https://github.com/qiongyusheng/Palette_reproducibility) contains incomplete code and no accessible datasets, preventing full replication of the published results

Response: We thank the reviewer for carefully evaluating the code availability and reproducibility of our work. In response, we have improved both the accessibility and completeness of the released code and data.

First, we have fixed the installation issues of the R package (https://github.com/qiongyusheng/Palette), including the previously reported BiocManager-related issue. The package installation procedure is now clearly documented in the GitHub README.

Second, all data required to run the tutorials is now publicly available and no longer requires individual requests. The corresponding data access links are provided in the GitHub README.

Third, we have updated and expanded the accompanying reproducibility repository (https://github.com/qiongyusheng/Palette_reproducibility) to include the code corresponding to the major analyses reported in the manuscript, together with a detailed description of the code structure in the GitHub README.

Finally, the processed datasets used in the study have been deposited in Zenodo and are publicly accessible (https://doi.org/10.5281/zenodo.18045027), enabling independent reproduction of the main figures and benchmarking results without additional data requests.

Point-by-point responses to the comments of Reviewer #3

Comment: I co-reviewed this manuscript with one of the reviewers who provided the listed reports. This is part of the Nature Communications initiative to facilitate training in peer review and to provide appropriate recognition for Early Career Researchers who co-review manuscripts.

Response: We thank the co-reviewer for their contribution to the peer-review process and for the code-related feedback provided in the report. In response to the reviewer's feedback, we have updated the codebase accordingly to address all the points raised. Please see our detailed point-by-point response below.

Comment: (Remarks on code availability)
R package: https://github.com/qiongyusheng/Palette.
- Not readily installation, issues with BiocManager: not `biocViews` included in description.
- Data for taking the tutorials only upon request.

Response: We thank the reviewer for carefully evaluating the code availability and tutorial data of our work. In response, we have improved both the accessibility and completeness of the released code and tutorial data.

We have fixed the installation issues of the R package (https://github.com/qiongyusheng/Palette), including the previously reported BiocManager-related issue. The package installation procedure is now clearly documented in the GitHub README.

In addition, all data required to run the tutorials is now publicly available and no longer requires individual requests. The corresponding data access links are provided in the GitHub README.

Comment: Results reproducibility: https://github.com/qiongyusheng/Palette_reproducibility.
- Incomplete code and no available data to reproduce results.

Response: We thank the reviewer for carefully evaluating the code reproducibility and data accessibility of our work.

In response, we have updated and expanded the accompanying reproducibility repository (https://github.com/qiongyusheng/Palette_reproducibility) to include the code corresponding to the major analyses reported in the manuscript, together with a detailed description of the code structure in the GitHub README.

In addition, the processed datasets used in the study have been deposited in Zenodo and are publicly accessible (https://doi.org/10.5281/zenodo.18045027), enabling independent reproduction of the main figures and benchmarking results without additional data requests.

Point-by-point responses to the comments of Reviewer #4

Comment: Sheng et al present Palette, a computational tool for mosaic integration of multi-modal single-cell datasets. They provide a detailed description of the mathematical principles behind the method, benchmark it against existing methods, and demonstrate its applicability in a variety of scenarios. Overall, the manuscript is very strong and the tool addresses an important problem in the field of single cell genomics. However there are a few concerns, addressing which would further strengthen this work:

Response: We thank the reviewer for the positive overall assessment of our manuscript and for the constructive suggestions to further strengthen the work. In response, we have carefully revised the manuscript to address all the points raised. The major revisions prompted by the reviewer's comments are summarized below.

(1) We have added a runtime and memory scalability analysis of Palette across multiple integration settings.
(2) We have included an additional experiment examining the trade-off between integration path length and the sparsity of intermediate modalities.
(3) We have added detailed justification for parameter choices and practical guidance for extending Palette to new modalities.
(4) We have added a benchmarking analysis for missing modality inference.

For more details, please see our point-by-point response below. For the reviewer's convenience, the corresponding revised manuscript is attached at the end of each response and is underlined to facilitate distinction from the response text.

Comment: Could the authors provide data on how computational resource requirements and runtime change with dataset size?

Response: We thank the reviewer for this helpful comment. To assess how computational resource requirements scale with dataset size, we evaluated the runtime and peak memory usage of Palette integration under progressively increasing numbers of cells. The analysis was performed for trimodal integration (ATAC + RNA + ADT) as well as for two bimodal settings (RNA + ADT and ATAC + RNA), while keeping default model parameters fixed.

We have added the details of runtime and memory scalability analysis in the **Methods** of the revised manuscript (*lines 1010-1025*), and the corresponding results have presented in **Supplementary Fig. 50** of the revised Supplementary Information.

Added Supplementary Figure:

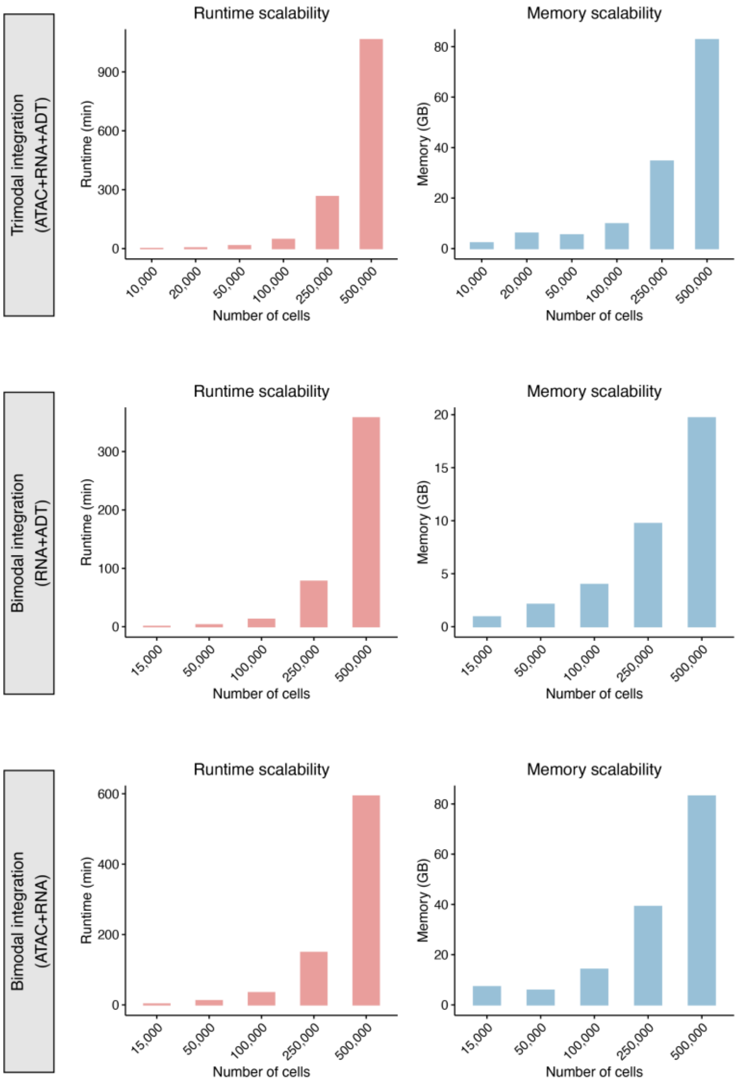

Supplementary Figure 50. Computational scalability of Palette integration. Runtime (left column) and peak RAM (right column) used for Palette integration under increasing cell numbers. Results are shown for trimodal integration (top row) and for two bimodal settings: RNA + ADT (middle row) and ATAC + RNA (bottom row).

Added Methods (lines 1010-1025):

Runtime and memory scalability analysis. We evaluated the scalability of Palette by increasing the number of cells under different modality compositions while using default parameter settings. Two bimodal scenarios were created from human BMMC CITE-seq (RNA and protein) and 10x Multiome (RNA and ATAC) datasets, and a trimodal scenario (RNA, protein, and ATAC) was generated by combining the two technologies. The transcriptome, proteome, and chromatin-accessibility modalities were represented by 3,000 highly variable genes, 134 surface proteins, and 113,709 peaks, respectively. Datasets of different sizes were constructed by subsampling or oversampling cells (15k–500k for

bimodal, 10k–500k for trimodal). In all scenarios, each batch contained 5,000 cells, so the batch number scaled with the dataset size. In the trimodal task, the CITE-seq and 10x Multiome datasets were constrained to have equal sample sizes in each test. In bimodal tasks, 20% of batches were assigned to the full multimodal subset and 40% to each unimodal subset, except in the 15k cell dataset, where only three batches were available and thus evenly divided. Runtime and peak memory usage were recorded using the peakRAM (v1.0.2) R package. All experiments were conducted on a Linux workstation equipped with an AMD EPYC 7513 CPU (1.50 GHz) and 256 GB of RAM. The results of this analysis are available in Supplementary Fig. 50.

Comment: Could the authors include a discussion on the tradeoff between the length of propagation path and the sparsity of modalities that lie on a path? E.g. is it better to follow a longer path if the shortest path means traversing through one or more extremely sparse data modalities?

Response: We thank the reviewer for this constructive comment. To address the trade-off between propagation path length and the sparsity of intermediate modalities, we performed a targeted simulation analysis. Using the human PBMC TEA-seq dataset, we constructed a simulated mosaic integration setting with multiple modalities exhibiting varying degrees of sparsity (Supplementary Fig. 43a).

For each missing modality, we evaluated multiple propagation strategies after quantifying the sparsity of each modality. In addition to Palette's default path selection, we compared (i) relatively shorter paths that traverse a larger number of sparser modalities and (ii) relatively longer paths that traverse a larger number of denser modalities. Missing data were inferred along each path and subsequently processed using the standard Palette integration procedure.

Quantitative evaluation across multiple metrics showed that Palette's default path selection achieved the best overall performance, and that shorter paths generally outperformed longer paths even when the shorter paths traversed relatively more sparse modalities (Supplementary Fig. 43 b). These results support the design choice of prioritizing shorter propagation paths in Palette.

We have added more details about the trade-off between the length of the propagation path and the modality sparsity in **Supplementary Note 3** of the revised Supplementary Information.

Added Supplementary Figure:

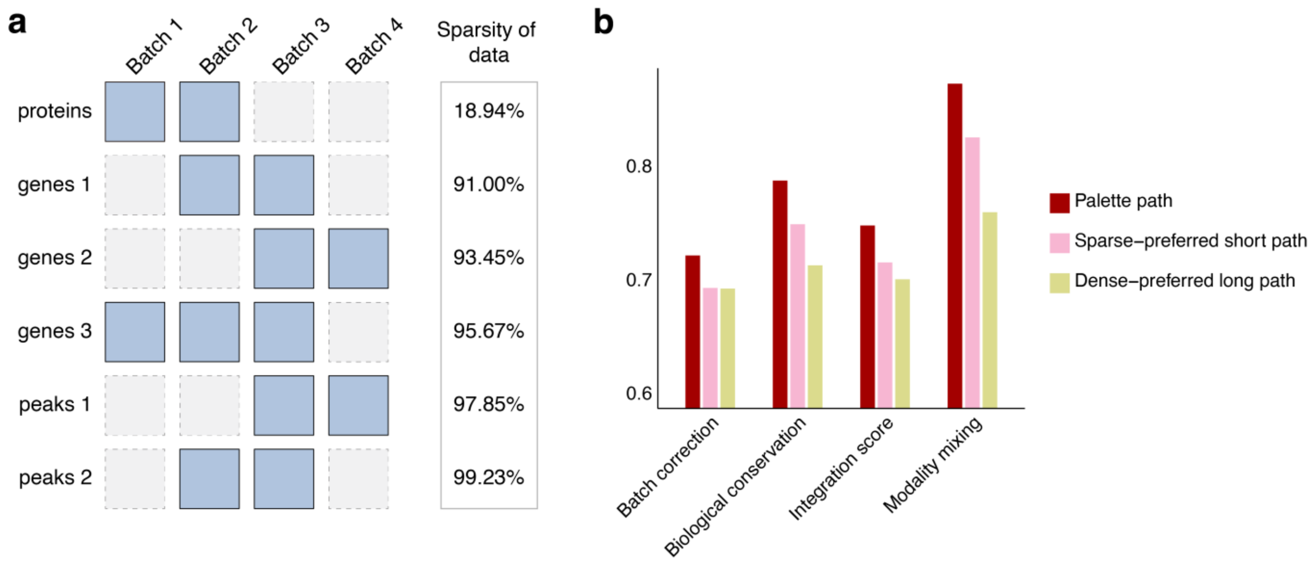

Supplementary Figure 43. Evaluating the trade-off between path length and intermediate-modality sparsity in integration. a. Schematic of the modality composition in the simulated data based on human PBMC TEA-seq dataset. The sparsity levels of each simulated modality are shown on the right. **b.** Quantitative comparison of different path selection strategies, with performance reported separately across four evaluation metrics: batch correction, biological conservation, overall integration, and modality mixing.

Added Supplementary Note:

Supplementary Note 3. Path selection evaluation under varying modality sparsity

Here, we used the human PBMC TEA-seq dataset to generate three distinct gene sets and two distinct peak sets through different feature selection strategies (Supplementary Methods), thereby constructing a six-modality mosaic integration scenario (Supplementary Fig. 43a). We first quantified the sparsity of each modality. Then, for each missing modality, three propagation paths were evaluated: (1) Palette's default path selection mode, i.e., the shortest path, which may traverse either sparse or dense modalities; (2) relatively shorter paths that traverse a larger number of sparser modalities; and (3) relatively longer paths that traverse a larger number of denser modalities.

Quantitative evaluations of the integrated embeddings under these three paths showed that Palette's default path selection achieved the best performance across all metrics (Supplementary Fig. 43b). The shorter path traversing sparser modalities ranked second, performing comparably to the longer path traversing denser modalities in batch correction metrics and outperforming it in all remaining metrics. These results indicate that choosing the shorter available path is generally more effective than selecting longer paths, even when the shorter path traverses sparser modalities than those included in the longer path.

Added Supplementary Methods:

Path selection analysis. We simulated modality-specific expression matrices with different sparsity levels by selecting distinct feature sets from the scRNA-seq and scATAC-seq modalities of the human PBMC TEA-seq dataset. For scRNA-seq, feature selection was performed using the FindVariableFeatures function in the Seurat R package, with the selection.method parameter set to "mean.var.plot," "dispersion," and "vst," respectively, yielding three feature sets containing 1,676, 4,725, and 3,994 genes. For scATAC-seq, we used two feature selection strategies. First, we applied FindVariableFeatures in Seurat with selection.method = "vst" and retained peaks ranked between 50,001 and 100,000. Second, we used the FindTopFeatures function in the Signac R package (default parameters) and similarly retained peaks ranked between 50,001 and 100,000. These feature sets were used to construct modality matrices exhibiting varying degrees of sparsity.

Added Methods (lines 629-634):

Modality sparsity may potentially influence the effectiveness of path propagation, raising the possibility that a short path relying on sparse modalities could underperform compared with a longer path using denser modalities. To examine this potential trade-off, we evaluated how modality sparsity and path length may interact during propagation and how these factors relate to integration quality (Supplementary Fig. 43, and Supplementary Note 3). The results support the design of the Palette path selection strategy.

Comment: Could the authors provide their rationale for choosing specific values of T1, theta-m, lambda-m and other important, modality specific parameters? This will especially be helpful for users who wish to use this for new single-cell modalities.

Response: We thank the reviewer for this constructive comment. In response, for key parameters in the Palette framework, we have added explicit explanations of the rationale behind the default parameter settings and provided practical guidance for selecting appropriate values when applying Palette to new single-cell modalities. These parameters include the quantile threshold for selecting representative cells, the thresholds T_1 and T_2 for identifying similar clusters across batches, and the Bi-sPCA regularization parameter λ used at different stages of the integration workflow.

Specifically, we have described how the representative-cell quantile threshold trades off computational cost against robustness to noisy cells, how the cluster-matching thresholds T_1 and T_2 control the stringency of cross-batch cluster pairing in the unsupervised setting, and how the Bi-sPCA regularization parameter λ modulates the balance between suppressing unwanted technical variation and preserving biologically meaningful structure.

We have added explicit explanations of the role and practical considerations behind the default parameter settings used in this study, together with the guidance for adapting them to new datasets, in the **Supplementary Methods**.

Added Supplementary Methods:

An explanation of parameter selection in Palette. Palette integration involves several parameters that are specified at different stages of the workflow, including the quantile threshold for selecting representative cells, the thresholds T_1 and T_2 for identifying similar clusters across batches, and the Bi-sPCA regularization parameter λ used at different integration stages. Here, we explain the rationale behind the default settings of these parameters and provide practical guidance for selecting appropriate values when applying Palette to new data modalities.

Quantile threshold for representative cell selection. The quantile threshold determines the proportion of representative cells selected within each cell type or cluster, based on silhouette width (SW) rankings as described earlier. Higher quantile thresholds correspond to selecting fewer cells. This design serves two purposes: it reduces computational complexity and improves robustness to noisy or potentially mislabeled cells. For modalities where measurements are sparse and cell–cell separability is weak, using a higher quantile threshold (i.e., retaining fewer, more representative cells) can reduce the influence of ambiguous observations on downstream similarity estimation. For large-scale datasets, higher thresholds can also reduce runtime.

Global modality-specific thresholds T_1 and T_2. The thresholds T_1 and T_2 are jointly used to filter similar clusters across batches in the unsupervised setting. Specifically, T_1 acts as a global, modality-specific lower bound on cluster similarity, whereas T_2 is the cosine value of an adaptive angular constraint that accounts for within-cluster variability through the combination of the median cluster angle θ_c and a modality-specific offset θ_m. A cluster-pair similarity is retained only if it exceeds $T = \max\{T_1, T_2\}$. These two thresholds play complementary roles: T_1 controls a global similarity baseline across batches, while T_2 adjusts the similarity requirement according to the intrinsic structure of each cluster. In practice, when within-dataset separability and cross-batch consistency are strong, users may consider slightly more stringent settings (higher T_1, smaller θ_m); conversely, when signals are weak or sparse, more permissive settings can help avoid discarding potentially related clusters.

Bi-sPCA regularization parameter λ. The Bi-sPCA framework includes a regularization parameter that controls the balance between suppressing unwanted technical variation and preserving biologically meaningful signals. In Palette, λ is used in two distinct contexts.

During the joint dimensionality reduction stage, λ^m is specified in a modality-specific manner to account for differences in dimensionality and sparsity across modalities. Modalities with relatively dense feature-by-cell matrices and strong cell–cell separability, such as scRNA-seq and ADT, **often tolerate** moderately larger λ^m, whereas sparse modalities, such as scATAC-seq, **may require** more conservative settings to reduce the risk of over-correction. Pseudo-modality matrices **may also require**

slightly stronger regularization in some settings, and we therefore recommend starting from a conservative baseline and refining if needed.

In the subsequent cross-batch alignment stage, a single global λ parameter is used to regulate the suppression of batch-associated variation across modalities. While this parameter serves a different role from λ^m, its selection follows a similar principle: stronger regularization is appropriate when technical heterogeneity across batches is pronounced, while monitoring potential loss of biological structure.

Practical guidance for parameter selection on new modalities. While Palette provides robust default parameters for established modalities (ADT, scRNA-seq, and scATAC-seq), which were used for all benchmarks in this study, users may encounter novel or domain-specific modalities. When applying Palette to a new modality, we recommend a principled "warm-start then refine" strategy. If the new modality shares similar characteristics, including signal-to-noise ratio, dimensionality, and sparsity patterns, with an established modality, its default parameters should serve as the initial baseline. Otherwise, we suggest the following systematic procedure.

Quantile threshold for representative cell selection. In most cases, the default quantile threshold for representative cell selection is sufficient. For extremely large datasets or modalities with very sparse measurements and weak separability, this threshold can be moderately increased to stabilize representative cell selection.

Global modality-specific thresholds T_1 *and* T_2. If no established modality defaults are applicable, we recommend using the pseudo-modality defaults (described in the Methods). This configuration provides a permissive filtering regime intended to reduce the risk of over-filtering potentially informative cross-batch similarity signals for integration. Note that these parameters are effectively bypassed in supervised integration modes.

Bi-sPCA regularization parameter λ. If cell-type labels are available, tuning should be guided by the interplay between batch correction and biological conservation metrics (both described in the Methods). An observation of high batch correction scores coupled with low biological conservation may suggest over-correction, necessitating a decrease in λ^m. Conversely, persistent batch separation despite preserved biological structure suggests under-correction, warranting an increase in λ^m. In the absence of labels, we recommend using batch-wise clustering consistency, such as mean ASW for each batch on the integrated embedding, as a proxy for biological conservation.

Finally, for the global cross-batch alignment parameter λ, while the default value is designed to be conservative and robust, it can be further refined following the same dual-criterion principle as λ^m, balancing alignment strength against biological conservation. When labels are available, users can optimize λ by monitoring the modality mixing score (as defined in the Results) instead of the batch mixing score. Otherwise, the default setting generally provides a robust and conservative choice.

Comment: Could the authors provide quantitative assessment of inference accuracy for missing modalities by holding out specific modalities, in cases where multi-omic data is available. Does this performance vary significantly by modality?

Response: We thank the reviewer for this helpful comment. In response, we have conducted a systematic evaluation of Palette's modality inference performance. Specifically, we benchmarked Palette on five benchmark scenarios that support missing modality inference and compared its performance with three state-of-the-art methods: MIDAS, scVAEIT, and Multigrate. Following a recent comprehensive benchmark study (added Supplementary ref. 12), we evaluated inference quality using six established metrics that capture both data structure preservation and clustering consistency.

Across all evaluated tasks, Palette successfully reconstructed missing modalities and demonstrated competitive and stable performance relative to existing methods (Supplementary Figs. 44 and 45). Only one metric (standardized mean squared error, sMSE) was slightly worse than the best-performing method under the scATAC-seq data inference task. Collectively, the missing modality inference performance of Palette is stable across modalities.

We have provided more details about experimental setups, evaluation protocols, and metric definitions in **Supplementary Note 4** and **Supplementary Methods** of the revised Supplementary Information.

Added Supplementary Figures:

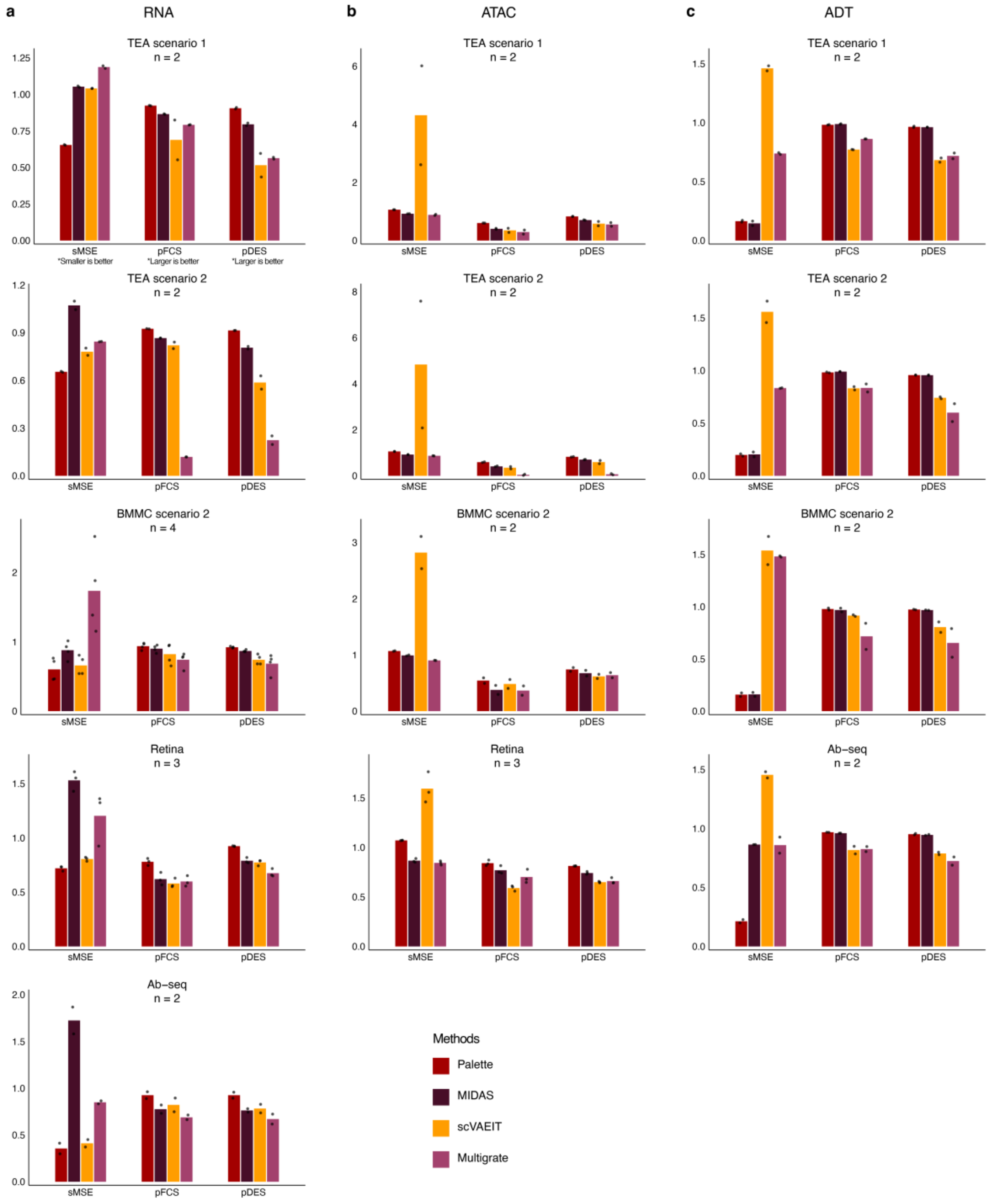

Supplementary Figure 44. Quantitative evaluation of inferred modality data using data structure metrics. Across five integration scenarios, we compared the modality inference performance of Palette with MIDAS, scVAEIT, and Multigrate using data structure metrics, including sMSE, pFCS, and pDES. For each task and each metric, bars indicate the mean performance across inferred batches, and individual points represent batch-level measurements. In addition, the number annotated in each panel denotes the number of batches inferred for the corresponding modality and task.

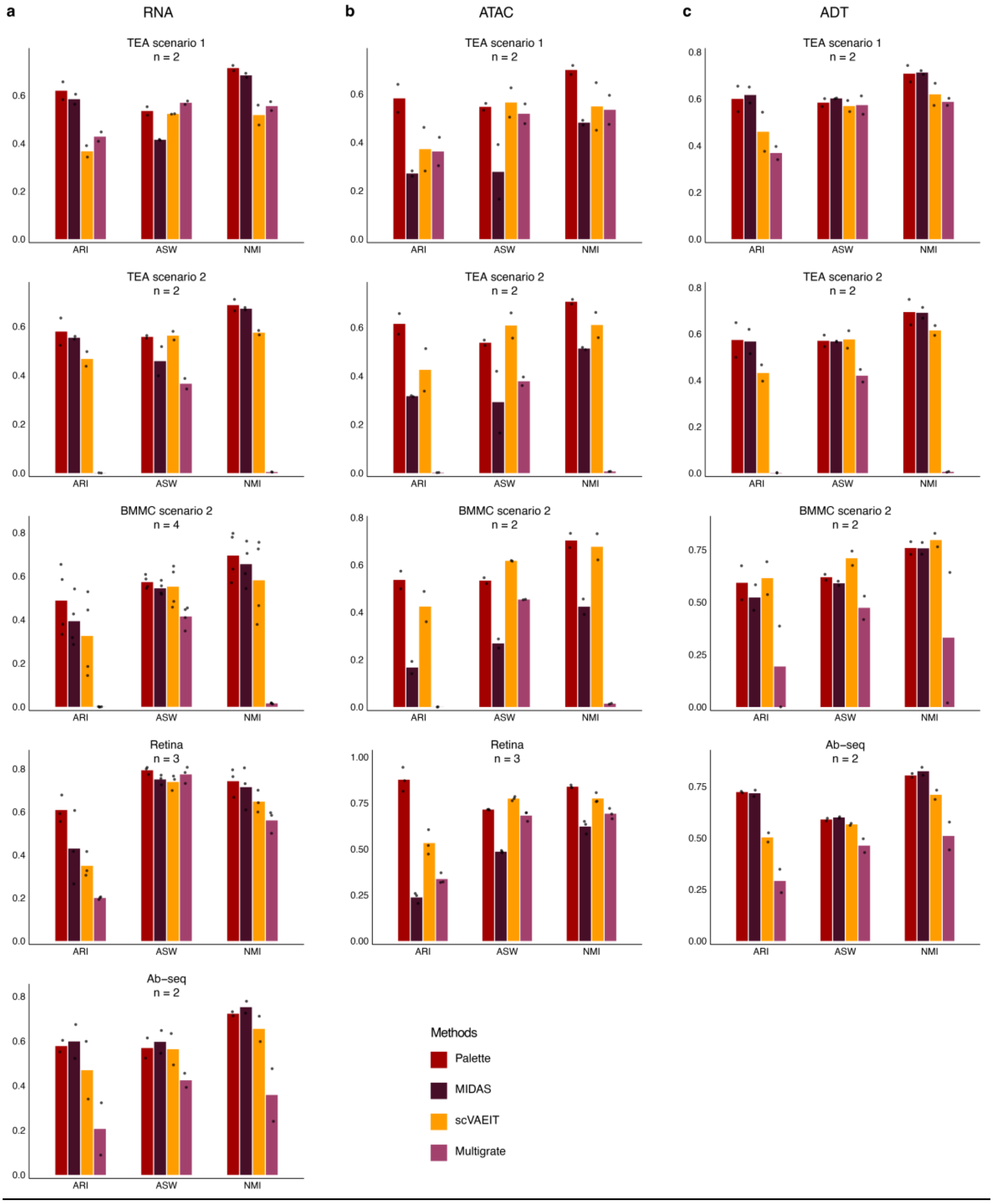

Supplementary Figure 45. Quantitative evaluation of inferred modality data using clustering-based metrics. Across five integration scenarios, we assessed modality inference performance for Palette, MIDAS, scVAEIT, and Multigrate using clustering-based metrics, including ARI, ASW, and NMI. For each task and each metric, bars indicate the mean performance across inferred batches, and individual points represent batch-level measurements. In addition, the number annotated in each panel denotes the number of batches inferred for the corresponding modality and task.

Added Supplementary Note:

Supplementary Note 4. Benchmarking modality inference performance

We evaluated missing-modality inference using five benchmark scenarios. BMMC Scenario 1 was excluded because the missing modality lacks real measurements and therefore cannot be quantitatively assessed. For each remaining scenario, one sub-experiment was selected for inference (Supplementary Methods). Palette was compared with three methods capable of performing missing-modality inference: MIDAS, Multigrate, and scVAEIT.

Inference performance was assessed in a modality-specific manner within each task using data structure and clustering-based metrics following a recent benchmark study[12] (Supplementary Methods). Across all scenarios and nearly all metrics, Palette either achieved the highest performance scores or exhibited performance comparable to the top-performing methods. An exception was observed for the standardized mean squared error (sMSE) metric on scATAC-seq data, where Palette did not rank among the top-performing methods (Supplementary Figs. 44 and 45). Overall, these results indicate that Palette enables accurate and reliable missing modality inference across diverse multimodal settings.

Added Supplementary Methods:

Modality inference analysis. We conducted missing-modality inference experiments using five of the six benchmark tasks that support this evaluation. For each task, one sub-experiment was randomly selected for inference (TEA Scenario 1: 2; TEA Scenario 2: 3; BMMC Scenario 2: 1; Retina: 3; Ab-seq: 4). Palette was compared with three methods designed for missing-modality inference: MIDAS, scVAEIT, and Multigrate. To ensure consistency across inferred outputs, we used the top 50,000 highly variable scATAC-seq peaks as input for all tasks except Ab-seq. For a given modality within a task, inference was performed across multiple batches. Performance was first evaluated separately for each batch using the corresponding metrics, and batch-level values were then averaged to obtain a single modality-level performance score used for comparison across methods and tasks.

Modality inference metrics. We quantitatively evaluated modality inference performance using the evaluation metrics described by Liu et al.[12], which cover both data structure metrics and clustering-based evaluation metrics. The data structure metrics include sMSE relative to the ground-truth data, preservation of feature correlation structure (pFCS), and preservation of differential expression statistics (pDES).

sMSE. We computed the standardized mean squared error between the inferred and ground-truth matrices, with lower values indicating closer agreement.

pFCS. For the inferred and ground-truth data, we independently selected highly variable features (100 for scRNA-seq and ADT, and 1,000 for scATAC-seq). Using each dataset's own feature set, we computed cell–cell correlation matrices and quantified preservation as the mean Pearson correlation

between the two matrices.

pDES. For each cell type, DE analysis was performed separately on the inferred and ground-truth data using limma (v3.54.2) to obtain DE feature sets. Cell–cell correlation matrices were then computed from each dataset using its corresponding DE features, and preservation was measured as the mean Pearson correlation between the two matrices.

For clustering-based evaluation, we used ARI, ASW, and NMI, as introduced earlier (Methods), to quantify the agreement between the Louvain-derived cluster labels and the ground-truth labels.

Added Supplementary Reference:

12. Liu, C., Ding, S., Kim, H.J. et al. Multitask benchmarking of single-cell multimodal omics integration methods. *Nat Methods* **22**, 2449–2460 (2025).

Comment: Could authors clarify the abbreviation TEA where it is first mentioned in the results and also explain what "randomly generated dataset" means (line 703). This will help simplify the text for readers.

Response: We thank the reviewer for this helpful comment. Regarding the term TEA, we clarify that TEA is not an acronym with an expanded full name, but follows the original naming convention of the TEA-seq technology, as introduced in the original study. We have revised the **Results** section to explicitly indicate the data source when TEA is first mentioned (*lines 110-111*).

With respect to the term "randomly generated dataset" (line 703), this wording referred to a specific sub-experiment defined under TEA scenario 1, for which the data generation procedure had already been described in the manuscript. In the revised version, we replaced this ambiguous phrasing with an explicit reference to the corresponding sub-experiment, so that the link between the description and the underlying experimental setup is clearer to readers (*line 746*).

Modified Results:

lines 107-111: We curated five multimodal single-cell datasets spanning both trimodal and bimodal modalities[9,28,29], constructing six mosaic integration tasks, four trimodal and two bimodal (Supplementary Figs. 2–4 and Methods). Specifically, for trimodal integration, we designed a full-modality single-hop and a multi-hop scenario using the TEA-seq technology data[9], corresponding to TEA scenarios 1 and 2, respectively.

Modified Methods:

lines 746-748: Parameters in Palette. We use the dataset of the third sub-experiment under the TEA scenario 1, as well as the human BMMC CITE-seq and 10x Multiome dataset, to evaluate the parameter sensitivity of Palette.

Comment: (Remarks on code availability):

The authors provide code on github to install their package and tutorials to sufficiently guide data analysis.

Response: We thank the reviewer for this positive assessment. We are pleased that the provided GitHub repository and accompanying tutorials are considered sufficient to support installation and data analysis using our package.

Point-by-point responses to the comments of Reviewer #1

Comment: (Remarks to the Author):

In their revised manuscript, the authors have now addressed my initial concerns regarding the validation of their method, its comparative benchmarking, and its overall methodological contribution.

Response: We thank the reviewer for the positive feedback about our work.

Comment: (Remarks on code availability):

The authors have now mapped the paper's analyses to the corresponding code and parameters, making the results reproducible.

Response: We thank the reviewer for the positive comment on the code reproducibility.

Point-by-point responses to the comments of Reviewer #2

Comment: (Remarks to the Author):

The authors have carefully addressed all our comments, providing a thorough and well-structured rebuttal and revising the manuscript accordingly.

The expanded evaluation strategy includes experiments under perturbed annotation settings, robustness and sensitivity analyses of modality mixing metrics, and single-modality integration benchmarks.

The revised manuscript now convincingly presents Palette as a valuable new tool for multi-modal dataset integration.

Response: We thank the reviewer for the positive feedback about our work. In response to the reviewer's feedback, we have revised the manuscript to address the points raised. Please see our detailed point-by-point response below.

Comment: A minor point remains for clarification:

Modified Methods (lines 885–891):

In the description of the horizontal and rectangular integration benchmarks, different numbers of highly variable genes (HVGs) are selected across datasets (4,000 HVGs for the MTG dataset versus 2,000 HVGs for the immune and pancreas datasets). It would be important to explain the rationale for using different number of HVG across these benchmarks. A brief justification (e.g., differences in dataset size, complexity, or consistency with prior studies) would help avoid potential confusion for readers.

Response: We thank the reviewer for this helpful comment. In the revised manuscript, we have clarified the rationale for using different numbers of HVGs across benchmarks. Briefly, the number of HVGs selected for each dataset was determined according to dataset complexity, which was defined by the number of cell types. The MTG dataset contains 24 cell types and exhibits higher biological complexity. Therefore, we selected the top 4,000 HVGs for integration. In contrast, the immune and pancreas datasets contain 16 and 14 cell types, respectively, and we selected the top 2,000 HVGs for integration. Within each benchmark, all compared methods were applied to the same HVG set, ensuring fair comparison.

We have added a clarification in the **Methods** section of the revised manuscript (*lines 828-835*) to make it clearer.

Modified Methods (lines 828-835):

Horizontal and rectangular integration datasets. We compiled three datasets for single-cell scRNA-seq data integration benchmarking from two studies. The human MTG scRNA-seq dataset from Suresh et al.[96], comprises five batches and 137,303 cells across 24 cell types. We selected 4,000 HVGs as features and retained all cells without additional quality control. The human immune and pancreas datasets from Luecken et al.[30], comprise ten batches with 33,506 cells across 14 cell types and nine batches with 16,382 across for 16 cell types, respectively. For both datasets, we selected 2,000 HVGs as features and retained all cells without additional quality control. The number of HVGs was determined according to dataset complexity, defined by the number of cell types.

Comment: (Remarks on code availability):

The package Palette is now installable and the tutorials are clear

Response: We thank the reviewer for the positive comment on the code implementation.

Point-by-point responses to the comments of Reviewer #3

Comment: I co-reviewed this manuscript with one of the reviewers who provided the listed reports. This is part of the Nature Communications initiative to facilitate training in peer review and to provide appropriate recognition for Early Career Researchers who co-review manuscripts.

Response: We thank the reviewer for the positive feedback about our work.

Point-by-point responses to the comments of Reviewer #4

Comment: (Remarks to the Author):

The authors have sufficiently addressed my comments, good luck to the team with the remaining publication process!

Response: We thank the reviewer for the positive feedback about our work.

Comment: (Remarks on code availability):

The authors provide code on github to install their package and tutorials to sufficiently guide data analysis.

Response: We thank the reviewer for the positive comment on the code implementation.
